# Polyketide Derivatives from Mangrove Derived Endophytic Fungus *Pseudopestalotiopsis theae*

**DOI:** 10.3390/md18020129

**Published:** 2020-02-23

**Authors:** Xiaoqin Yu, Werner E. G. Müller, Dieter Meier, Rainer Kalscheuer, Zhiyong Guo, Kun Zou, Blessing O. Umeokoli, Zhen Liu, Peter Proksch

**Affiliations:** 1 Institute of Pharmaceutical Biology and Biotechnology, Heinrich Heine University Duesseldorf, 40225 Duesseldorf, Germany; xiyu101@hhu.de (X.Y.); dieter.meier@hhu.de (D.M.); Rainer.Kalscheuer@hhu.de (R.K.); 2 Hubei Key Laboratory of Natural Products Research and Development, College of Biological and Pharmaceutical Sciences, China Three Gorges University, Yichang 443002, China; zhyguoctgu@foxmail.com (Z.G.); kzou@ctgu.edu.cn (K.Z.); 3 Institute of Physiological Chemistry, Universitätsmedizin der Johannes Gutenberg-Universität Mainz, 55128 Mainz, Germany; wmueller@uni-mainz.de; 4 Department of Pharmaceutical and Medicinal Chemistry, Nnamdi Azikiwe University, 420281 Awka, Nigeria; blessingumeokoli@gmail.com

**Keywords:** *Pseudopestalotiopsis theae*, endophytic fungus, polyketide, cytotoxicity

## Abstract

Chemical investigation of secondary metabolites from the endophytic fungus *Pseudopestalotiopsis theae* led to the isolation of eighteen new polyketide derivatives, pestalotheols I–Q (**1**–**9**) and cytosporins O–W (**15**–**23**), together with eight known analogs (**10**–**14** and **24**–**26**). The structures of the new compounds were elucidated by HRMS and 1D and 2D NMR data, as well as by comparison with literature data. Modified Mosher’s method was applied to determine the absolute configuration of some compounds. Compound **23** showed significant cytotoxicity against the mouse lymphoma cell line L5178Y with an IC_50_ value of 3.0 μM. Furthermore, compounds **22** and **23** showed moderate antibacterial activity against drug-resistant *Acinetobacter baumannii* (ATCC BAA-1605) in combination with sublethal colistin concentrations.

## 1. Introduction

Since the discovery of antibacterial penicillin from *Penicillium notatum*, there have been tremendous achievements with regard to the development of fungal drugs [1]. The antifungal medicine griseofulvin, which was initially isolated from *Penicillium griseofulvum*, has been applied for the treatment of ringworm in skin and nails in animals and humans [2]. Lovastatin, the first commercial statin isolated from a fermentation broth of *Aspergillus terreus*, has been used to treat high blood cholesterol and reduce the risk of cardiovascular disease [3]. The immunosuppressant agent cyclosporine was isolated from the fungus *Tolypocladium inflatum* [4]. Endophytic fungi occur in all plants investigated up to now and are important for the fitness and survival of their hosts [5]. The chemically remarkably creative fungal genus *Pestalotiopsis* contains around 234 described fungal species listed in Index Fungorum, including 44 taxol producers [6,7]. *Pestalotiopsis* has attracted considerable attention due to the abundance and diversity of secondary products found in members of this genus [8,9]. Pestalachloride A, a new chlorinated benzophenone alkaloid produced by *Pestalotiopsis adusta*, showed potent antifungal activity against *Fusarium culmorum* with an IC_50_ value of 0.89 μM [10]. The first chlorinated pupukeanane derivative, chloropupukeananin, which was isolated from fermentation broth of *Pestalotiopsis fici*, inhibited HIV-1 replication in C8166 cells with an IC_50_ value of 14.6 μM [11]. Pestaloquinols A and B, two unique isoprenylated epoxyquinol derivatives obtained from rice cultures of *Pestalotiopsis* sp., both exhibited cytotoxicity against HeLa cell line with IC_50_ values of 8.8 μM [12]. Pestalotines A and B, two novel phytotoxic *γ*-lactonic dimers from *Pestalotiopsis* sp., showed potent phytotoxicity against the radical growth of *Echinochloa crusgalli* with IC_50_ values of 0.19 and 0.25 μM, respectively [13].

**Figure 1:**
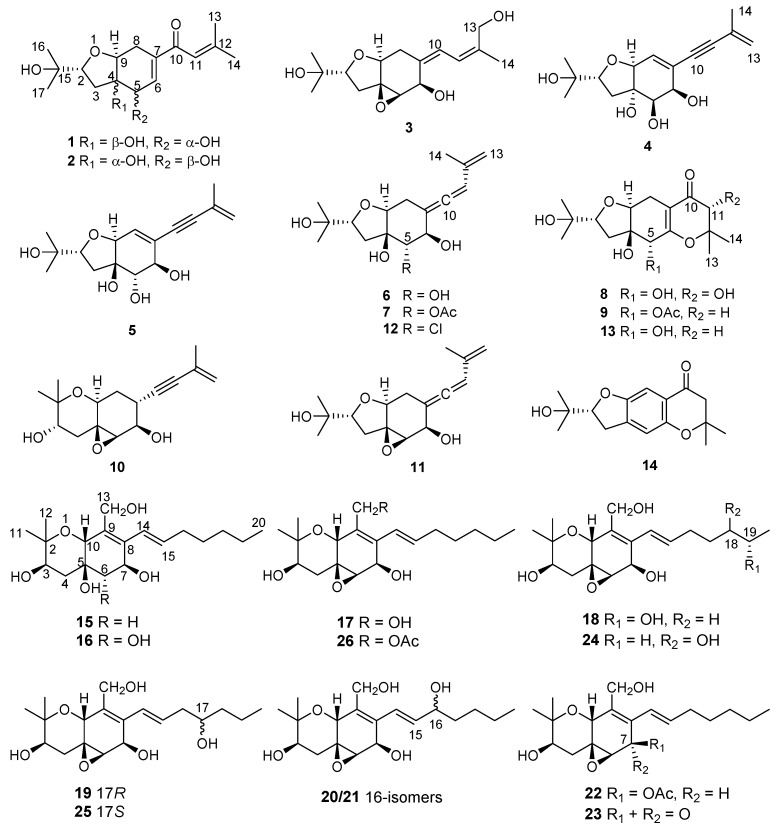
Structures of compounds **1**–**26** isolated from *Pseudopestalotiopsis theae*.

In previous studies from our group, cytotoxic 14-membered macrolides were obtained from the mangrove-derived fungus *Pestalotiopsis microspora* with IC_50_ values ranging from 0.7 to 5.6 μM [14]. In the present study, the endophytic fungus *Pseudopestalotiopsis theae*, which was previously reported as *Pestalotiopsis theae* and taxonomically revised in 2014 [7] was isolated from roots of the mangrove plant *Rhizophora racemosa* collected around Lagos, Nigeria. Fermentation of this fungus on solid rice medium yielded eighteen new polyketides, namely pestalotheols I–Q (**1**–**9**) (Supplementary information) and cytosporins O–W (**15**–**23**), in addition to eight known congeners (Figure 1). In this paper, the isolation, structure elucidation, and bioactivity of all isolated compounds are reported.

## 2. Results and Discussion

**Figure 2:**
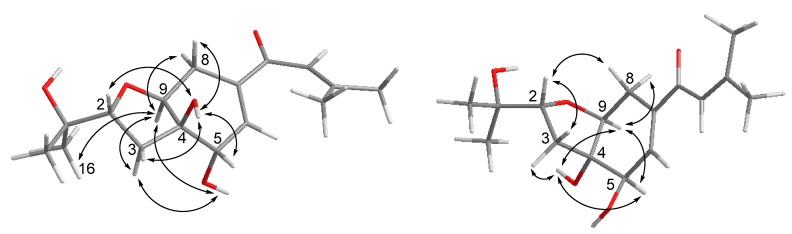
Key NOE correlations for compound **1** (left) and **2** (right).

**Figure 3:**
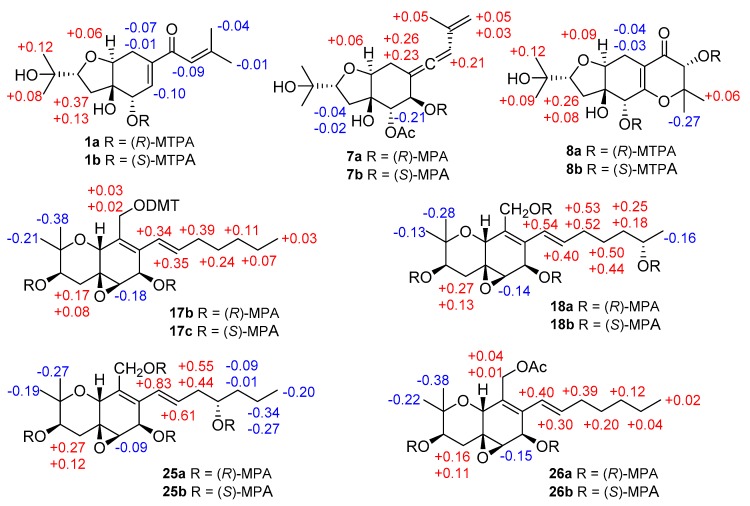
∆*δ* = (*δ_S_* − *δ_R_*) values (in ppm) for the methoxytrifluoromethylphenylacetic acid (MTPA) esters of **1** and **8**, ∆*δ* = (*δ_R_* − *δ_S_*) values (in ppm) for the MPA esters of **7**, **17**, **18**, **25**, and **26**.

Compound **1** was obtained as yellowish gel. The molecular formula of **1** was determined as C_16_H_24_O_5_ by HRESIMS data, indicating five degrees of unsaturation. The ^13^C NMR data (Table 1) for **1** revealed the presence of a carbonyl at *δ*_C_ 191.9 (C-10), four olefinic carbons at *δ*_C_ 153.7 (C-12), 138.7 (C-7), 138.0 (C-6), and 120.7 (C-11), five oxygenated carbons at *δ*_C_ 84.2 (C-2), 77.8 (C-4), 76.3 (C-9), 70.8 (C-15), and 68.9 (C-5), two methylene groups at *δ*_C_ 34.0 (C-3) and 25.7 (C-8), together with four methyls at *δ*_C_ 27.2 (C-14), 25.9 (C-17), 25.8 (C-16), and 20.5 (C-13), accounting for three degrees of unsaturation. Thus, compound **1** was suggested to be bicyclic in nature. The COSY correlations between H-2/H-3ab, OH-5/H-5/H-6, H-8/H-9, together with the HMBC correlations from H-2 to H-9, from OH-4 to C-3, C-4, C-5, and C-9, from H-5 to C-4 and C-9, from H-6 to C-4 and C-8, and from H-8ab to C-4, C-6, C-7, and C-9, established the presence of a tetrahydrofuranocyclohexene moiety with two hydroxy groups at C-4 and C-5. Additional HMBC correlations from Me-14 to C-11, C-12, and C-13, and from H-11, H-6 and H-8a to C-10 indicated the attachment of a 3-methyl-1-oxobut-2-en-1-yl side chain at C-7. Moreover, the location of a 2-hydroxyisopropyl subunit at C-2 was confirmed by the HMBC correlations from Me-16(17) to C-2, C-15 and C-17(16), and from OH-15 to C-2, C-15, C-16, and C-17. Thus, the planar structure of **1** was established as shown. The relative configuration of **1** was elucidated by coupling constants and NOE correlations (Figure 2). The large values of *J*_H-2/H-3a_ (9.9 Hz) and *J*_H-9/H-8b_ (10.7 Hz) suggested trans-diaxial orientation of these protons. The NOE correlations from H-9 to Me-16, H-3a, OH-5, and H-8a, and between H-3a and OH-5 indicated these protons to be on the same side, whereas the NOE correlations from OH-4 to H-2, H-3b, H-5, and H-8b confirmed they were in opposite orientation. Based on the summarized results (Figure 3) of the modified Mosher’s method, the absolute configuration of C-5 was determined to be *S*, thereby assigning 2*R*, 4*R*, 5*S*, 9*S* absolute configuration for pestalotheol I (**1**).

Based on the HRESIMS result as well as 1D and 2D NMR data (Table 1), the gross structure of **2** was elucidated as being identical to that of **1**. However, different NOE relationships were observed in the ROESY spectrum of **2** compared to **1** (Figure 2). The NOE correlations between H-9/H-8b, H-9/H-5, H-9/OH-4, OH-4/H-5, OH-4/H-3b indicated that these protons were on the α-face, whereas the NOE correlations from H-2 to H-3a and H-8a indicated they were β-oriented. Thus, the absolute configuration of **2** was tentatively assigned as 2*R*, 4*S*, 5*R*, 9*S* in comparison to **1**.

Pestalotheol K (**3**) has the molecular formula C_16_H_24_O_5_, containing one additional oxygen atom compared to truncateol Q, a polyketide analog isolated from *Truncatella angustata* [15]. The NMR data of **3** (Table 1) were similar to those of truncateol Q, except for the replacement of an olefinic methyl by an oxygenated methylene moiety at *δ*_C_ 60.9 and *δ*_H_ 4.21 and 4.16 (CH-13) and a hydroxy group at *δ*_H_ 3.70 (t, OH-13). The COSY correlation between OH-13/H-13ab and the HMBC correlations from H-13ab to C-11 (*δ*_C_ 122.8), C-12 (*δ*_C_ 139.9), and C-14 (*δ*_C_ 22.0) confirmed the location of this additional oxygenated methylene moiety at C-13. The remaining substructure and relative configuration of **3** were elucidated to be identical to truncateol Q after detailed analysis of the 2D NMR spectroscopic data of **3**.

The NMR data (Table 2) of pestalotheol L (**4**) were similar to those of truncateol K, a known polyketide containing a chloride atom at C-4 [16]. The molecular formula of **4** was determined as C_16_H_22_O_5_ by HRESIMS, suggesting the replacement of the chlorine atom at C-4 by a hydroxy group in **4**, which was supported by the observation of four hydroxy groups at *δ*_H_ 5.08, 5.05, 4.77, and 4.31 in the ^1^H NMR spectrum of **4** rather than three signals of hydroxy groups in truncateol K. On the basis of the 2D NMR spectroscopic data of **4**, the remaining substructure and relative configuration of **4** were determined to be identical to truncateol K.

Compound **5** has the same planar structure as **4** based on the 1D and 2D NMR spectroscopic data as well as on the HRESIMS data of **5**. However, the NOE interactions of **5** differed from those of **4** as also observed between **1** and **2**. In the ROESY spectrum of **5**, correlations from H-9 to Me-16, H-3a and OH-5, and from OH-5 to H-3a and H-6 were found, indicating they were on the same side. On the other hand, the NOE correlations between H-3b/H-2, H-2/OH-4, OH-4/H-5, H-5/OH-6 placed those protons on the other side of the ring. Hence the relative configuration of **5** was elucidated as 2R*, 4R*, 5S*, 6R*, 9S* whereas that of **4** was 2R*, 4S*, 5R*, 6R*, 9S*.

The molecular formula of pestalotheol N (**6**) was deduced as C_16_H_24_O_5_ from the HRESIMS data. The NMR data (Table 2) of **6** were compatible with those of the co-isolated known compound truncateol H (**12**) except for the lack of the chloride atom at C-5 [16]. Instead, the COSY correlation between the D_2_O changeable proton at *δ*_H_ 5.28 and H-5 (*δ*_H_ 3.83) suggested C-5 to be hydroxylated. Similar NOE interactions were observed for **6** and **12**, indicating the relative configuration of **6** to be identical to that of **12**, for which the absolute configuration had previously been determined by Mosher’s reaction and by the X-ray data of its 6-epimer [16].

Pestalotheol O (**7**) was obtained as colorless oil. The molecular formula of **7** was determined as C_18_H_26_O_6_ by HRESIMS, containing an additional acetyl group when compared to **6**, which was further supported by the presence of a carbonyl carbon at *δ*_C_ 169.2 and a methyl group at *δ*_C_ 20.5 and *δ*_H_ 2.09 in the NMR data of **7** (Table 3). The location of this additional acetyl group at C-5 was evident from the HMBC correlation from H-5 (*δ*_H_ 5.01) to the carbonyl carbon of the acetyl group. Analysis of 1D and 2D NMR data of **7** concluded its remaining substructure to be the same as **6**. The absolute configuration of C-6 in **7** was determined as *R* by comparison of chemical shifts between its methoxyphenylacetic acid (MPA) esters (Figure 3).

Based on the HRESIMS data, the molecular formula of compound **8** was deduced as C_16_H_25_O_7_, containing one additional oxygen atom when compared to the coisolated known derivative, pestalotheol A (**13**) [17]. The COSY correlation between the D_2_O changeable proton at *δ*_H_ 4.30 and H-11 (*δ*_H_ 4.13) and the HMBC correlations from H-11 to C-10, C-12, C-13, and C-14 confirmed the replacement of the methylene group by a hydroxy group at C-11 in **8**. Compounds **8** and **13** share the same relative configuration at C-2, C-4, C-5, and C-9 as evident from their similar coupling constants and NOE relationships. Moreover, Mosher’s method was applied to determine the absolute configuration at C-5 and C-11 of **8**. Due to the influence of multiple MPA moieties, anomalous ∆*δ* values were observed for Me-13 (−0.27) and Me-14 (+0.06). The absolute configuration at C-5 and C-11 of **8** was tentatively assigned as *R* (Figure 3). Therefore, the 2*R*, 4*R*, 5*R*, 9*S,* and 11*R* absolute configuration was assigned for pestalotheol P (**8**).

The HRESIMS of pestalotheol Q (**9**) showed the pseudomolecular ion peak at 355.1754 [M + H]^+^, indicating the molecular formula of C_18_H_27_O_7_. The UV spectrum and the NMR data of **9** suggested it to be an analog of pestalotheol A [17], with addition of an acetyl group as evident from signals in the ^1^H and ^13^C NMR spectroscopic data at *δ*_H_ 2.09, *δ*_C_ 169.5 and 20.5. The HMBC correlations from H-5 (*δ*_H_ 5.28) to the carbonyl carbon of the acetyl group indicated the attachment of the acetyl group at C-5. Based on the similar coupling constants and NOE relationships, the relative configuration of **9** was suggested to be identical to that of **13**. Thus, pestalotheol Q (**9**) was elucidated to be 5-acetoxy derivative of pestalotheol A (**13**).

**Figure 4:**
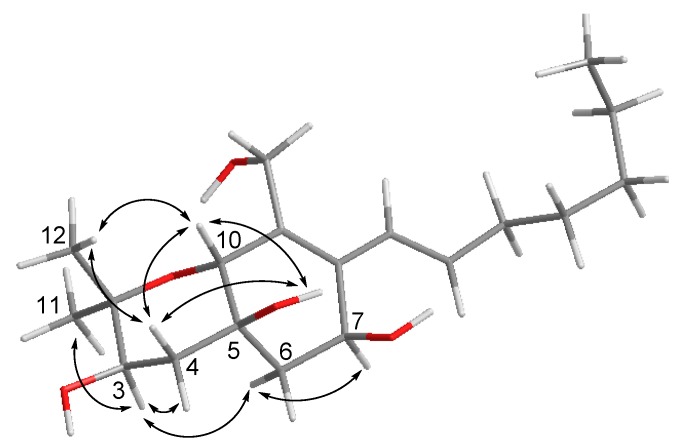
Key NOE correlations for compound **15**.

Cytosporin O (**15**) was obtained as yellowish gel. The molecular formula of **15** was established as C_19_H_32_O_5_ from the HRESIMS data, indicating four degrees of unsaturation. The ^13^C NMR data (Table 4) displayed signals of four olefinic carbons at *δ*_C_ 133.8 (C-8), 132.1 (C-15), 132.0 (C-9), and 126.0 (C-14), accounting for two degrees of unsaturation. Therefore, **15** was suggested to contain a bicyclic skeleton. In addition, six oxygenated carbons at *δ*_C_ 74.9 (C-2), 70.6 (C-3), 70.0 (C-10), 69.3 (C-5), 63.9 (C-7), and 57.7 (C-13), and three methyls at *δ*_C_ 27.9 (C-11), 16.1 (C-12), and 13.9 (C-20) were observed. Upon analysis of the 1D and 2D NMR data, **15** was suggested to be a derivative of cytosporin D [18] except for the replacement of signals of the epoxy ring at C5/C6 by an additional D_2_O exchangeable proton at *δ*_H_ 5.07 (s, OH-5) and an additional methylene group at *δ*_C_ 35.7, and *δ*_H_ 2.00, 1.54 (CH_2_-6). The HMBC correlations from OH-5 to C-4, C-5, C-6, and C-10, together with the COSY correlations between H-6ab and H-7, indicated a hydroxy group at C-5 and a methylene group at C-6. Thus, the planar structure of **15** was established as shown. The large coupling constant (15.7 Hz) revealed *E*-geometry of the double bond at C-15/C-16. The NOE interactions between H-10/OH-5, OH-5/H-4b, H-4b/H-10, H-10/Me-12, Me-12/H-4b indicated those protons to be orientated on the same side, whereas the NOE correlations between Me-11/H-3, H-3/H-4a, H-3/H-6a, H-6a/H-7 supported them to be on the opposite side (Figure 4).

Compound **16** has the molecular formula C_19_H_32_O_6_ as deduced from the sodium adduct ion at *m*/*z* 379.2094 [M + Na] ^+^ in HRESIMS, containing an additional oxygen atom when compared to **15**. The NMR data (Table 4) of **16** resembled those of **15** except for the replacement of a methylene group by an oxygenated methine at *δ*_C_ 75.3 and *δ*_H_ 3.55 (CH-6). The presence of an additional D_2_O exchangeable proton at *δ*_H_ 3.98 and its COSY correlation with H-6 indicated a hydroxy group at C-6. Therefore, **16** was elucidated as 6-hydroxylated derivative of cytosporin O (**15**). The relative configuration at C-3, C-5, C-7, and C-10 in **16** was determined to be identical to that of **15** by comparison of their coupling constants and NOE relationships. In addition, the NOE correlations from OH-6 to H-3 and H-7, and from H-6 to 5-OH and 7-OH confirmed α-orientation of OH-6 in **16**.

The HRESIMS as well as 1D and 2D NMR data of cytosporin Q (**17**) suggested that it possessed the same planar structure as the known cytosporin D bearing an epoxy ring at C-5/C-6 [18]. The NOE correlations observed in **17** between H-10/Me-12, Me-12/H-4a, H-4a/H-10, and between Me-11/H-3, H-3/H-4b, H-4b/H-6, indicated that **17** shared the same relative configuration at C-3, C-5, C-6, and C-10 as cytosporin D. However, the relative configuration at C-7 could not be determined by NOE relationships or *J* values. Therefore, the modified Mosher’s method was applied to determine the absolute configuration at C-3 and C-7 of **17**. In order to exclude the influence of the primary alcohol at C-13, 4,4′-dimethoxytrytil chloride (DMT-Cl) was used for protection before Mosher’s reaction, yielding compound **17a**. Meanwhile, Mosher’s reaction was also conducted with the coisolated known compound cytosporin F (**26**) [19], bearing an acetyl group attached to C-13. The differences in chemical shifts of (*R*)- and (*S*)-MPA esters assigned 3*R* and 7*R* absolute configuration for both **17a** and cytosporin F (**26**) (Figure 3). Thus, **17** was identified as 7-epimer of cytosporin D and has the absolute configuration of 3*R*, 5*S*, 6*R*, 7*R,* and 10*R*. Cytosporin D had been firstly reported from the fungus *Eutypella scoparia* derived from a marine pulmonate mollusk [18], while cytosporins F–K were produced by *Pestalotiopsis theae*, an endophyte from leaves of *Turraeanthus longipes* [19]. From biogenetic considerations and the results of Mosher’s reaction, it is reasonable to revise the absolute configurations of cytosporins F–K [19] as 3*R*, 5*S*, 6*R*, 7*R,* and 10*R*, the same as **17** rather than that of cytosporin D.

The molecular formula of cytosporin R (**18**) was determined as C_19_H_30_O_6_ by HRESIMS, with an additional oxygen atom compared to cytosporin Q (**17**). A methyl doublet at *δ*_H_ 1.03 (Me-20) was found in the ^1^H NMR data of **18** instead of a methyl triplet in **17** (Table 5). Detailed examination of the 2D NMR spectroscopic data revealed **18** to be the 19-OH substituted analog of cytosporin Q (**17**). Similar NOE interactions were observed in **18** as for **17**, indicating that both compounds shared the same relative configuration regarding the ring system. Due to the low amount, cytosporin R (**18**) was subjected for Mosher’s method directly without protecting the primary alcohol at C-13. The absolute configuration at C-19 of side chain was determined as *S* (Figure 3). From biogenetic considerations, the remaining stereocenters of **18** are assumed to share the same absolute configuration as cytosporin Q (**17**).

Compound **19** has the same molecular formula of C_19_H_30_O_6_ as cytosporin R (**18**) as determined by the HRESIMS data. After detailed analysis of the 1D and 2D NMR spectroscopic data of **19**, both compounds were found to differ in the position of the hydroxy group in the side chain. The COSY correlations between the D_2_O exchangeable proton at *δ*_H_ 4.34 and H-17 (*δ*_H_ 3.46), and between H-14/H-15/H-16ab/H-17 confirmed that C-17 was hydroxylated in **19**. Therefore, compound **19** was suggested to be the C-17 epimer of cytosporin K (**25**) [19]. Similar NOE correlations were observed in **19** and **25**, suggesting that both compounds share the same relative configuration regarding the bicyclic ring core. Due to the low amount of cytosporin S (**19**), Mosher’s reaction was carried out for its 17-epimer cytosporin K (**25**) [19], whose absolute configuration in the side chain was uncertain before. Comparison of the chemical shifts of (*R*)- and (*S*)-MPA esters of **25** (Figure 3) suggested 17*S* absolute configuration for cytosporin K (**25**). Thus, 17*R* absolute configuration was proposed for cytosporin S (**19**).

Cytosporins T and U (**20** and **21**) have the same molecular formula of C_19_H_30_O_6_ as deduced by the HRESIMS data. Their NMR data (Table 5 and Table 6) were comparable to those of **19**. However, the olefinic proton at H-15 appeared as dd in **20** and **21** rather than as dt in **19**, suggesting the hydroxy group in the side chain to be located at C-16 in **20** and **21** rather than at C-17 in **19**. This was further supported by the COSY correlations observed between H-14/H-15/H-16/H-17ab, as well as between H-17 and a D_2_O exchangeable proton in **20** and **21**. Detailed analysis of the 2D NMR spectroscopic data of **20** and **21** revealed that the remaining substructure was identical to that of **19**. Cytosporins T and U (**20** and **21**) only differ in the absolute configuration in the side chain, as found for **19** and **25**. The absolute configuration at C-16 of both compounds was not determined due to the low amounts.

The NMR data of compound **22** were similar to those of cytosporin F(**26**) [19], and both compounds have the same molecular formula as evident from the HRESIMS data. However, the shielded methylene group at C-13 (*δ*_C_ 57.6, *δ*_H_ 4.11 and 3.78 in **22** vs. *δ*_C_ 63.2, *δ*_H_ 4.74 and 4.67 in **26**), and the deshielded oxygenated methine at C-7 (*δ*_H_ 5.85 in **22** vs. *δ*_H_ 4.63 in **26**) in **22** suggested that the acetoxy moiety was attached at C-7 in **22** rather than at C-13 in **26**, which was further confirmed by the HMBC correlation from H-7 to the carbonyl carbon (*δ*_C_ 170.0) of the acetoxy moiety. The remaining substructure of **22** was elucidated to be identical to that of **26**. Based on the similar NOE correlations and the biogenetic relationship between **22** and **26**, compound **22** was suggested to share the same 3*R*, 5*S*, 6*R*, 7*R,* and 10*R* absolute configuration as **26**.

The molecular formula C_19_H_28_O_5_ was suggested for cytosporin W (**23**) by the HRESIMS data, indicating an additional degree of unsaturation when compared to **17**. The absence of one D_2_O changeable proton in the ^1^H NMR data (Table 6), along with the HMBC correlations from H-6 (*δ*_H_ 3.54) and H-14 (*δ*_H_ 6.04) to a carbonyl carbon at *δ*_C_ 195.8 indicated a ketone carbonyl to be located at C-7, which accounted for the additional degree of unsaturation. The remaining structure including the relative configuration was determined to be identical to that of **17** after detailed analysis of the 2D NMR spectroscopic data of **23**.

The remaining known compounds were identified as truncateols B (**10**), D (**11**), and H (**12**) [16], pestalotheols A (**13**) and D (**14**) [17], cytosporins J (**24**), K (**25**), and F (**26**) [19]. Both new compounds pestalotheol O (**7**) and Q (**9**) contain acetoxyl group at C-5. In order to exclude the possibility that they might be artifacts formed during the extraction with EtOAc, relative compounds pestalotheol N (**6**) and pestalotheol A (**13**) were incubated in EtOAc for 48 h at room temperature. Unchanged retention time and molecular weight from HPLC together with MS results indicated that they were natural products rather than artifacts.

In previous report, cytosporins A–C were known as angiotensin II binding inhibitors [20], while cytosporin L was active against the bacteria *Micrococcus lysodeikticus* and *Enterobacter aerogenes* with MIC values of 3.12 µM [21,22]. In the present study, all isolated compounds were tested for their cytotoxicity and antibacterial activity. Only compound **23**, with a carbonyl group at C-7 instead of a hydroxy group, showed significant cytotoxicity against the mouse lymphoma cell line L5178Y with an IC_50_ value of 3.0 μM, even stronger than that of the positive control kahalalide F (IC_50_ 4.3 μM), whereas the other compounds were not active (IC_50_ > 20 μM), suggesting the importance of α,β-unsaturated ketone moiety in **23** for cytotoxicity. In the antibacterial activity assay, none of the isolated compounds were active against the Gram-positive bacteria *Staphylococcus aureus* (ATCC 29213) and *Mycobacterium tuberculosis* (H37Rv) and the Gram-negative bacterium *Pseudomonas aeruginosa* (ATCC 27853) and drug-resistant *Acinetobacter baumannii* (ATCC BAA-1605) (MIC > 100 µM). However, in combination with a sublethal colistin concentration of 0.1 µM, compounds **22** and **23** displayed antibacterial activity against drug-resistant *Acinetobacter baumannii* (ATCC BAA-1605) with MIC values of 50 and 100 µM, respectively.

## 3. Materials and Methods

### 3.1. General Experimental Procedures

Optical rotations were measured with a Jasco P-2000 polarimeter (JASCO, Tokyo, Japan). Analytical HPLC was performed with a Dionex UltiMate-3400SD system coupled to an LPG-3400SD pump and a DAD300RS photodiode array detector (Dionex Softron, Germering, Germany). The analytical column (125 × 4 mm) was prefilled with Eurosphere-10 C18 (Knauer, Berlin, Germany), following the program: 0 min (10% MeOH); 5 min (10% MeOH); 35 min (100% MeOH); 45 min (100% MeOH). NMR spectra were recorded with Bruker ARX 300 or 600 NMR spectrometers (Bruker, Karlsruhe, Germany). Chemical shifts were referenced to the solvent residual peaks. HRESIMS were recorded on a UHR-QTOF maXis 4G mass spectrometer (Bruker Daltonics, Bremen, Germany). Column chromatography was conducted with Merck MN silica gel 60M (0.04–0.063 mm). TLC plates precoated with silica gel F_254_ (Merck) were used with detection under 254 and 366 nm. Distilled and spectral grade solvents were used for column chromatography and spectroscopic measurements, respectively. Semipreparative HPLC was performed using a Merck Hitachi HPLC System (UV detector L-5410; pump L-5100; Eurosphere-100 C18, 300 × 8 mm), with a mixture of MeOH-H_2_O or MeCN-H_2_O as mobile phase.

### 3.2. Fungal Material

The fungus was isolated from roots of the mangrove plant Rhizophora racemosa collected around Lagos in October 2017. It was identified as *Pseudopestalotiopsis theae* (GenBank accession number MN814071) by DNA amplification and sequencing of its ITS region as described before [23].

### 3.3. Fermentation, Extraction, and Isolation

The fungus was cultivated on solid rice medium in 10 Erlenmeyer flasks (1 L with 100 g rice and 110 mL demineralized water per flask, autoclaved at 121 °C for 20 min before inoculation). EtOAc (700 mL) was added to each flask after fermentation of 14 days at 20 °C. The fungal cultures were subjected to shaking at 140 rpm for 8 h following addition of EtOAc. After removal of the solvent under reduced pressure, the obtained residue (12.4 g) was subjected to liquid–liquid partition between *n*-hexane and MeOH. After HPLC analysis of both phases, only the methanolic phase was further chromatographically investigated.

The MeOH fraction (8.1 g) was subjected to a vacuum liquid silica gel column chromatography using a gradient solvent system of *n*-hexane and EtOAc (100:1 to 0:100; *v/v*) to obtain seven fractions (Fr. 1–7). Following Sephadex LH-20 column (60 × 3 cm) chromatography with MeOH as mobile phase, Fr. 3 (1.8 g) was separated into four subfractions (Fr. 3.1–3.4). Fr. 3.4 (0.6 g) was then subjected to vacuum liquid RP-18 column (60 × 200 mm) chromatography using a solvent gradient (from 100% H_2_O to 100% MeOH) to give five subfractions (Fr.3.4.1–3.4.5). Compounds **6** (4.0 mg), **7** (2.5 mg), **9** (2.2 mg), **10** (5.0 mg), **11** (15 mg),**12** (1.9 mg), **14** (1.7 mg), **22** (3.1 mg), and **23** (2.9 mg) were obtained by semipreparative HPLC (MeOH-H_2_O: 0–2 min, 40%; 2–27 min, from 40% to 60%; 27–32 min, 100%) from Fr.3.4.1 (110 mg). Fr. 4 (2.7g) was subjected to vacuum liquid RP-18 column chromatography (60 × 200 mm) using a solvent gradient (from 100% H_2_O to 100% MeCN) to give seven subfractions (Fr. 4.1–4.7). Compounds **1** (2.5 mg), **2** (1.6 mg), **4** (3.0 mg), **5** (1.4 mg), **18** (3.7 mg), and **24** (5.8 mg) were obtained by semipreparative HPLC (MeCN-H_2_O: 0–2 min, 8%; 2–22 min, from 8% to 20%; 22–30 min, 100%) from Fr.4.3 (210.0 mg), while compounds **15** (3.3 mg), **16** (3.0 mg), **17** (37.2 mg), and **26** (13.0 mg) were obtained by semipreparative HPLC (MeOH-H_2_O: 0–2 min, 30%; 2–22 min, from 30% to 60%; 22–30 min, 100%) from Fr.4.6 (200.0 mg). Following similar procedures, Fr. 5 (1.8 g) was also separated by vacuum liquid RP-18 column (60 × 200 mm) chromatography to give four subfractions (Fr.5.1–Fr.5.4). Compounds **13** (3.5 mg), **19** (1.5 mg), **20** (1.3 mg), **21**(3.8 mg), and **25** (6.2 mg) were obtained from Fr. 5.3 (130 mg) by semipreparative HPLC (MeOH-H_2_O: 0–2 min, 38%; 2–22 min, from 38% to 58%; 22–30 min, 100%), while compounds **3** (2.5 mg) and **8** (4.1 mg) were obtained by semipreparative HPLC (MeCN-H_2_O: 0–2 min, 5%; 2–22 min, from 5% to 15%; 22–30 min, 100%) from Fr. 5.4 (68.0 mg).

*Pestalotheol I* (1): colorless oil; ${\left[\alpha \right]}_{D}^{20}$ +115 (*c* 0.25, MeOH); UV (MeOH) λ_max_ 219 nm; ^1^H and ^13^C NMR data, Table 1; HRESIMS *m/z* 297.1694 [M + H]^+^ (C_16_H_25_O_5_, calcd. 297.1697), 319.1513 [M + Na]^+^ (C_16_H_24_O_5_Na, calcd. 319.1516).

*Pestalotheol J* (**2**): colorless oil; ${\left[\alpha \right]}_{D}^{20}$ −12 (*c* 0.20, MeOH); UV (MeOH) λ_max_ 268 nm; ^1^H and ^13^C NMR data, Table 1; HRESIMS *m/z* 297.1700 [M + H]^+^ (C_16_H_25_O_5_, calcd. 297.1702).

*Pestalotheol K* (**3**): colorless oil; ${\left[\alpha \right]}_{D}^{20}$ +111 (*c* 0.25, MeOH); UV (MeOH) λ_max_ 246 nm; ^1^H and ^13^C NMR data, Table 1; HRESIMS *m/z* 297.1700 [M + H]^+^ (C_16_H_25_O_5_, calcd. 297.1702).

*Pestalotheol L* (**4**): colorless oil; ${\left[\alpha \right]}_{D}^{20}$ −60 (*c* 0.30, MeOH); UV (MeOH) λ_max_ 260 nm; ^1^H and ^13^C NMR data, Table 2; HRESIMS *m/z* 317.1362 [M + Na]^+^ (C_16_H_22_O_5_Na, calcd. 317.1359).

*Pestalotheol M* (**5**): colorless oil; ${\left[\alpha \right]}_{D}^{20}$ +17 (*c* 0.25, MeOH); UV (MeOH) λ_max_ 262 nm; ^1^H and ^13^C NMR data, Table 2; HRESIMS *m/z* 317.1360 [M + Na]^+^ (C_16_H_22_O_5_Na, calcd. 317.1359).

*Pestalotheol N* (**6**): colorless oil; ${\left[\alpha \right]}_{D}^{20}$ +47 (*c* 0.15, MeOH); UV (MeOH) λ_max_ 219 nm; ^1^H and ^13^C NMR data, Table 2; HRESIMS *m/z* 319.1518 [M + Na]^+^ (C_16_H_24_O_5_Na, calcd.319.1516).

*Pestalotheol O* (**7**): colorless oil; ${\left[\alpha \right]}_{D}^{20}$ +35 (*c* 0.22, MeOH); UV (MeOH) λ_max_ 218 nm; ^1^H and ^13^C NMR data, Table 3; HRESIMS *m/z* 361.1619 [M + Na]^+^ (C_18_H_26_O_6_Na, calcd. 361.1622).

*Pestalotheol P* (**8**): colorless oil; ${\left[\alpha \right]}_{D}^{20}$ +89 (*c* 0.22, MeOH); UV (MeOH) λ_max_ 282 nm; ^1^H and ^13^C NMR data, Table 3; HRESIMS *m/z* 329.1595 [M + H]^+^ (C_16_H_25_O_7_, calcd. 329.1595).

*Pestalotheol Q* (**9**): colorless oil; ${\left[\alpha \right]}_{D}^{20}$ +121 (*c* 0.15, MeOH); UV (MeOH) λ_max_ 279 nm; ^1^H and ^13^C NMR data, Table 3; HRESIMS *m/z* 355.1754 [M + H]^+^ (C_18_H_27_O_7_, calcd. 355.1757).

*Cytosporin O* (**15**): colorless oil; ${\left[\alpha \right]}_{D}^{20}$ −8 (*c* 0.30, MeOH); UV (MeOH) λ_max_ 243 nm; ^1^H and ^13^C NMR data, Table 4; HRESIMS *m/z* 363.2144 [M + Na]^+^ (C_19_H_32_O_5_Na, calcd. 363.2142).

*Cytosporin P* (**16**): colorless oil; ${\left[\alpha \right]}_{D}^{20}$ −4 (*c* 0.35, MeOH); UV (MeOH) λ_max_ 240 nm; ^1^H and ^13^C NMR data, Table 4; HRESIMS *m/z* 379.2094 [M + Na]^+^ (C_19_H_32_O_6_Na, calcd. 379.2091).

*Cytosporin Q* (**17**): colorless oil; ${\left[\alpha \right]}_{D}^{20}$ +16 (*c* 0.22, MeOH); UV (MeOH) λ_max_ 237 nm; ^1^H and ^13^C NMR data, Table 4; HRESIMS *m/z* 356.2432 [M + NH_4_]^+^ (C_19_H_34_O_5_N, calcd. 356.2431).

*Cytosporin R* (**18**): colorless oil; ${\left[\alpha \right]}_{D}^{20}$ +6 (*c* 0.20, MeOH); UV (MeOH) λ_max_ 239 nm; ^1^H and ^13^C NMR data, Table 5; HRESIMS *m/z* 355.2117 [M + H]^+^ (C_19_H_31_O_6_, calcd. 355.2121).

*Cytosporin S* (**19**): colorless oil; ${\left[\alpha \right]}_{D}^{20}$ +14 (c 0.10, MeOH); UV (MeOH) λ_max_ 239 nm; ^1^H and ^13^C NMR data, Table 5; HRESIMS *m/z* 377.1934 [M + Na]^+^ (C_19_H_30_NaO_6_, calcd. 377.1935).

*Cytosporin T* (**20**): colorless oil; ${\left[\alpha \right]}_{D}^{20}$ −1 (*c* 0.20, MeOH); UV (MeOH) λ_max_ 238 nm; ^1^H and ^13^C NMR data, Table 5; HRESIMS *m/z* 377.1931[M + Na]^+^ (C_19_H_30_O_6_Na, calcd. 377.1935).

*Cytosporin U* (**21**): colorless oil; ${\left[\alpha \right]}_{D}^{20}$ + 4 (c 0.20, MeOH); UV (MeOH) λ_max_ 237 nm; ^1^H and ^13^C NMR data, Table 6; HRESIMS m/z 372.2381[M + NH_4_]^+^ (C_19_H_34_NO_6_, calcd. 372.2381), 377.1935 [M + Na]^+^ (C_19_H_30_O_6_Na, calcd. 377.1935).

*Cytosporin V* (**22**): colorless oil; ${\left[\alpha \right]}_{D}^{20}$ −21 (*c* 0.30, MeOH); UV (MeOH) λ_max_ 237 nm; ^1^H and ^13^C NMR data, Table 6; HRESIMS *m/z* 398.2537[M + NH_4_]^+^ (C_21_H_36_NO_6_, calcd. 398.2537), 403.2089 [M + Na]^+^ (C_21_H_32_O_6_Na, calcd. 403.2091).

*Cytosporin W* (**23**): colorless oil; ${\left[\alpha \right]}_{D}^{20}$ −4 (*c* 0.10, MeOH); UV (MeOH) λ_max_ 215, 281 nm; ^1^H and ^13^C NMR data, Table 6; HRESIMS *m/z* 359.1829 [M + Na]^+^ (C_19_H_28_O_5_Na, calcd. 359.1829).

### 3.4. Preparation of (R)- and (S)-MTPA Esters

Compound **1** (0.8 mg) was dissolved in prydine-*d*_6_ (500 μL) and then transferred to an NMR tube. (*S*)-MTPACl (10.0 µL, 0.050 mmol) was added quickly. By shaking the NMR tube, the reagent and the dissolved compound were mixed. After 10 h at room temperature, ^1^H NMR data were obtained for the (*R*)-MTPA ester (**1a**). Following the same protocol, the (*S*)-MTPA ester (**1b**) was produced. A similar procedure was applied for compound **8**.

### 3.5. Preparation of Compound 17a

Compound **17** (10 mg, 0.030 mmol) was dissolved in anhydrous CH_2_Cl_2_ (3 mL) together with catalytic amount of DMAP and 10.0 mg of 4,4′-dimethoxytrytil chloride (0.030 mmol, DMTCl) at room temperature for 2 h. After removal of the solvent, preparative thin-layer chromatography was used for purification, using CH_2_Cl_2_-MeOH (95:5), to give compound **17a**.

### 3.6. Preparation of (R)- and (S)-MPA Esters

Compound **7** (0.8 mg, 0.002 mmol), along with DMAP (0.5 mg, 0.004 mmol), *N*,*N*′-dicyclohexylcarbodiimide (0.8 mg, 0.004 mmol, DCC) and (*R*)-MPA (0.7 mg, 0.004 mmol), was mixed in anhydrous CH_2_Cl_2_ (0.3 mL). The solvent was removed after 2 h at room temperature. The (*R*)-MPA ester (**7a**) was obtained from the crude product by semipreparative HPLC (MeOH-H_2_O: 0-2 min, 40%; 2–15 min; 15–25 min, 100%). Following a similar procedure, the (*S*)-MPA ester (**7b**) was obtained by using (*S*)-MPA. The above steps were also applied to compounds **17a**, **18**, **25,** and **26**.

### 3.7. Cytotoxicity and Antibacterial Assay

The MTT method was applied for the cytotoxicity assay against the mouse lymphoma cell line L5178Y as described before [24]. Antibacterial activity against Gram-positive bacteria *Staphylococcus aureus* (ATCC 29213) and *Mycobacterium tuberculosis* (H37Rv), and the Gram-negative bacteria *Pseudomonas aeruginosa* (ATCC 27853) and drug-resistant *Acinetobacter baumannii* (ATCC BAA-1605), was evaluated using the microdilution method in alignment with the CLSI guidelines using Muller Hinton broth for bacteria and 7H9 broth for Mycobacteria [25]. Additionally, the antibacterial activity was performed in combination with sublethal concentrations of the antibiotic colistin (0.1 µM) for drug-resistant *A. baumannii* (ATCC BAA-1605) to elucidate synergistic effects of the combinatory therapy.

## Figures and Tables

**Table 1:** NMR Data of compounds **1**–**3**.

No.	1 *^a^*		2 *^a,c^*		3 *^b^*	
	δ_C_, Type	δ_H_ (*J* in Hz)	δ_C_, Type	δ_H_ (*J* in Hz)	δ_C_, Type	δ_H_ (*J* in Hz)
2	84.2, CH	3.95, dd (9.9, 6.4)	84.1, CH	3.57, dd (8.3, 5.7)	86.0, CH	4.07, dd (9.4, 6.7)
3	34.0, CH_2_	2.20, dd (12.4, 9.9) 1.60, dd (12.4, 6.4)	36.1, CH_2_	2.08, dd (13.5, 8.3) 1.81, dd (13.5, 5.7)	31.0, CH_2_	2.55, dd, (13.3, 9.4) 1.72, dd, (13.3, 6.7)
4	77.8, C		82.5, C		69.5, C	
5	68.9, CH	4.05, dd (5.3, 4.6)	72.3, CH	4.24, dd (5.0, 2.5)	60.1, CH	3.75, d (2.8)
6	138.0, CH	6.71, dd (4.6, 2.3)	141.0, CH	6.69, t (2.5)	64.6, CH	4.65, dd (8.8, 2.8)
7	138.7, C		137.8, C		135.5, C	
8	25.7, CH_2_	2.53, dd (16.0, 5.3) 2.09, ddd (16.0, 10.7, 2.3)	26.8, CH_2_	2.54, dd (16.9, 3.9) 2.14, ddd (16.9, 5.4, 2.5)	35.4, CH_2_	2.50, dd (12.1, 11.5) 2.21, dd, (12.1, 4.8)
9	76.3, CH	3.63, dd (10.7, 5.3)	82.7, CH	4.05, dd (5.4, 3.9)	77.7, CH	3.86, dd (11.5, 4.8)
10	191.9, C		190.1, C		127.6, CH	6.25, d (11.7)
11	120.7, CH	6.57, s	120.0, CH	6.59, s	122.8, CH	6.29, d (11.7)
12	153.7, C		153.6, C		139.9, C	
13	20.5, CH_3_	2.00, s	20.5, CH_3_	2.00, s	60.9, CH_2_	4.21, dd (12.5, 5.3) 4.16, dd (12.5, 5.3)
14	27.2, CH_3_	1.90, s	27.1, CH_3_	1.90, s	22.0, CH_3_	1.85, s
15	70.8, C		70.9, C		71.9, C	
16	25.8, CH_3_	1.07, s	26.0, CH_3_	1.09, s	26.6, CH_3_	1.23, s
17	25.9, CH_3_	1.01, s	26.7, CH_3_	0.99, s	25.8, CH_3_	1.09, s
4-OH		4.54, s		5.37, s		
5-OH		5.38, d (5.3)		5.52, d (5.0)		
6-OH						3.97, d (8.8)
13-OH						3.70, t (5.3)
15-OH		4.16, s		4.75, s		3.34, s

^a^ Recorded at 600 MHz (^1^H) and 150 MHz (^13^C) in DMSO-*d*_6_. ^b^ Recorded at 600 MHz (^1^H) and 150 MHz (^13^C) in Acetone-*d*_6_. ^c^ Data extracted from the HSQC and HMBC spectra.

**Table 2:** NMR Data of compounds **4**–**6**.

No.	4 *^a^*		5 *^a,b^*		6 *^a^*	
	δ_C_, Type	δ_H_ (*J* in Hz)	δ_C_, Type	δ_H_ (*J* in Hz)	δ_C_, Type	δ_H_ (*J* in Hz)
2	85.2, CH	3.73, dd (8.1, 7.7)	84.6, CH	4.01, dd (9.6, 6.4)	83.7, CH	3.86, dd (9.9, 6.3)
3	37.5, CH_2_	2.43, dd (13.0, 7.7) 1.79, dd (13.0, 8.1)	33.5, CH_2_	2.12, (dd, 12.0, 9.6) 1.59, dd, (12.0, 6.4)	34.1, CH_2_	2.12, dd (12.0, 9.9) 1.53, dd (12.0, 6.3)
4	79.1, C		76.4, C		79.8, C	
5	71.8, CH	3.60, t (4.0)	72.5, CH	3.81, d (3.7)	71.1, CH	3.83, d (3.8)
6	67.6, CH	4.11, dd (9.0, 4.0)	74.1, CH	3.73, d (9.1)	74.9, CH	4.01, s
7	124.1, C		122.2, C		102.2, C	
8	134.0, CH	5.87, d (3.2)	132.9, CH	6.10, d (1.7)	27.8, CH_2_	2.52, ddd (12.4, 11.9, 3.9) 2.23, dd (11.9, 4.4)
9	81.2, CH	4.10, d (3.2)	77.1, CH	4.35, d (1.7)	77.4, CH	3.81, dd (12.4, 4.4)
10	88.6, C		88.8, C		204.6, C	
11	90.2, C		88.7, C		95.7, CH	5.92, d (3.9)
12	126.4, C		126.0, C		139.0, C	
13	122.1, CH_2_	5.32, s 5.27, s	121.6, CH_2_	5.30, s 5.26, s	113.5, CH_2_	4.93, s 4.82, s
14	23.2, CH_3_	1.87, s	23.0, CH_3_	1.87, s	19.5, CH_3_	1.69, s
15	70.2, C		70.5, C		70.5, C	
16	25.9, CH_3_	1.07, s	25.8, CH_3_	1.10, s	26.1, CH_3_	1.07, s
17	26.4, CH_3_	1.00, s	25.5, CH_3_	0.99, s	25.9, CH_3_	0.98, s
4-OH		5.05, s		4.68, s		5.22, s
5-OH		4.77, d (4.0)		5.47, d (3.7)		5.28, d (3.8)
6-OH		5.08, d (9.0)		4.72, d (9.1)		5.74, br s
15-OH		4.31, s		4.23, s		4.17, s

^a^ Recorded at 600 MHz (^1^H) and 150 MHz (^13^C) in DMSO-*d*_6_. ^b^ Data extracted from the HSQC and HMBC spectra.

**Table 3:** NMR Data of compounds **7**–**9**.

No.	7 *^a^*		8 *^b^*		9 *^a,c^*	
	δ_C_, Type	δ_H_ (*J* in Hz)	δ_C_, Type	δ_H_ (*J* in Hz)	δ_C_, Type	δ_H_ (*J* in Hz)
2	83.5, CH	3.88, dd (9.6, 6.4)	86.1, CH	4.13, dd (10.1, 6.4)	84.4, CH	3.95, dd (9.5, 6.5)
3	34.0, CH	1.83, dd (12.3, 9.6) 1.62, dd (12.3, 6.4)	35.0, CH_2_	2.49, dd (12.5, 10.1) 1.77, dd (12.5, 6.4)	33.9, CH_2_	1.80, dd (12.5, 9.5) 1.66, dd (12.5, 6.5)
4	77.8, C		80.1, C		76.4, C	
5	72.0, CH	5.01, d (2.5)	72.2, CH	4.08, d (5.1)	71.3, CH	5.28, s
6	71.7, CH	4.04, d (2.5)	168.3, C		162.3, C	
7	101.5, C		107.0, C		110.0, C	
8	27.4, CH_2_	2.56, ddd (12.4, 12.0, 3.9,) 2.31, dd (12.0, 4.4)	22.4, CH_2_	2.70, dd (14.9, 5.7) 2.11 (dd, 14.9, 10.6)	21.3, CH_2_	2.55, dd (14.9, 5.5) 2.01, dd (14.9, 10.7)
9	78.0, CH	3.76, dd (12.4. 4.4)	76.9, CH	3.91, dd (10.6, 5.7)	76.5, CH	3.63, dd (10.7, 5.5)
10	204.4, CH		194.6, C		191.9, C	
11	96.5, CH	6.02, d (3.9)	76.1, CH	4.13, d (2.8)	46.4, CH_2_	2.65, d (16.7) 2.42, d (16.7)
12	138.2, C		84.4, C		80.4, C	
13	114.5, CH_2_	4.99, s 4.88, s	26.9, CH_3_	1.49, s	27.3, CH_3_	1.36, s
14	19.5, CH_3_	1.71, s	16.5, CH_3_	1.14, s	23.4, CH_3_	1.24, s
15	70.5, C		72.0, C		70.5, C	
16	25.9, CH_3_	1.09, s	26.7, CH_3_	1.18, s	26.1, CH_3_	0.99, s
17	26.0, CH_3_	0.98, s	25.6, CH_3_	1.09, s	25.6, CH_3_	1.08, s
4-OH		5.45, s		3.97, s		5.31, s
5-OH				5.09, d (5.1)		
6-OH		5.91, br s				
11-OH				4.30, d (2.8)		
13-OH		4.24, s				
15-OH				3.22, s		4.25, s
5-OAc	20.6, CH_3_ 169.2, C	2.00, s			20.5, CH_3_ 169.5, C	2.09, s

^a^ Recorded at 600 MHz (^1^H) and 150 MHz (^13^C) in DMSO-*d*_6_. ^b^ Recorded at 600 MHz (^1^H) and 150 MHz (^13^C) in Acetone-*d*_6_. ^c^ Data extracted from the HSQC and HMBC spectra.

**Table 4:** NMR Data of compounds **15**–**17**.

No.	15 *^a^*		16 *^a^*		17 *^b^*	
	δ_C_, Type	δ_H_ (*J* in Hz)	δ_C_, Type	δ_H_ (*J* in Hz)	δ_C_, Type	δ_H_ (*J* in Hz)
2	74.9, C		75.0, C		75.4, C	
3	70.6, CH	3.20, dt (12.0, 4.9)	69.8, CH	3.78, dt (11.4, 5.5)	72.1, CH	3.38, dt (11.7, 5.0)
4	42.7, CH_2_	1.75, dd (12.8, 4.9) 1.65, dd (12.8, 12.0)	42.3, CH_2_	2.00, dd (13.2, 5.5) 1.68, dd (13.2, 11.4)	35.8, CH_2_	2.04, dd (12.7, 11.7) 1.50, dd (12.7, 5.0)
5	69.3, C		69.2, C		58.1, C	
6	35.7, CH_2_	2.00, dd (13.9, 4.3) 1.54, d (13.9)	75.3, CH	3.55, d (8.0)	61.8, CH	3.24, d (2.7)
7	63.9, CH	4.30, dd (7.6, 4.3)	69.5, CH	4.11, d (7.6)	64.8, CH	4.47, dd (7.3, 2.7)
8	133.8, C		131.8, C		132.9, C	
9	132.0, C		131.7, C		130.0, C	
10	70.0, CH	3.87, s	70.1, CH	4.01, s	65.4, CH	4.34, s
11	27.9, CH_3_	1.07, s	27.7, CH_3_	1.06, s	27.7, CH_3_	1.12, s
12	16.1, CH_3_	1.10, s	16.4, CH_3_	1.13, s	16.1, CH_3_	1.16, s
13	57.7, CH_2_	4.21, dd (11.9, 4.0) 3.81, dd (11.9, 6.2)	57.5, CH_2_	4.24, dd (11.9, 4.2) 3.92, dd (11.9, 6.2)	58.0, CH_2_	4.09, br d (11.6) 3.79, br d (11.6)
14	126.0, CH	6.36, d (15.7)	126.4, CH	6.44, d (15.8)	124.9, CH	6.06, d (15.8)
15	132.1, CH	5.90, dt (15.7, 7.0)	132.1, CH	5.91, dt (15.8, 7.0)	134.5, CH	5.85, dt (15.8, 6.8)
16	32.8, CH_2_	2.10, m	32.8, CH_2_	2.12, m	33.0, CH_2_	2.06, m
17	28.6, CH_2_	1.37, m	28.6, CH_2_	1.38, m	28.5, CH_2_	1.36, m
18	30.8, CH_2_	1.27, m	30.8, CH_2_	1.27, m	30.8, CH_2_	1.27, m
19	22.0, CH_2_	1.29, m	22.0, CH_2_	1.29, m	22.0, CH_2_	1.28, m
20	13.9, CH_3_	0.86, t (6.9)	13.9, CH_3_	0.87, t (6.9)	13.9, CH_3_	0.86, t (7.0)
3-OH		4.76, d (4.9)		4.70, d (5.5)		5.05, d (5.0)
5-OH		5.07, s		5.16, s		
6-OH				3.98, d (8.0)		
7-OH		4.75, d (7.6)		4.88, d (7.6)		4.92, d (7.3)
13-OH		4.53, dd (6.2, 4.0)		4.54, dd (6.2, 4.2)		4.55, br s

^a^ Recorded at 600 MHz (^1^H) and 150 MHz (^13^C) in DMSO-*d*_6_. ^b^ Recorded at 300 MHz (^1^H) and 75 MHz (^13^C) in DMSO-*d*_6_.

**Table 5:** NMR Data of compounds **18**–**20**.

No.	18 *^a^*		19 *^b,c^*		20 *^b,c^*	
	δ_C_, Type	δ_H_ (*J* in Hz)	δ_C_, Type	δ_H_ (*J* in Hz)	δ_C_, Type	δ_H_ (*J* in Hz)
2	75.4, C		75.2, C		75.2, C	
3	72.1, CH	3.39, m	71.8, CH	3.38, dt (11.8, 5.0)	71.8, CH	3.38, dt (11.8, 4.9)
4	35.8, CH_2_	2.04, m 1.50, dd (12.7, 5.0)	35.5, CH_2_	2.05, dd (12.6, 11.8) 1.50, dd (12.6, 5.0)	35.5, CH_2_	2.05, dd (12.7, 11.8) 1.50, dd (12.7, 4.9)
5	58.1, C		57.7, C		57.7, C	
6	61.8, CH	3.24, d (2.5)	61.5, CH	3.25, d (2.6)	61.5, CH	3.25, d (2.5)
7	64,8, CH	4.48, dd (7.6, 2.5)	64.5, CH	4.48, dd (7.4, 2.6)	64.4, CH	4.49, d (7.5, 2.5)
8	132.9, C		132.5, C		132.3, C	
9	130.0, C		129.8, C		130.4, C	
10	65.4, CH	4.34, s	65.1, CH	4.34, s	65.0, CH	4.35, s
11	27.7, CH_3_	1.12, s	27.5, CH_3_	1.12, s	27.4, CH_3_	1.12, s
12	16.1, CH_3_	1.16, s	15.8, CH_3_	1.16, s	15.8, CH_3_	1.16, s
13	58.0, CH_2_	4.09, dd (12.0, 2.0) 3.79, dd (12.0, 3.5)	57.7, CH_2_	4.11, dd (11.9, 2.3) 3.79, dd (11.9, 4.2)	57.6, CH_2_	4.12, dd (11.9, 2.0) 3.79, dd (11.9, 4.0)
14	124.9, CH	6.06, d (15.9)	126.2, CH	6.08, d (16.0)	122.8, CH	6.20, d (16.0)
15	134.6, CH	5.85, dt (15.9, 7.0)	131.4, CH	5.88, dt (16.0, 7.2)	137.8, CH	5.86, dd (16.0, 6.2)
16	33.1, CH_2_	2.05, m	41.5, CH_2_	2.15, m	71.0, CH_2_	3.95, m
17	25.2, CH_2_	1.40, m 1.34, m	69.2, CH	3.46, m	36.9, CH_2_	1.40, m
18	38.5, CH_2_	1.33, m 1.30, m	38.4, CH_2_	1.36, m 1.27, m	27.0, CH_2_	1.28, m
19	65.6, CH	3.57, m	18.1, CH_2_	1.38, m 1.27, m	21.9, CH_2_	1.27, m
20	23.7, CH_3_	1.03, d (6.1)	13.8, CH_3_	0.85, t (6.9)	13.7, CH_3_	0.86, t (6.9)
3-OH		5.06, d (4.5)		5.07, d (5.0)		5.07, d (4.9)
7-OH		4.94, d (7.6)		4.92, d (7.4)		4.94, d (7.5)
13-OH		4.56, dd (3.5, 2.0)		4.56, dd (4.2, 2.3)		4.58, dd (4.0, 2.0)
16-OH						4.62, d (4.0)
17-OH				4.34, br s		
19-OH		4.31, d (4.3)				

^a^ Recorded at 300 MHz (^1^H) and 75 MHz (^13^C) in DMSO-*d*_6_. ^b^ Recorded at 600 MHz (^1^H) and 150 MHz (^13^C) in DMSO-*d*_6_. ^c^ Data extracted from the HSQC and HMBC spectra.

**Table 6:** NMR Data of compounds **21**–**23**.

No.	21 *^a^*		22 *^b^*		23 *^b,c^*	
	δ_C_, Type	δ_H_ (*J* in Hz)	δ_C_, Type	δ_H_ (*J* in Hz)	δ_C_, Type	δ_H_ (*J* in Hz)
2	75.4, C		75.7, C		76.7, C	
3	72.0, CH	3.39, dt (11.8, 4.9)	71.8, CH	3.42, dt (11.8, 4.9)	71.9, CH	3.45, dt (11.6, 5.0)
4	35.8, CH_2_	2.05, dd (12.6, 11.8) 1.50, dd (12.6, 4.9)	35.2, CH_2_	2.06, dd (12.7, 11.8) 1.53, dd (12.7, 4.9)	34.0, CH_2_	2.21, dd (12.8, 11.6) 1.63, dd (12.8, 5.0)
5	57.9, C		58.0, C		61.1, C	
6	61.7, CH	3.25, d (2.3)	58.1, CH	3.39, d (2.8)	59.3, CH	3.54, d (1.2)
7	64.8, CH	4.48, dd (7.3, 2.3)	67.6, CH	5.85, d (2.8)	195.8, C	
8	132.5, C		128.0, C		130.3, C	
9	130.7, C		133.4, C		148.2, C	
10	65.3, CH	4.35, s	64.7, CH	4.40, s	64.5, CH	4.74, d (1.2)
11	27.7, CH_3_	1.12, s	27.6, CH_3_	1.13, s	27.5, CH_3_	1.16, s
12	16.1, CH_3_	1.16, s	16.1, CH_3_	1.17, s	16.1, CH_3_	1.24, s
13	58.0, CH_2_	4.12, dd (11.8, 4.1) 3.79, dd (11.8, 5.0)	57.6, CH_2_	4.11, dd (12.0, 4.4) 3.78, dd (12.0, 5.8)	58.1, CH_2_	4.26, dd (13.5, 2.1) 4.02, dd (13.5, 4.5)
14	123.3, CH	6.18, d (16.0)	123.8, CH	6.04, d (16.1)	120.6, CH	6.04, m
15	138.2, CH	5.86, dd (16.0, 6.1)	133.9, CH	5.49, dt (16.1, 7.0)	138.4, CH	6.03, m
16	71.3, CH	3.94, m	32.6, CH_2_	2.04, m	33.0, CH_2_	2.10, m
17	37.1, CH_2_	1.40, m 1.34, m	28.4, CH_2_	1.32, m	28.2, CH_2_	1.37, m
18	27.2, CH_2_	1.27, m	30.6, CH_2_	1.23, m	30.7, CH_2_	1.26, m
19	22.2, CH_2_	1.25, m	21.9, CH_2_	1.27, m	21.9, CH_2_	1.28, m
20	14.0, CH_3_	0.86, t (6.9)	13.9, CH_3_	0.86, t (6.8)	13.9, CH_3_	0.86, t (7.0)
3-OH		5.07, d (4.9)		5.12, d (4.9)		5.26, d (5.0)
7-OH		4.91, d (7.3)				
13-OH		4.57, dd (5.0, 4.1)		4.73, dd (5.8, 4.4)		5.17, dd (4.5, 2.1)
16-OH		4.64, d (4.5)				
7-OAc			20.6, CH_3_ 170.0, C	2.02, s		

^a^ Recorded at 300 MHz (^1^H) and 75 MHz (^13^C) in DMSO-*d*_6_. ^b^ Recorded at 600 MHz (^1^H) and 150 MHz (^13^C) in DMSO-*d*_6_. ^c^ Data extracted from the HSQC and HMBC spectra.

## Supplementary Materials

The following are available online at https://www.mdpi.com/1660-3397/18/2/129/s1, UV, HRMS, 1D and 2D NMR spectra of all the new compounds **1**–**9** and **15**–**23** as well as spectra for Mosher’s esters of **1**, **7**, **8**, **17**, **18**, **25** and **26**.
