# Supplementary material for: Polyketide Derivatives from Mangrove Derived Endophytic Fungus Pseudopestalotiopsis theae
Source: Mar Drugs. 2020 Feb 23;18(2):129. doi: 10.3390/md18020129 (PMC7073511; doi:10.3390/md18020129)
Supplement: Supplementary file 1 [file marinedrugs-18-00129-s001.pdf]

**Support Information**

# Polyketide Derivatives from Mangrove Derived Endophytic Fungus *Pseudopestalotiopsis theae*

**Xiaoqin Yu [1,2], Werner E. G. Müller [3], Dieter Meier [1], Rainer Kalscheuer [1], Zhiyong Guo [2], Kun Zou [2], Blessing O. Umeokoli [4], Zhen Liu [1,*] and Peter Proksch [1,2,*]**

[1] Institute of Pharmaceutical Biology and Biotechnology, Heinrich-Heine-University Duesseldorf, 40225 Duesseldorf, Germany; xiyu101@hhu.de (X.Y.); dieter.meier@hhu.de (D.M.); Rainer.Kalscheuer@hhu.de (R.K.)

[2] Hubei Key Laboratory of Natural Products Research and Development, College of Biological and Pharmaceutical Sciences, China Three Gorges University, Yichang 443002, People's Republic of China; zhyguoctgu@foxmail.com (Z.G.); kzou@ctgu.edu.cn (K.Z.)

[3] Institute of Physiological Chemistry, Universitätsmedizin der Johannes Gutenberg-Universität Mainz, 55128 Mainz, Germany; wmueller@uni-mainz.de (W.E.G.M.)

[4] Department of Pharmaceutical and Medicinal Chemistry, Nnamdi Azikiwe University, Awka, Nigeria; blessingumeokoli@gmail.com (B.O.U.)

* Correspondence: zhenfeizi0@sina.com (Z.L.); proksch@uni-duesseldorf (P.P.); Tel.: +49-211-81-14163

## Table of Content

**Figure S1.** The HRESIMS of compound **1**. 8

**Figure S2.** The UV spectrum of compound **1**. 8

**Figure S3.** The $^{1}$H-NMR (600 MHz, DMSO-$d_6$) spectrum of compound **1**. 9

**Figure S4.** The $^{13}$C-NMR (150 MHz, DMSO-$d_6$) spectrum of compound **1**. 9

**Figure S5.** The $^{1}$H-$^{1}$H COSY (600 MHz, DMSO-$d_6$) spectrum of compound **1**. 10

**Figure S6.** The HSQC (600 MHz, DMSO-$d_6$) spectrum of compound **1**. 10

**Figure S7.** The HMBC (600 MHz, DMSO-$d_6$) spectrum of compound **1**. 11

**Figure S8.** The ROESY (600 MHz, DMSO-$d_6$) spectrum of compound **1**. 11

**Figure S9.** The HRESIMS of compound **2**. 12

**Figure S10.** The UV spectrum of compound **2**. 12

**Figure S11.** The $^{1}$H-NMR (600 MHz, DMSO-$d_6$) spectrum of compound **2**. 13

**Figure S12.** The $^{1}$H-$^{1}$H COSY (600 MHz, DMSO-$d_6$) spectrum of compound **2**. 13

**Figure S13.** The HSQC (600 MHz, DMSO-$d_6$) spectrum of compound **2**. 14

**Figure S14.** The HMBC (600 MHz, DMSO-$d_6$) spectrum of compound **2**. 14

**Figure S15.** The ROESY (600 MHz, DMSO-$d_6$) spectrum of compound **2**. 15

**Figure S16.** The HRESIMS of compound **3**. 15

**Figure S17.** The UV spectrum of compound **3**. 16

**Figure S18.** The $^{1}$H-NMR (600 MHz, Acetone-$d_6$) spectrum of compound **3**. 16

**Figure S19.** The $^{13}$C-NMR (150 MHz, Acetone-$d_6$) spectrum of compound **3**. 17

**Figure S20.** The $^{1}$H-$^{1}$H COSY (600 MHz, Acetone-$d_6$) spectrum of compound **3**. 17

**Figure S21.** The HSQC (600 MHz, Acetone-$d_6$) spectrum of compound **3**. 18

**Figure S22.** The HMBC (600 MHz, Acetone-$d_6$) spectrum of compound **3**. 18

**Figure S23.** The ROESY (600 MHz, Acetone-$d_6$) spectrum of compound **3**. 19

**Figure S24.** The HRESIMS of compound **4**. 19

**Figure S25.** The UV spectrum of compound **4**. 20

**Figure S26.** The $^{1}$H-NMR (600 MHz, DMSO-$d_6$) spectrum of compound **4**. 20

**Figure S27.** The $^{13}$C-NMR (150 MHz, DMSO-$d_6$) spectrum of compound **4**. 21

**Figure S28.** The $^{1}$H-$^{1}$H COSY (600 MHz, DMSO-$d_6$) spectrum of compound **4**. 21

**Figure S29.** The HSQC (600 MHz, DMSO-$d_6$) spectrum of compound **4**. 22

**Figure S30.** The HMBC (600MHz, DMSO-$d_6$) spectrum of compound **4**. 22

**Figure S31.** The ROESY (600 MHz, DMSO-$d_6$) spectrum of compound **4.** 23
**Figure S32.** The HRESIMS of compound **5**. 23
**Figure S33.** The UV spectrum of compound **5.** 24
**Figure S34.** The $^1$H-NMR (600 MHz, DMSO-$d_6$) spectrum of compound **5.** 24
**Figure S35.** The $^1$H-$^1$H COSY (600 MHz, DMSO-$d_6$) spectrum of compound **5**. 25
**Figure S36.** The HSQC (600 MHz, DMSO-$d_6$) spectrum of compound **5.** 25
**Figure S37.** The HMBC (600MHz, DMSO-$d_6$) spectrum of compound **5**. 26
**Figure S38.** The ROESY (600 MHz, DMSO-$d_6$) spectrum of compound **5.** 26
**Figure S39.** The HRESIMS of compound **6**. 27
**Figure S40.** The UV spectrum of compound **6.** 27
**Figure S41.** The $^1$H-NMR (600 MHz, DMSO-$d_6$) spectrum of compound **6.** 28
**Figure S42.** The $^{13}$C-NMR (150 MHz, DMSO-$d_6$) spectrum of compound **6.** 28
**Figure S43.** The $^1$H-$^1$H COSY (600 MHz, DMSO-$d_6$) spectrum of compound **6**. 29
**Figure S44.** The HSQC (600 MHz, DMSO-$d_6$) spectrum of compound **6.** 29
**Figure S45.** The HMBC (600 MHz, DMSO-$d_6$) spectrum of compound **6**. 30
**Figure S46.** The ROESY (600 MHz, DMSO-$d_6$) spectrum of compound **6.** 30
**Figure S47.** The HRESIMS of compound **7**. 31
**Figure S48.** The UV spectrum of compound **7.** 31
**Figure S49.** The $^1$H-NMR (600 MHz, DMSO-$d_6$) spectrum of compound **7.** 32
**Figure S50.** The $^{13}$C-NMR (150 MHz, DMSO-$d_6$) spectrum of compound **7.** 32
**Figure S51.** The $^1$H-$^1$H COSY (600 MHz, DMSO-$d_6$) spectrum of compound **7**. 33
**Figure S52.** The HSQC (600 MHz, DMSO-$d_6$) spectrum of compound **7.** 33
**Figure S53.** The HMBC (600 MHz, DMSO-$d_6$) spectrum of compound **7**. 34
**Figure S54.** The ROESY (600 MHz, DMSO-$d_6$) spectrum of compound **7.** 34
**Figure S55.** The HREISMS of compound **8**. 35
**Figure S56.** The UV spectrum of compound **8.** 35
**Figure S57.** The $^1$H-NMR (600 MHz, Acetone-$d_6$) spectrum of compound **8.** 36
**Figure S58.** The $^{13}$C-NMR (150 MHz, Acetone-$d_6$) spectrum of compound **8.** 36
**Figure S59.** The $^1$H-H COSY (600 MHz, Acetone-$d_6$) spectrum of compound **8.** 37
**Figure S60.** The HSQC (600 MHz, Acetone-$d_6$) spectrum of compound **8**. 37
**Figure S61.** The HMBC (600 MHz, Acetone-$d_6$) spectrum of compound **8**. 38
**Figure S62.** The ROESY (600 MHz, Acetone-$d_6$) spectrum of compound **8.** 38

**Figure S63.** The HREISMS of compound **9.** ........ 39
**Figure S64.** The UV spectrum of compound **9.** ........ 39
**Figure S65.** The $^{1}$H-NMR (600 MHz, DMSO-$d_6$) spectrum of compound **9.** ........ 40
**Figure S66.** The $^{1}$H-$^{1}$H COSY (600 MHz, DMSO-$d_6$) spectrum of compound **9.** ........ 40
**Figure S67.** The HSQC (600 MHz, DMSO-$d_6$) spectrum of compound **9.** ........ 41
**Figure S68.** The HMBC (600 MHz, DMSO-$d_6$) spectrum of compound **9.** ........ 41
**Figure S69.** The ROESY (600 MHz, DMSO-$d_6$) spectrum of compound **9.** ........ 42
**Figure S70.** The HREISMS of compound **15.** ........ 42
**Figure S71.** The UV spectrum of compound **15.** ........ 43
**Figure S72.** The $^{1}$H-NMR (600 MHz, DMSO-$d_6$) spectrum of compound **15.** ........ 43
**Figure S73.** The $^{13}$C-NMR (150 MHz, DMSO-$d_6$) spectrum of compound **15.** ........ 44
**Figure S74.** The $^{1}$H-$^{1}$H COSY (600 MHz, DMSO-$d_6$) spectrum of compound **15.** ........ 44
**Figure S75.** The HSQC (600 MHz, DMSO-$d_6$) spectrum of compound **15.** ........ 45
**Figure S76.** The HMBC (600 MHz, DMSO-$d_6$) spectrum of compound **15.** ........ 45
**Figure S77.** The ROESY (600 MHz, DMSO-$d_6$) spectrum of compound **15.** ........ 46
**Figure S78.** The HREISMS of compound **16.** ........ 46
**Figure S79.** The UV spectrum of compound **16.** ........ 47
**Figure S80.** The $^{1}$H-NMR (600 MHz, DMSO-$d_6$) spectrum of compound **16.** ........ 47
**Figure S81.** The $^{13}$C-NMR (150 MHz, DMSO-$d_6$) spectrum of compound **16.** ........ 48
**Figure S82.** The $^{1}$H-$^{1}$H COSY (600 MHz, DMSO-$d_6$) spectrum of compound **16.** ........ 48
**Figure S83.** The HSQC (600 MHz, DMSO-$d_6$) spectrum of compound **16.** ........ 49
**Figure S84.** The HMBC (600 MHz, DMSO-$d_6$) spectrum of compound **16.** ........ 49
**Figure S85.** The ROESY (600 MHz, DMSO-$d_6$) spectrum of compound **16.** ........ 50
**Figure S86.** The HREISMS of compound **17.** ........ 50
**Figure S87.** The UV spectrum of compound **17.** ........ 51
**Figure S88.** The $^{1}$H-NMR (300 MHz, DMSO-$d_6$) spectrum of compound **17.** ........ 51
**Figure S89**. The $^{13}$C-NMR (75 MHz, DMSO-$d_6$) spectrum of compound **17.** ........ 52
**Figure S90**. The $^{1}$H-$^{1}$H COSY (300 MHz, DMSO-$d_6$) spectrum of compound **17.** ........ 52
**Figure S91.** The HSQC (300 MHz, DMSO-$d_6$) spectrum of compound **17.** ........ 53
**Figure S92.** The HMBC (300 MHz, DMSO-$d_6$) spectrum of compound **17.** ........ 53
**Figure S93.** The ROESY (300 MHz, DMSO-$d_6$) spectrum of compound **17.** ........ 54
**Figure S94.** The HREISMS of compound **18.** ........ 54

**Figure S95.** The UV spectrum of compound **18.** 55

**Figure S96.** The $^1$H-NMR (300 MHz, DMSO-$d_6$) spectrum of compound **18.** 55

**Figure S97**. The $^{13}$C-NMR (75 MHz, DMSO-$d_6$) spectrum of compound **18.** 56

**Figure S98.** The $^1$H-$^1$H COSY (300 MHz, DMSO-$d_6$) spectrum of compound **18.** 56

**Figure S99.** The HSQC (300 MHz, DMSO-$d_6$) spectrum of compound **18.** 57

**Figure S100.** The HMBC (300 MHz, DMSO-$d_6$) spectrum of compound **18.** 57

**Figure S101.** The ROESY (300 MHz, DMSO-$d_6$) spectrum of compound **18.** 58

**Figure S102.** The HREISMS of compound **19.** 58

**Figure S103.** The UV spectrum of compound **19.** 59

**Figure S104.** The $^1$H-NMR (600 MHz, DMSO-$d_6$) spectrum of compound **19.** 59

**Figure S105.** The $^1$H-$^1$H COSY (600 MHz, DMSO-$d_6$) spectrum of compound **19.** 60

**Figure S106.** The HSQC (600MHz, DMSO-$d_6$) spectrum of compound **19.** 60

**Figure S107.** The HMBC (600MHz, DMSO-$d_6$) spectrum of compound **19.** 61

**Figure S108.** The ROESY (600 MHz, DMSO-$d_6$) spectrum of compound **19.** 61

**Figure S109.** The HREISMS of compound **20.** 62

**Figure S110.**The UV spectrum of compound **20.** 62

**Figure S111.**The $^1$H-NMR (600 MHz, DMSO-$d_6$) spectrum of compound **20.** 63

**Figure S112.** The $^1$H-$^1$H COSY (600 MHz, DMSO-$d_6$) spectrum of compound **20.** 63

**Figure S113.** The HSQC (600 MHz, DMSO-$d_6$) spectrum of compound **20.** 64

**Figure S114.** The HMBC (600 MHz, DMSO-$d_6$) spectrum of compound **20.** 64

**Figure S115.** The ROESY ((600 MHz, DMSO-$d_6$) spectrum of compound **20.** 65

**Figure S116.** The HREISMS of compound **21.** 65

**Figure S117.** The UV spectrum of compound **21.** 66

**Figure S118**. The $^1$H-NMR (300 MHz, DMSO-$d_6$) spectrum of compound **21.** 66

**Figure S119.** The $^{13}$C-NMR (75 MHz, DMSO-$d_6$) spectrum of compound **21.** 67

**Figure S120.** The $^1$H-$^1$H COSY (300 MHz, DMSO-$d_6$) spectrum of compound **21.** 67

**Figure S121.** The HSQC (300 MHz, DMSO-$d_6$) spectrum of compound **21.** 68

**Figure S122.** The HMBC (300 MHz, DMSO-$d_6$) spectrum of compound **21.** 68

**Figure S123.** The ROESY (300 MHz, DMSO-$d_6$) spectrum of compound **21.** 69

**Figure S124.** The HREISMS of compound **22.** 69

**Figure S125.** The UV spectrum of compound **22.** 70

**Figure S126.** The $^1$H-NMR (600 MHz, DMSO-$d_6$) spectrum of compound **22.** 70

**Figure S127**. The $^{13}$C-NMR (150 MHz, DMSO-$d_6$) spectrum of compound **22.** ........ 71
**Figure S128.** The $^{1}$H-$^{1}$H COSY (600 MHz, DMSO-$d_6$) spectrum of compound **22.** ........ 71
**Figure S129.** The HSQC (600 MHz, DMSO-$d_6$) spectrum of compound **22.** ........ 72
**Figure S130.** The HMBC (600 MHz, DMSO-$d_6$) spectrum of compound **22.** ........ 72
**Figure S131.** The ROESY (600 MHz, DMSO-$d_6$) spectrum of compound **22.** ........ 73
**Figure S132.** The HREISMS of compound **23.** ........ 73
**Figure S133.** The UV spectrum of compound **23.** ........ 74
**Figure S134.** The $^{1}$H-NMR (600 MHz, DMSO-$d_6$) spectrum of compound **23.** ........ 74
**Figure S135.** The $^{1}$H-$^{1}$H COSY (600 MHz, DMSO-$d_6$) spectrum of compound **23.** ........ 75
**Figure S136.** The HSQC (600 MHz, DMSO-$d_6$) spectrum of compound **23.** ........ 75
**Figure S137.** The HMBC (600 MHz, DMSO-$d_6$) spectrum of compound **23.** ........ 76
**Figure S138.** The ROESY (600 MHz, DMSO-$d_6$) spectrum of compound **23.** ........ 76
**Figure S139.** The $^{1}$H-NMR (600 MHz, Prydine-$d_5$) spectrum of **1a.** ........ 77
**Figure S140.** The $^{1}$H-$^{1}$H COSY (600 MHz, Prydine-$d_5$) spectrum of **1a.** ........ 77
**Figure S141.** The ESIMS of **1a.** ........ 78
**Figure S142.** The $^{1}$H-NMR (600 MHz, Prydine-$d_5$) spectrum of **1b.** ........ 78
**Figure S143.** The $^{1}$H-$^{1}$H COSY (600 MHz, Prydine-$d_5$) spectrum of **1b.** ........ 79
**Figure S144.** The ESIMS of **1b.** ........ 79
**Figure S145.** The $^{1}$H-NMR (600 MHz, $CD_3OD$) spectrum of **7a.** ........ 80
**Figure S146.** The $^{1}$H-$^{1}$H COSY (600 MHz, $CD_3OD$) spectrum of **7a.** ........ 80
**Figure S147.** The ESIMS of **7a.** ........ 81
**Figure S148.** The $^{1}$H-NMR (600 MHz, $CD_3OD$) spectrum of **7b.** ........ 81
**Figure S149.** The $^{1}$H-$^{1}$H COSY (600 MHz, $CD_3OD$) spectrum of **7b.** ........ 82
**Figure S150.** The ESIMS of **7b.** ........ 82
**Figure S151.** The $^{1}$H-NMR (600 MHz, $CD_3OD$) spectrum of **8a.** ........ 83
**Figure S152.** The $^{1}$H-$^{1}$H COSY (600 MHz, $CD_3OD$) spectrum of **8a.** ........ 83
**Figure S153.** The ROESY (600 MHz, $CD_3OD$) spectrum of **8a.** ........ 84
**Figure S154.** The ESIMS of **8a.** ........ 84
**Figure S155.** The $^{1}$H-NMR (600 MHz, $CD_3OD$) spectrum of **8b.** ........ 85
**Figure S156.** The $^{1}$H-$^{1}$H COSY (600 MHz, $CD_3OD$) spectrum of **8b.** ........ 85
**Figure S157.** The ROESY (600 MHz, $CD_3OD$) spectrum of **8b.** ........ 86
**Figure S158.** The ESIMS of **8b.** ........ 86

**Figure S159.** The $^{1}$H-NMR (600 MHz, $CD_3OD$) spectrum of 1**7a**. 87

**Figure S160.** The ESIMS of 1**7a**. 87

**Figure S161.** The $^{1}$H-NMR (600 MHz, $CD_3OD$) spectrum of 1**7b**. 88

**Figure S162.** The $^{1}$H-$^{1}$H COSY (600 MHz, $CD_3OD$) spectrum of 1**7b**. 88

**Figure S163.** The ESIMS of 1**7b**. 89

**Figure S164.** The $^{1}$H-NMR (600 MHz, $CD_3OD$) spectrum of **17c**. 89

**Figure S165.** The $^{1}$H-$^{1}$H COSY (600 MHz, $CD_3OD$) spectrum of 1**7c.** 90

**Figure S166.** The ESIMS of 1**7c**. 90

**Figure S167.** The $^{1}$H-NMR (600 MHz, $CD_3OD$) spectrum of **18a**. 91

**Figure S168.** The $^{1}$H-$^{1}$H COSY (600 MHz, CD3OD) spectrum of 1**8a.** 91

**Figure S169.** The ESIMS of 1**8a**. 92

**Figure S170.** The $^{1}$H-NMR (600 MHz, $CD_3OD$) spectrum of **18b**. 92

**Figure S171.** The $^{1}$H-$^{1}$H COSY (600 MHz, $CD_3OD$) spectrum of 1**8b**. 93

**Figure S172.** The ESIMS of 1**8b**. 93

**Figure S173.** The $^{1}$H-NMR (600 MHz, $CD_3OD$) spectrum of **25a**. 94

**Figure S174.** The $^{1}$H-$^{1}$H COSY (600 MHz, $CD_3OD$) spectrum of **25a**. 94

**Figure S175.** The ESIMS of **25a**. 95

**Figure S176.** The $^{1}$H-NMR (600 MHz, $CD_3OD$) spectrum of **25b**. 96

**Figure S177.** The $^{1}$H-$^{1}$H COSY (600 MHz, $CD_3OD$) spectrum of **25b**. 96

**Figure S178.** The ESIMS of **25b**. 97

**Figure S179.** The $^{1}$H-NMR (600 MHz, $CD_3OD$) spectrum of **26a**. 98

**Figure S180.** The $^{1}$H-$^{1}$H COSY (600 MHz, $CD_3OD$) spectrum of **26a**. 98

**Figure S181.** The ESIMS of **26a**. 99

**Figure S182.** The $^{1}$H-NMR (600 MHz, $CD_3OD$) spectrum of **26b**. 99

**Figure S183.** The $^{1}$H-$^{1}$H COSY (600 MHz, $CD_3OD$) spectrum of **26b**. 100

**Figure S184.** The ESIMS of **26b**. 100

**Table S1.** SMILES table of compounds **1**−**26**. 101

**Table S2**. Results of cytotoxicity and antibacterial activity assay of compounds **1**−**26**. 102

**Acquisition Parameter**

| | | | | | | | |
|---|---|---|---|---|---|---|---|
| Source Type | ESI | Ion Polarity | Positive | Set Nebulizer | 0.3 Bar | | |
| Focus | Not active | Set Capillary | 4000 V | Set Dry Heater | 180 °C | | |
| Scan Begin | 50 m/z | Set End Plate Offset | -500 V | Set Dry Gas | 4.0 l/min | | |
| Scan End | 1500 m/z | Set Collision Cell RF | 600.0 Vpp | Set Divert Valve | Source | | |

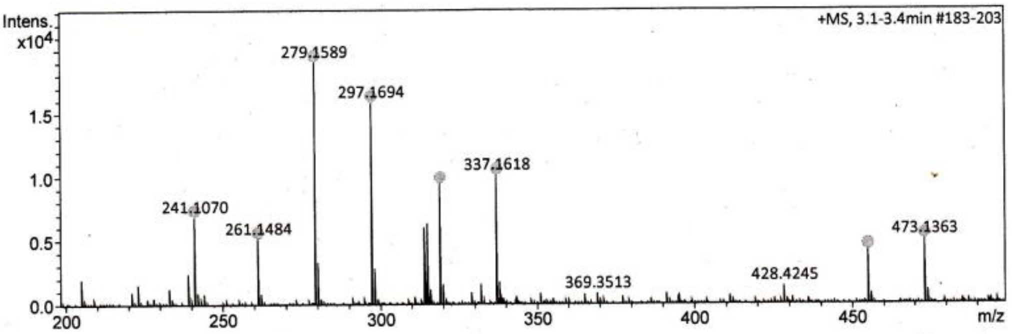


| Meas. m/z | # | Ion Formula | m/z | err [ppm] | mSigma | # mSigma | Score | rdb | e⁻ Conf | N-Rule |
|---|---|---|---|---|---|---|---|---|---|---|
| 241.1070 | 1 | C12H17O5 | 241.1071 | 0.0 | 3.9 | 1 | 100.00 | 4.5 | even | ok |
| 261.1484 | 1 | C16H21O3 | 261.1485 | 0.6 | 12.1 | 1 | 100.00 | 6.5 | even | ok |
| 279.1589 | 1 | C16H23O4 | 279.1591 | 0.5 | 3.0 | 1 | 100.00 | 5.5 | even | ok |
| 297.1694 | 1 | C16H25O5 | 297.1697 | 1.0 | 1.6 | 1 | 100.00 | 4.5 | even | ok |
| 319.1513 | 1 | C16H24NaO5 | 319.1516 | 1.1 | 7.4 | 1 | 89.02 | 4.5 | even | ok |
| | 2 | C14H19N6O3 | 319.1513 | 0.2 | 7.6 | 2 | 100.00 | 8.5 | even | ok |

**Figure S1.** The HRESIMS of compound **1**.

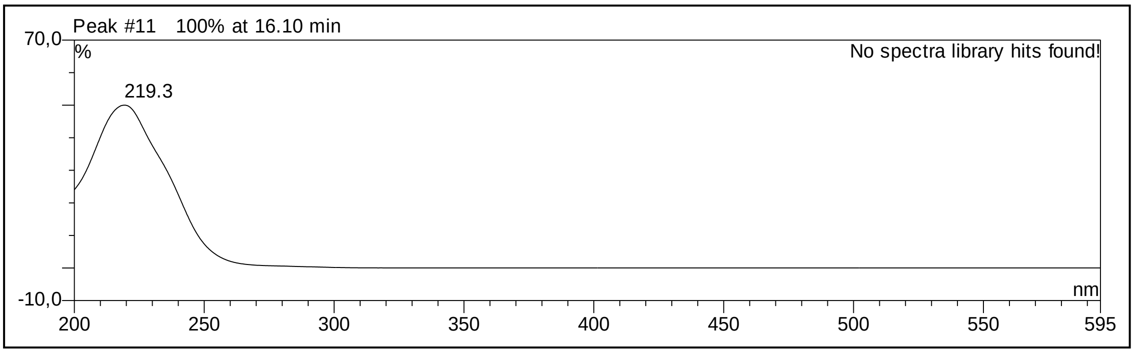


**Figure S2.** The UV spectrum of compound **1**.

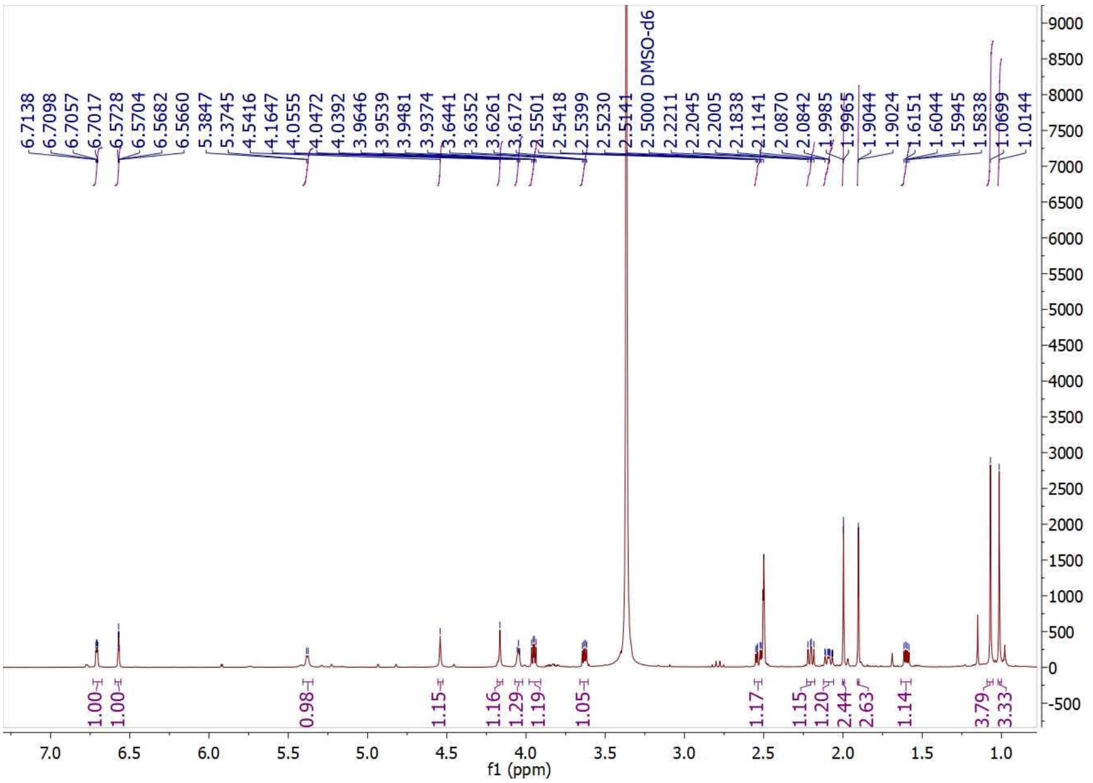


**Figure S3.** The $^{1}$H-NMR (600 MHz, DMSO-$d_6$) spectrum of compound **1**.

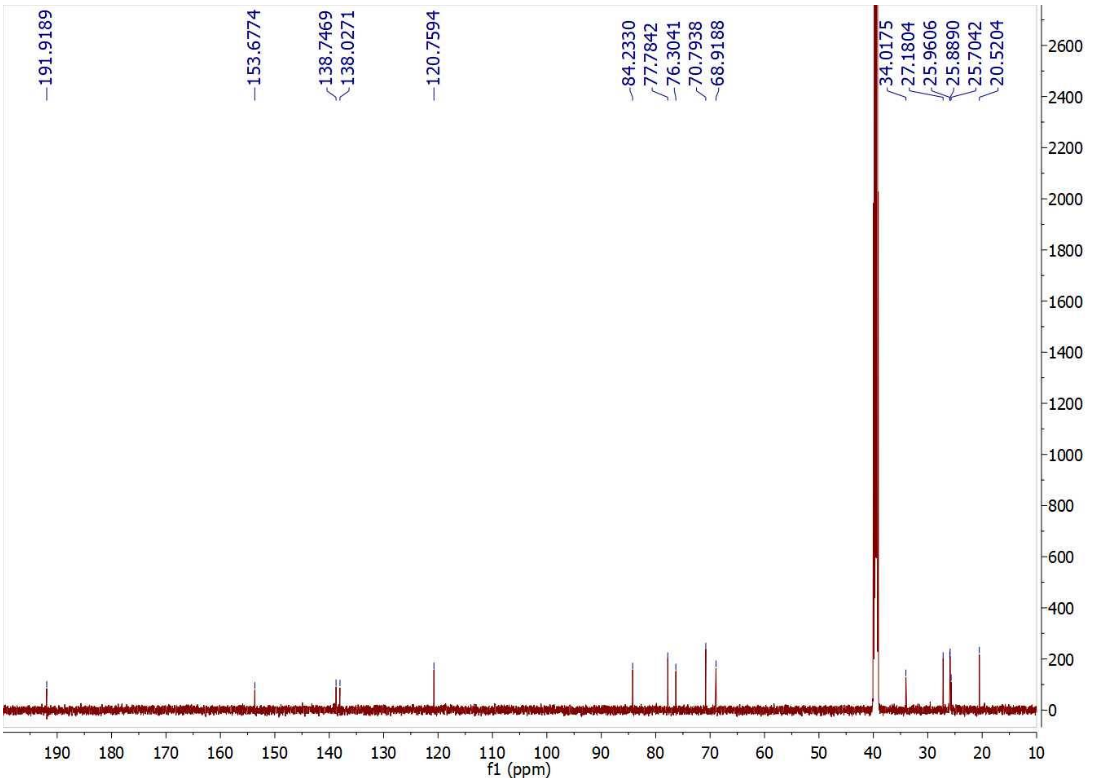


**Figure S4.** The $^{13}$C-NMR (150 MHz, DMSO-$d_6$) spectrum of compound **1**.

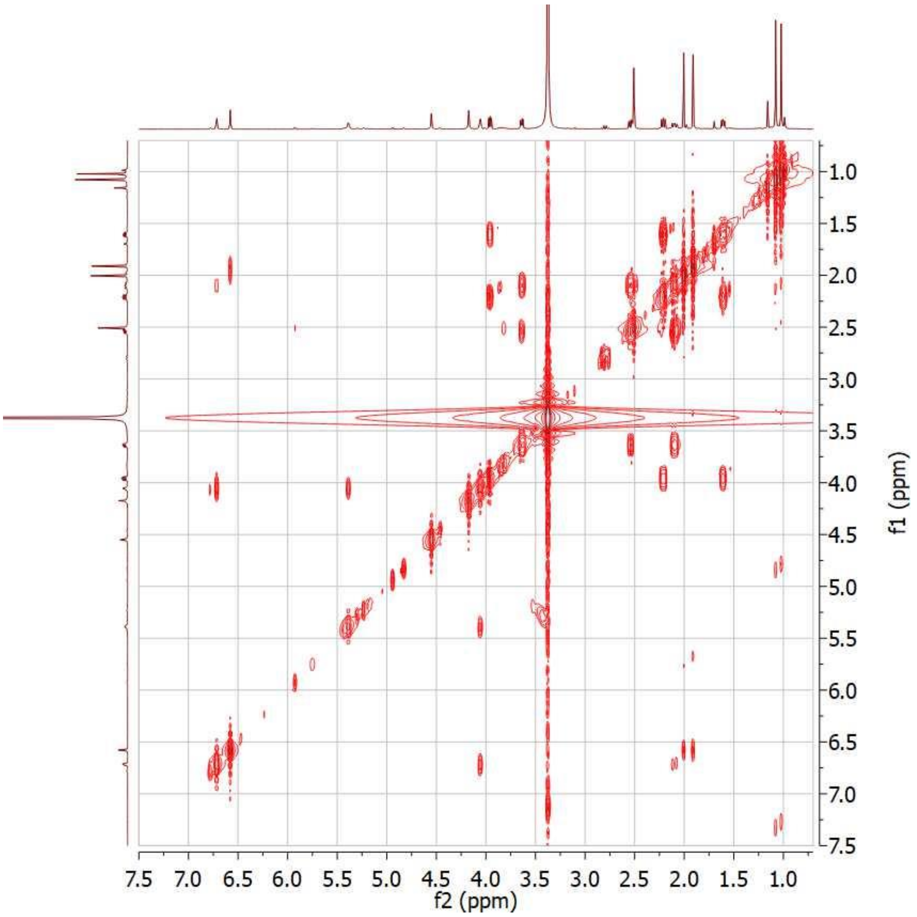


**Figure S5.** The $^{1}$H-$^{1}$H COSY (600 MHz, DMSO-$d_6$) spectrum of compound **1**.

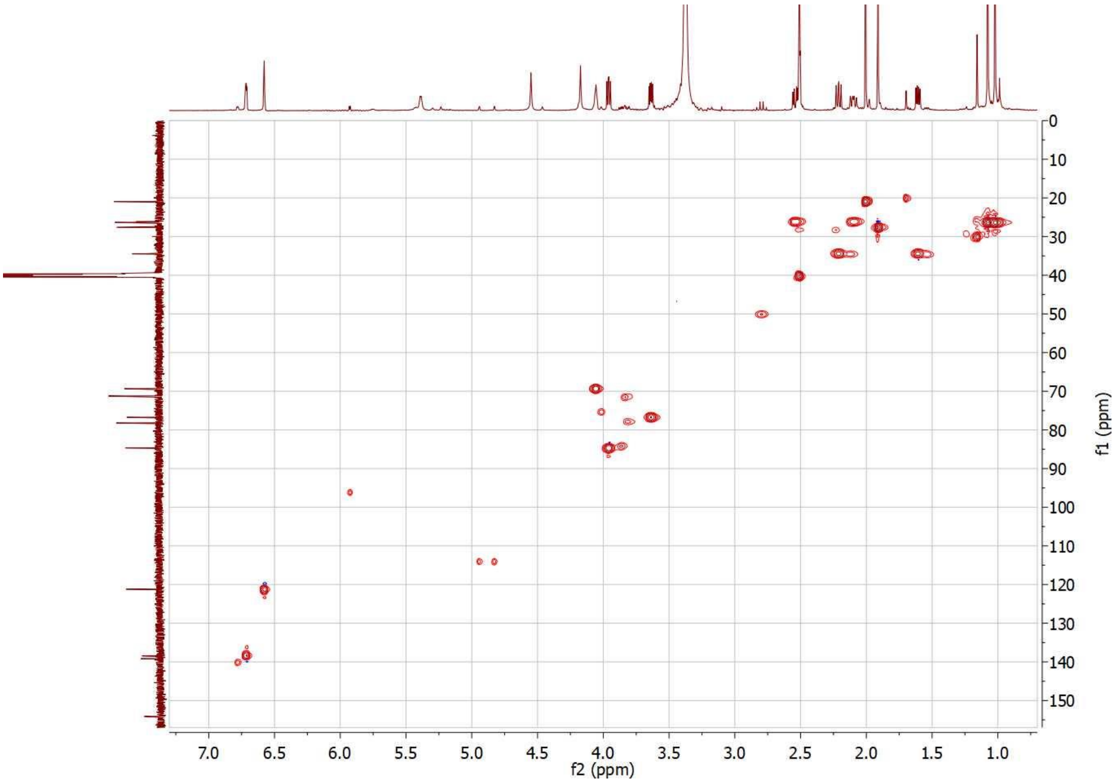


**Figure S6.** The HSQC (600 MHz, DMSO-$d_6$) spectrum of compound **1**.

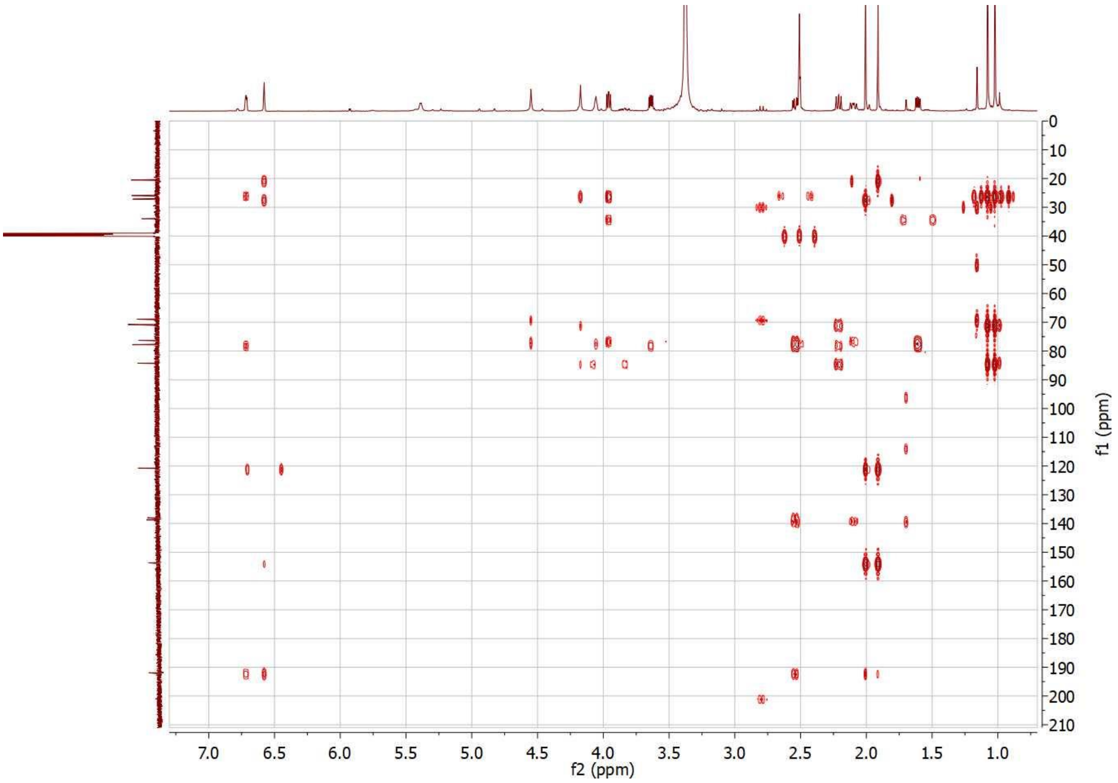


**Figure S7.** The HMBC (600 MHz, DMSO-$d_6$) spectrum of compound **1**.

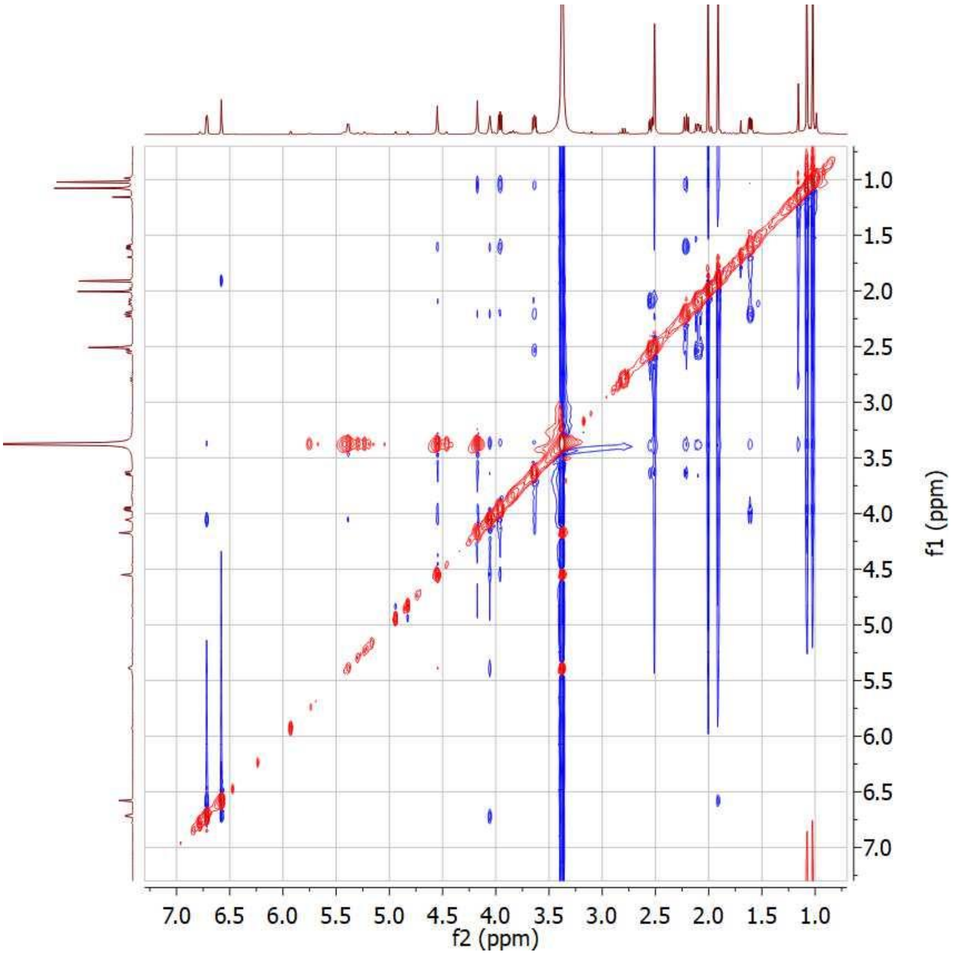


**Figure S8.** The ROESY (600 MHz, DMSO-$d_6$) spectrum of compound **1**.

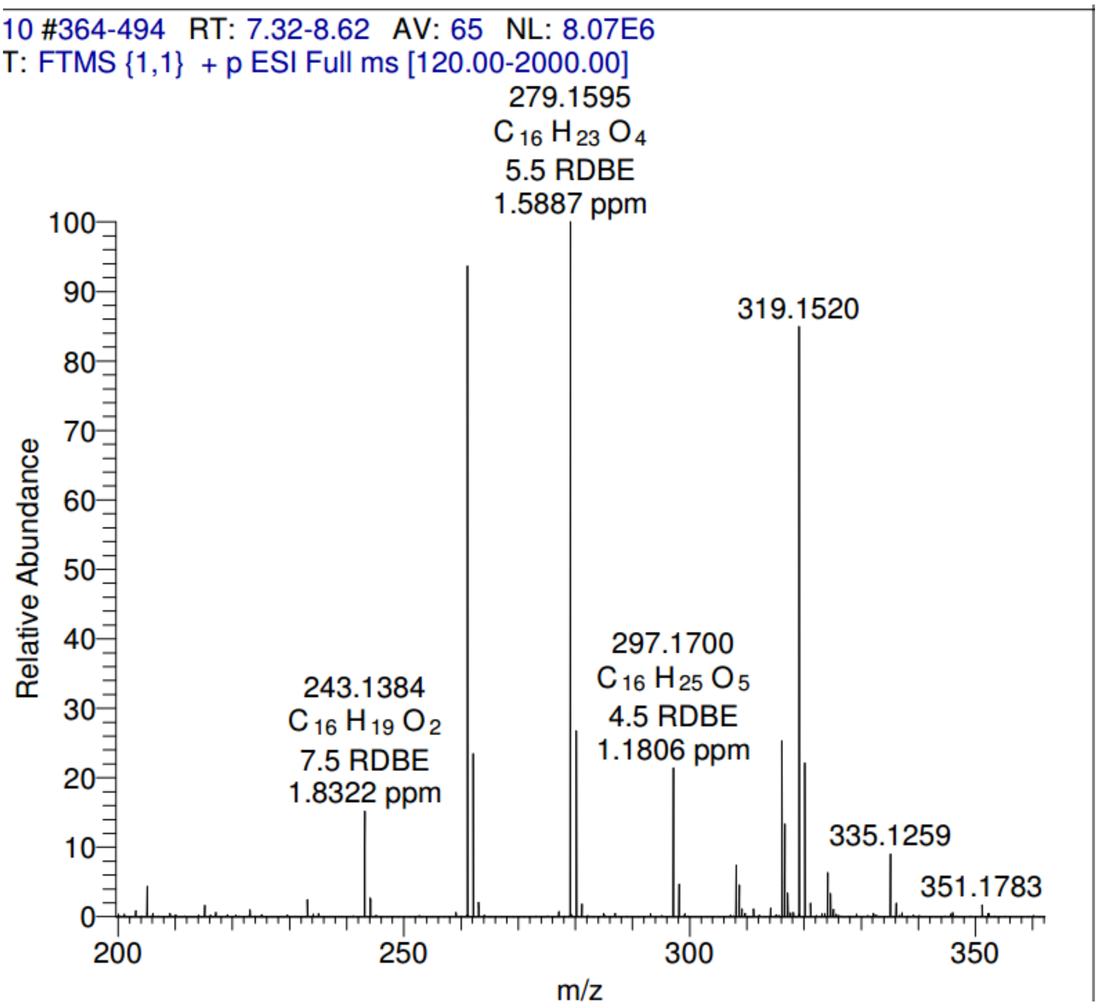


**Figure S9.** The HRESIMS of compound **2**.

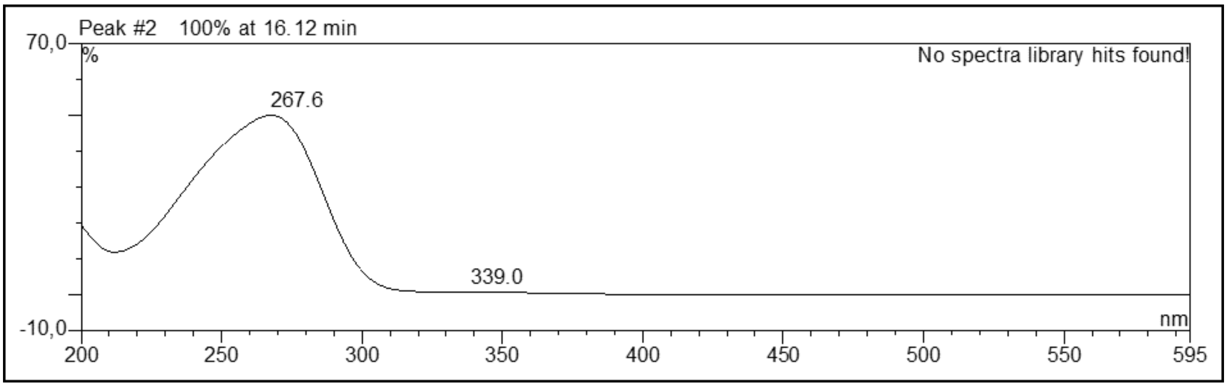


**Figure S10.** The UV spectrum of compound **2**.

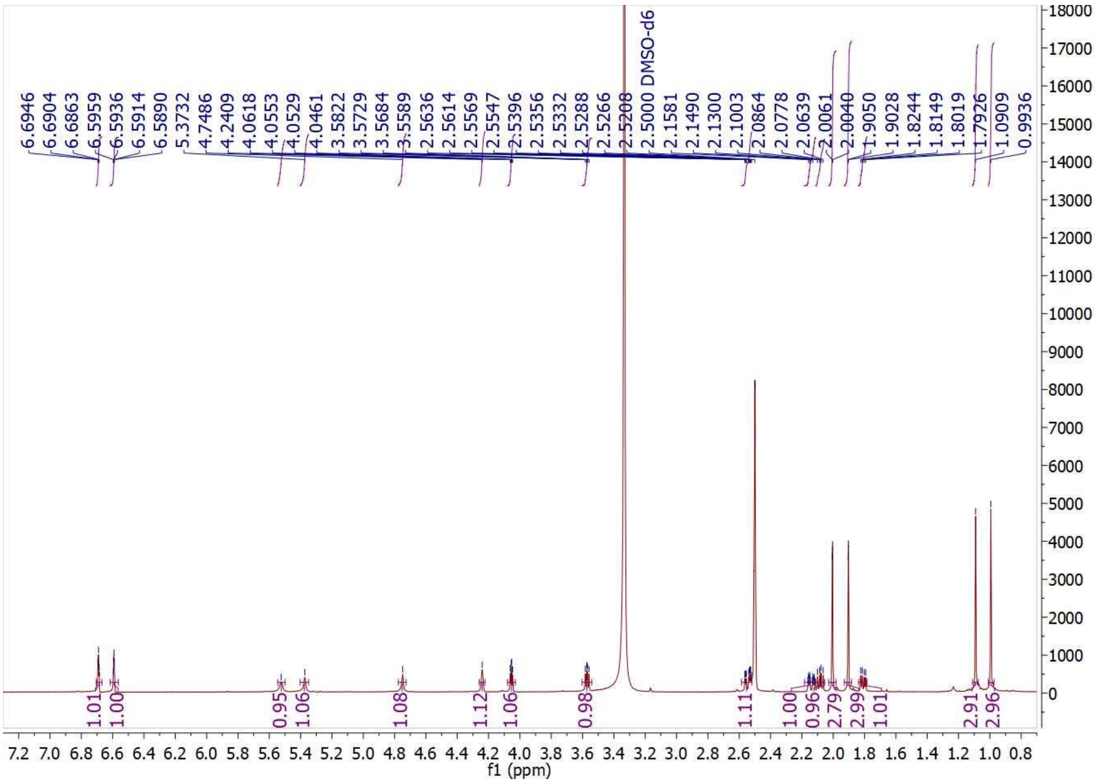


**Figure S11.** The $^{1}$H-NMR (600 MHz, DMSO-$d_6$) spectrum of compound **2**.

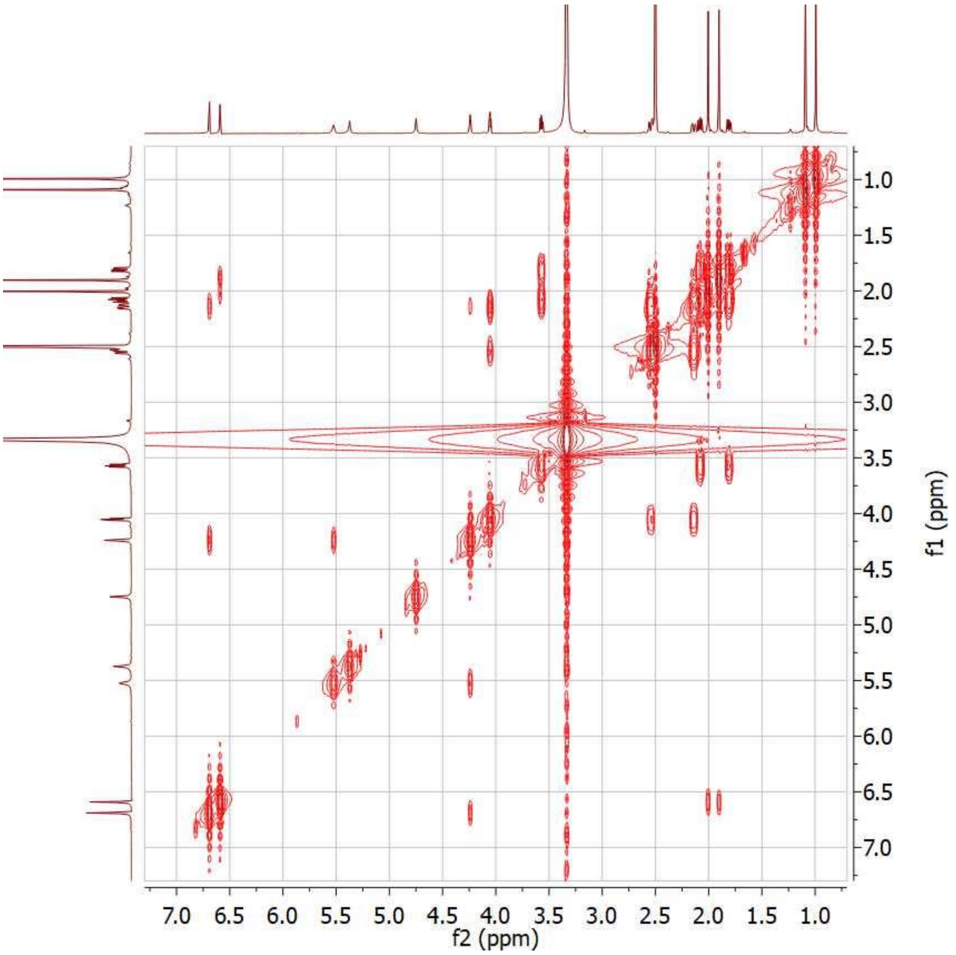


**Figure S12.** The $^{1}$H-$^{1}$H COSY (600 MHz, DMSO-$d_6$) spectrum of compound **2**.

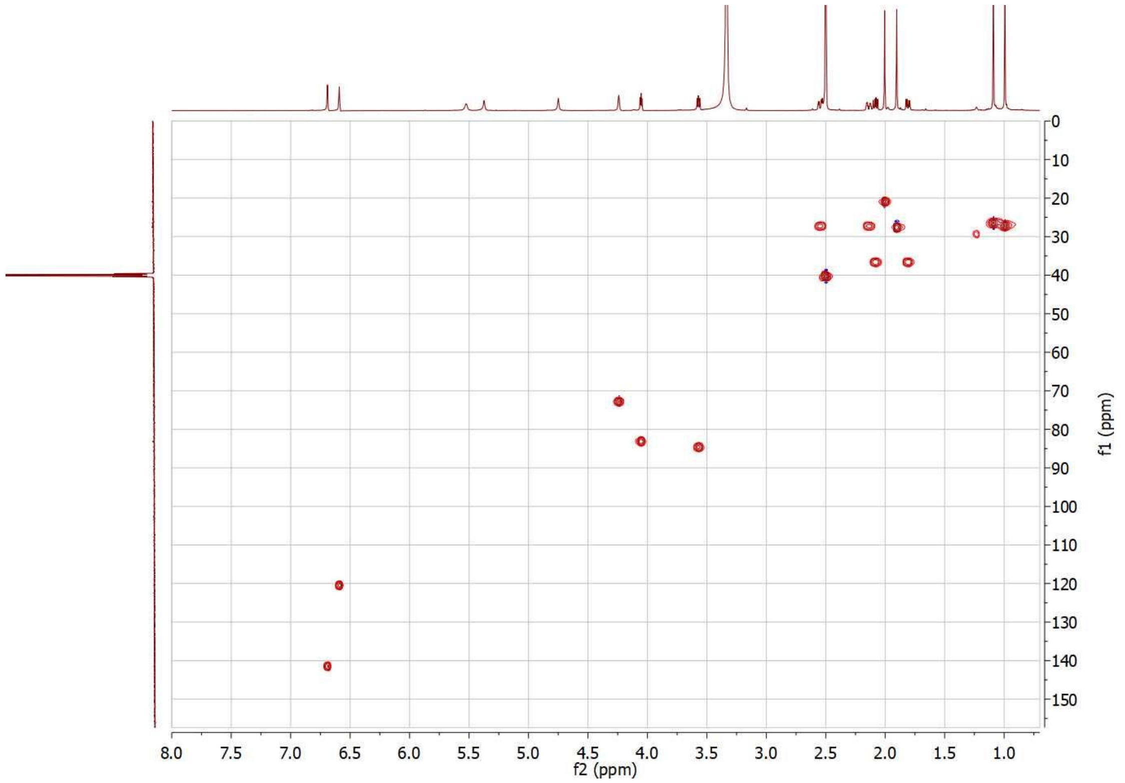


**Figure S13.** The HSQC (600 MHz, DMSO-$d_6$) spectrum of compound **2**.

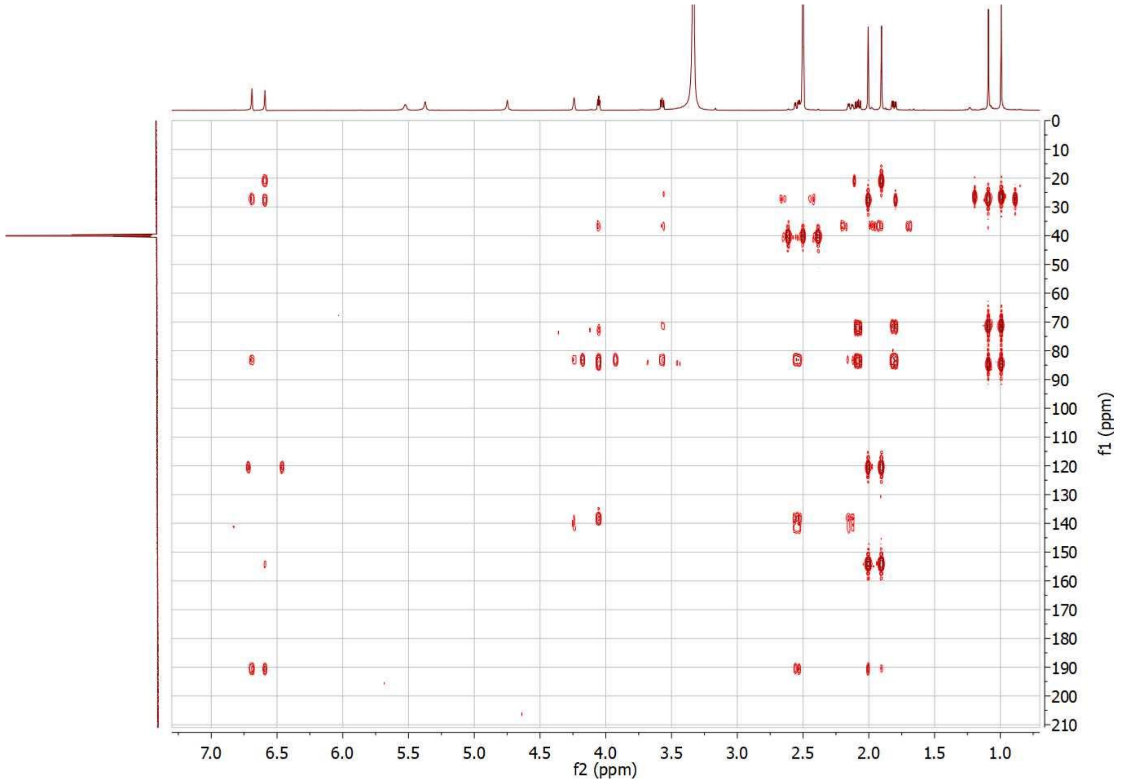


**Figure S14.** The HMBC (600 MHz, DMSO-$d_6$) spectrum of compound **2**.

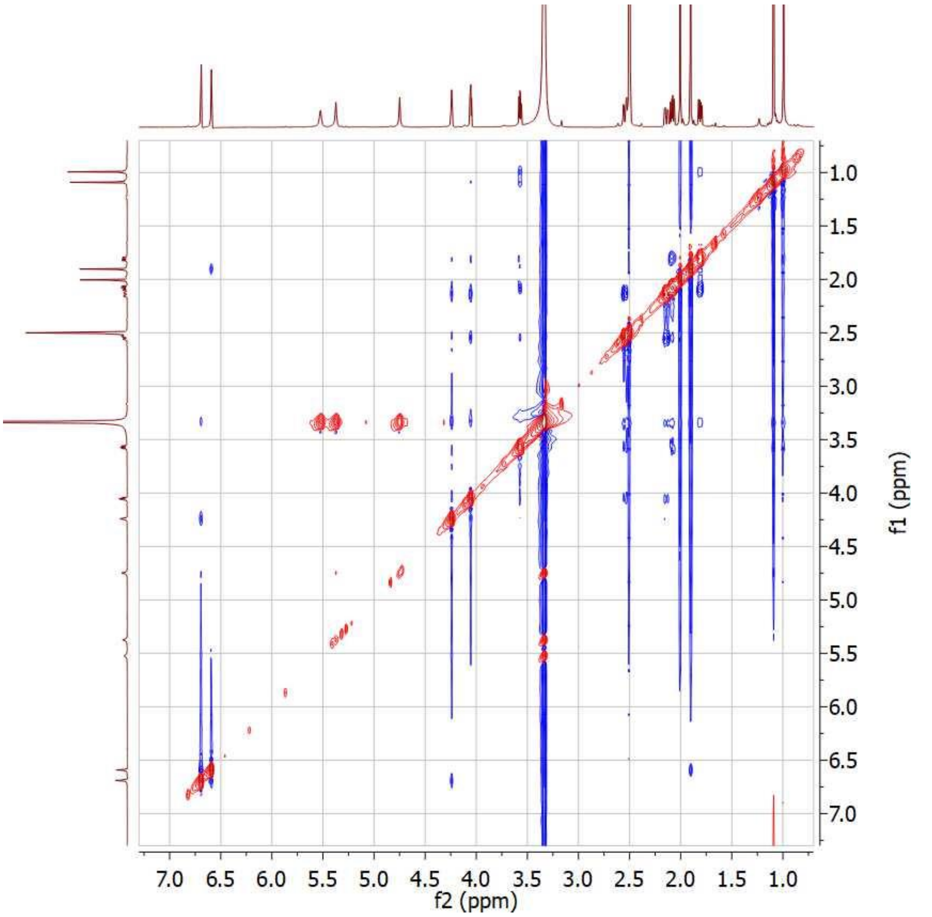


**Figure S15.** The ROESY (600 MHz, DMSO-$d_6$) spectrum of compound **2**.

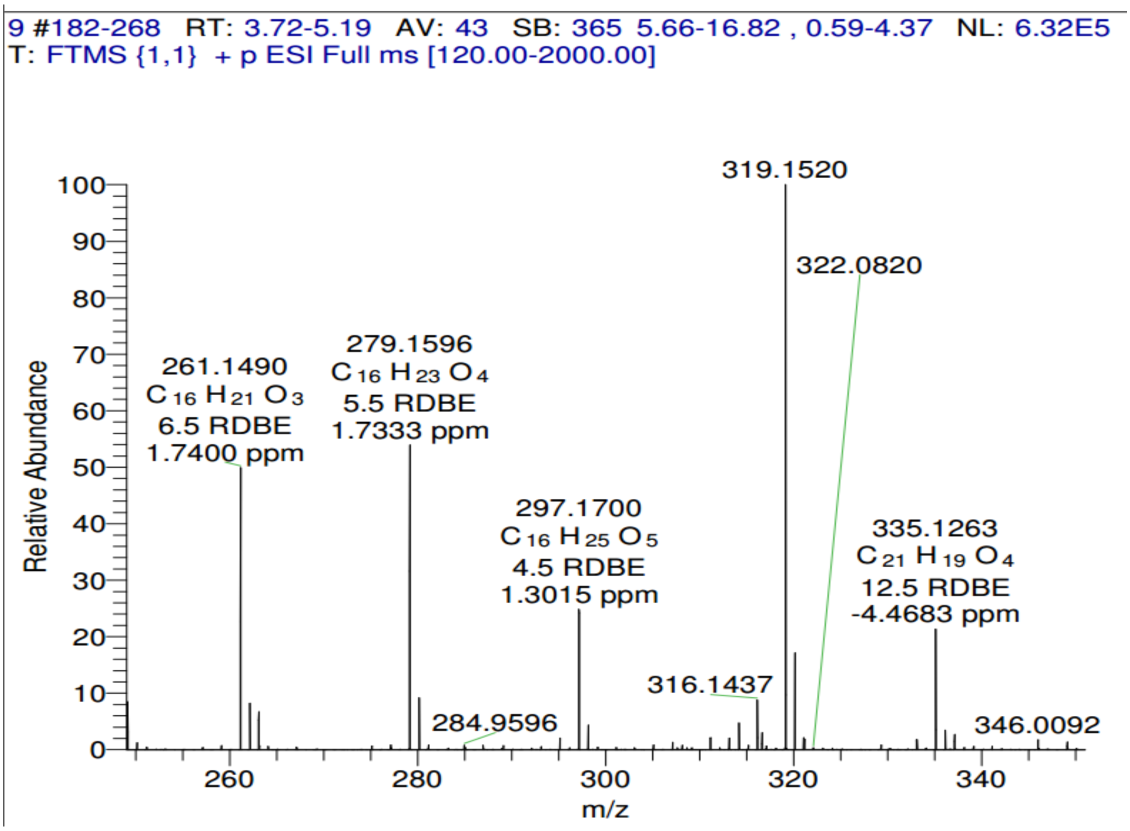


**Figure S16.** The HRESIMS of compound **3**.

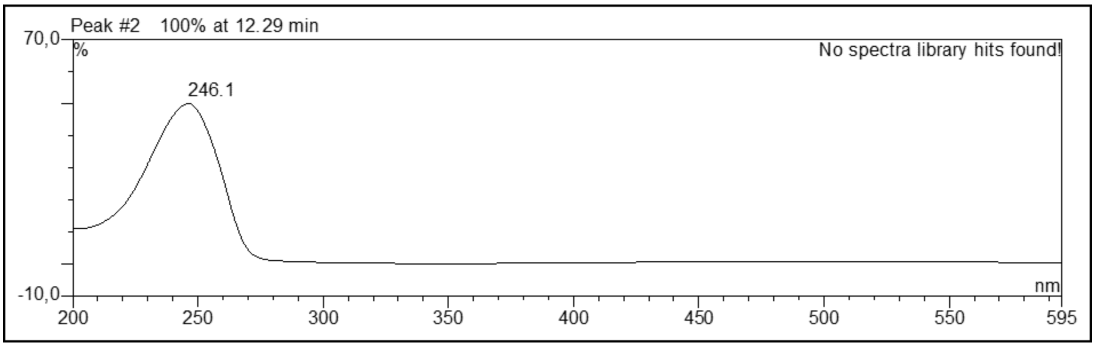


**Figure S17.** The UV spectrum of compound **3**.

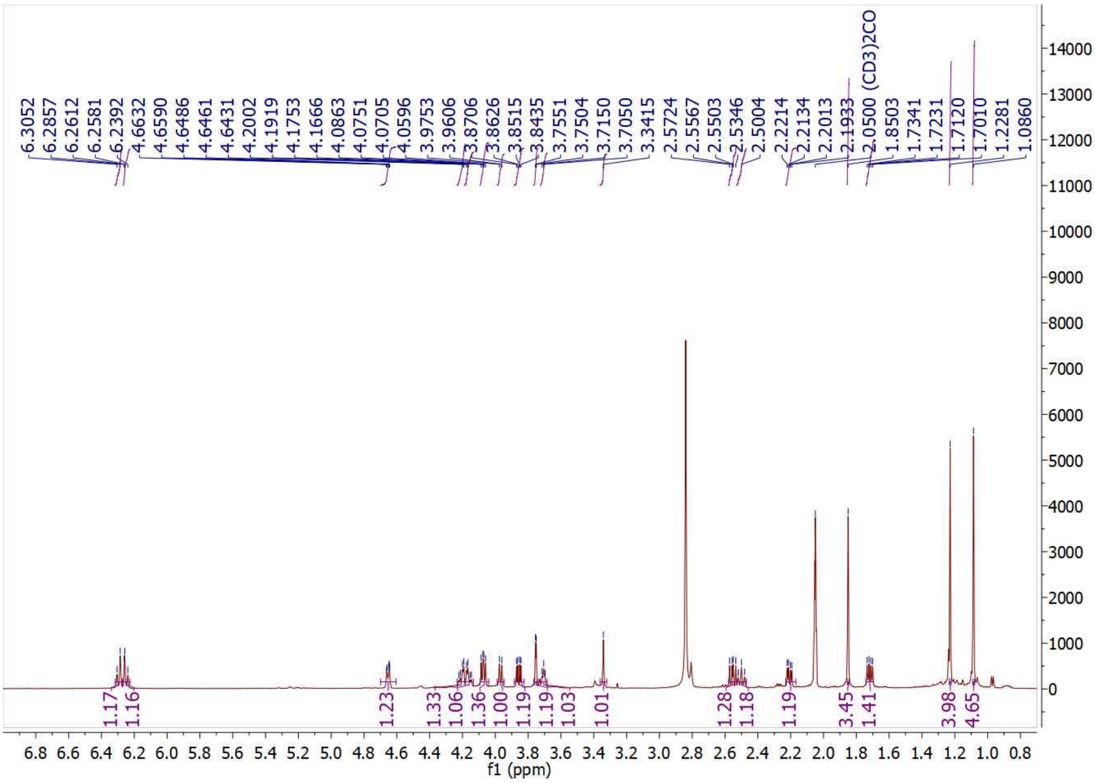


**Figure S18.** The $^{1}$H-NMR (600 MHz, Acetone-$d_6$) spectrum of compound **3**.

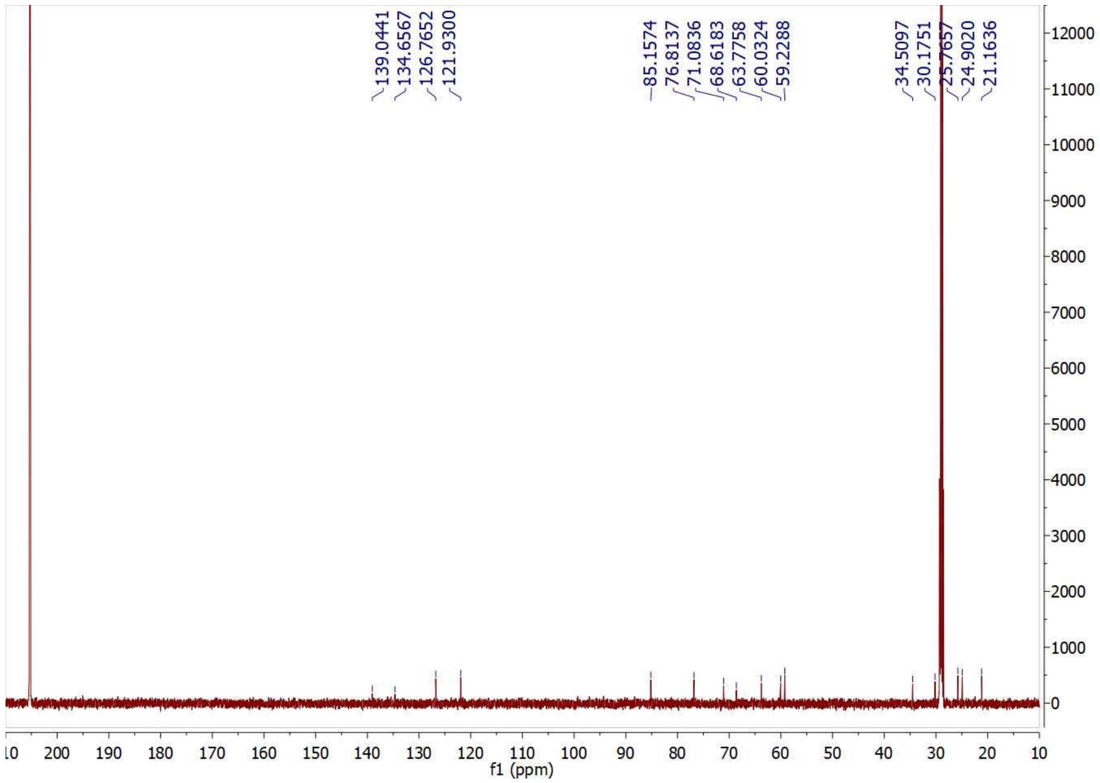


**Figure S19.** The $^{13}$C-NMR (150 MHz, Acetone-$d_6$) spectrum of compound **3**.

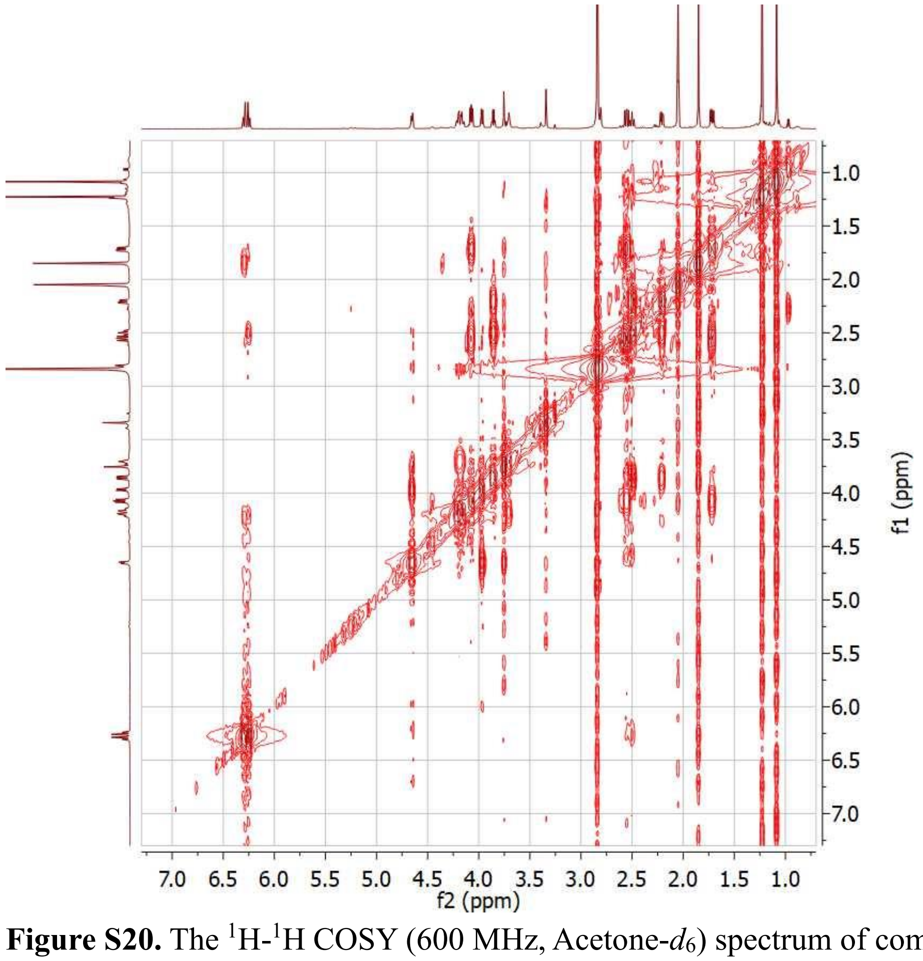


**Figure S20.** The $^1$H-$^1$H COSY (600 MHz, Acetone-$d_6$) spectrum of compound **3**.

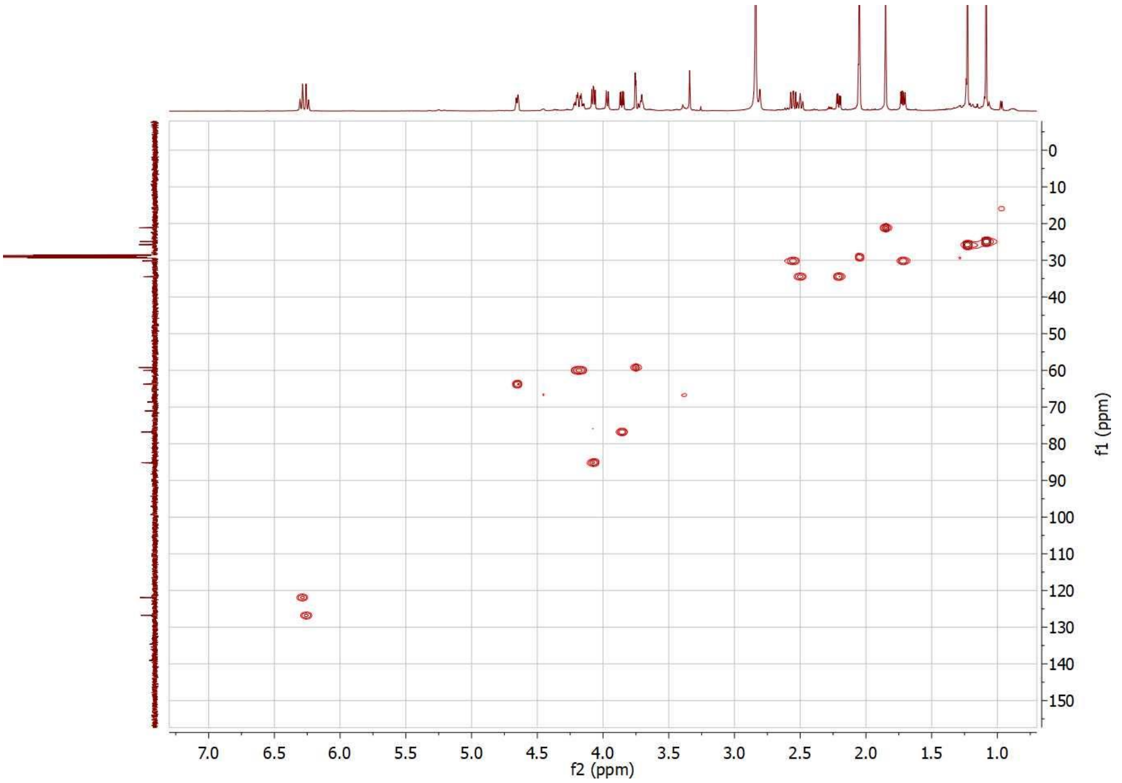


**Figure S21.** The HSQC (600 MHz, Acetone-$d_6$) spectrum of compound **3**.

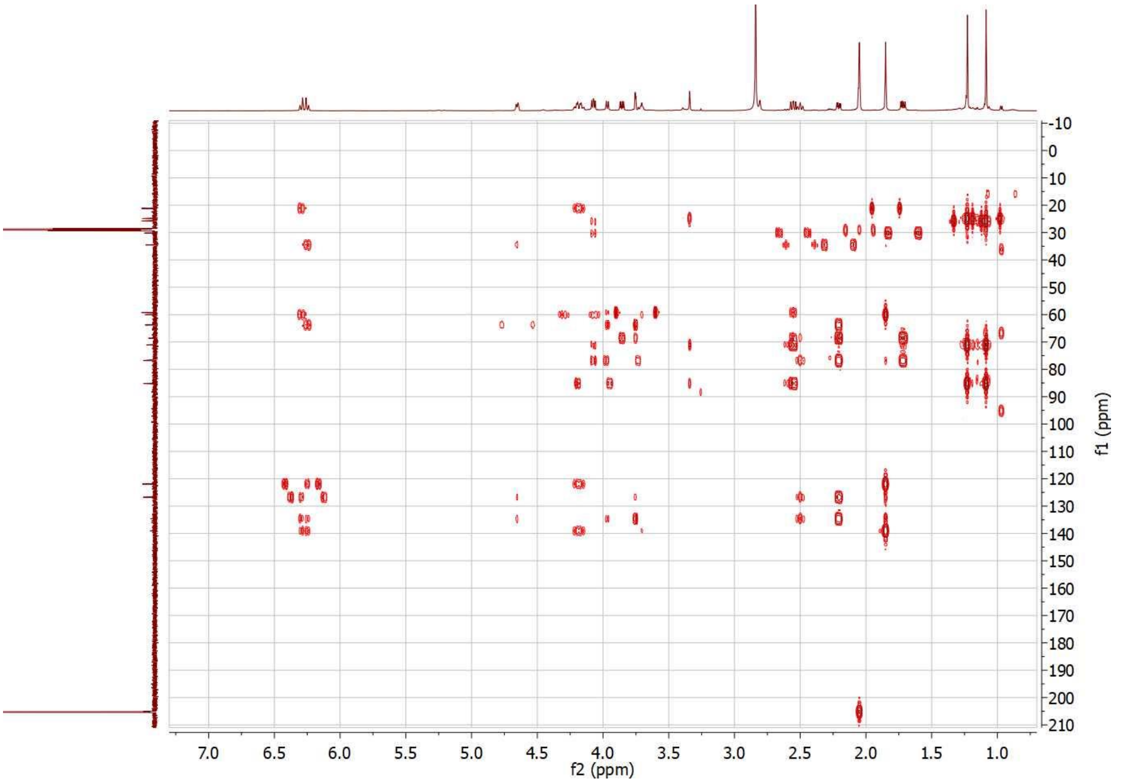


**Figure S22.** The HMBC (600 MHz, Acetone-$d_6$) spectrum of compound **3**.

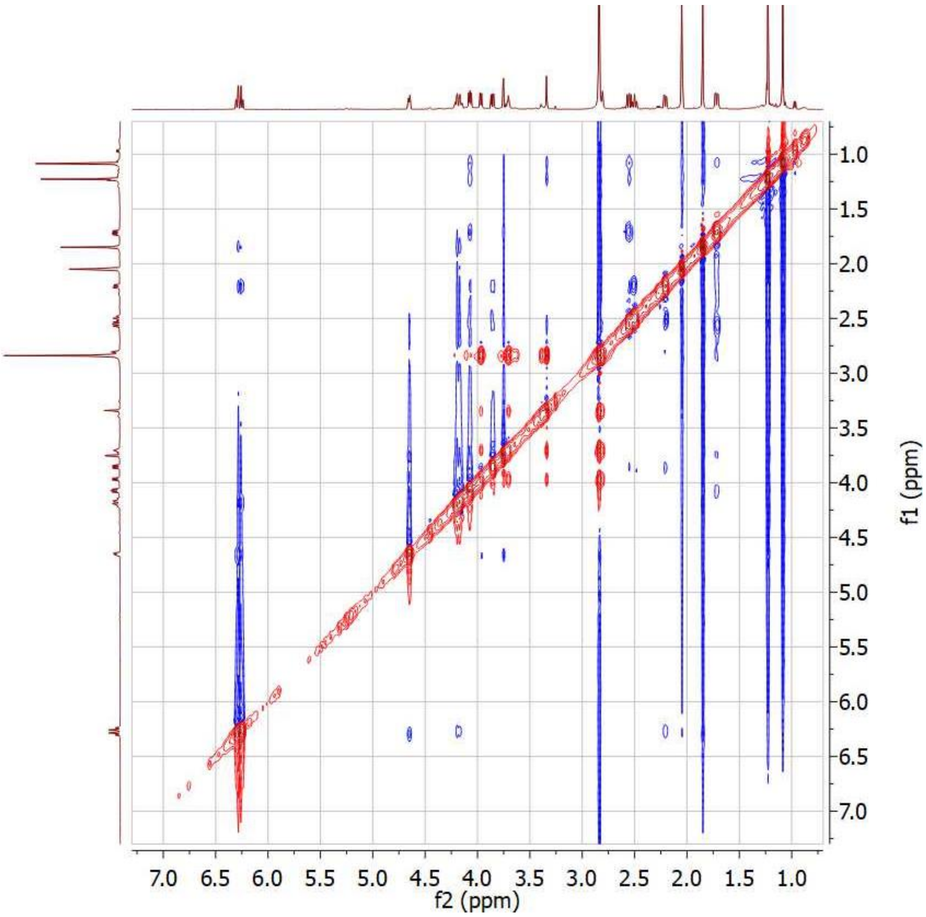


**Figure S23.** The ROESY (600 MHz, Acetone-d$_6$) spectrum of compound **3**.

**Acquisition Parameter**

| | | | | | |
|---|---|---|---|---|---|
| Source Type | ESI | Ion Polarity | Positive | Set Nebulizer | 0.3 Bar |
| Focus | Not active | Set Capillary | 4000 V | Set Dry Heater | 180 °C |
| Scan Begin | 50 m/z | Set End Plate Offset | -500 V | Set Dry Gas | 4.0 l/min |
| Scan End | 1500 m/z | Set Collision Cell RF | 600.0 Vpp | Set Divert Valve | Source |

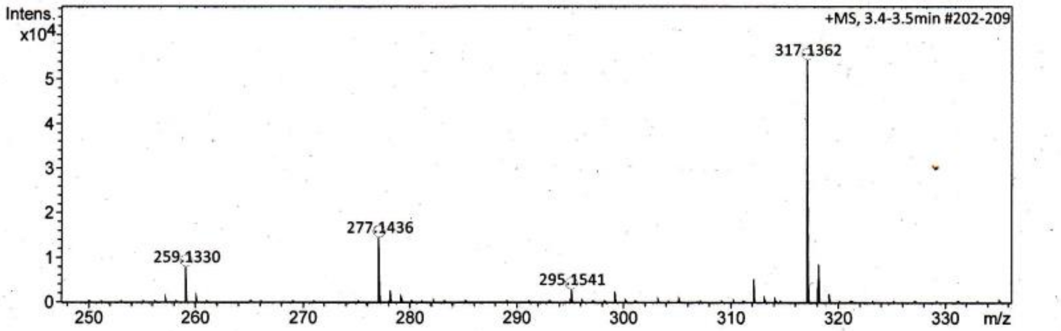


| Meas. m/z | # | Ion Formula | m/z | err [ppm] | mSigma | # mSigma | Score | rdb | e⁻ Conf | N-Rule |
|---|---|---|---|---|---|---|---|---|---|---|
| 259.1330 | 1 | C16H19O3 | 259.1329 | -0.4 | 18.4 | 1 | 100.00 | 7.5 | even | ok |
| 277.1436 | 1 | C16H21O4 | 277.1434 | -0.7 | 37.7 | 1 | 100.00 | 6.5 | even | ok |
| 295.1541 | 1 | C16H23O5 | 295.1540 | -0.4 | 55.0 | 1 | 100.00 | 5.5 | even | ok |
| 317.1362 | 1 | C16H22NaO5 | 317.1359 | -0.9 | 13.4 | 1 | 100.00 | 5.5 | even | ok |

**Figure S24.** The HRESIMS of compound **4**.

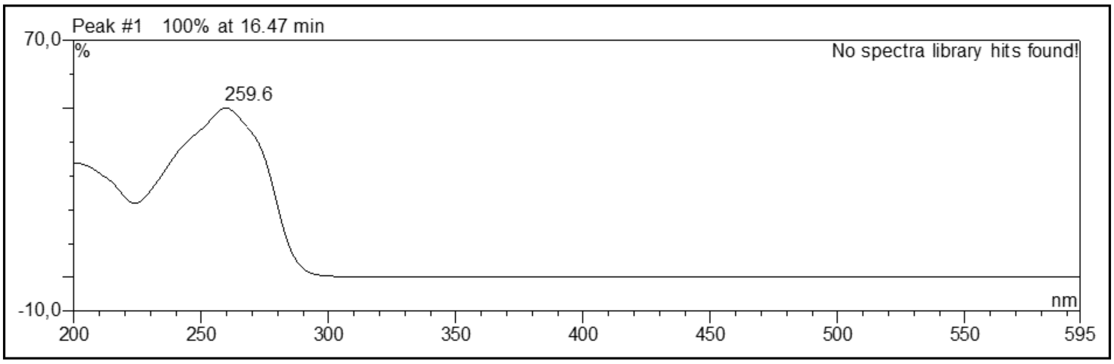


**Figure S25.** The UV spectrum of compound **4**.

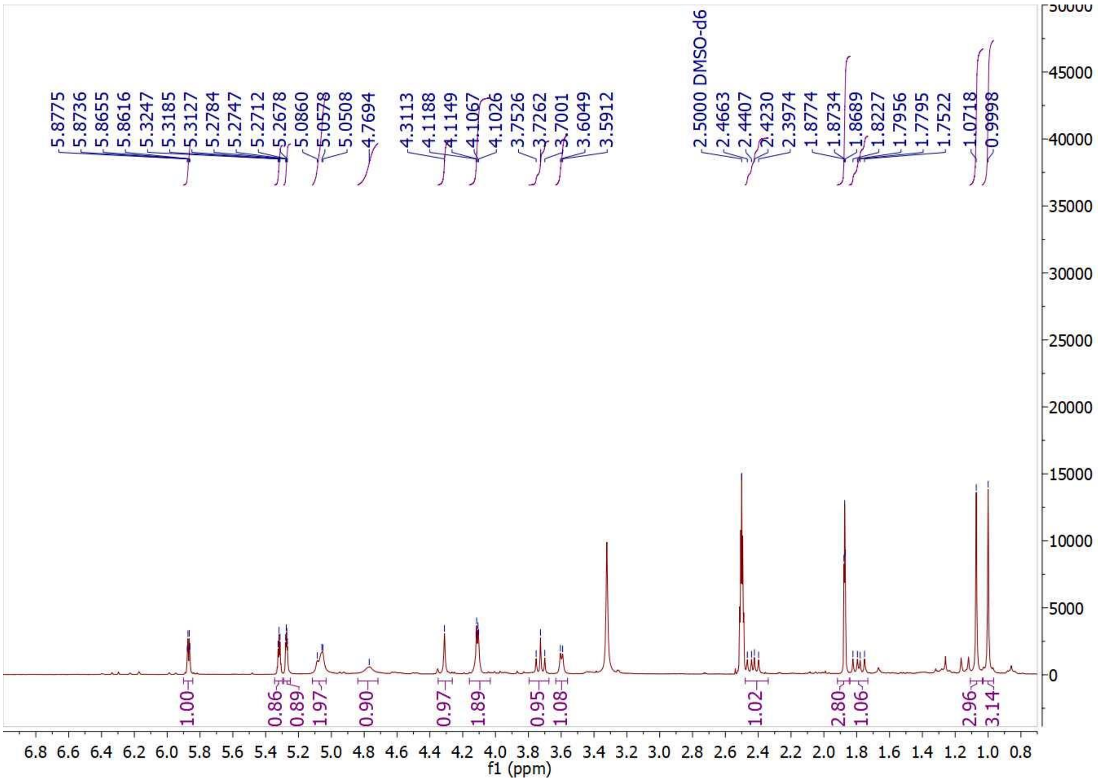


**Figure S26.** The $^{1}$H-NMR (600 MHz, DMSO-$d_6$) spectrum of compound **4**.

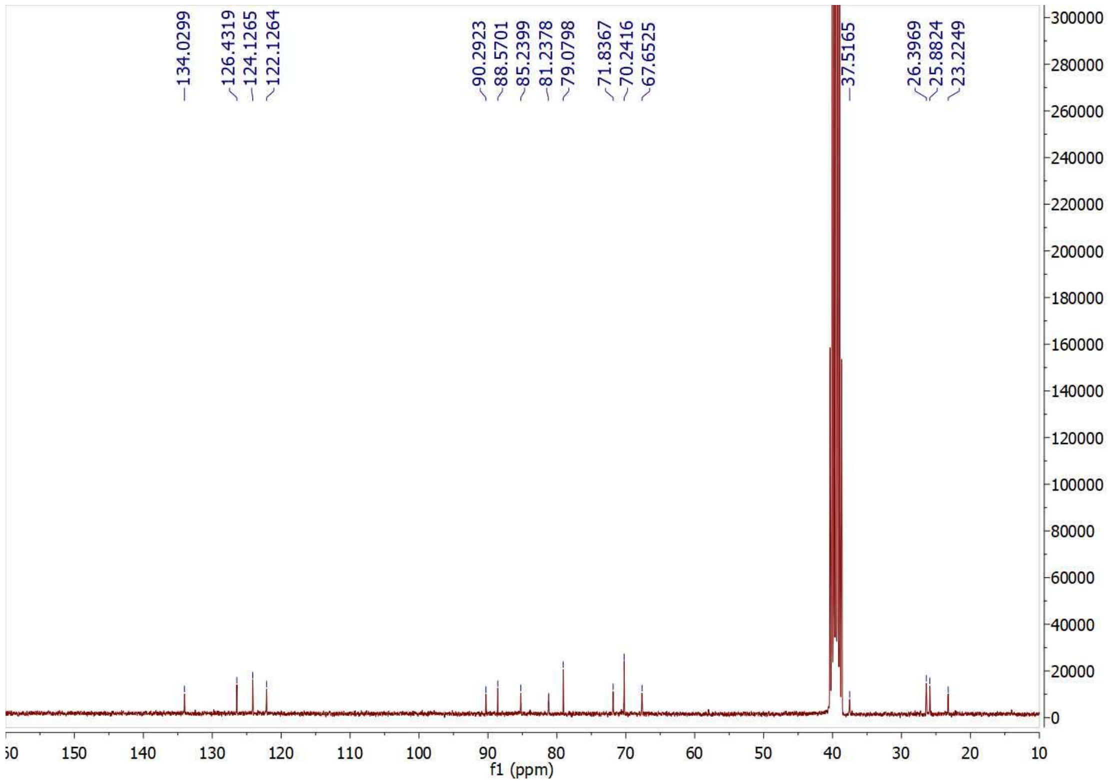


**Figure S27.** The $^{13}$C-NMR (150 MHz, DMSO-$d_6$) spectrum of compound **4**.

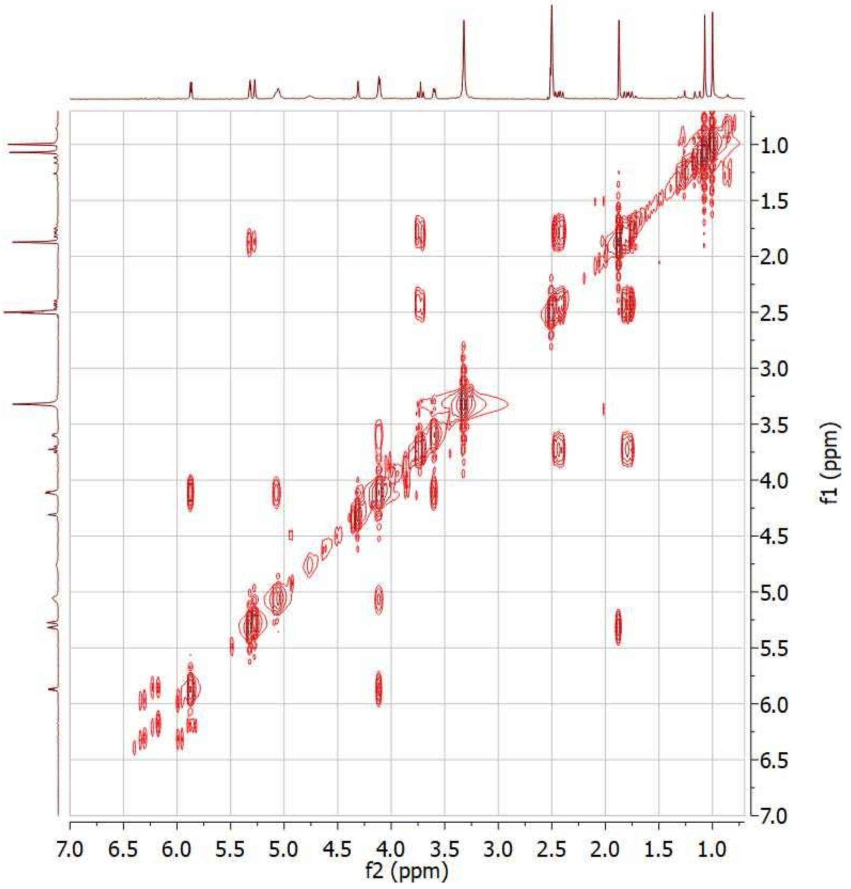


**Figure S28.** The $^{1}$H-$^{1}$H COSY (600 MHz, DMSO-$d_6$) spectrum of compound **4**.

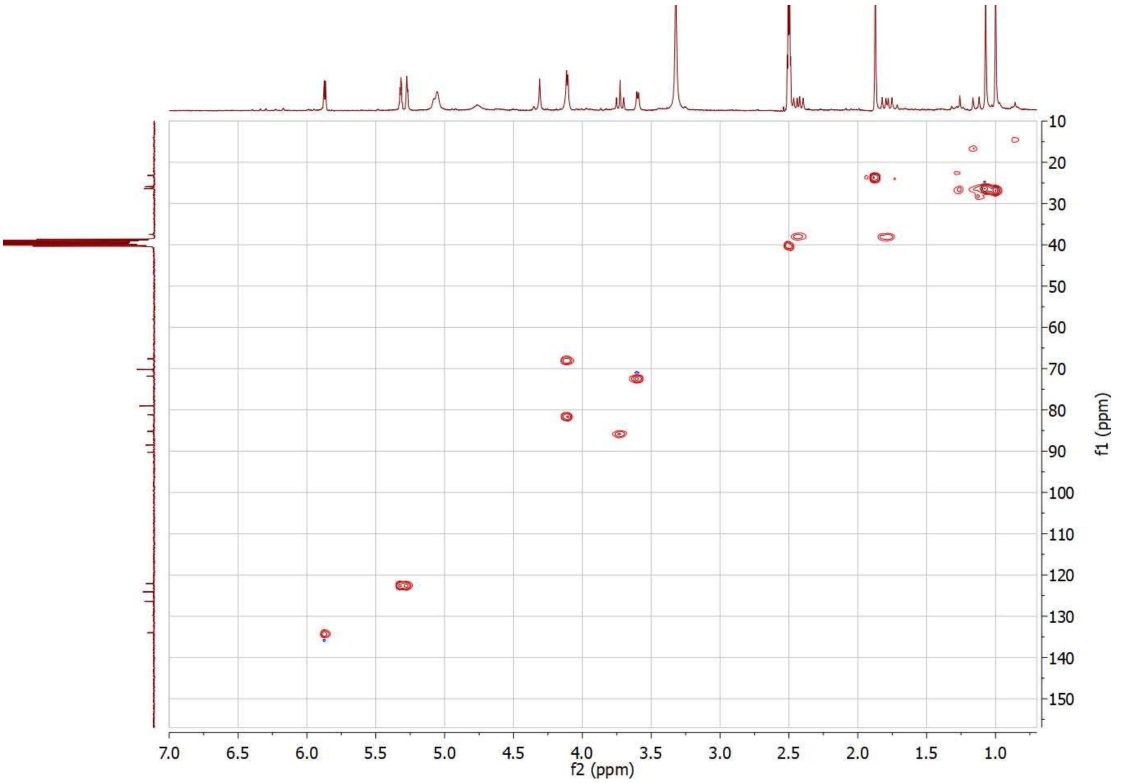


**Figure S29.** The HSQC (600 MHz, DMSO-$d_6$) spectrum of compound **4**.

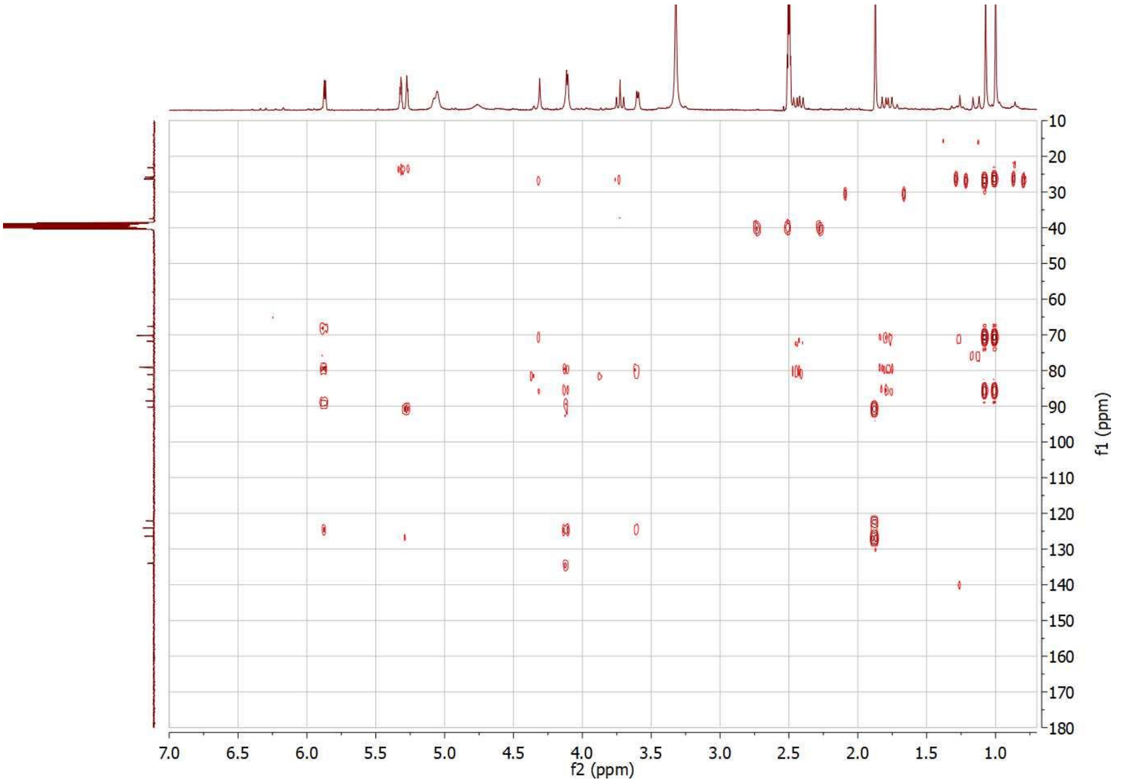


**Figure S30.** The HMBC (600MHz, DMSO-$d_6$) spectrum of compound **4**.

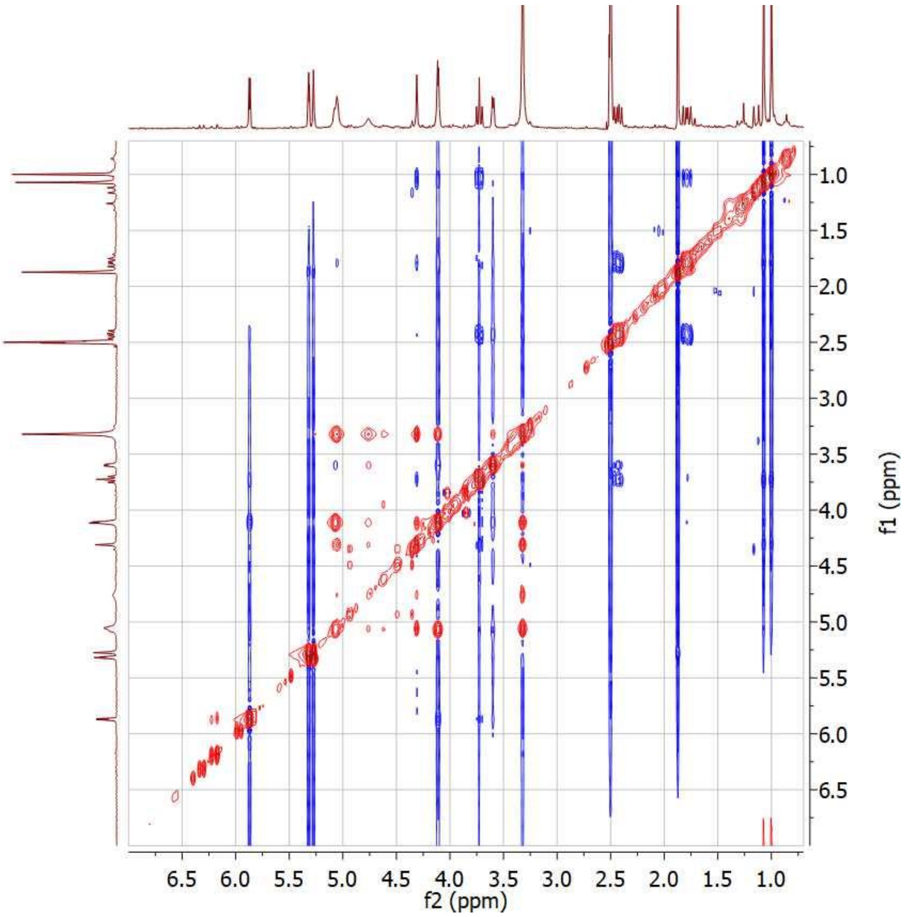


**Figure S31.** The ROESY (600 MHz, DMSO-$d_6$) spectrum of compound **4**.

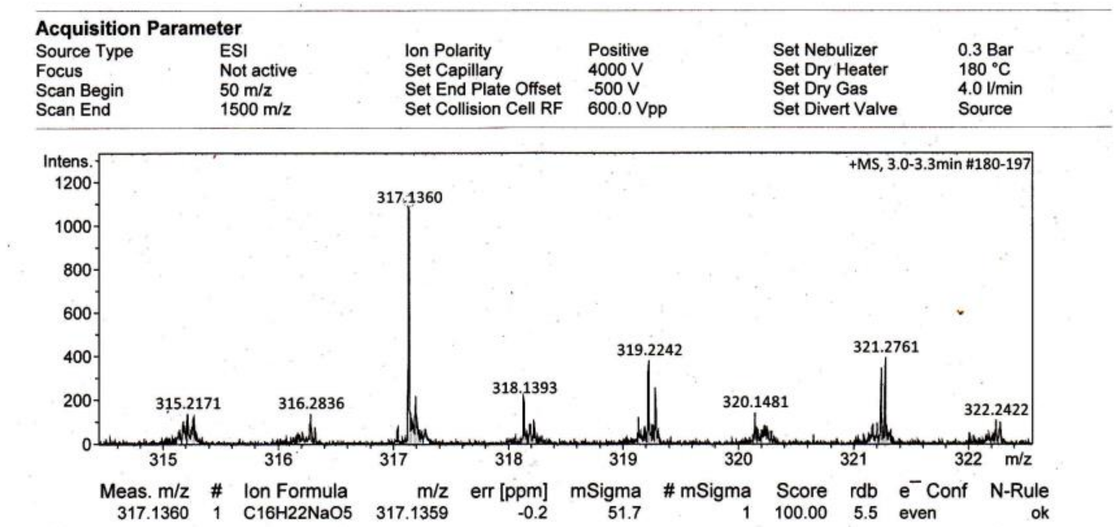


**Figure S32.** The HRESIMS of compound **5**.

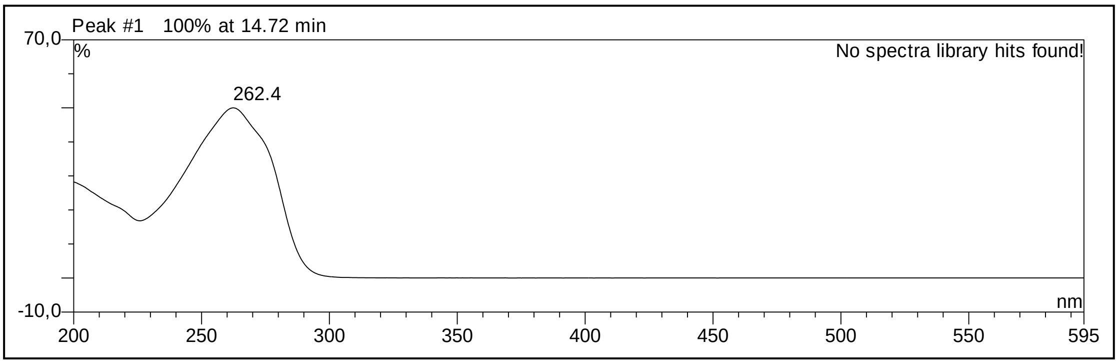


**Figure S33.** The UV spectrum of compound **5**.

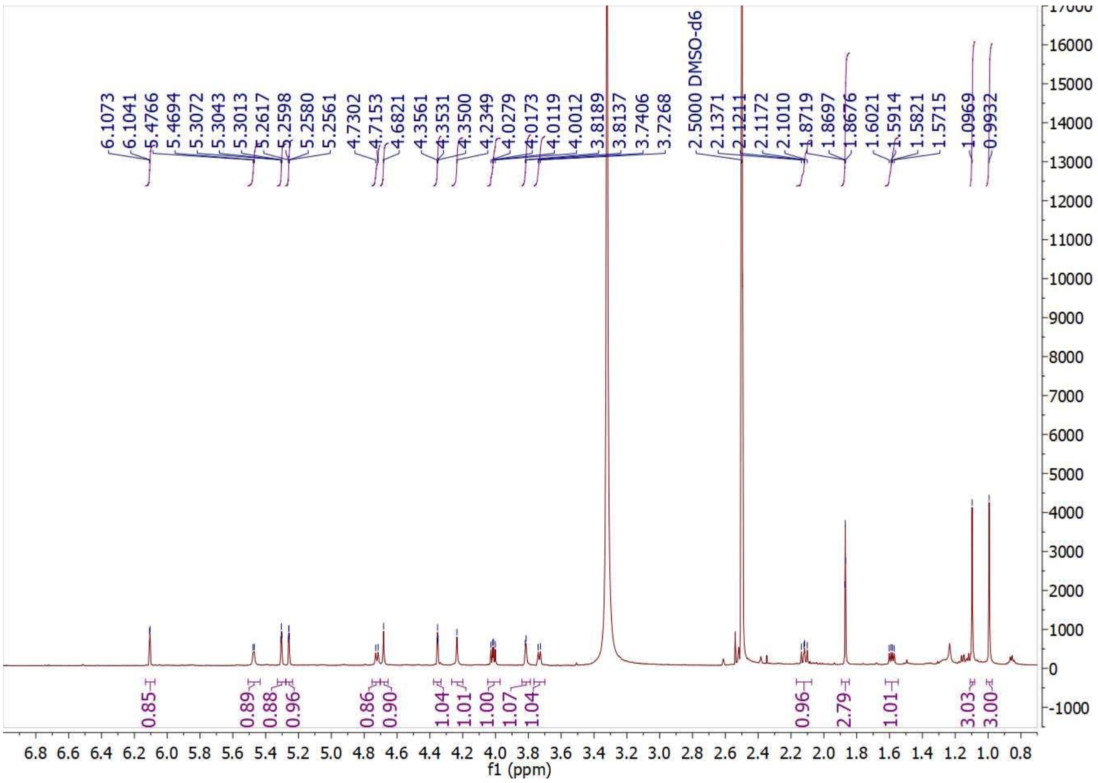


**Figure S34.** The $^1$H-NMR (600 MHz, DMSO-$d_6$) spectrum of compound **5**.

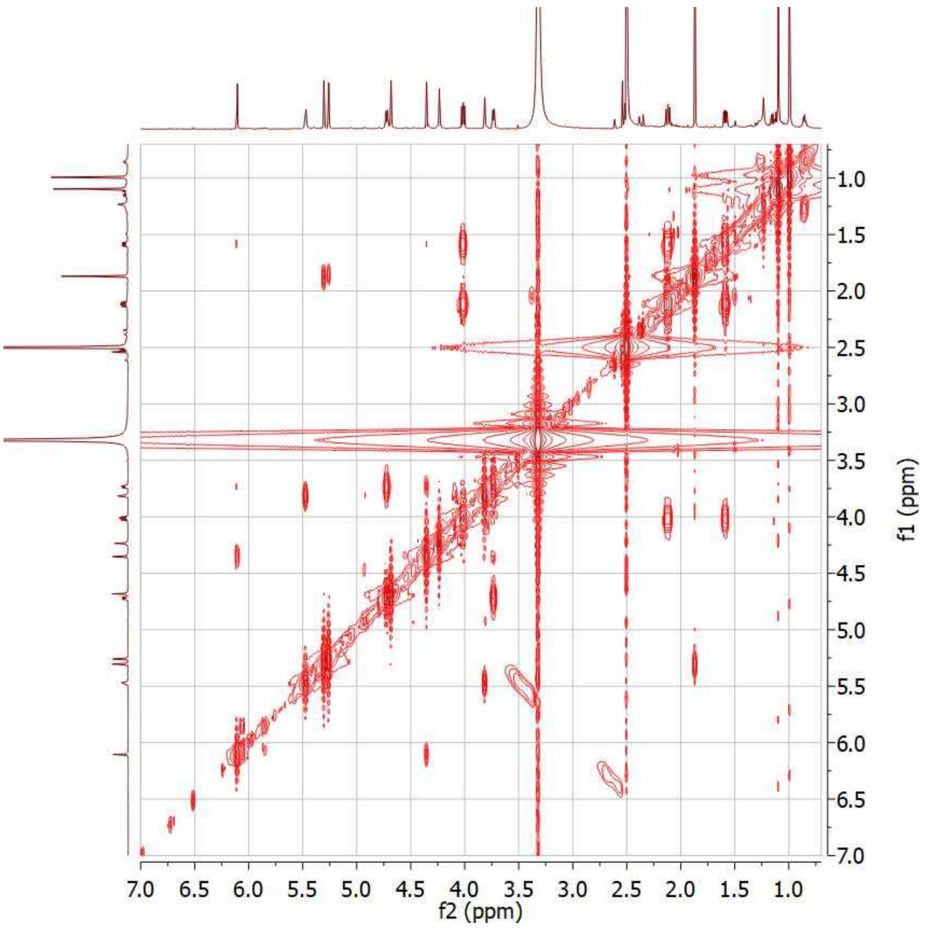


**Figure S35.** The $^{1}H$-$^{1}H$ COSY (600 MHz, DMSO-$d_6$) spectrum of compound **5**.

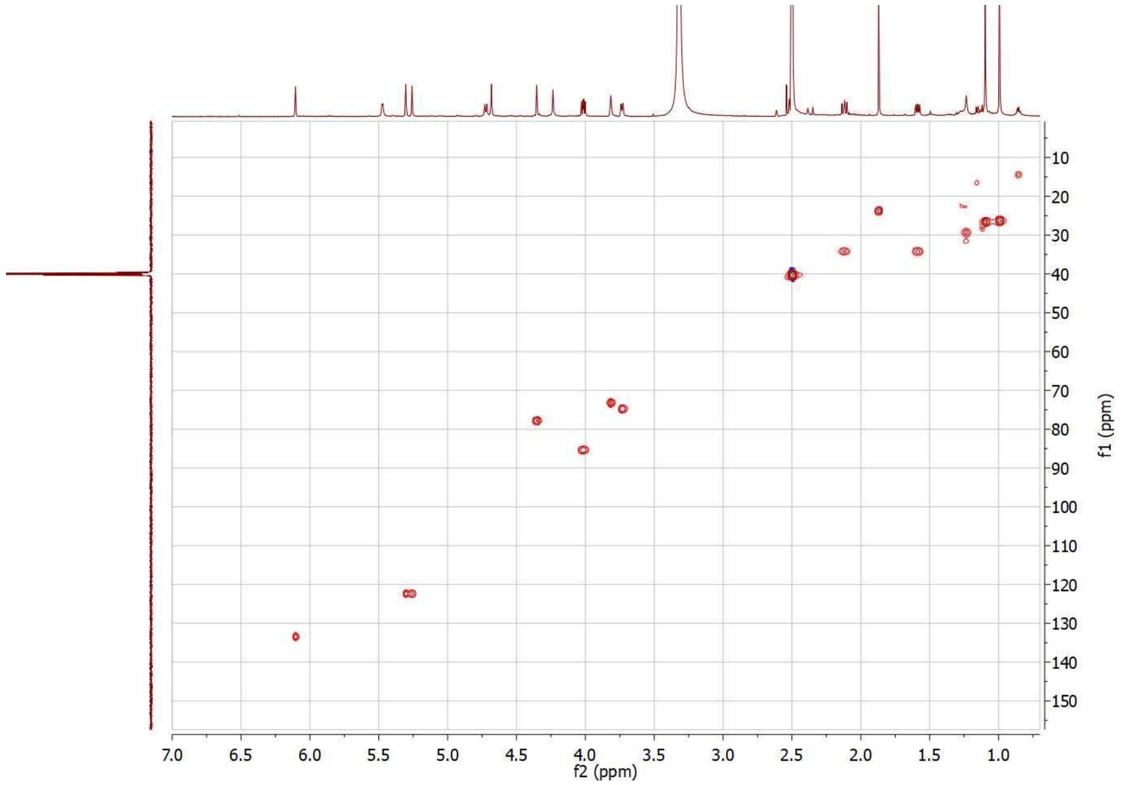


**Figure S36.** The HSQC (600 MHz, DMSO-$d_6$) spectrum of compound **5**.

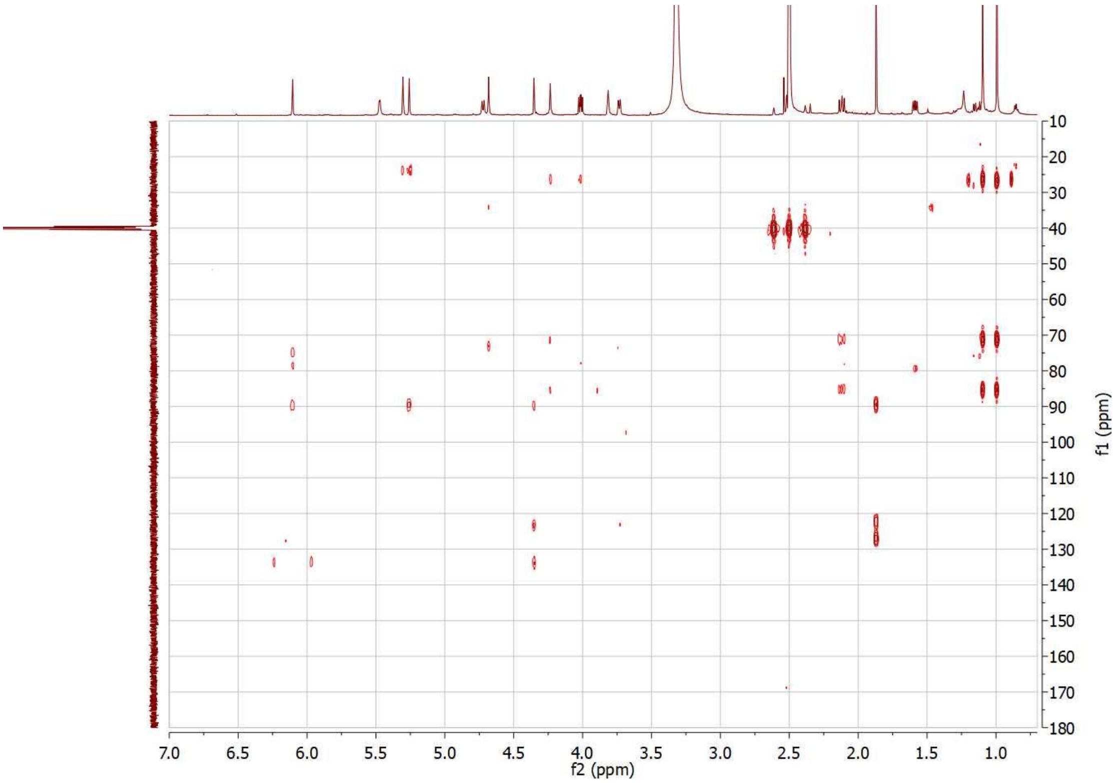


**Figure S37.** The HMBC (600MHz, DMSO-$d_6$) spectrum of compound **5**.

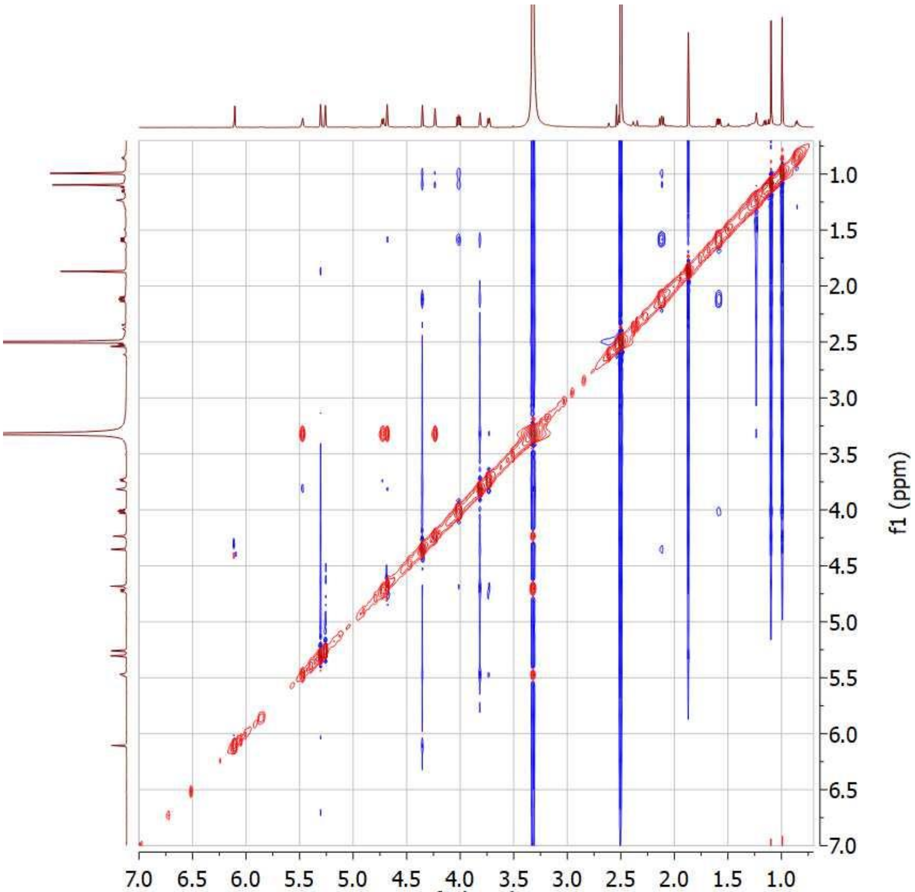


**Figure S38.** The ROESY (600 MHz, DMSO-$d_6$) spectrum of compound **5**.

**Acquisition Parameter**

| | | | | | |
|---|---|---|---|---|---|
| Source Type | ESI | Ion Polarity | Positive | Set Nebulizer | 0.3 Bar |
| Focus | Not active | Set Capillary | 4000 V | Set Dry Heater | 180 °C |
| Scan Begin | 50 m/z | Set End Plate Offset | -500 V | Set Dry Gas | 4.0 l/min |
| Scan End | 1500 m/z | Set Collision Cell RF | 600.0 Vpp | Set Divert Valve | Source |

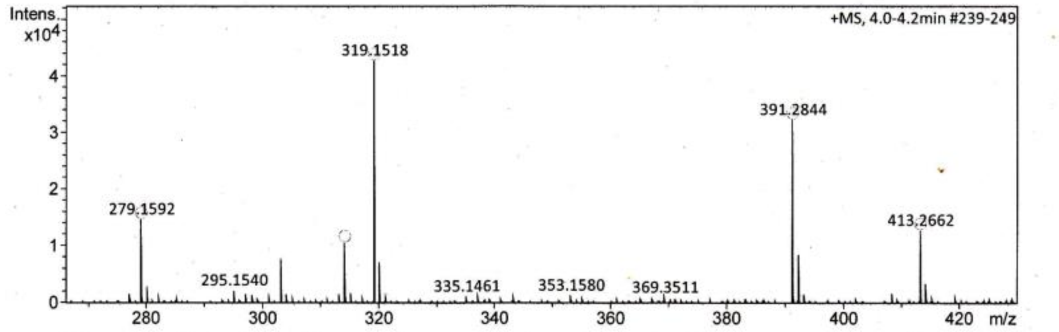


| Meas. m/z | # | Ion Formula | m/z | err [ppm] | mSigma | # mSigma | Score | rdb | e⁻ Conf | N-Rule |
|---|---|---|---|---|---|---|---|---|---|---|
| 279.1592 | 1 | C17H19N4 | 279.1604 | 4.4 | 9.7 | 1 | 57.04 | 10.5 | even | ok |
| | 2 | C16H23O4 | 279.1591 | -0.4 | 10.4 | 2 | 100.00 | 5.5 | even | ok |
| | 3 | C15H20N4Na | 279.1580 | -4.2 | 12.9 | 3 | 55.47 | 7.5 | even | ok |
| 314.1963 | 1 | C16H28NO5 | 314.1962 | -0.5 | 8.9 | 1 | 100.00 | 3.5 | even | ok |
| | 2 | C15H25N5NaO | 314.1951 | -3.9 | 10.9 | 2 | 55.67 | 5.5 | even | ok |
| | 3 | C17H24N5O | 314.1975 | 3.8 | 22.6 | 3 | 44.40 | 8.5 | even | ok |
| 319.1518 | 1 | C14H19N6O3 | 319.1513 | -1.4 | 6.7 | 1 | 90.01 | 8.5 | even | ok |
| | 2 | C13H23N2O7 | 319.1500 | -5.6 | 7.5 | 2 | 40.26 | 3.5 | even | ok |
| | 3 | C16H24NaO5 | 319.1516 | -0.5 | 7.8 | 3 | 100.00 | 4.5 | even | ok |
| | 4 | C17H20N4NaO | 319.1529 | 3.7 | 20.8 | 4 | 45.83 | 9.5 | even | ok |

**Figure S39.** The HRESIMS of compound **6**.

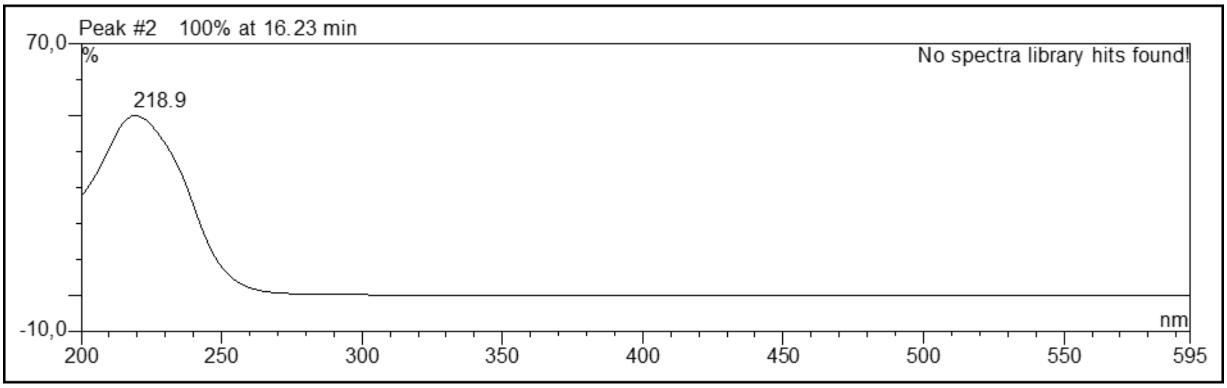


**Figure S40.** The UV spectrum of compound **6**.

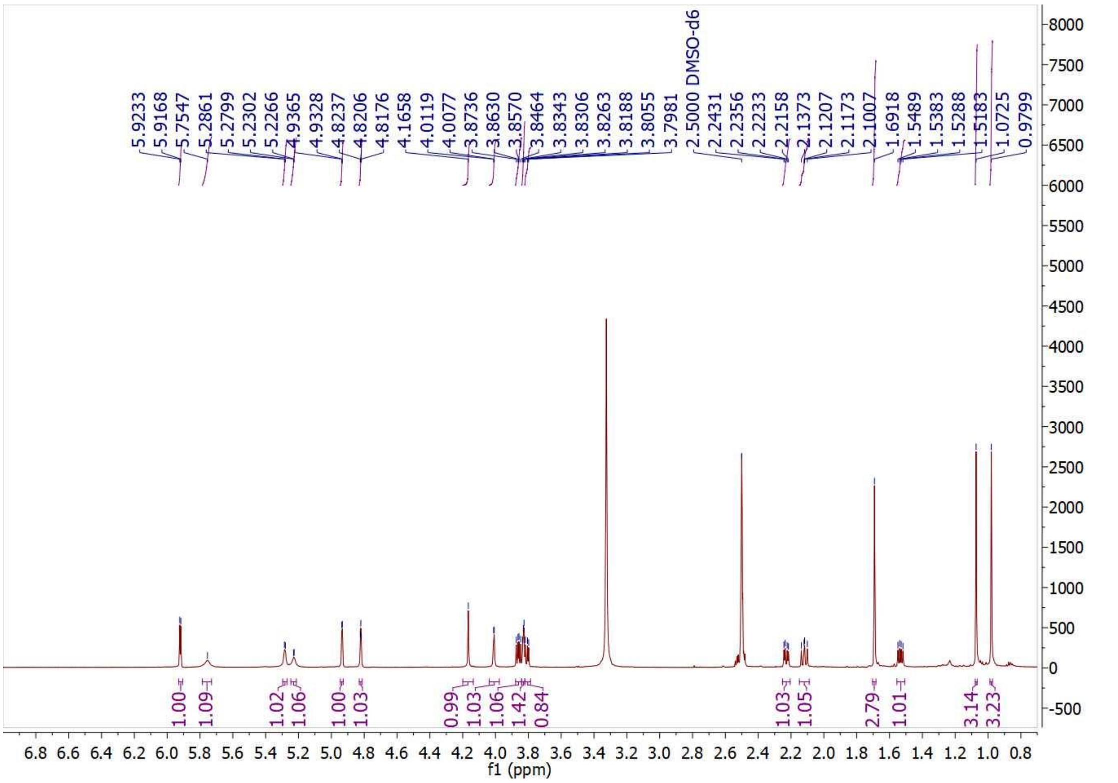

**Figure S41.** The $^{1}$H-NMR (600 MHz, DMSO-$d_6$) spectrum of compound **6**.

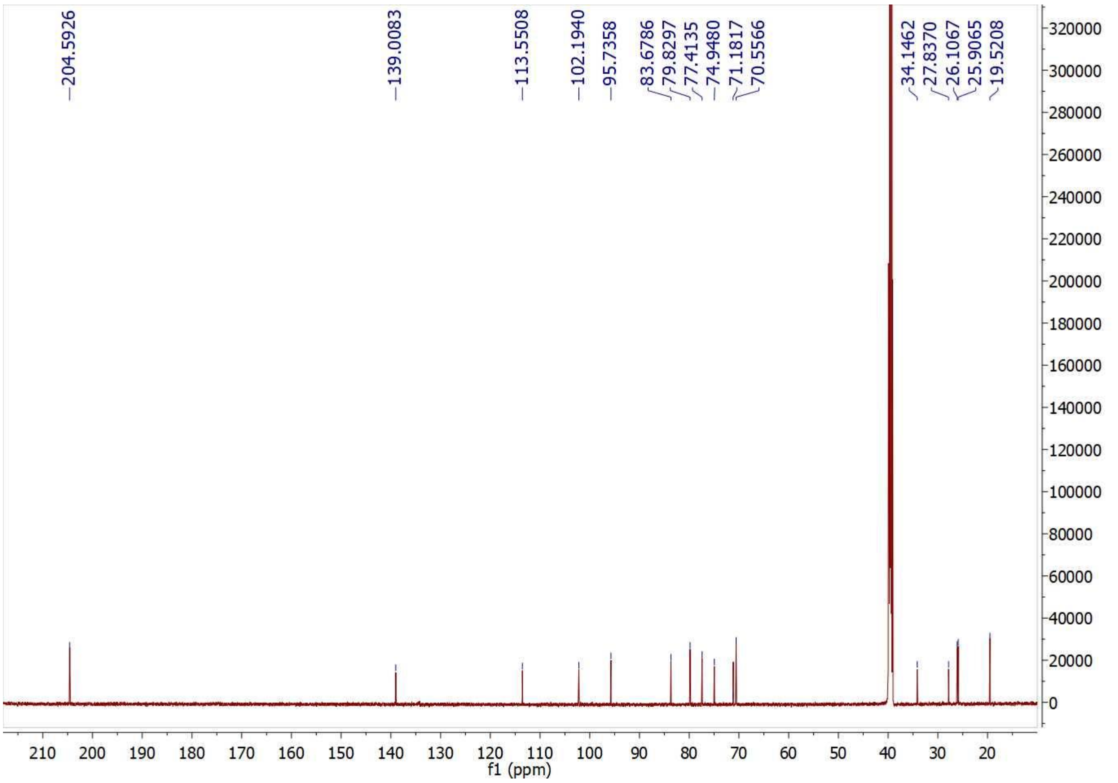

**Figure S42.** The $^{13}$C-NMR (150 MHz, DMSO-$d_6$) spectrum of compound **6**.

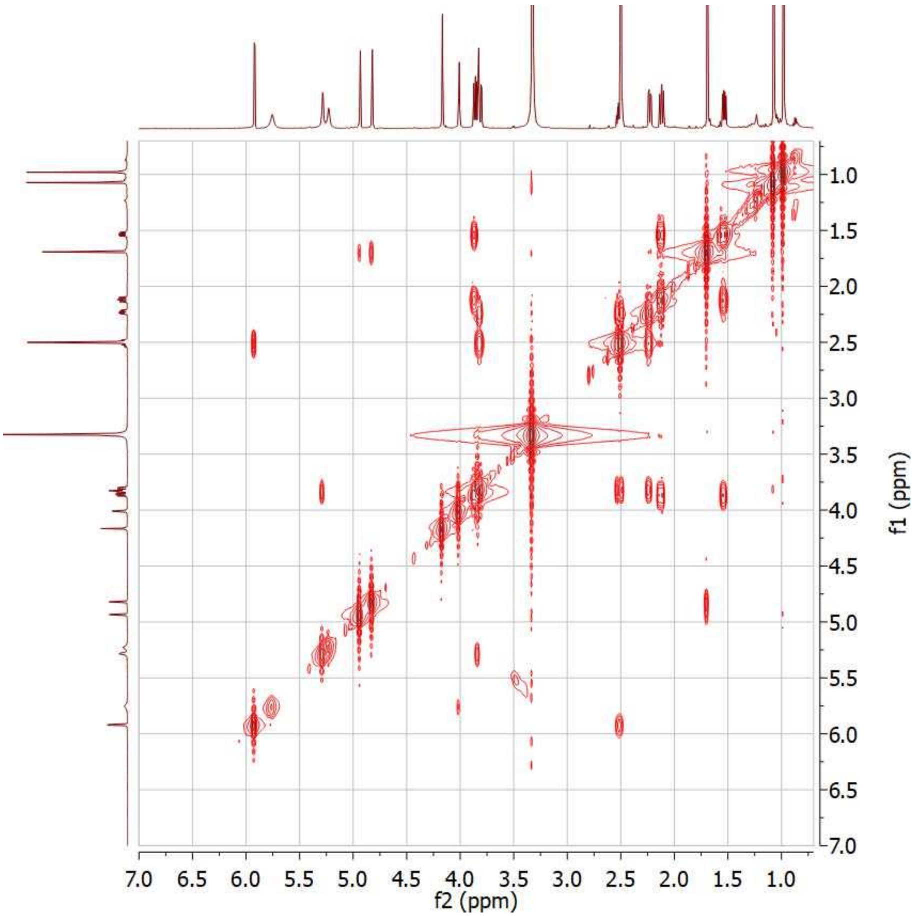


**Figure S43.** The $^{1}$H-$^{1}$H COSY (600 MHz, DMSO-$d_6$) spectrum of compound **6**.

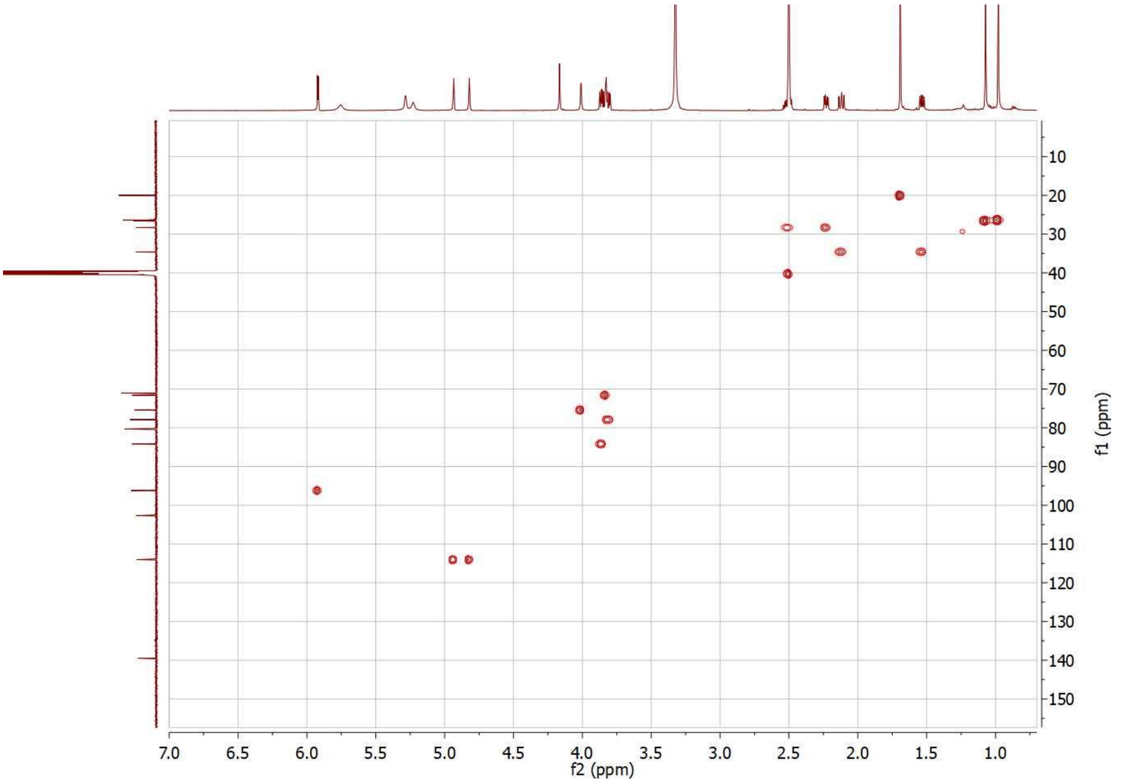


**Figure S44.** The HSQC (600 MHz, DMSO-$d_6$) spectrum of compound **6**.

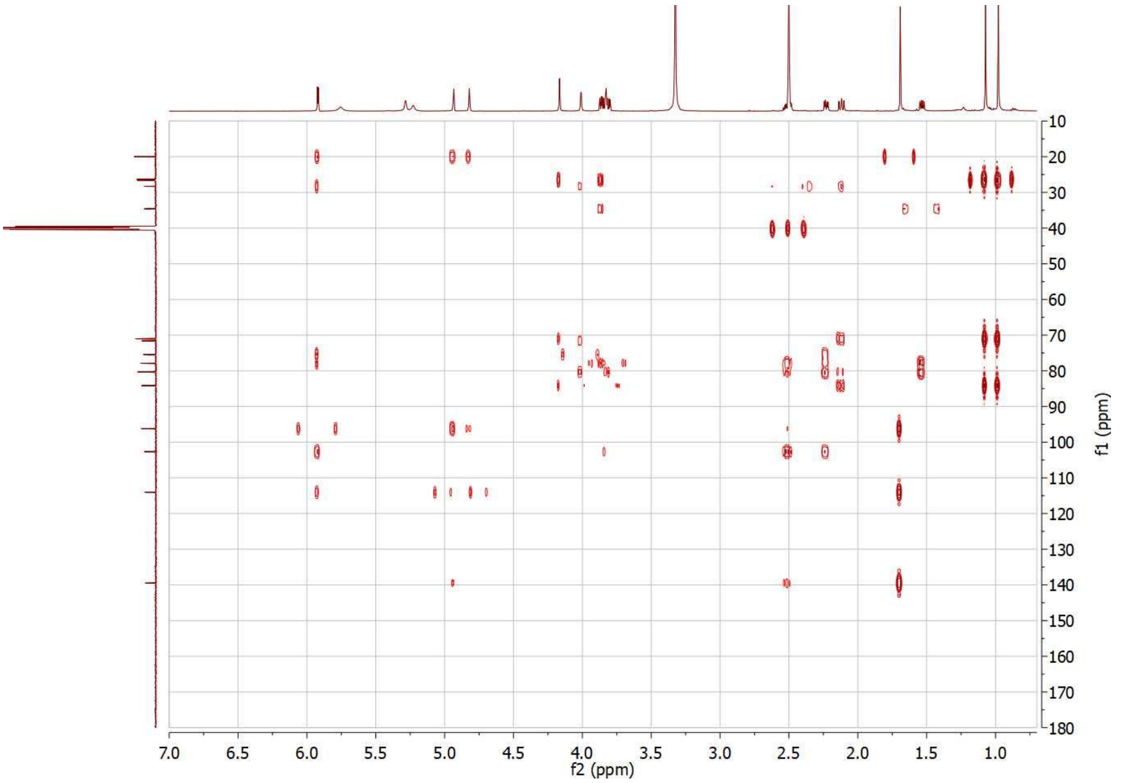


**Figure S45.** The HMBC (600 MHz, DMSO-$d_6$) spectrum of compound **6**.

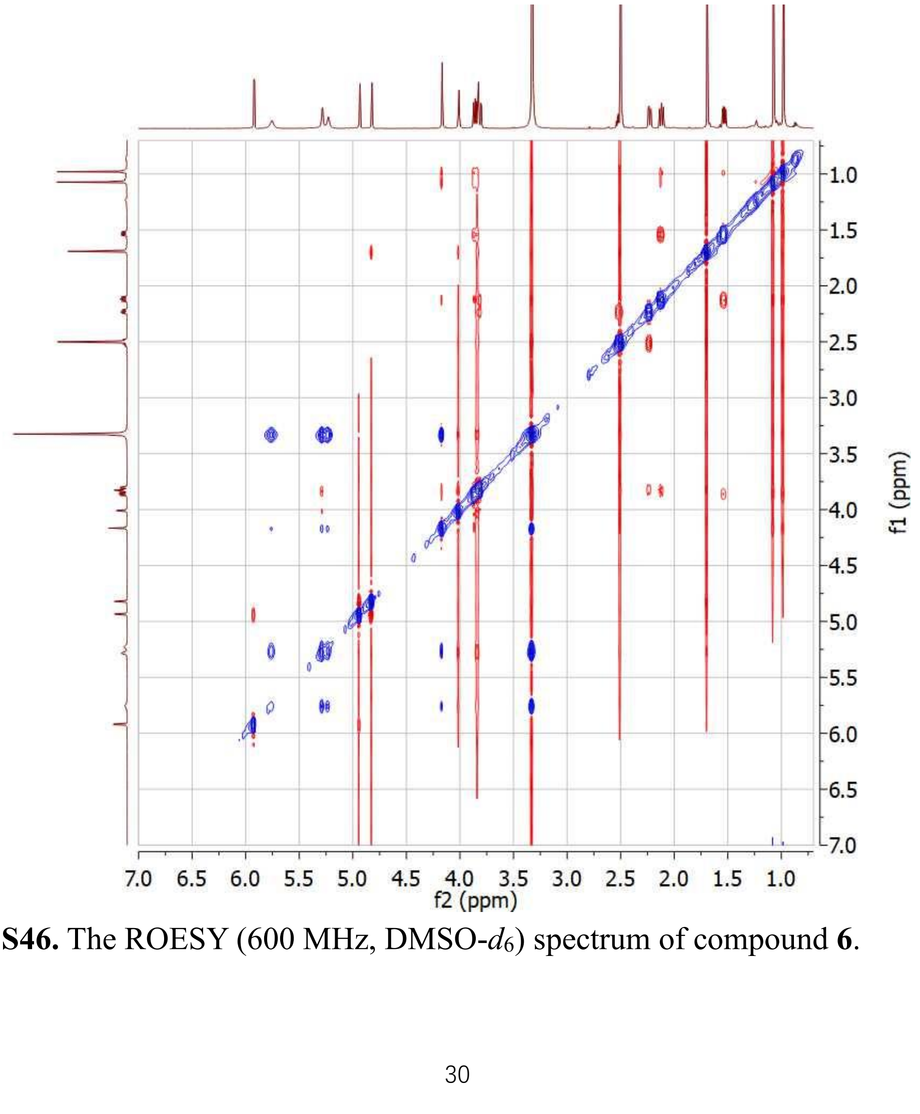


**Figure S46.** The ROESY (600 MHz, DMSO-$d_6$) spectrum of compound **6**.

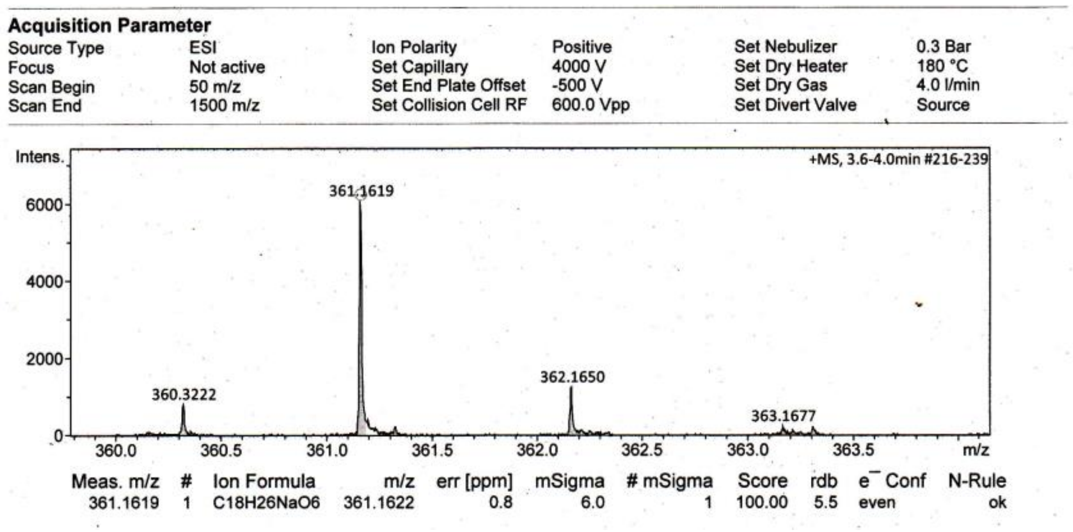


**Figure S47.** The HRESIMS of compound **7**.

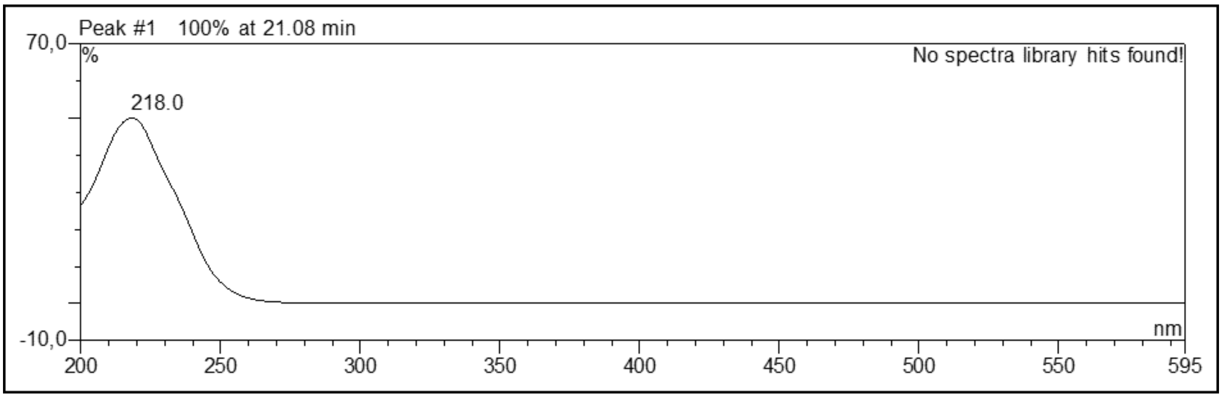


**Figure S48.** The UV spectrum of compound **7**.

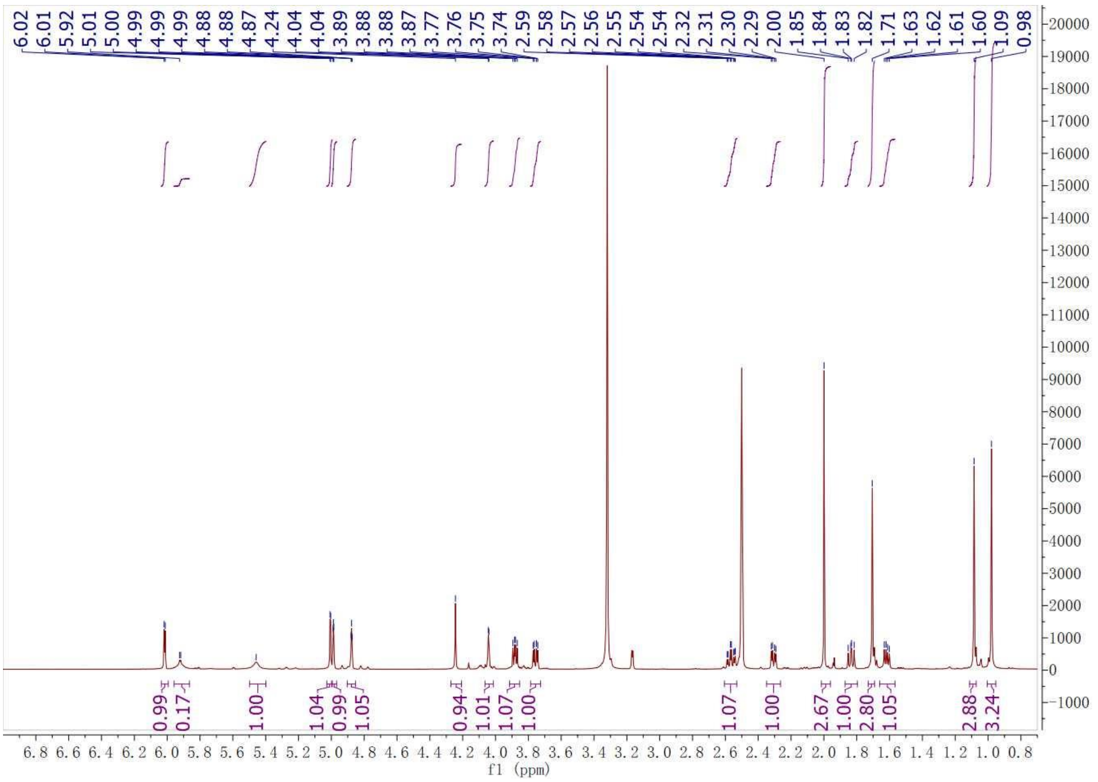

**Figure S49.** The $^{1}$H-NMR (600 MHz, DMSO-$d_6$) spectrum of compound **7**.

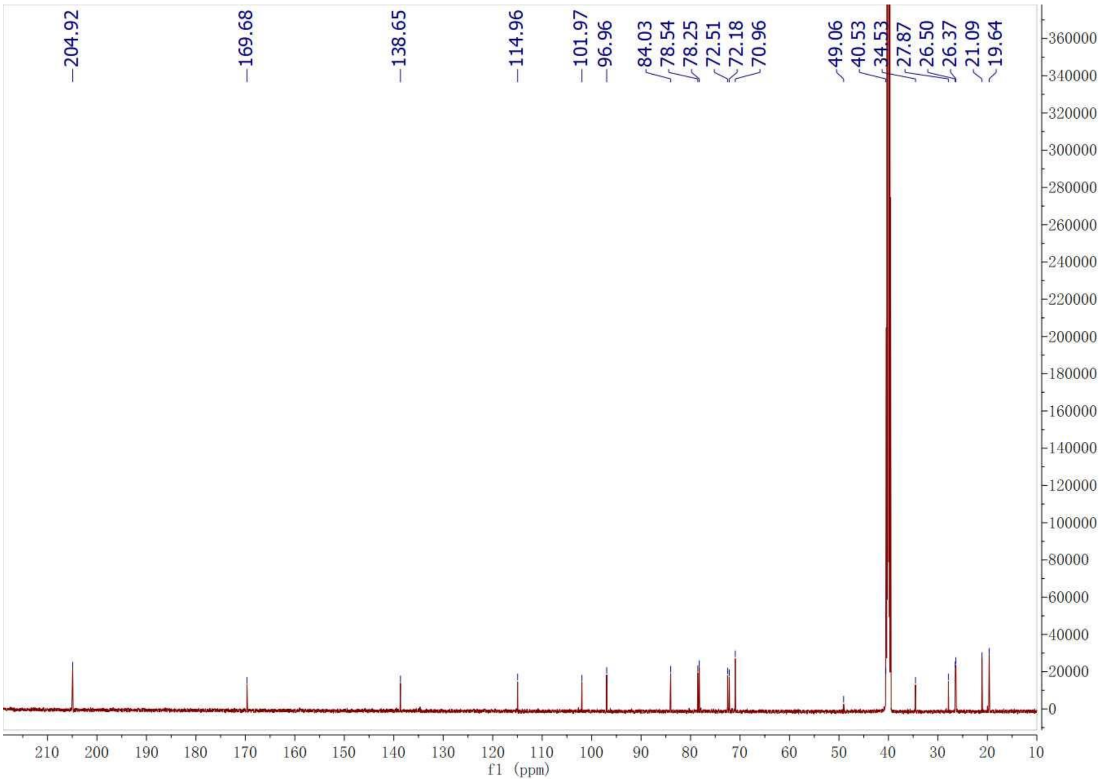

**Figure S50.** The $^{13}$C-NMR (150 MHz, DMSO-$d_6$) spectrum of compound **7**.

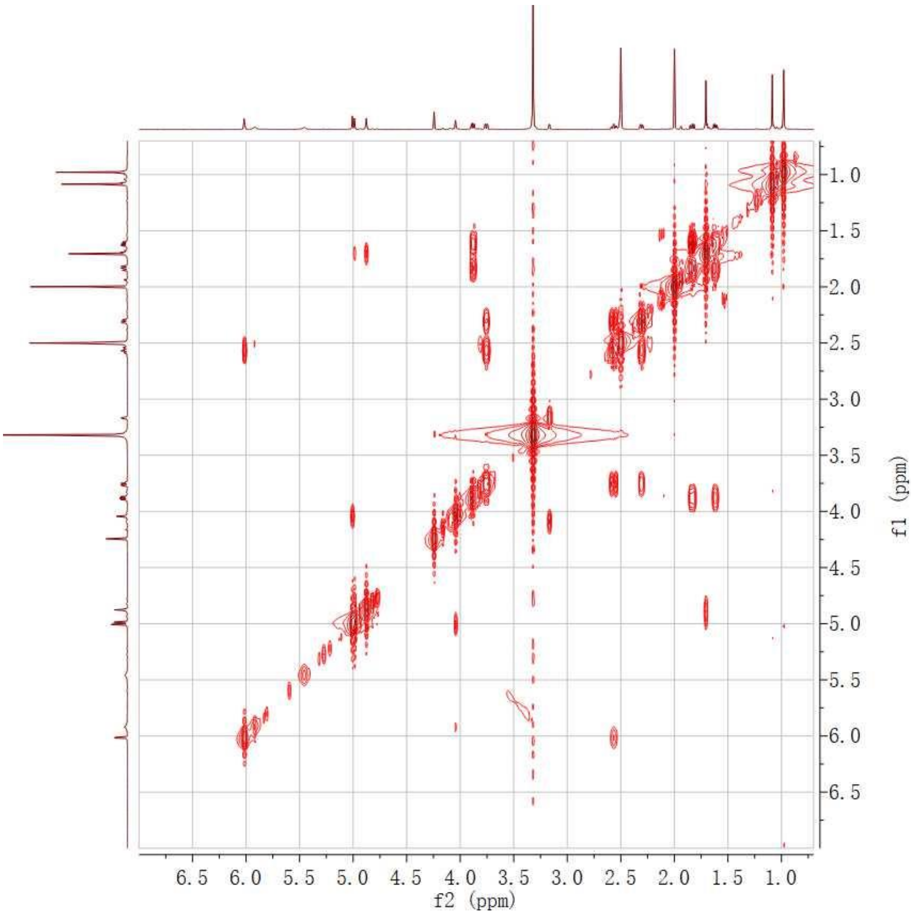


**Figure S51.** The $^{1}$H-$^{1}$H COSY (600 MHz, DMSO-$d_6$) spectrum of compound **7**.

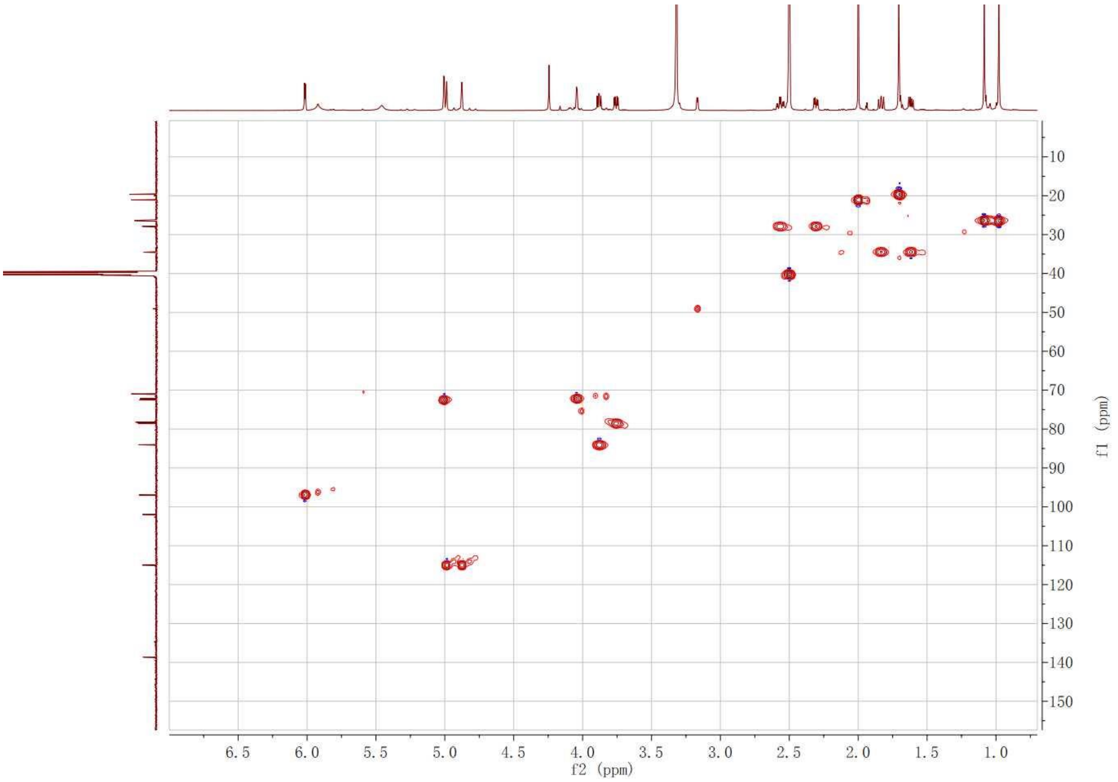


**Figure S52.** The HSQC (600 MHz, DMSO-$d_6$) spectrum of compound **7**.

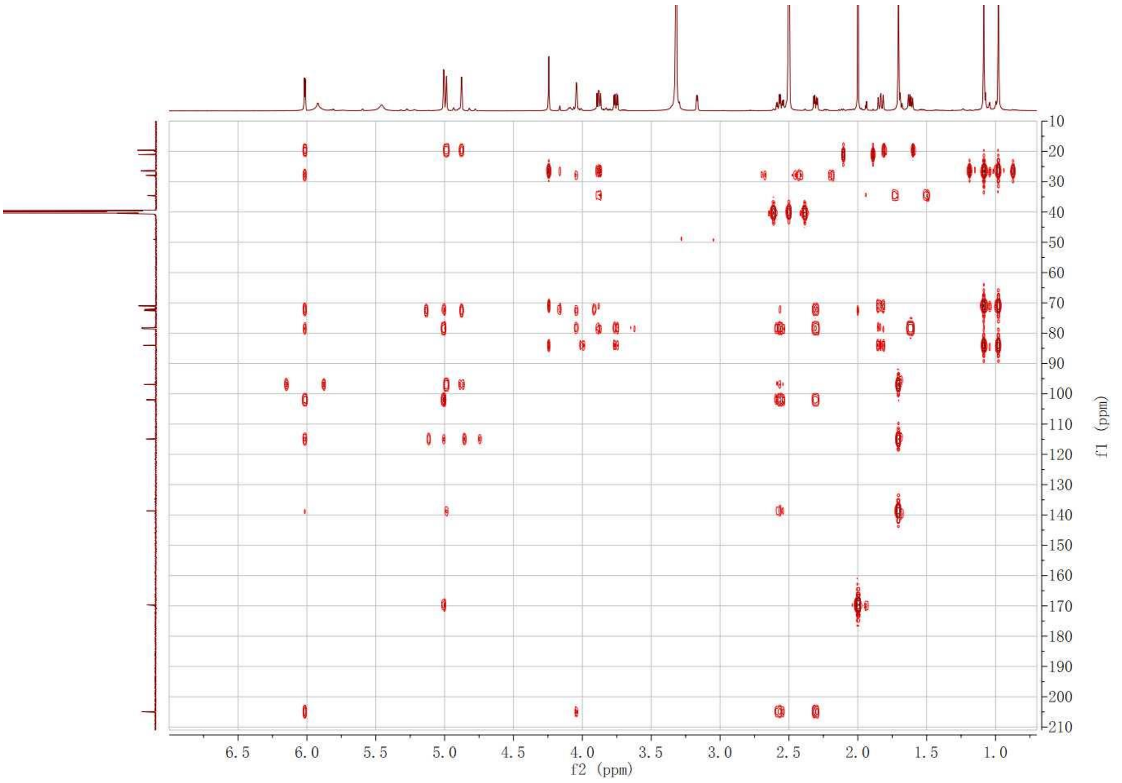


**Figure S53.** The HMBC (600 MHz, DMSO-$d_6$) spectrum of compound **7**.

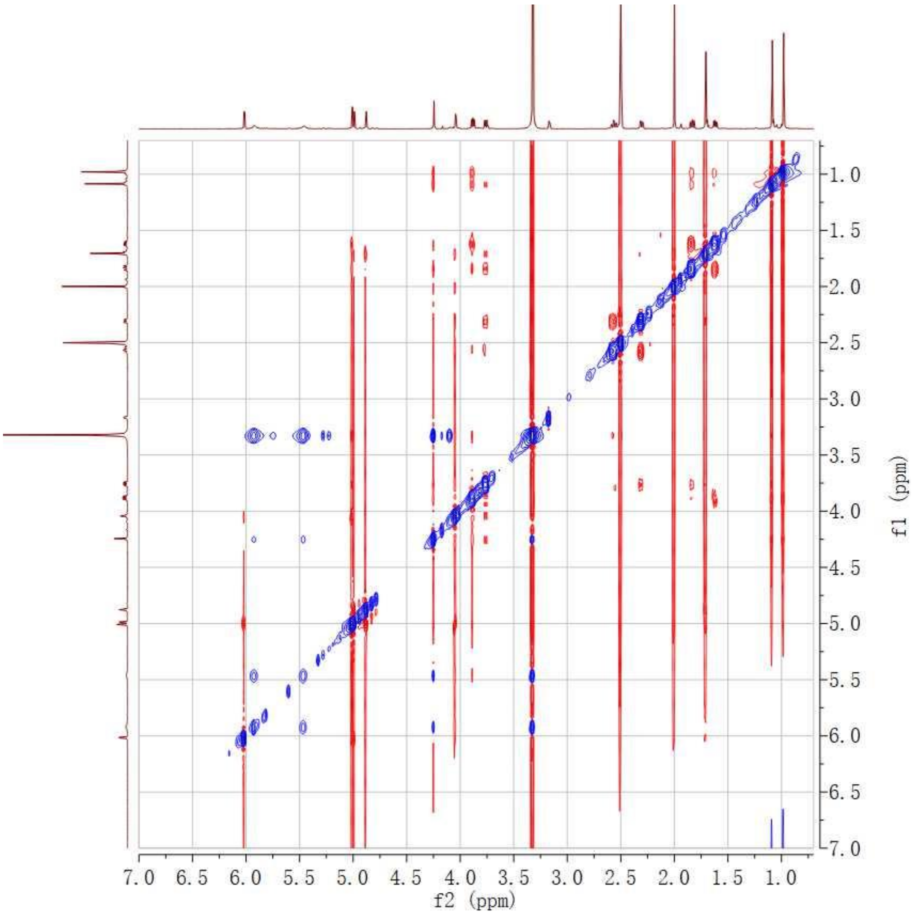


**Figure S54.** The ROESY (600 MHz, DMSO-$d_6$) spectrum of compound **7**.

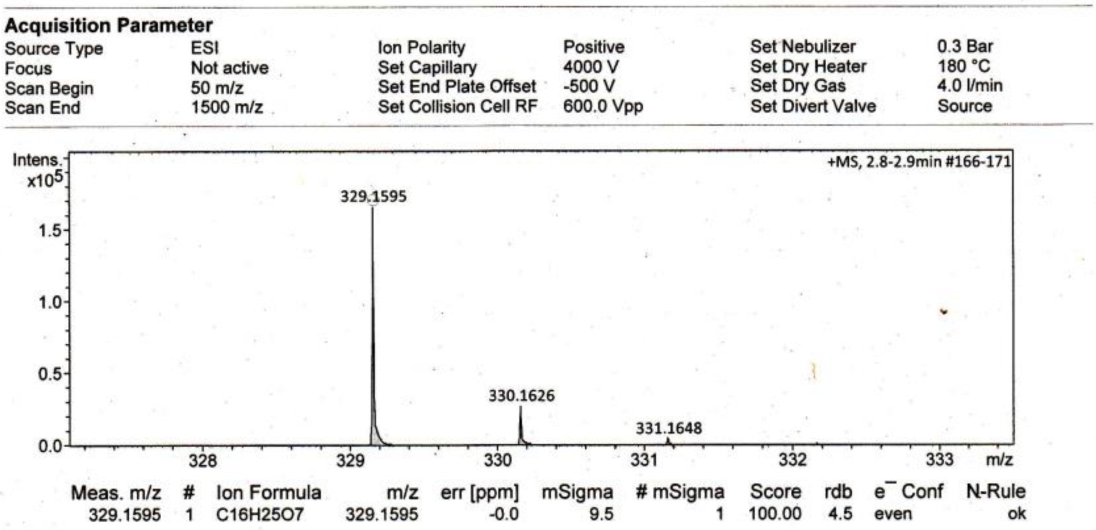


**Figure S55.** The HREISMS of compound **8**.

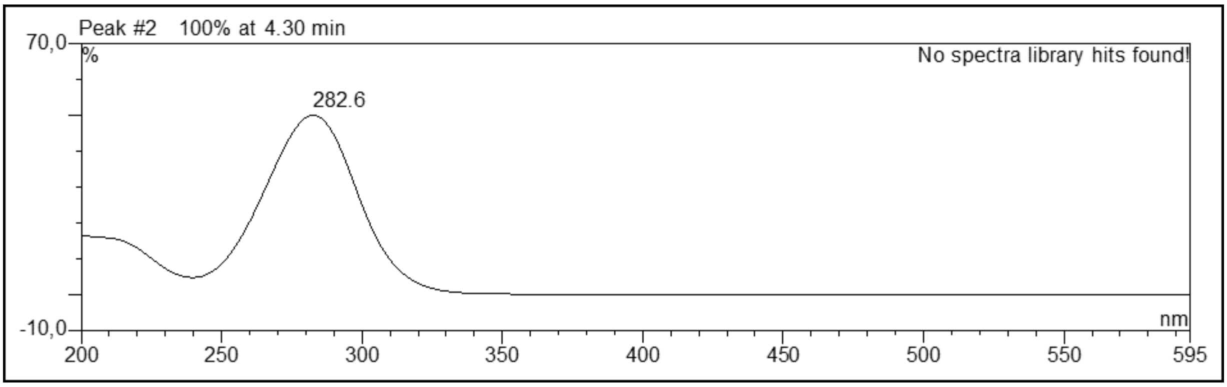


**Figure S56.** The UV spectrum of compound **8**.

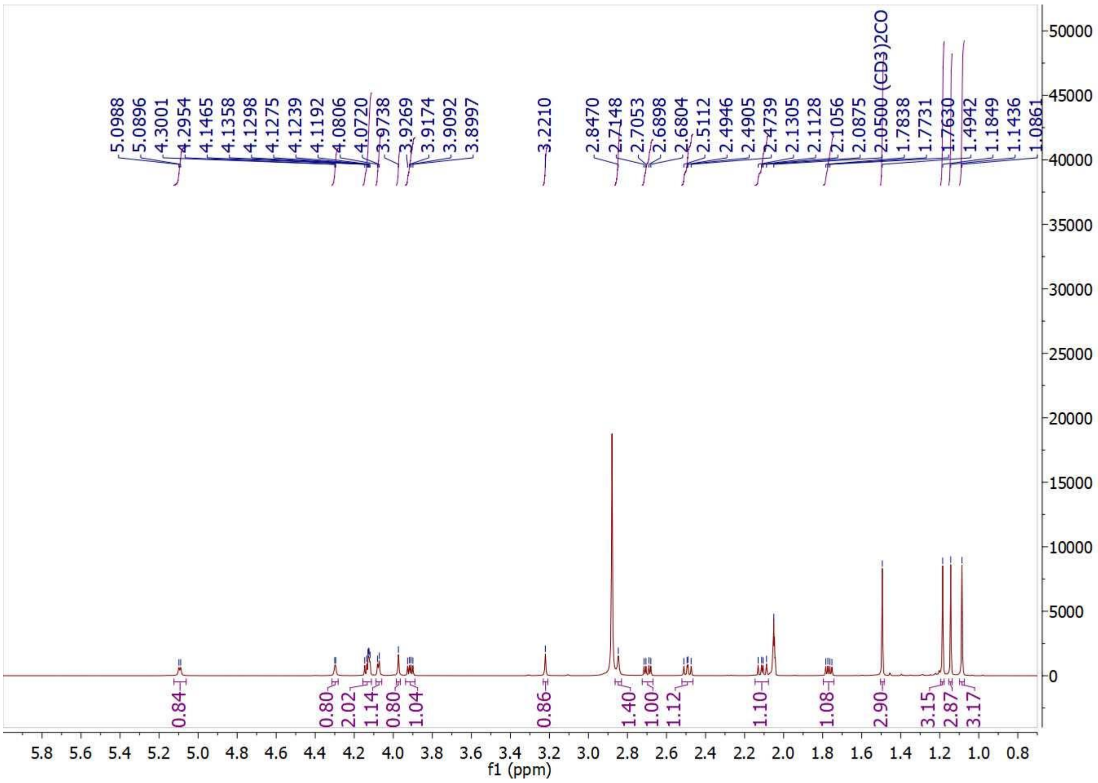

**Figure S57.** The $^{1}H$-NMR (600 MHz, Acetone-$d_6$) spectrum of compound **8**.

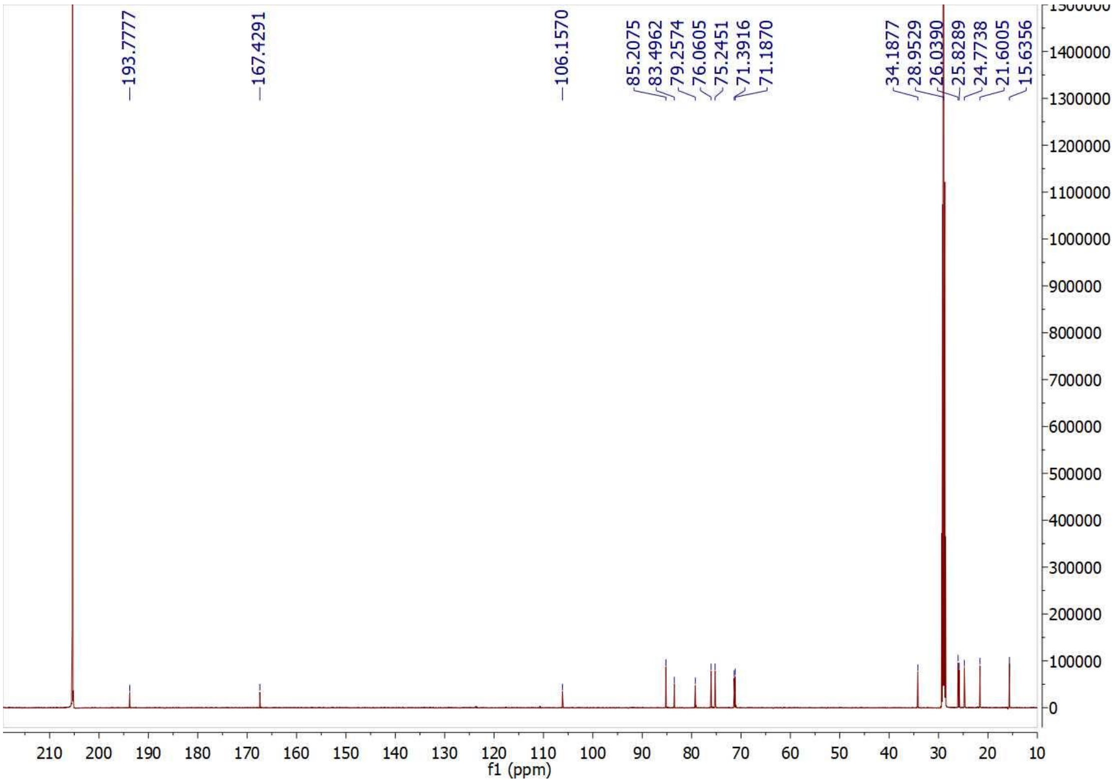

**Figure S58.** The $^{13}C$-NMR (150 MHz, Acetone-$d_6$) spectrum of compound **8**.

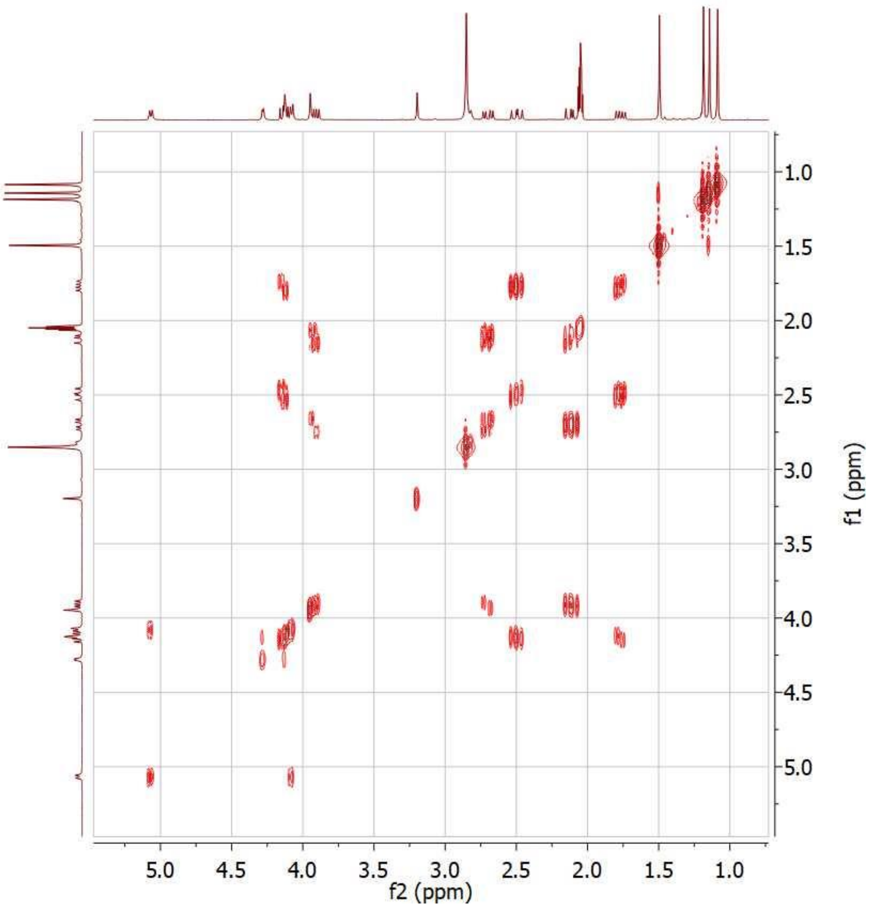


**Figure S59.** The $^1$H-H COSY (600 MHz, Acetone-$d_6$) spectrum of compound **8**.

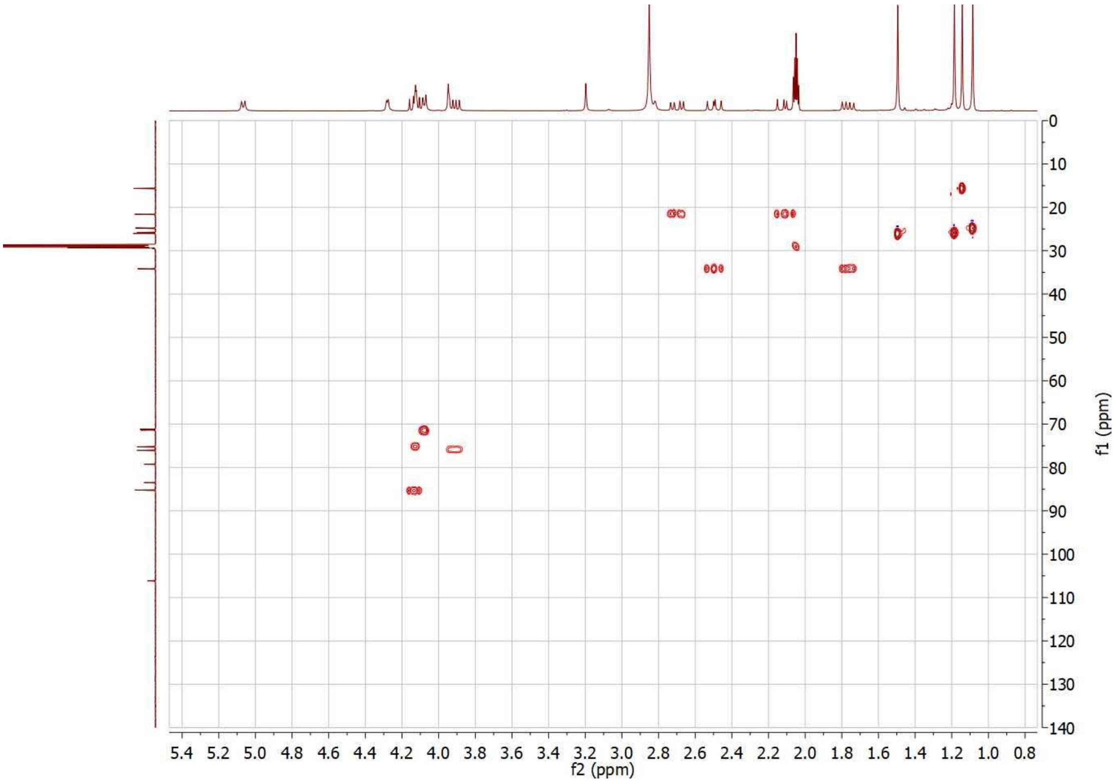


**Figure S60.** The HSQC (600 MHz, Acetone-$d_6$) spectrum of compound **8**.

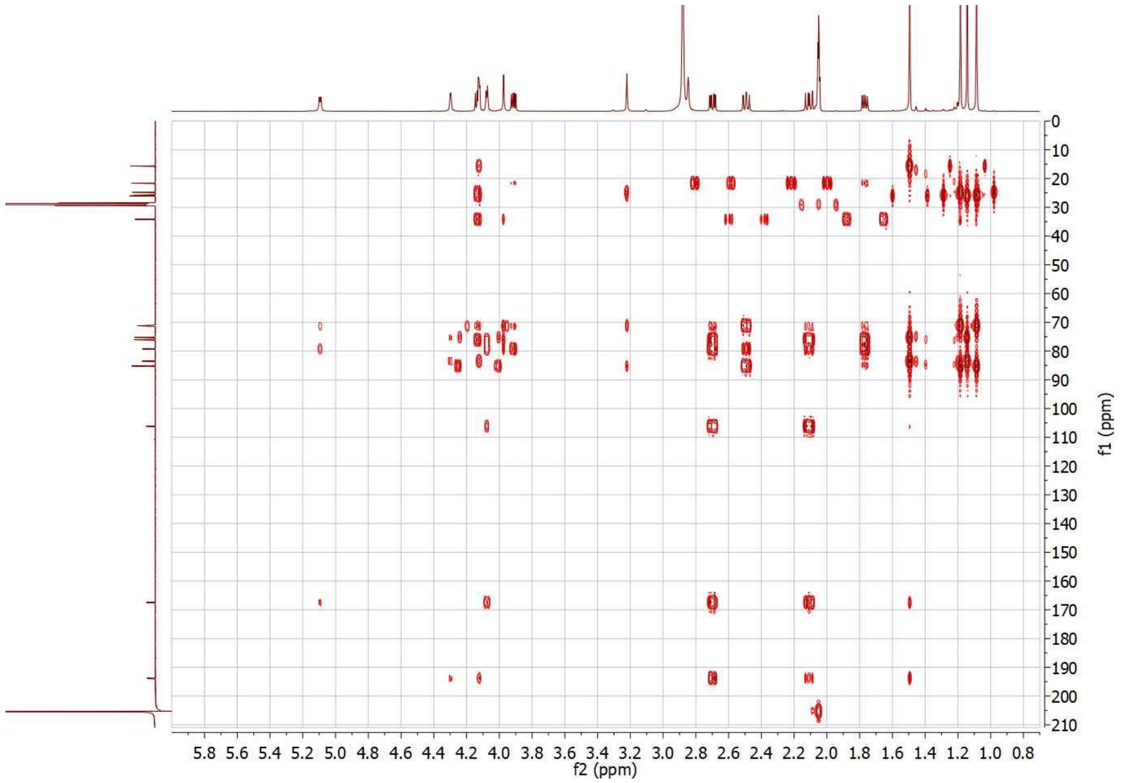


**Figure S61.** The HMBC (600 MHz, Acetone-$d_6$) spectrum of compound **8**.

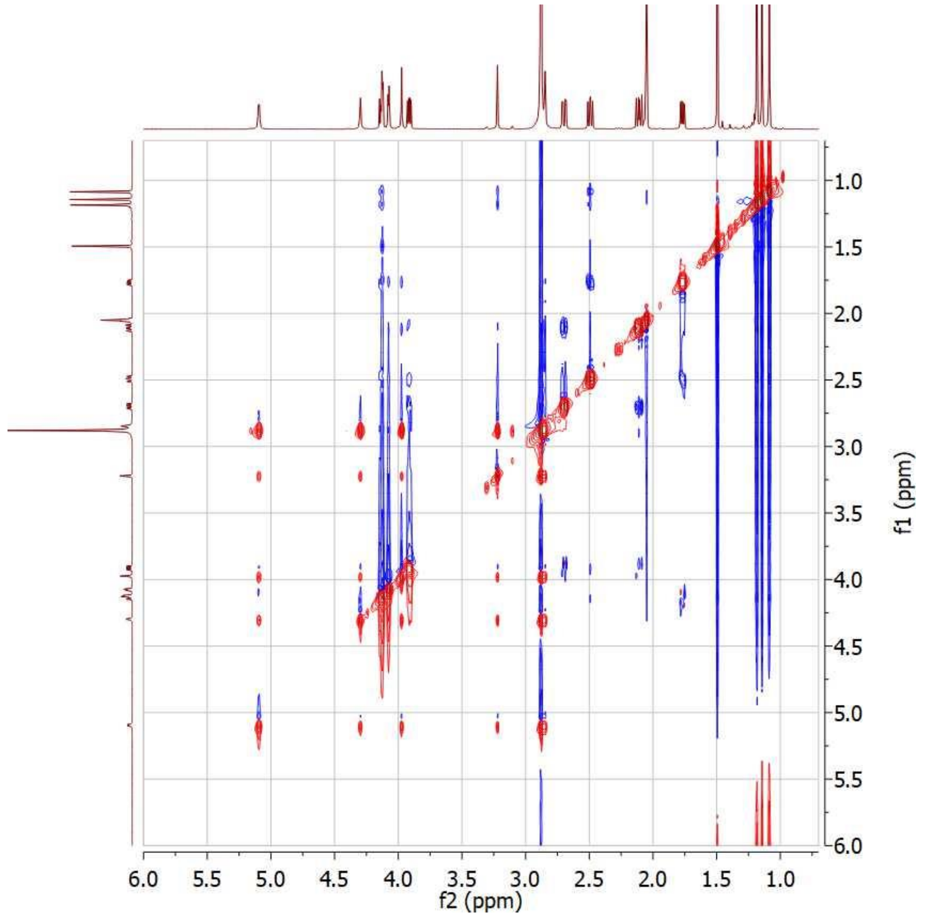


**Figure S62.** The ROESY (600 MHz, Acetone-$d_6$) spectrum of compound **8**.

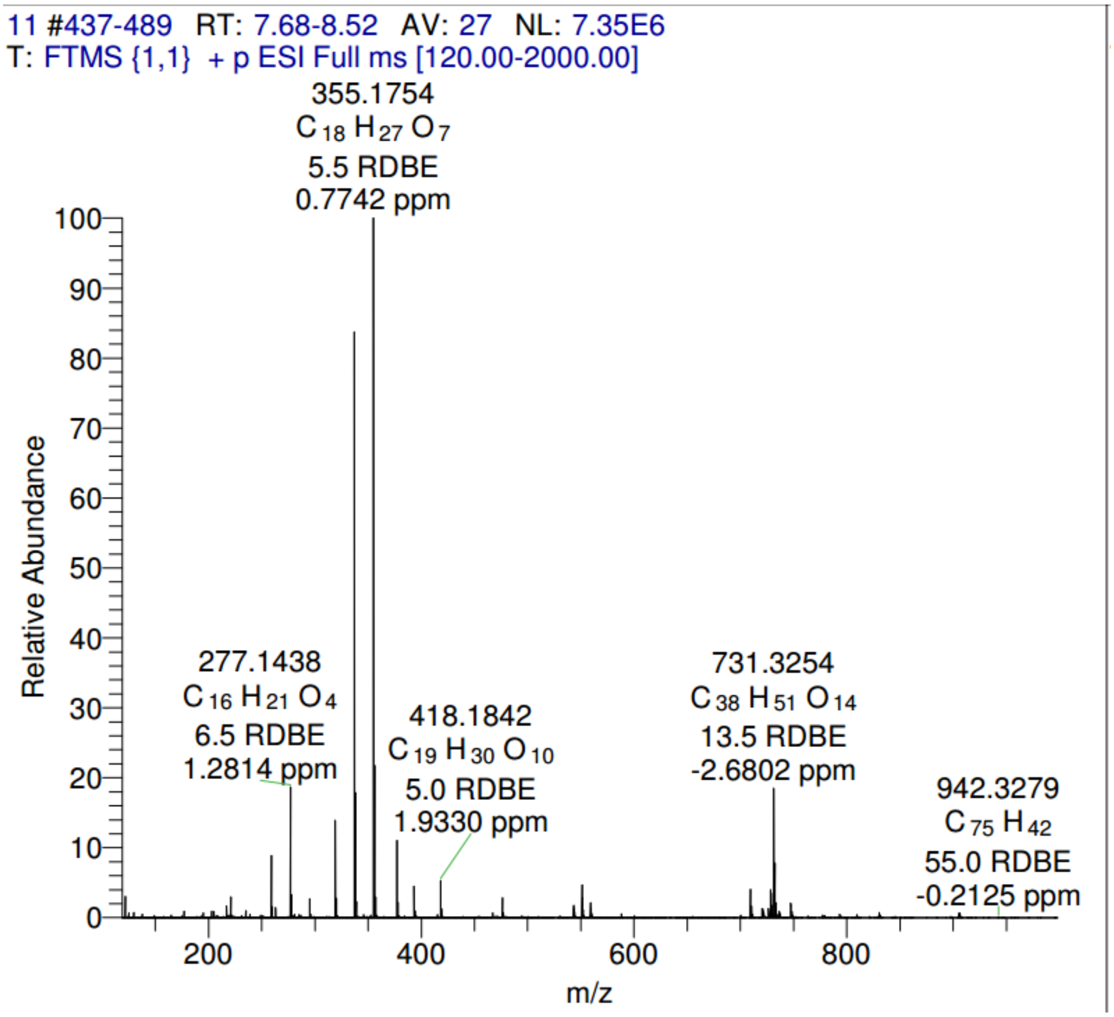


**Figure S63.** The HREISMS of compound **9**.

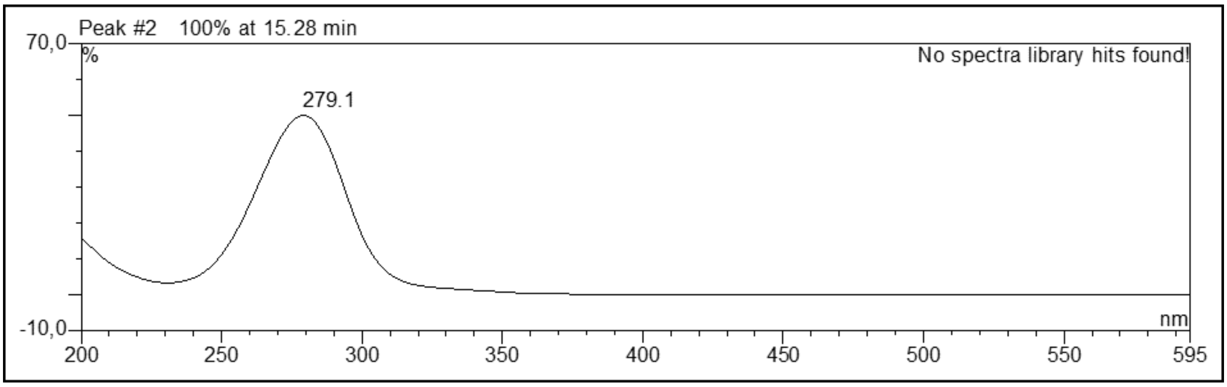


**Figure S64.** The UV spectrum of compound **9**.

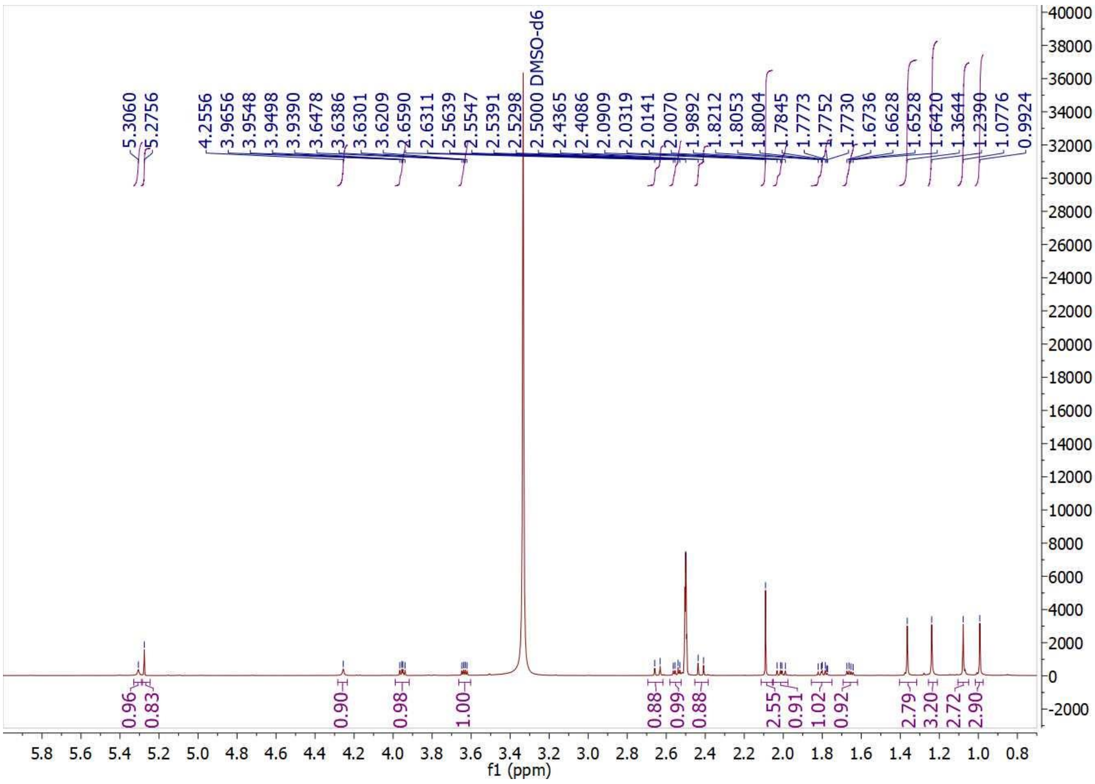


**Figure S65.** The $^{1}$H-NMR (600 MHz, DMSO-$d_6$) spectrum of compound **9**.

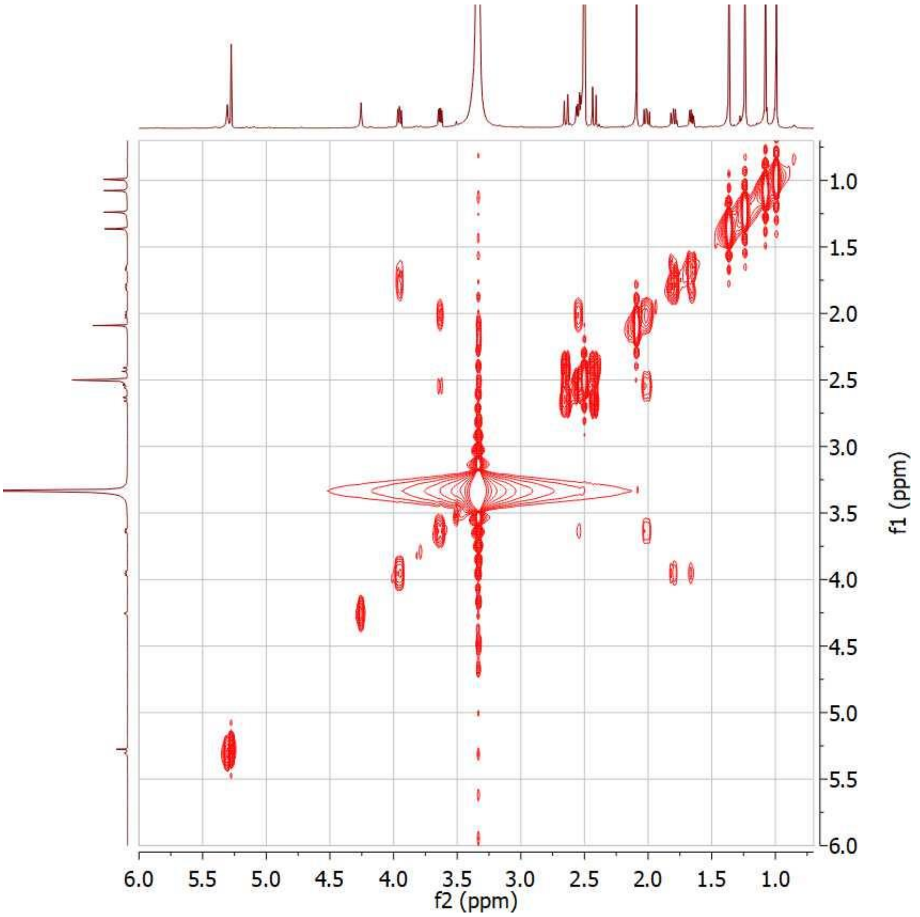


**Figure S66.** The $^{1}$H-$^{1}$H COSY (600 MHz, DMSO-$d_6$) spectrum of compound **9**.

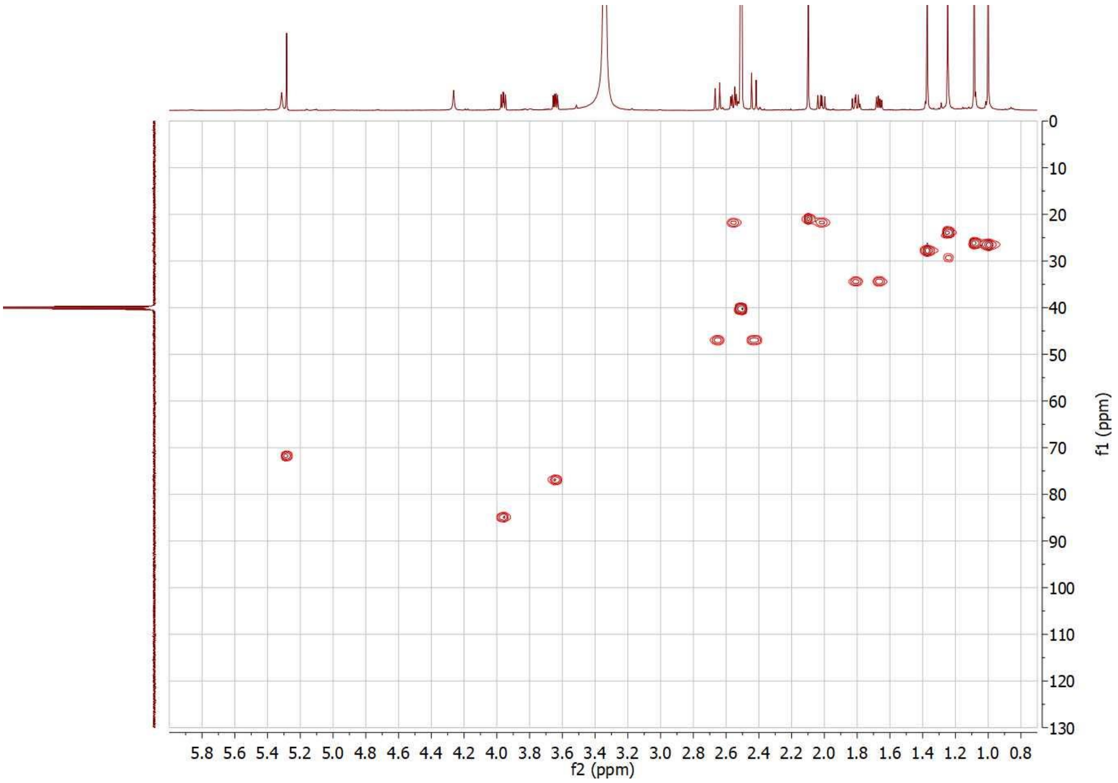


**Figure S67.** The HSQC (600 MHz, DMSO-$d_6$) spectrum of compound **9**.

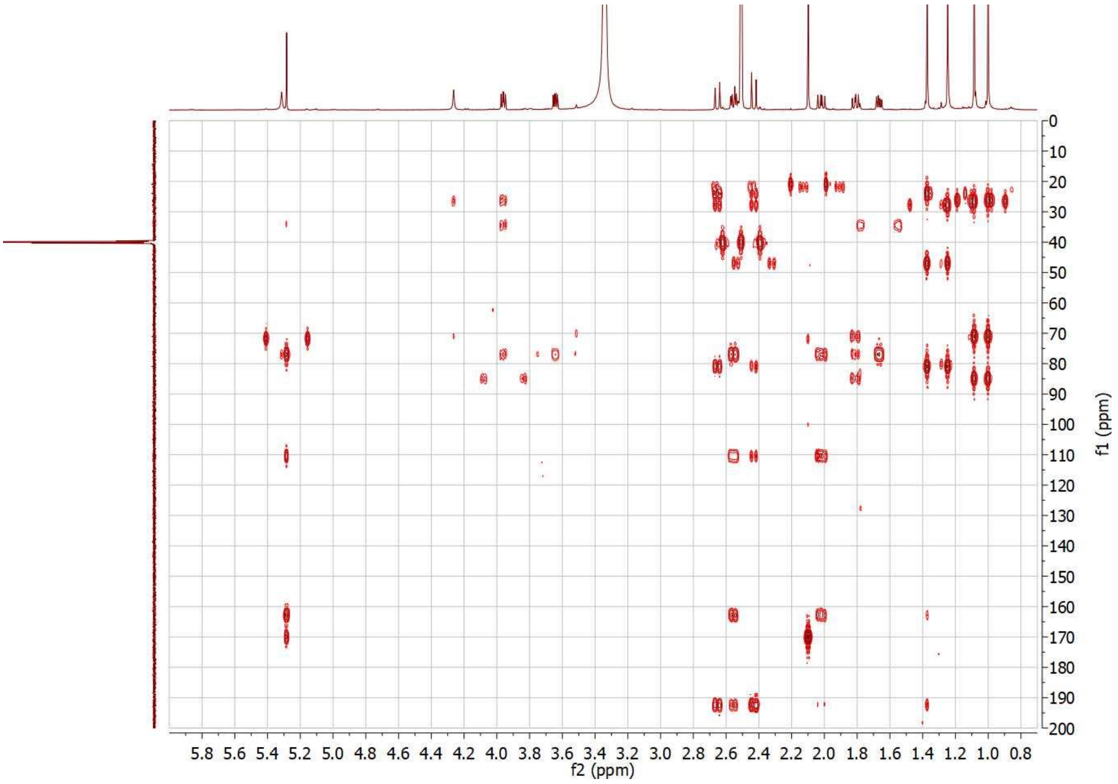


**Figure S68.** The HMBC (600 MHz, DMSO-$d_6$) spectrum of compound **9**.

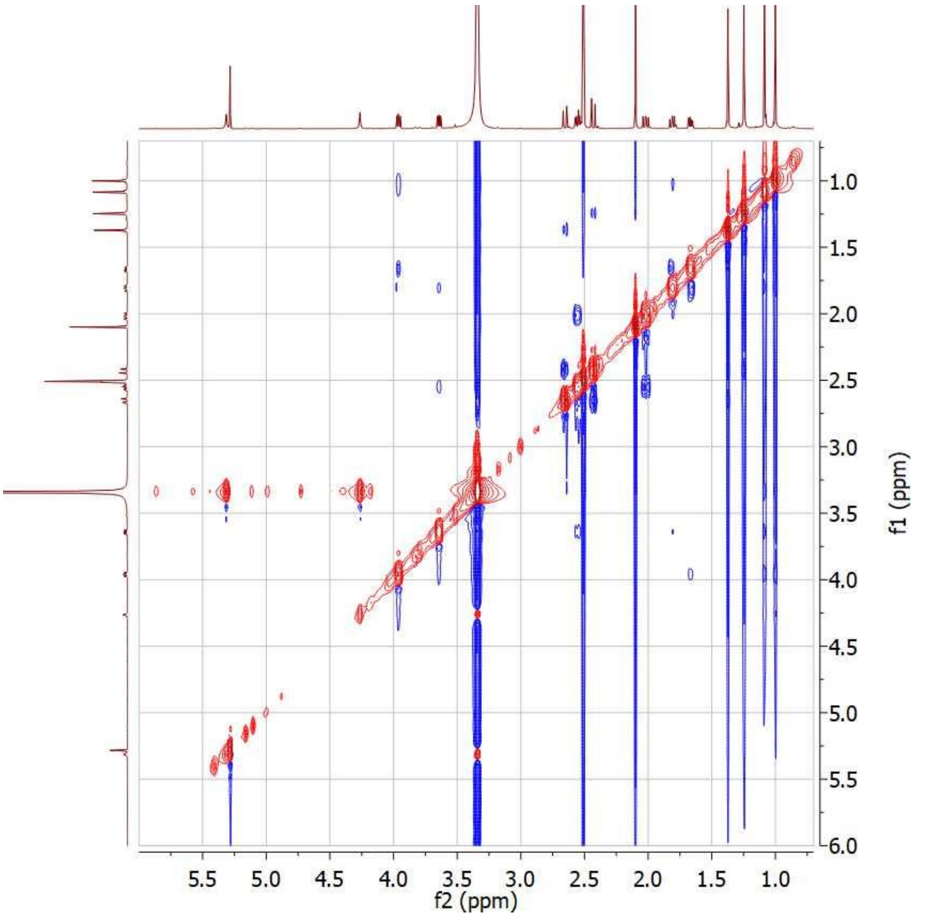

**Figure S69.** The ROESY (600 MHz, DMSO-$d_6$) spectrum of compound **9**.

**Acquisition Parameter**

| | | | | | |
|---|---|---|---|---|---|
| Source Type | ESI | Ion Polarity | Positive | Set Nebulizer | 0.3 Bar |
| Focus | Not active | Set Capillary | 4000 V | Set Dry Heater | 180 °C |
| Scan Begin | 50 m/z | Set End Plate Offset | -500 V | Set Dry Gas | 4.0 l/min |
| Scan End | 1500 m/z | Set Collision Cell RF | 600.0 Vpp | Set Divert Valve | Source |

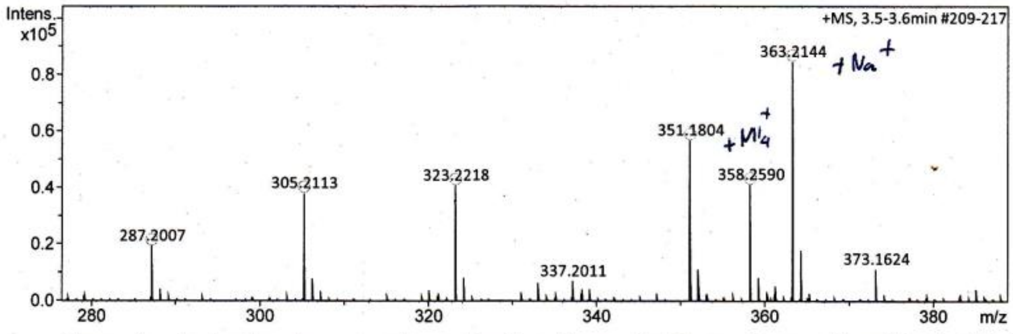


| Meas. m/z | # | Ion Formula | m/z | err [ppm] | mSigma | # mSigma | Score | rdb | e⁻ Conf | N-Rule |
|---|---|---|---|---|---|---|---|---|---|---|
| 287.2007 | 1 | C19H27O2 | 287.2006 | -0.5 | 42.2 | 1 | 100.00 | 6.5 | even | ok |
| 305.2113 | 1 | C19H29O3 | 305.2111 | -0.5 | 34.9 | 1 | 100.00 | 5.5 | even | ok |
| 323.2218 | 1 | C19H31O4 | 323.2217 | -0.4 | 3.4 | 1 | 100.00 | 4.5 | even | ok |
| 351.1804 | 1 | C19H27O6 | 351.1802 | -0.5 | 6.9 | 1 | 100.00 | 6.5 | even | ok |
| 358.2590 | 1 | C19H36NO5 | 358.2588 | -0.6 | 10.3 | 1 | 100.00 | 2.5 | even | ok |
| 363.2144 | 1 | C19H32NaO5 | 363.2142 | -0.7 | 1.0 | 1 | 100.00 | 3.5 | even | ok |

**Figure S70.** The HREISMS of compound **15**.

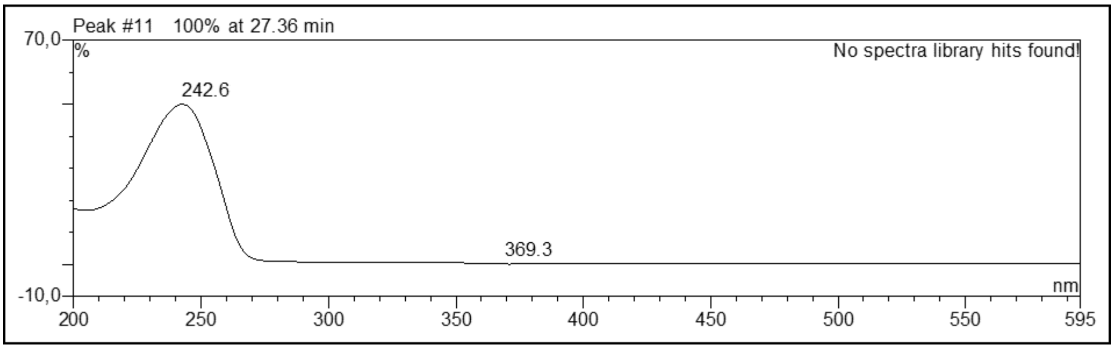


**Figure S71.** The UV spectrum of compound **15**.

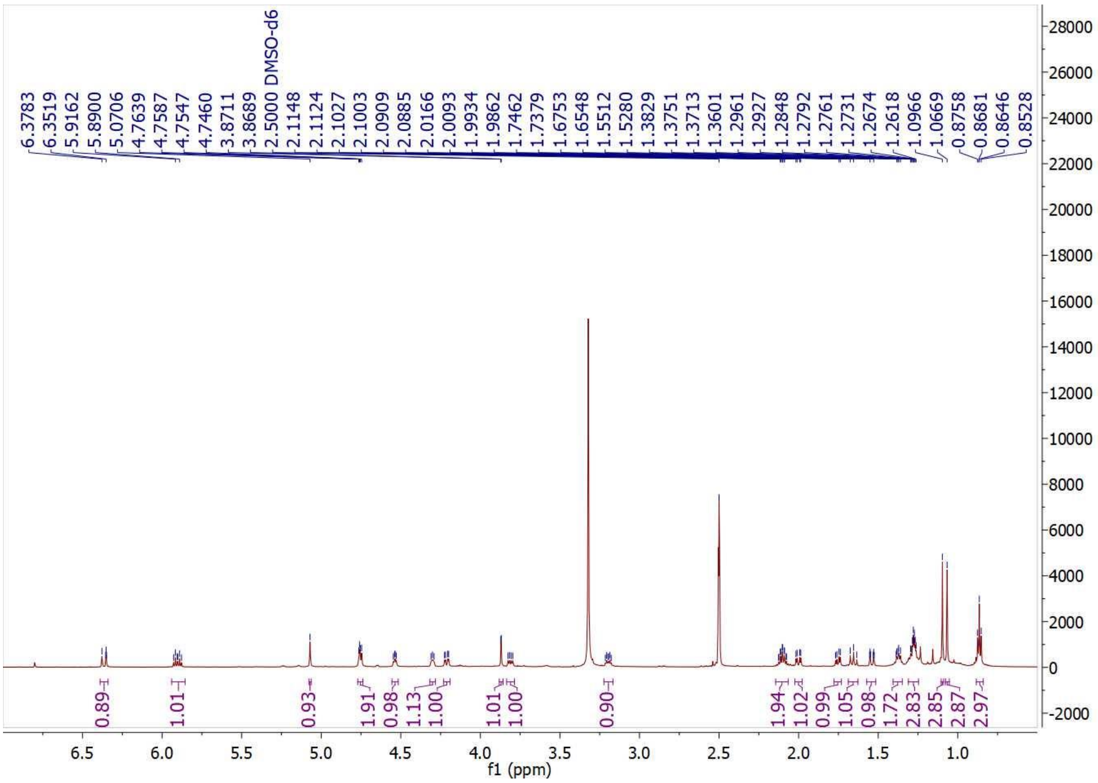

**Figure S72.** The $^{1}$H-NMR (600 MHz, DMSO-$d_6$) spectrum of compound **15**.

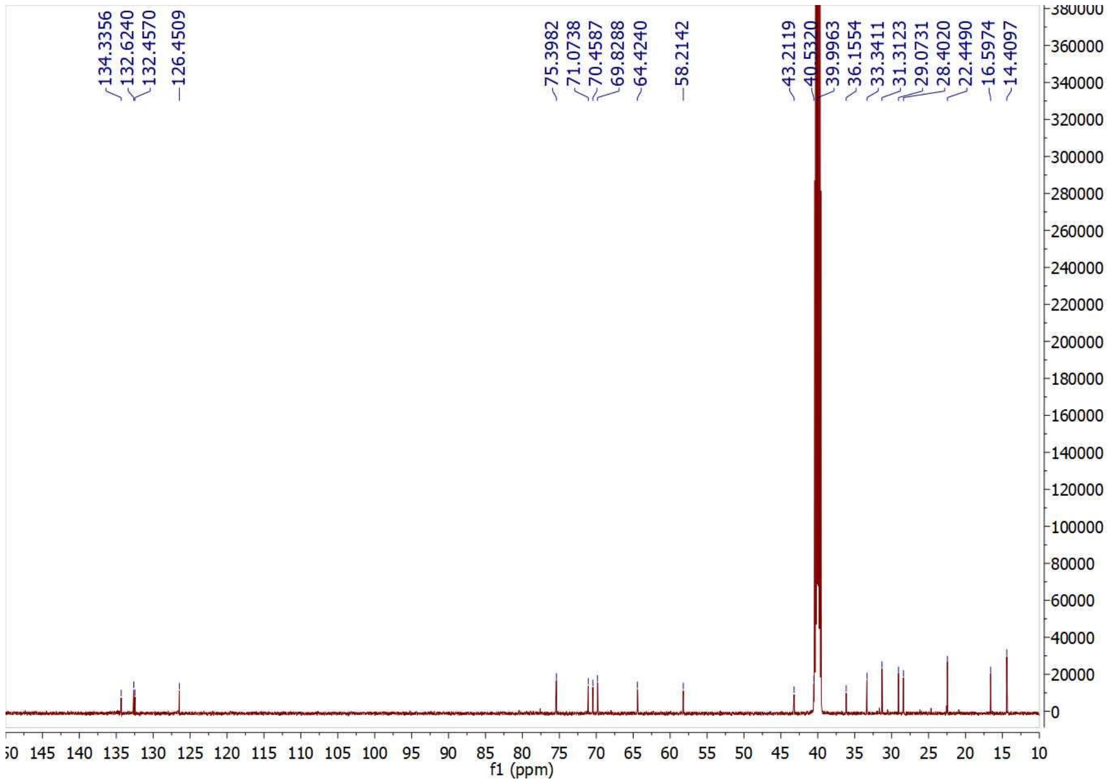


**Figure S73.** The $^{13}$C-NMR (150 MHz, DMSO-$d_6$) spectrum of compound **15**.

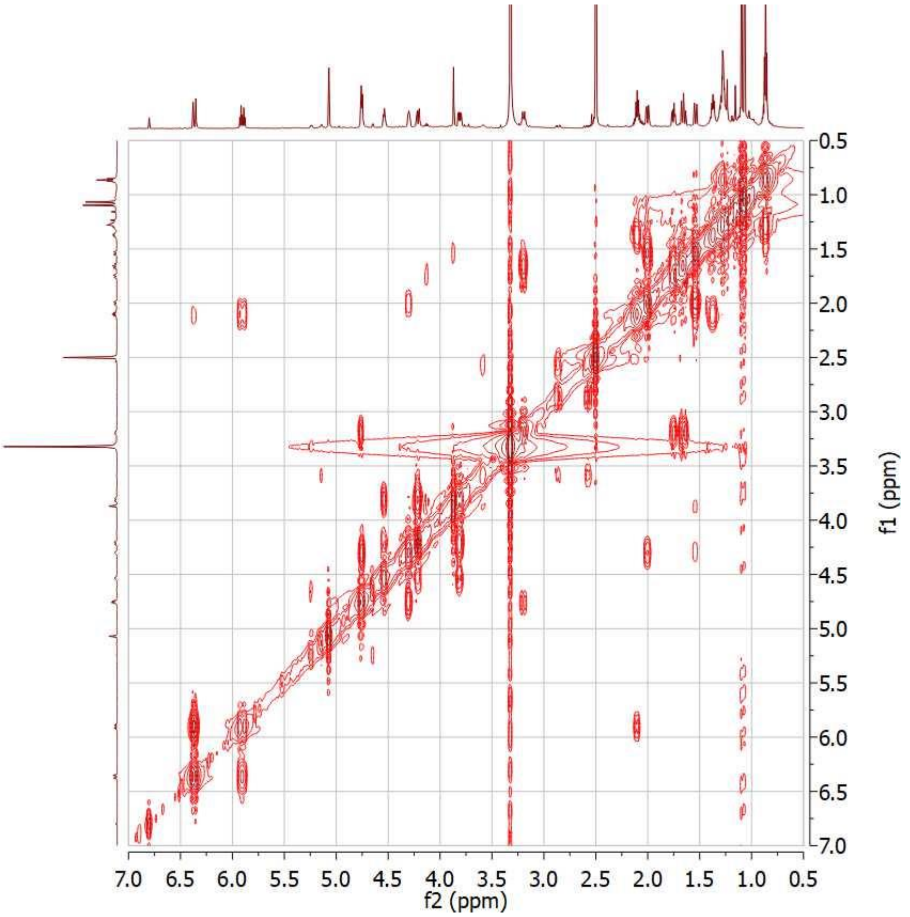


**Figure S74.** The $^{1}$H-$^{1}$H COSY (600 MHz, DMSO-$d_6$) spectrum of compound **15**.

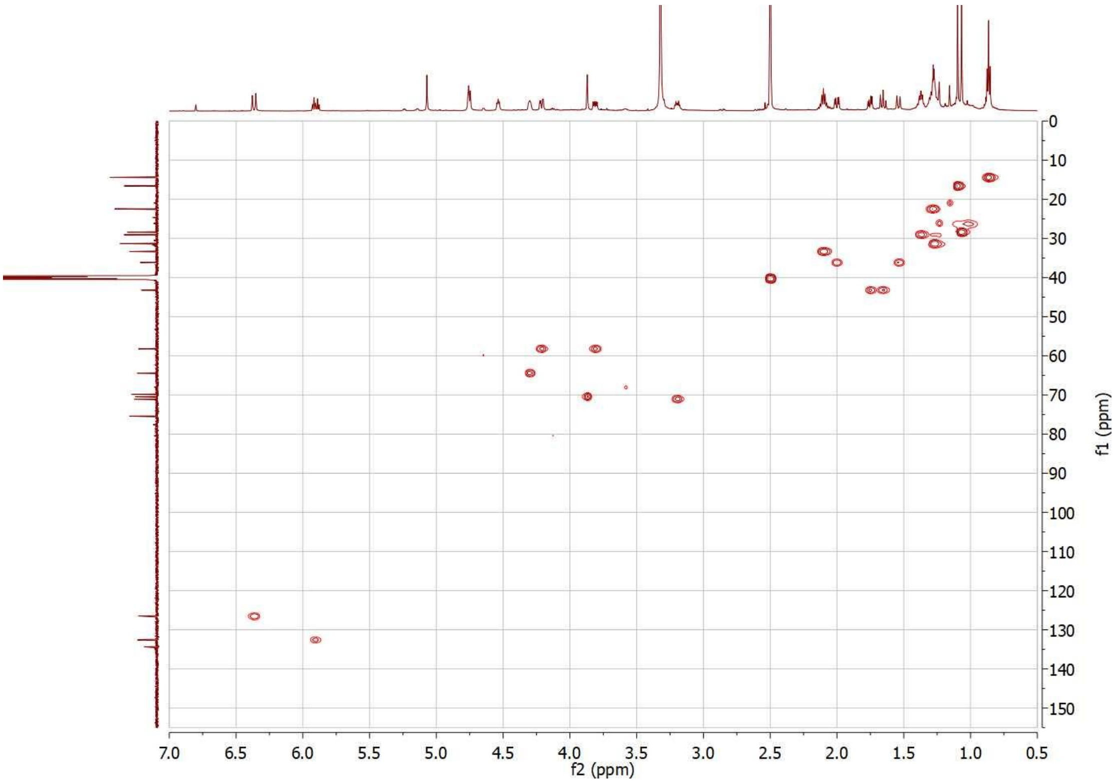

**Figure S75.** The HSQC (600 MHz, DMSO-$d_6$) spectrum of compound **15**.

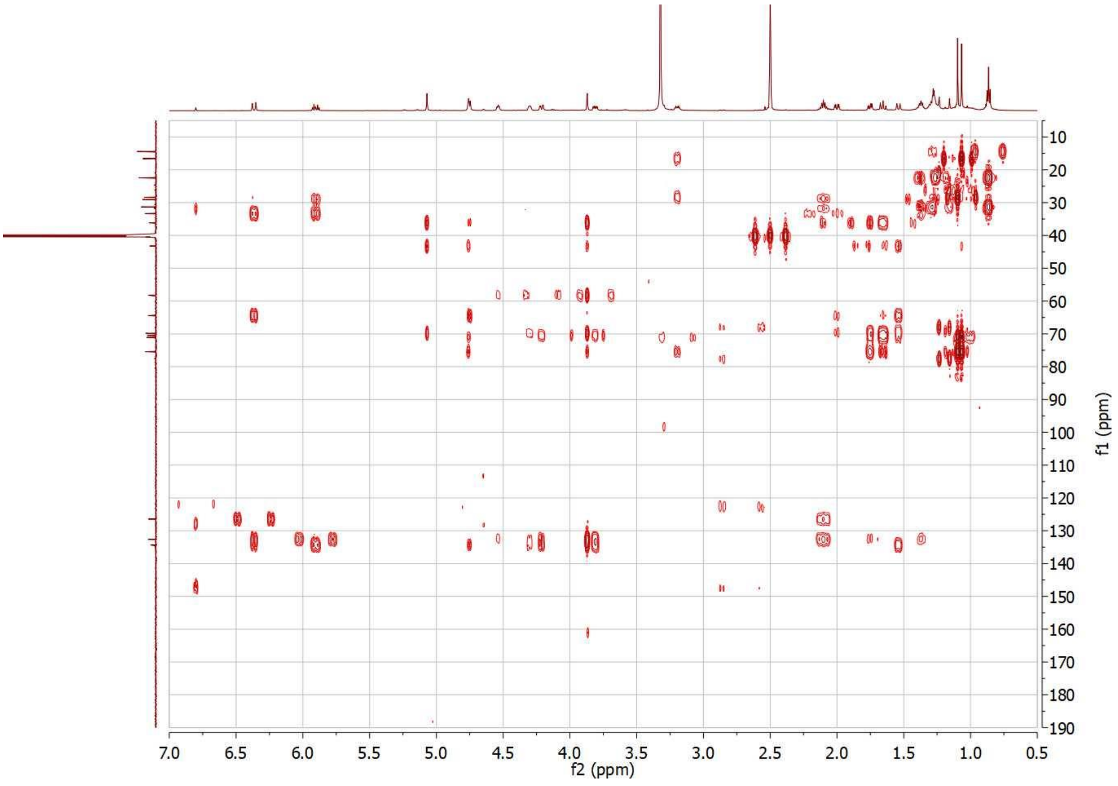

**Figure S76.** The HMBC (600 MHz, DMSO-$d_6$) spectrum of compound **15**.

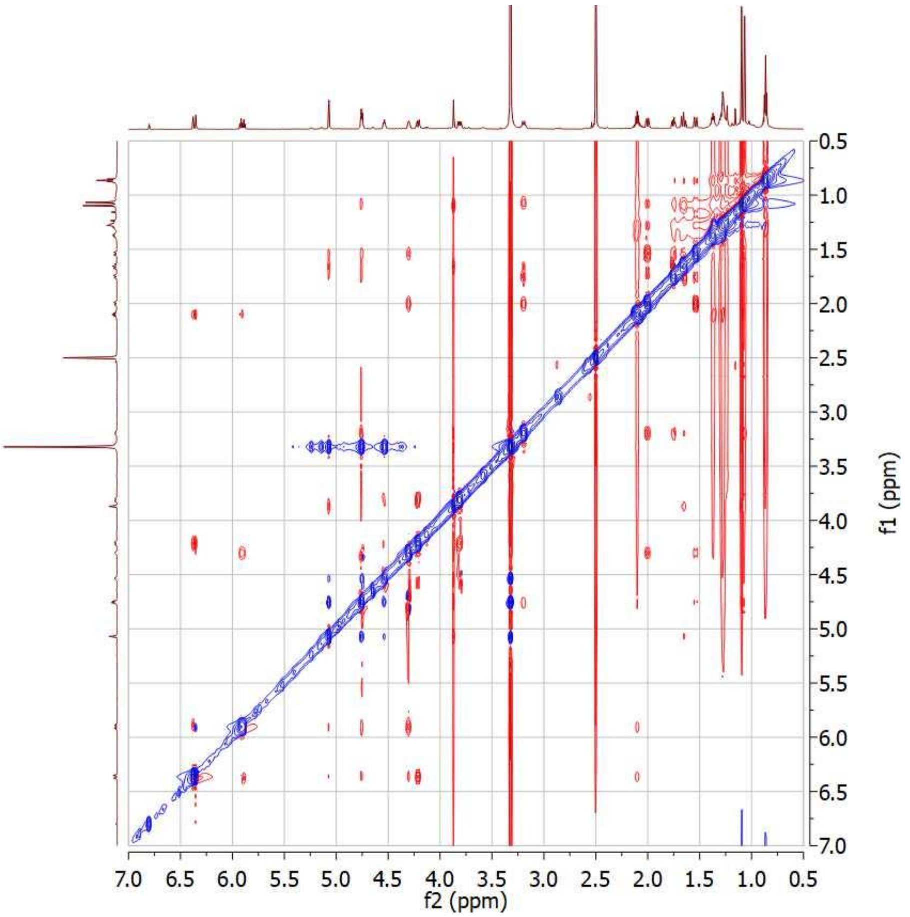


**Figure S77.** The ROESY (600 MHz, DMSO-$d_6$) spectrum of compound **15**.

**Acquisition Parameter**

| | | | | | |
|---|---|---|---|---|---|
| Source Type | ESI | Ion Polarity | Positive | Set Nebulizer | 0.3 Bar |
| Focus | Not active | Set Capillary | 4000 V | Set Dry Heater | 180 °C |
| Scan Begin | 50 m/z | Set End Plate Offset | -500 V | Set Dry Gas | 4.0 l/min |
| Scan End | 1500 m/z | Set Collision Cell RF | 600.0 Vpp | Set Divert Valve | Source |

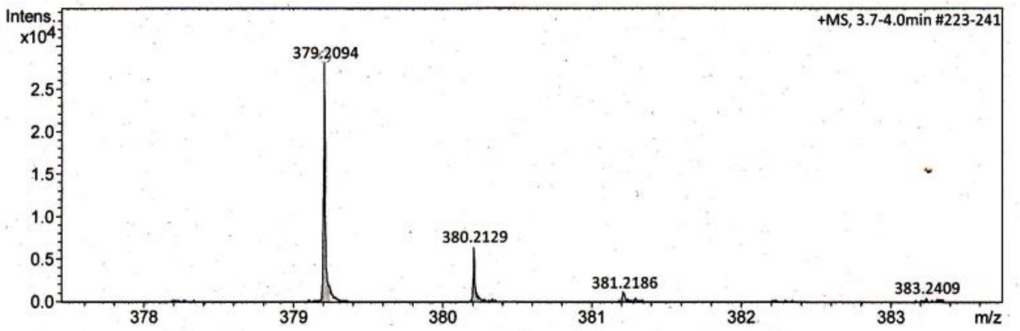


| Meas. m/z | # | Ion Formula | m/z | err [ppm] | mSigma | # mSigma | Score | rdb | e⁻ Conf | N-Rule |
|---|---|---|---|---|---|---|---|---|---|---|
| 379.2094 | 1 | C19H32NaO6 | 379.2091 | -0.8 | 10.0 | 1 | 100.00 | 3.5 | even | ok |

**Figure S78.** The HREISMS of compound **16**.

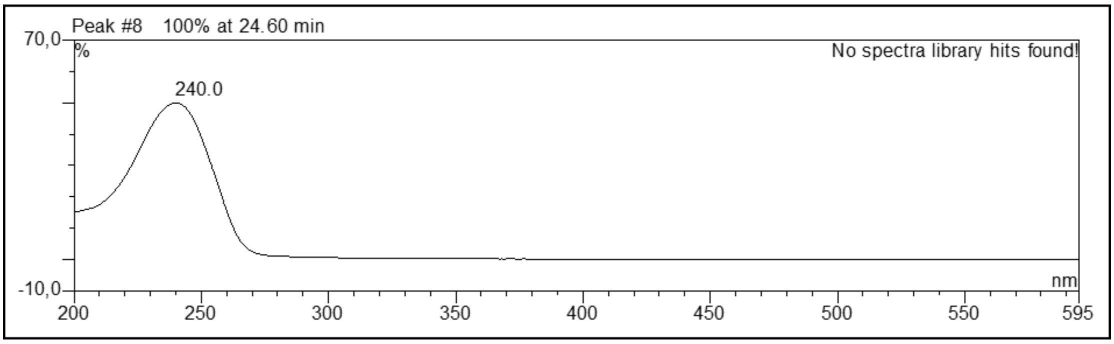


**Figure S79.** The UV spectrum of compound **16**.

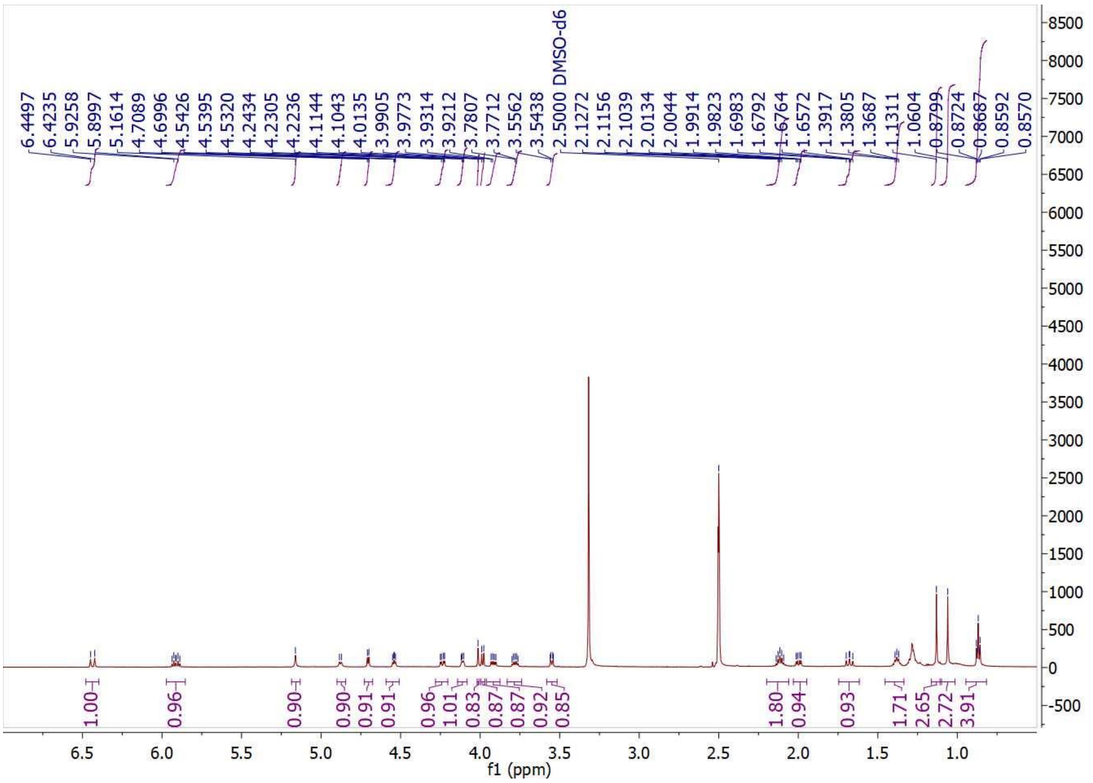


**Figure S80.** The $^{1}$H-NMR (600 MHz, DMSO-$d_6$) spectrum of compound **16**.

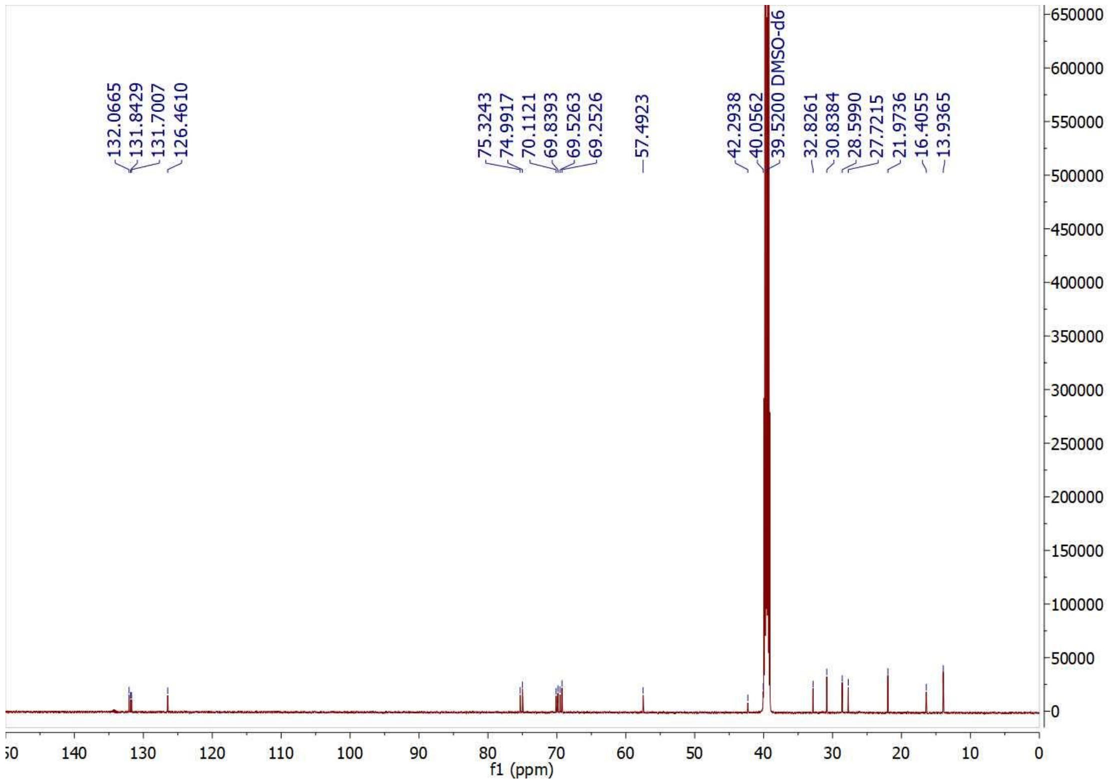


**Figure S81.** The $^{13}$C-NMR (150 MHz, DMSO-$d_6$) spectrum of compound **16**.

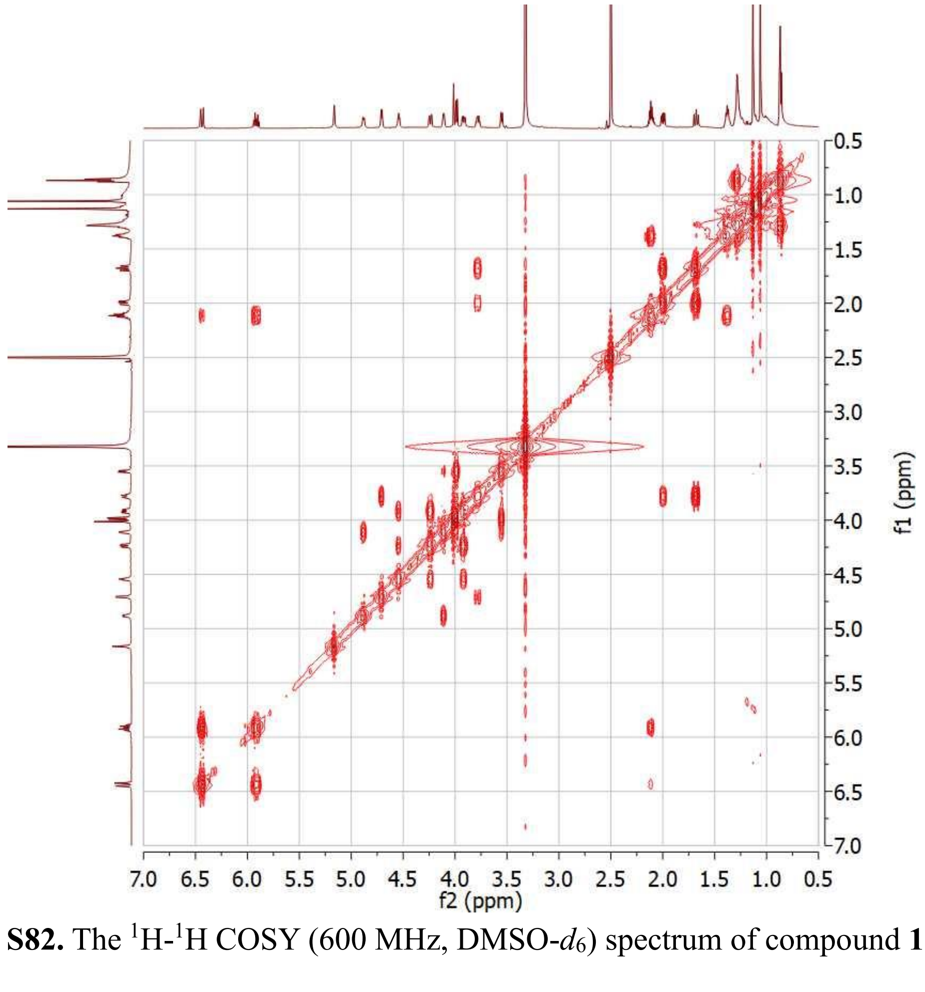


**Figure S82.** The $^{1}$H-$^{1}$H COSY (600 MHz, DMSO-$d_6$) spectrum of compound **16**.

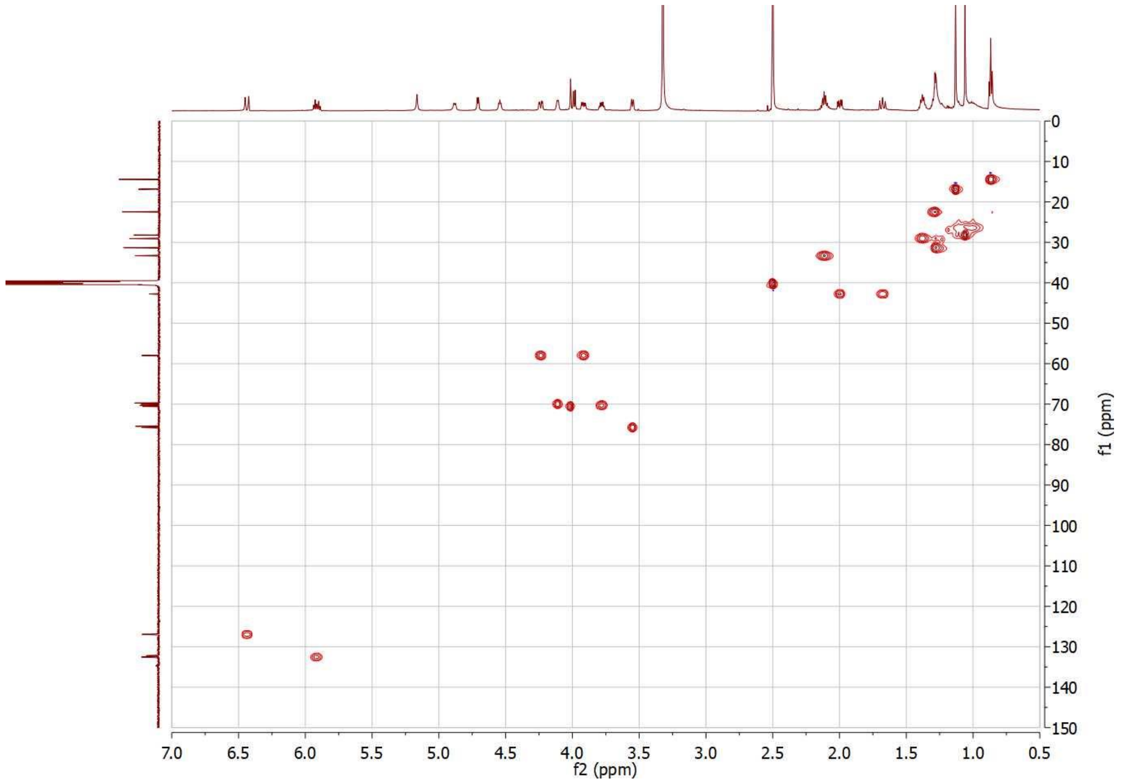


**Figure S83.** The HSQC (600 MHz, DMSO-$d_6$) spectrum of compound **16**.

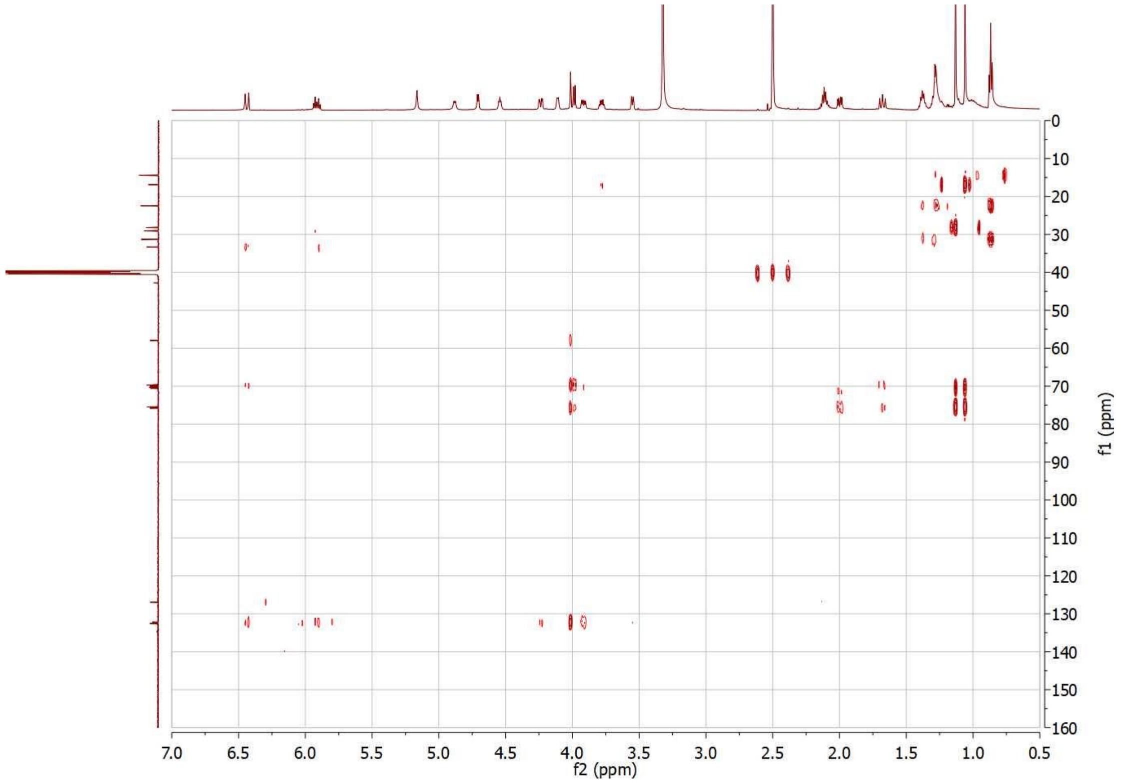


**Figure S84.** The HMBC (600 MHz, DMSO-$d_6$) spectrum of compound **16**.

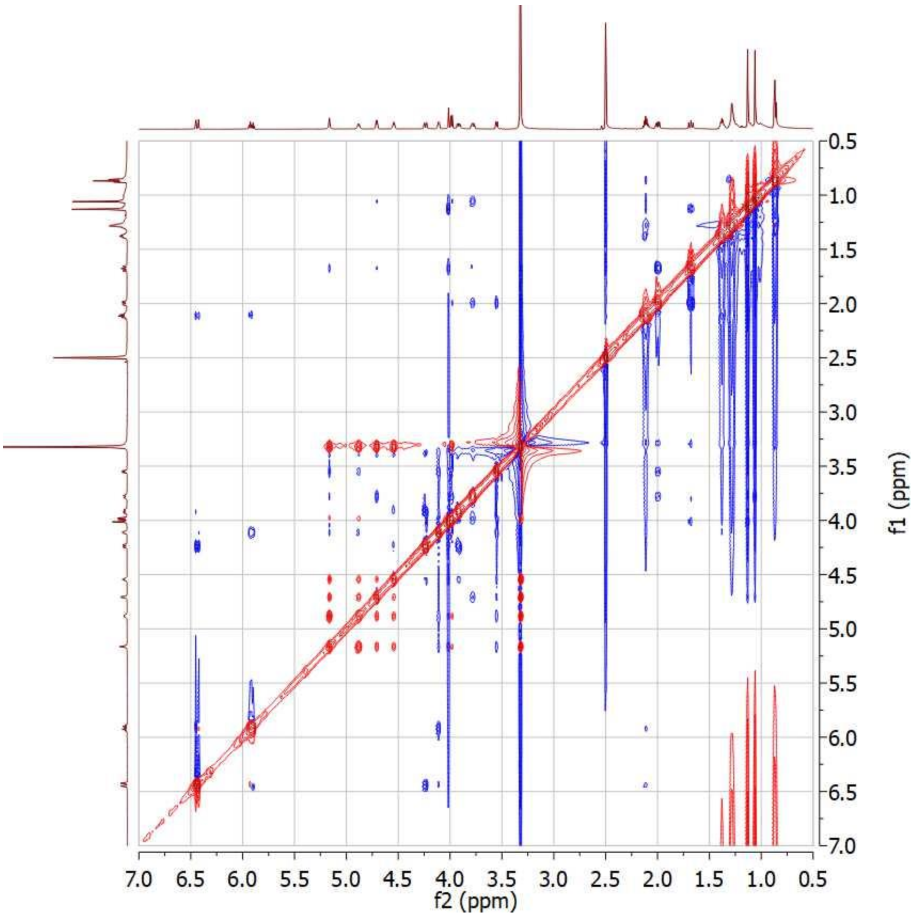


**Figure S85.** The ROESY (600 MHz, DMSO-$d_6$) spectrum of compound **16**.

**Acquisition Parameter**

| | | | | | |
|---|---|---|---|---|---|
| Source Type | ESI | Ion Polarity | Positive | Set Nebulizer | 0.3 Bar |
| Focus | Not active | Set Capillary | 4000 V | Set Dry Heater | 180 °C |
| Scan Begin | 50 m/z | Set End Plate Offset | -500 V | Set Dry Gas | 4.0 l/min |
| Scan End | 1500 m/z | Set Collision Cell RF | 600.0 Vpp | Set Divert Valve | Source |

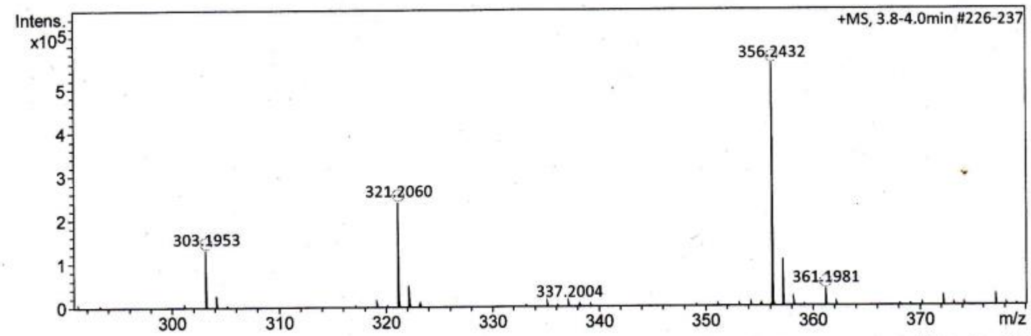


| Meas. m/z | # | Ion Formula | m/z | err [ppm] | mSigma | # mSigma | Score | rdb | e⁻ Conf | N-Rule |
|---|---|---|---|---|---|---|---|---|---|---|
| 303.1953 | 1 | C19H27O3 | 303.1955 | 0.5 | 6.3 | 1 | 100.00 | 6.5 | even | ok |
| 321.2060 | 1 | C19H29O4 | 321.2060 | 0.1 | 4.9 | 1 | 100.00 | 5.5 | even | ok |
| | 2 | C18H26N4Na | 321.2050 | -3.3 | 7.5 | 2 | 57.53 | 7.5 | even | ok |
| | 3 | C20H25N4 | 321.2074 | 4.2 | 18.5 | 3 | 38.70 | 10.5 | even | ok |
| 356.2432 | 1 | C19H34NO5 | 356.2431 | -0.2 | 13.3 | 1 | 100.00 | 3.5 | even | ok |
| | 2 | C18H31N5NaO | 356.2421 | -3.2 | 14.5 | 2 | 57.33 | 5.5 | even | ok |

**Figure S86.** The HREISMS of compound **17**.

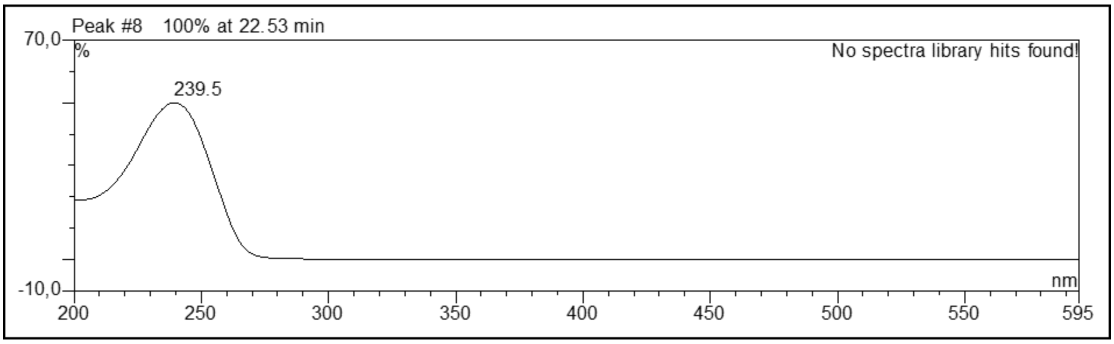


**Figure S87.** The UV spectrum of compound **17**.

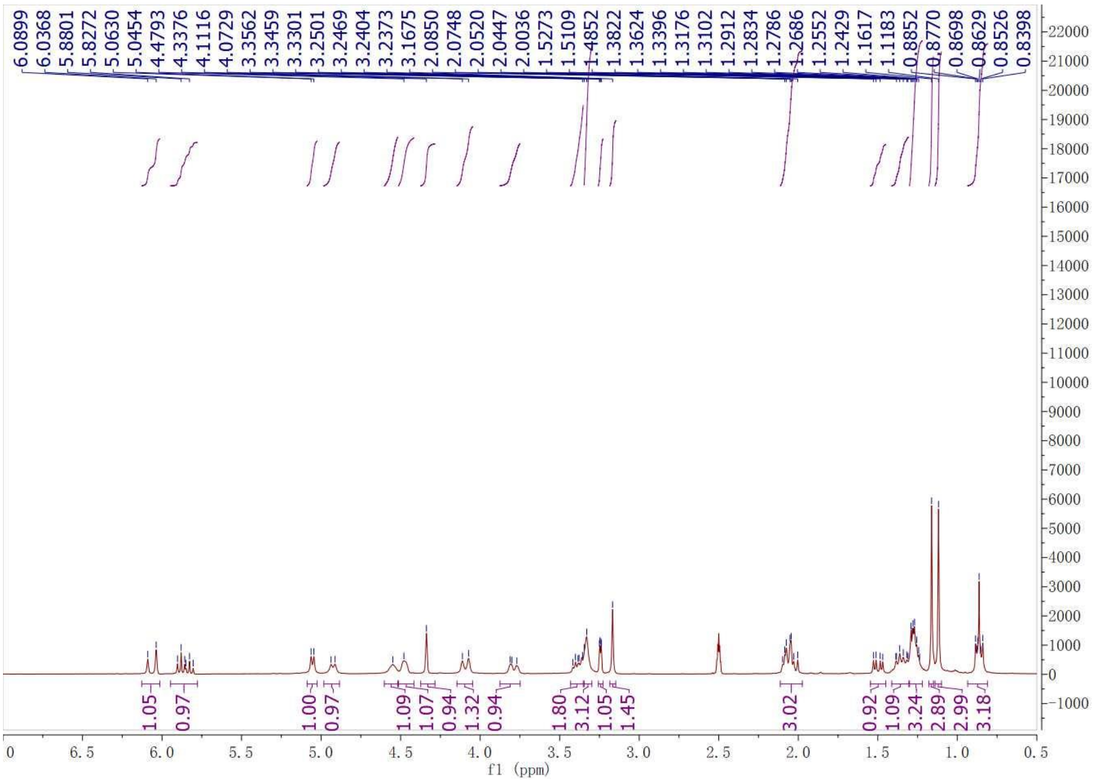


**Figure S88.** The $^{1}$H-NMR (300 MHz, DMSO-$d_6$) spectrum of compound **17**.

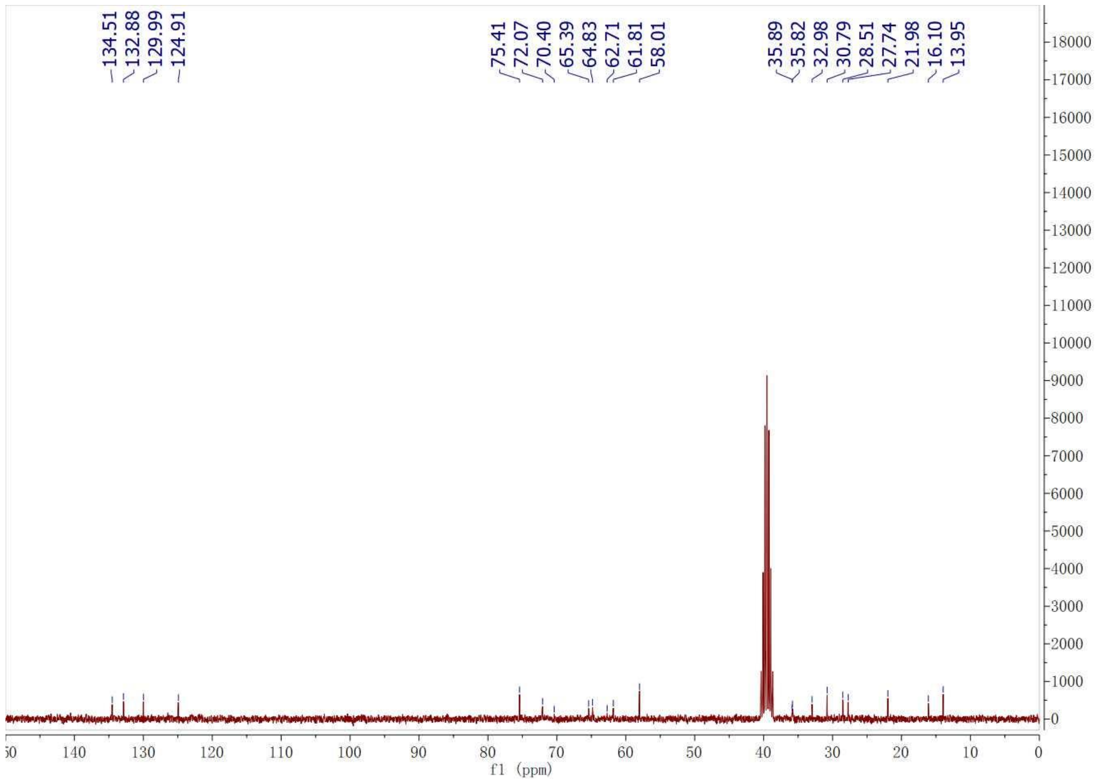


**Figure S89**. The $^{13}$C-NMR (75 MHz, DMSO-$d_6$) spectrum of compound **17**.

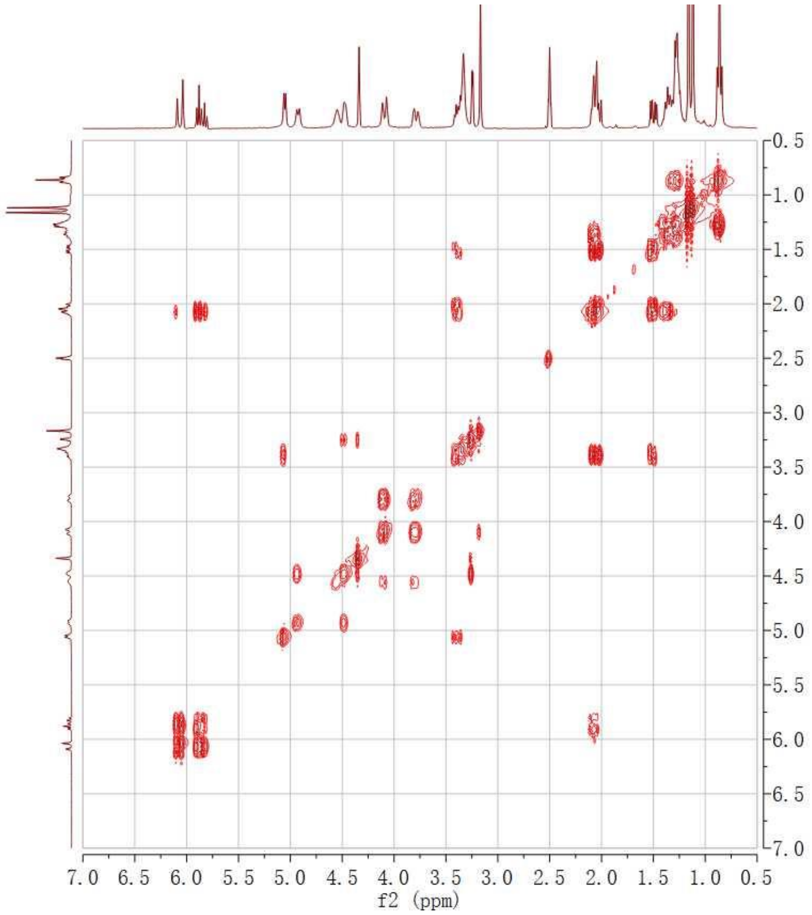


**Figure S90**. The $^{1}$H-$^{1}$H COSY (300 MHz, DMSO-$d_6$) spectrum of compound **17**.

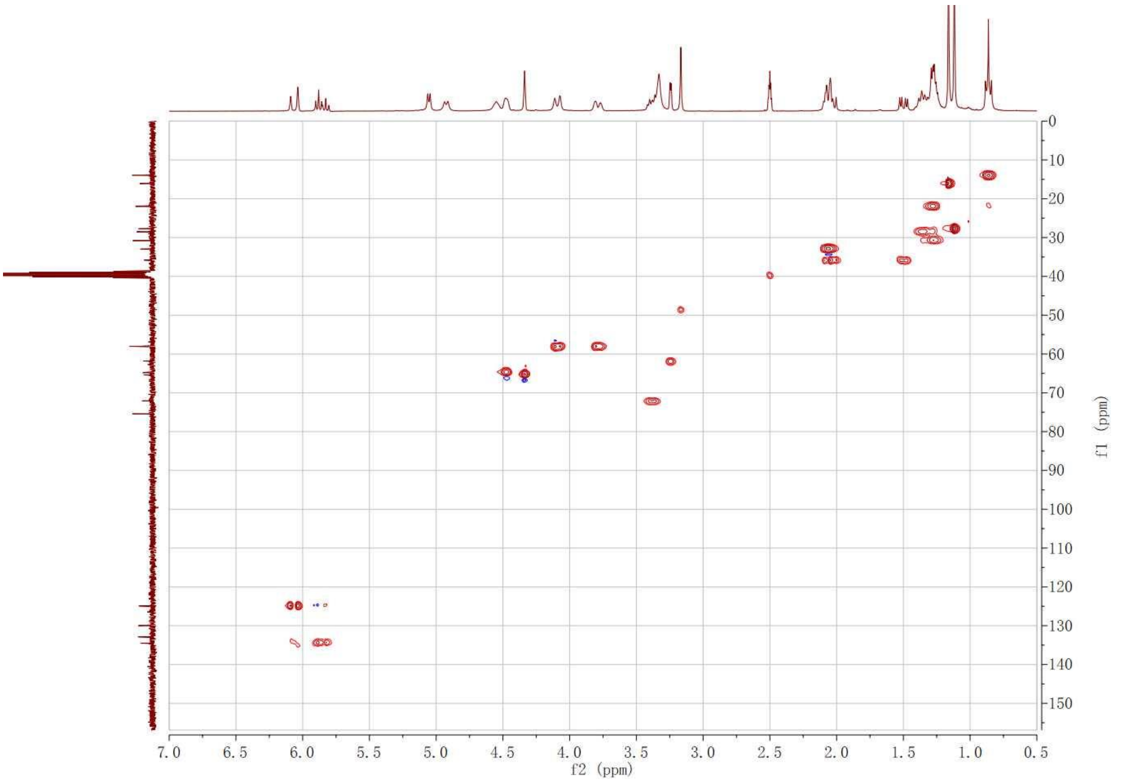


**Figure S91.** The HSQC (300 MHz, DMSO-$d_6$) spectrum of compound **17**.

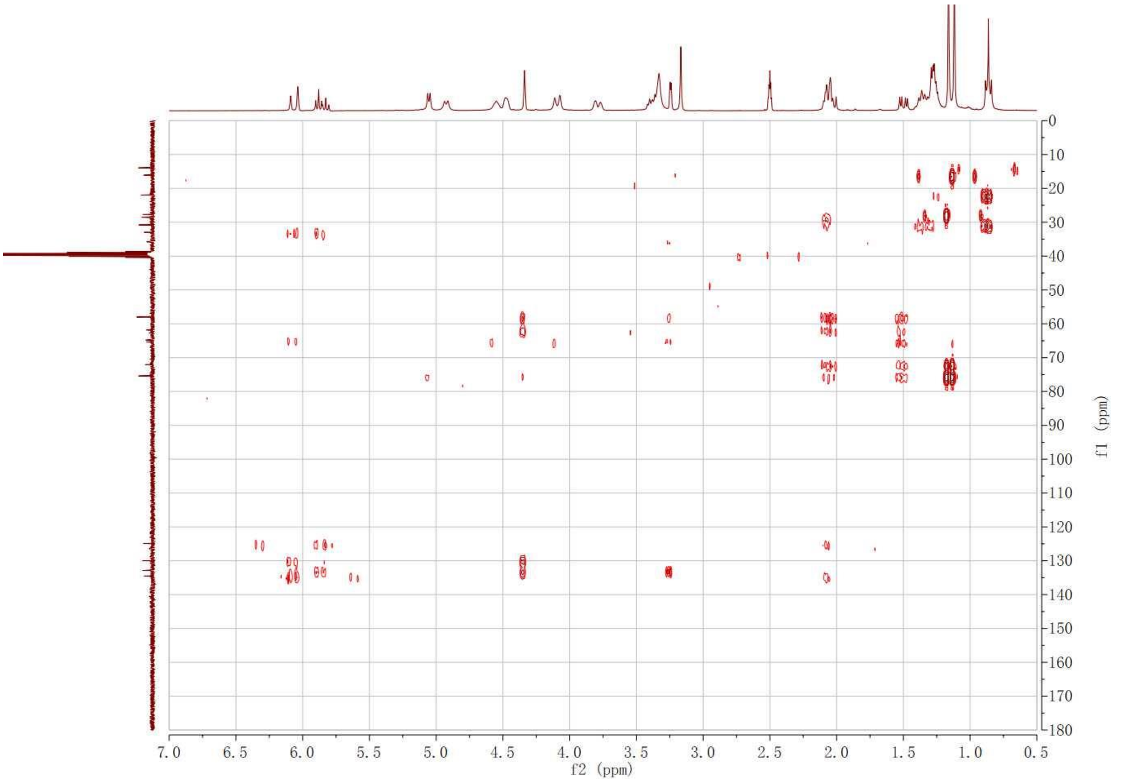


**Figure S92.** The HMBC (300 MHz, DMSO-$d_6$) spectrum of compound **17**.

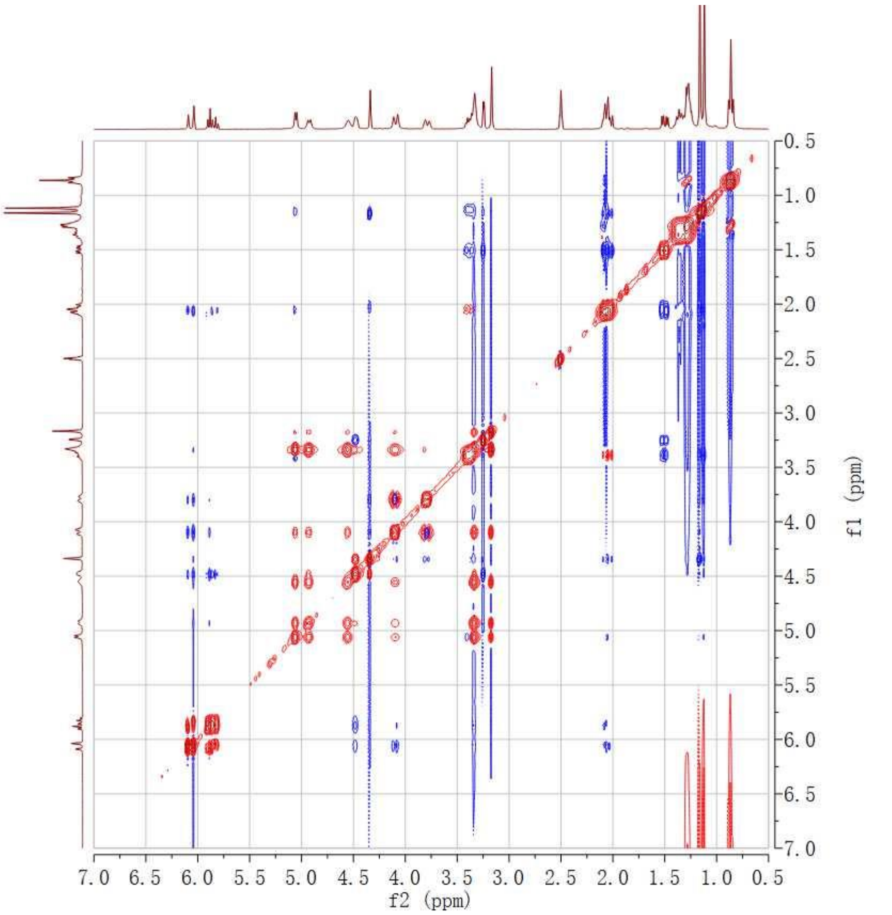


Figure S93. The ROESY (300 MHz, DMSO-$d_6$) spectrum of compound **17**.

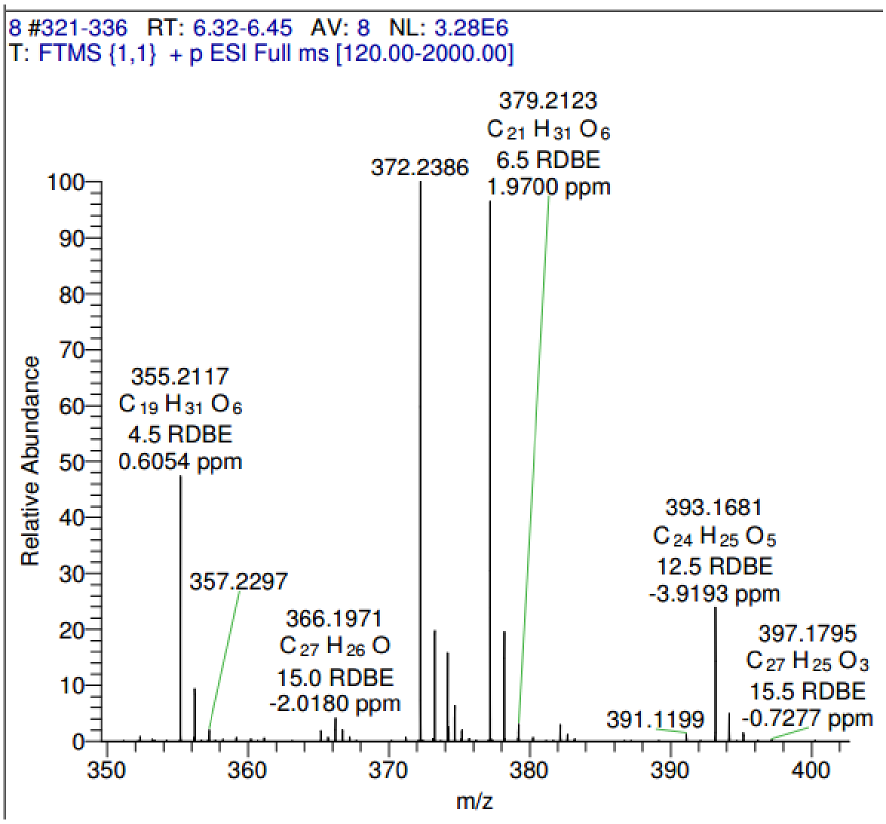


**Figure S94.** The HREISMS of compound **18**.

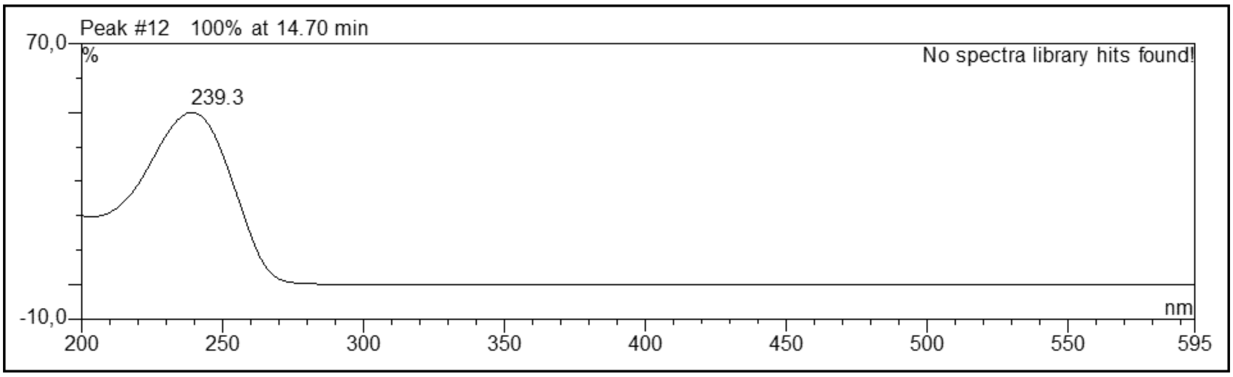


**Figure S95.** The UV spectrum of compound **18**.

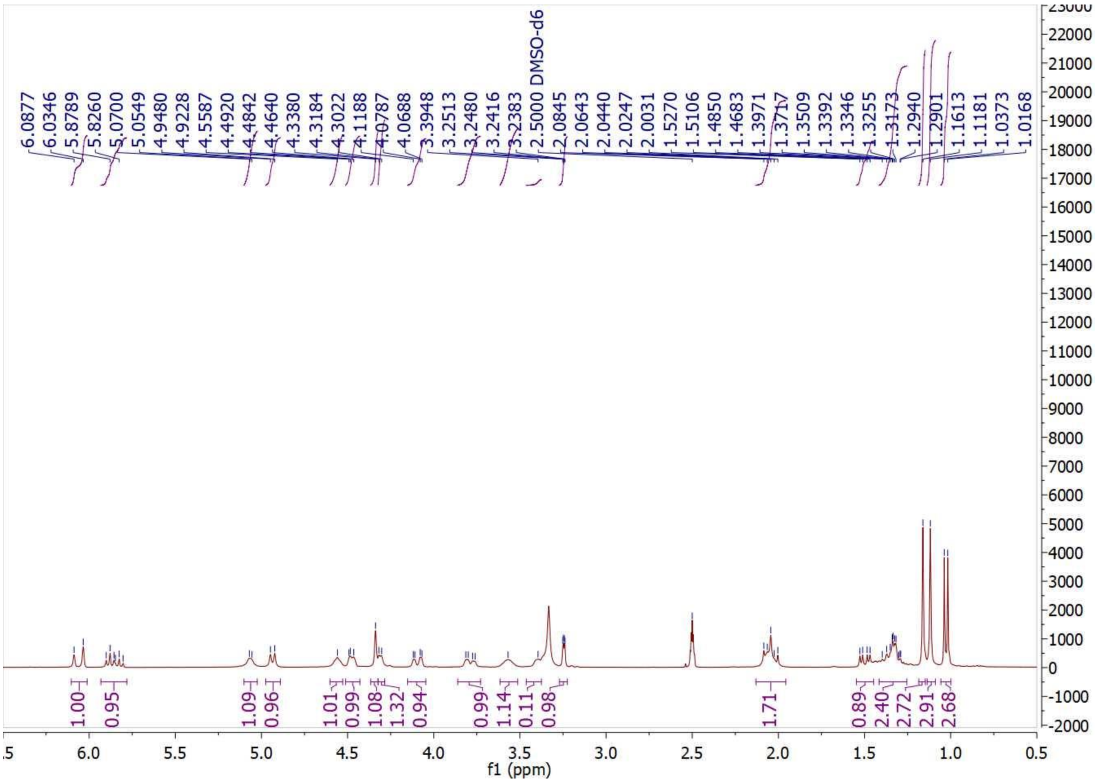


**Figure S96.** The $^{1}$H-NMR (300 MHz, DMSO-$d_6$) spectrum of compound **18**.

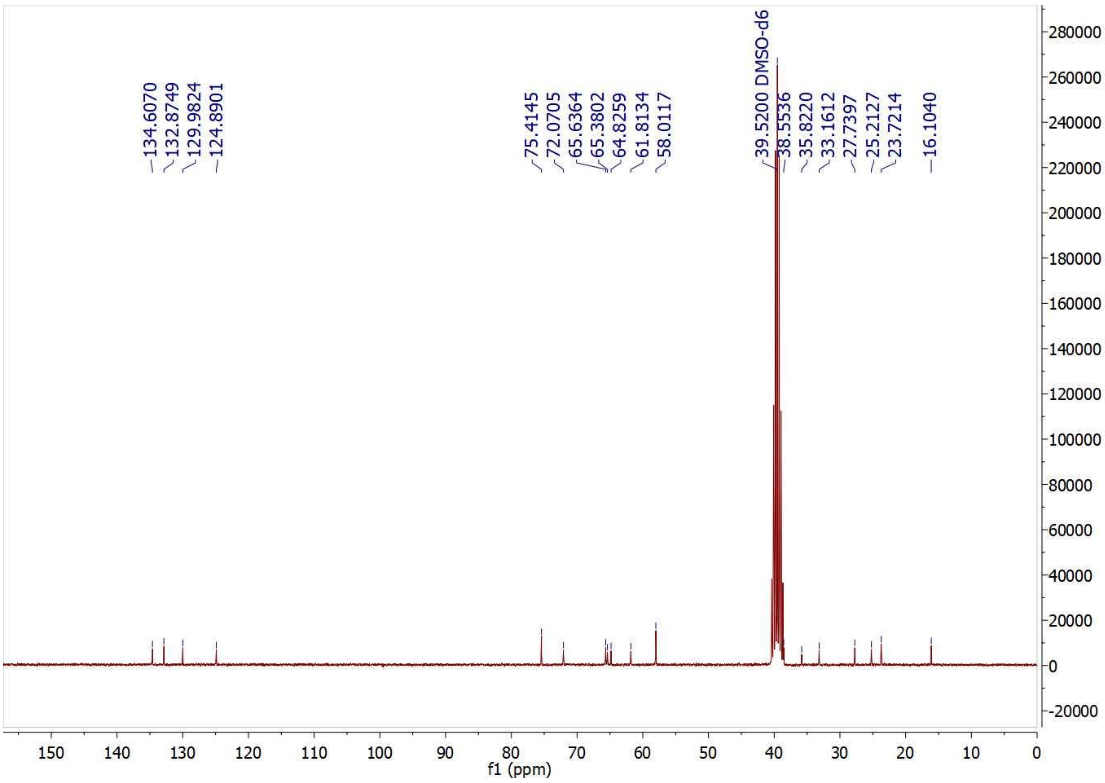


**Figure S97**. The $^{13}$C-NMR (75 MHz, DMSO-$d_6$) spectrum of compound **18**.

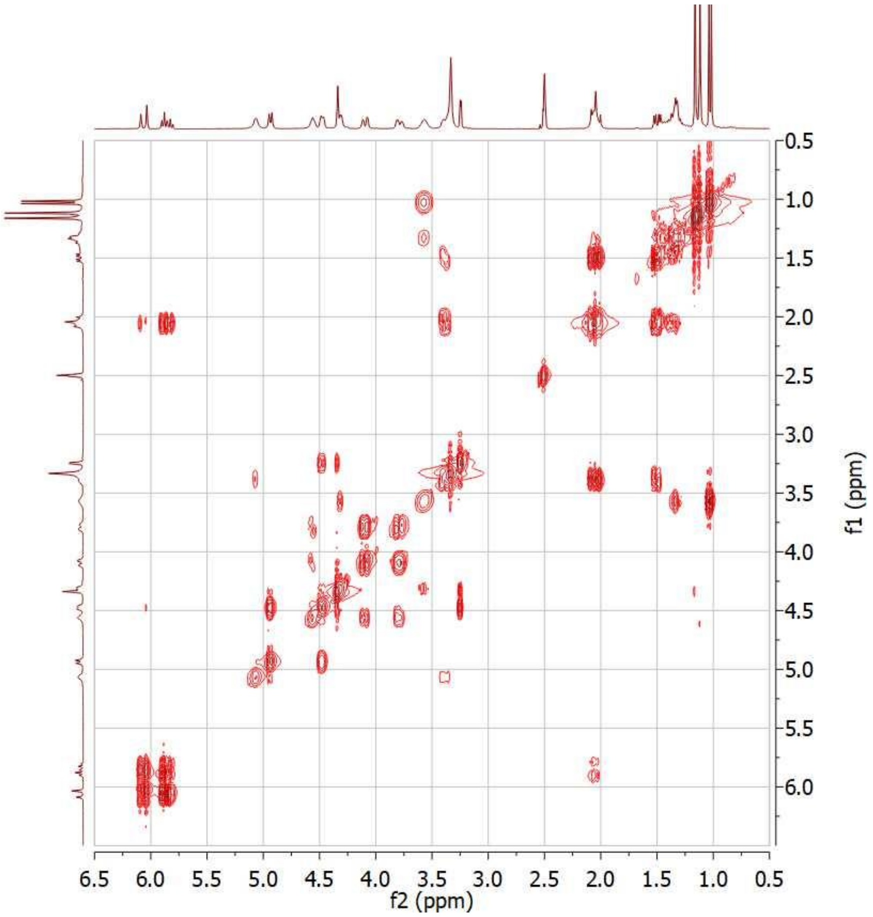


**Figure S98.** The $^{1}$H-$^{1}$H COSY (300 MHz, DMSO-$d_6$) spectrum of compound **18**.

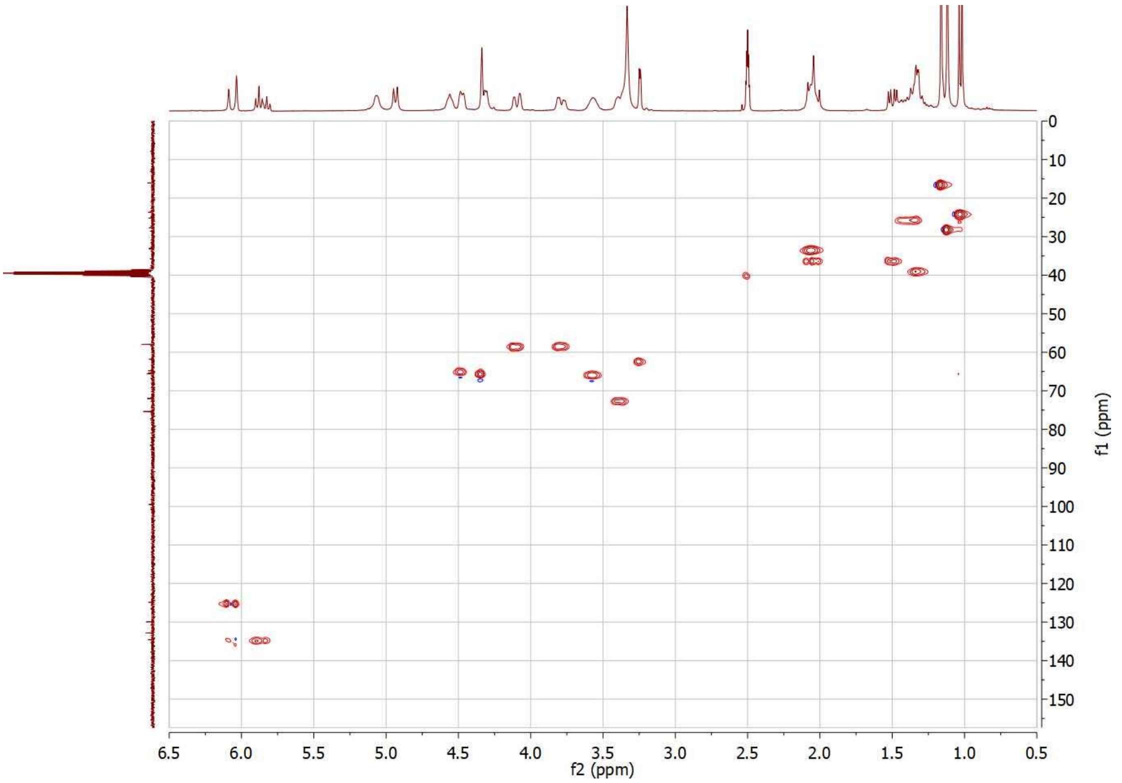


**Figure S99.** The HSQC (300 MHz, DMSO-$d_6$) spectrum of compound **18**.

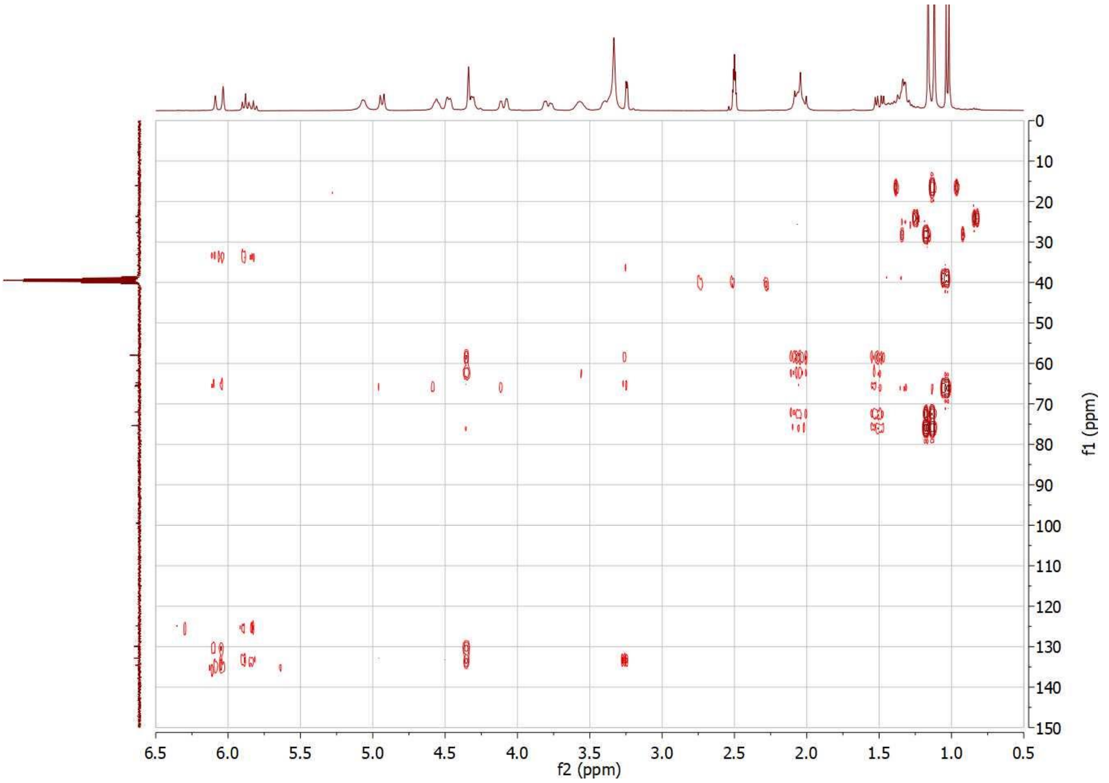


**Figure S100.** The HMBC (300 MHz, DMSO-$d_6$) spectrum of compound **18**.

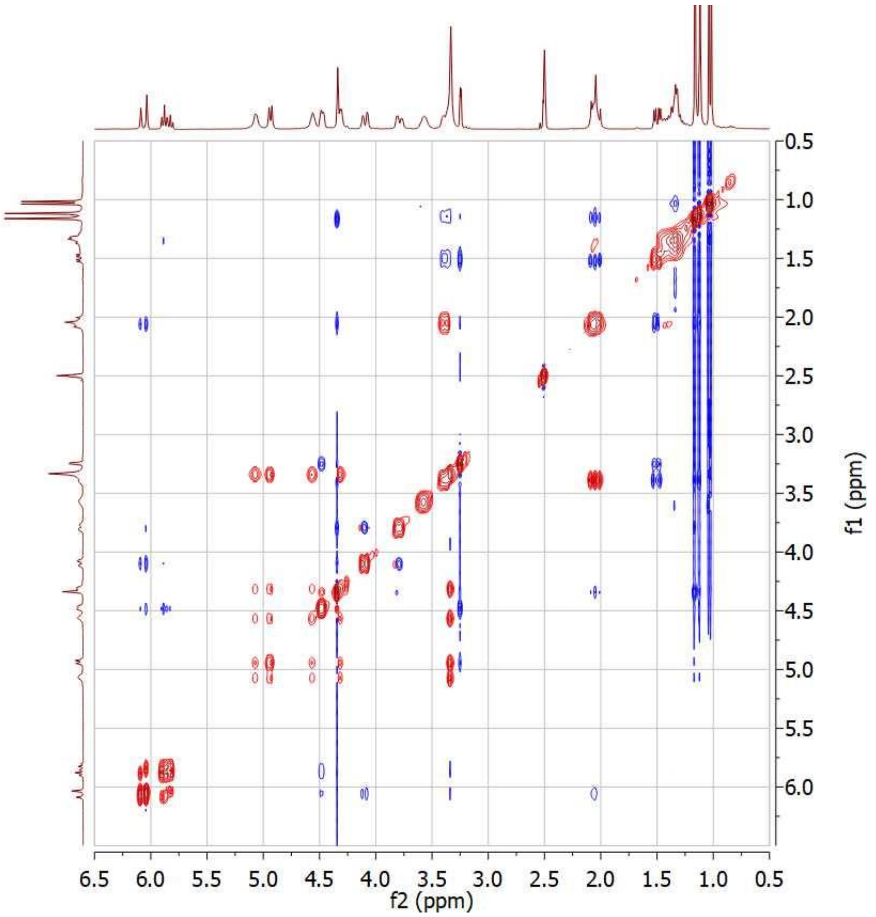


**Figure S101.** The ROESY (300 MHz, DMSO-$d_6$) spectrum of compound **18**.

**Acquisition Parameter**

| | | | | | |
|---|---|---|---|---|---|
| Source Type | ESI | Ion Polarity | Positive | Set Nebulizer | 0.3 Bar |
| Focus | Not active | Set Capillary | 4000 V | Set Dry Heater | 180 °C |
| Scan Begin | 50 m/z | Set End Plate Offset | -500 V | Set Dry Gas | 4.0 l/min |
| Scan End | 1500 m/z | Set Collision Cell RF | 600.0 Vpp | Set Divert Valve | Source |

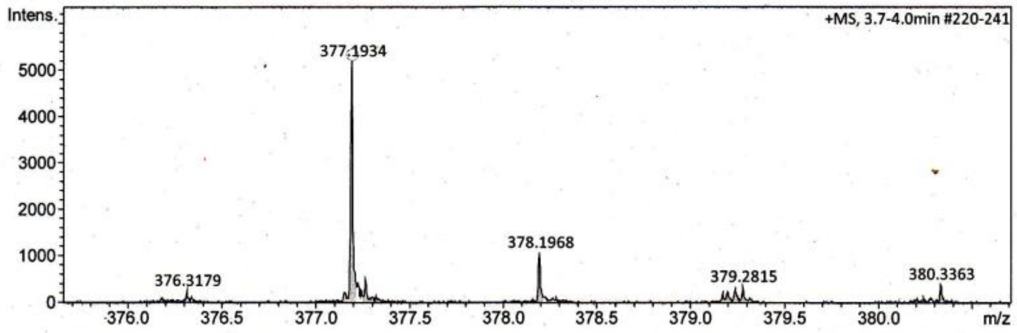


| Meas. m/z | # | Ion Formula | m/z | err [ppm] | mSigma | # mSigma | Score | rdb | e⁻ Conf | N-Rule |
|---|---|---|---|---|---|---|---|---|---|---|
| 377.1934 | 1 | C19H30NaO6 | 377.1935 | 0.2 | 4.9 | 1 | 100.00 | 4.5 | even | ok |

**Figure S102.** The HREISMS of compound **19**.

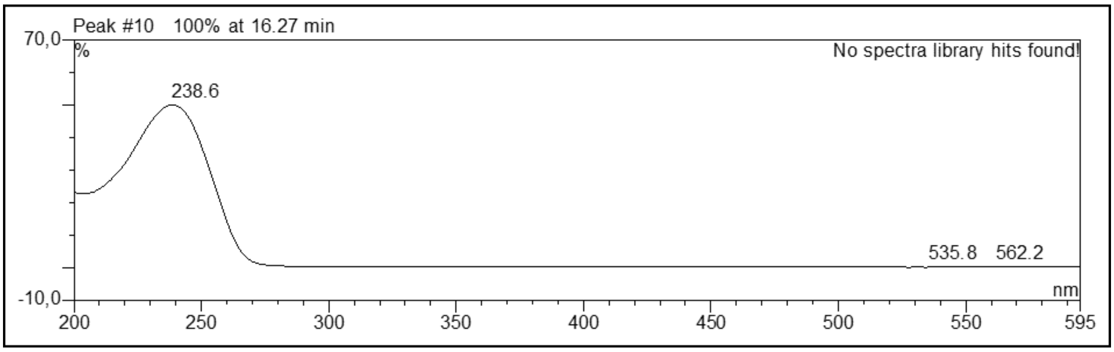


**Figure S103.** The UV spectrum of compound **19**.

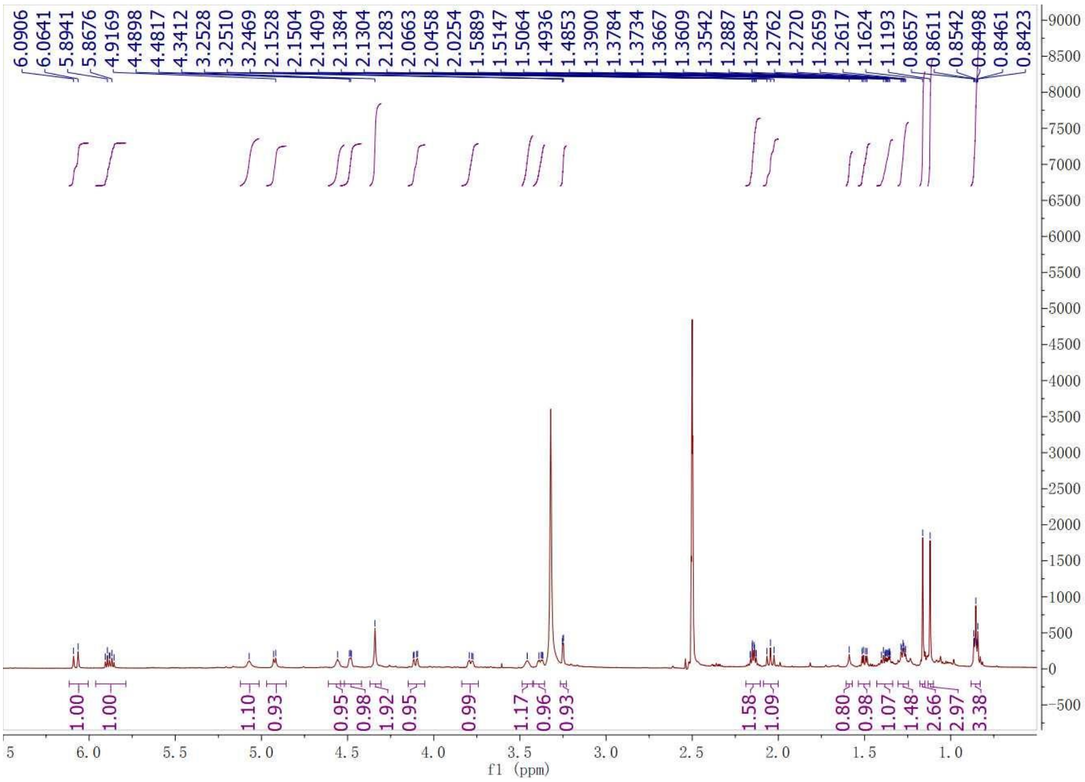


**Figure S104.** The $^1$H-NMR (600 MHz, DMSO-$d_6$) spectrum of compound **19**.

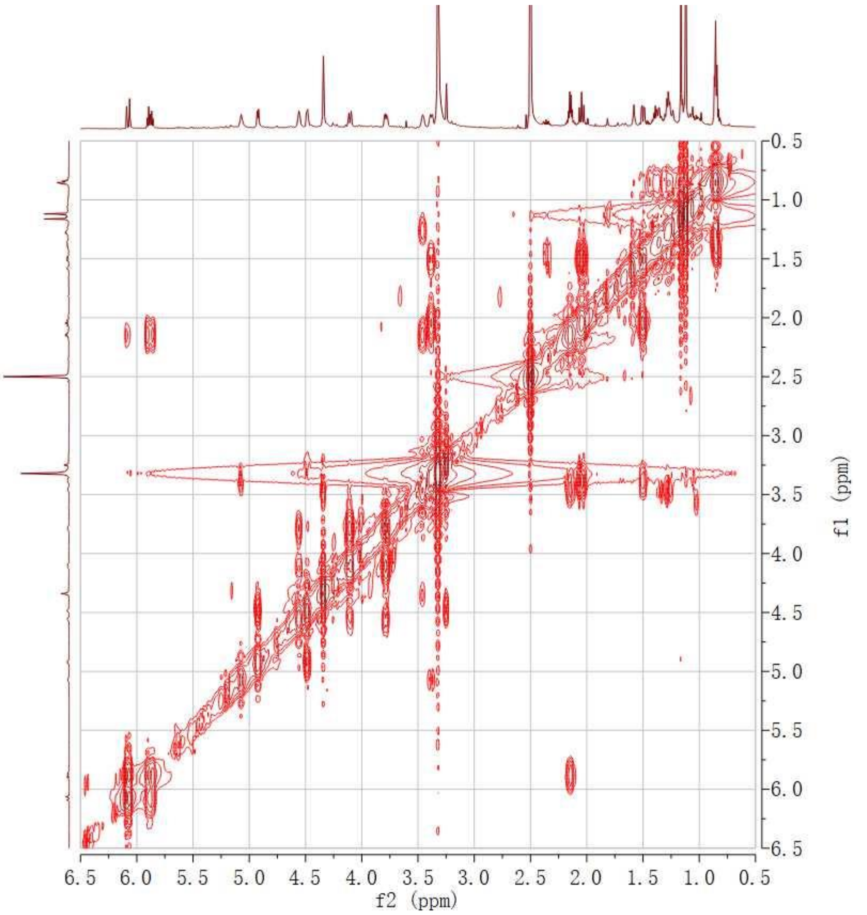


**Figure S105.** The $^1$H-$^1$H COSY (600 MHz, DMSO-$d_6$) spectrum of compound **19**.

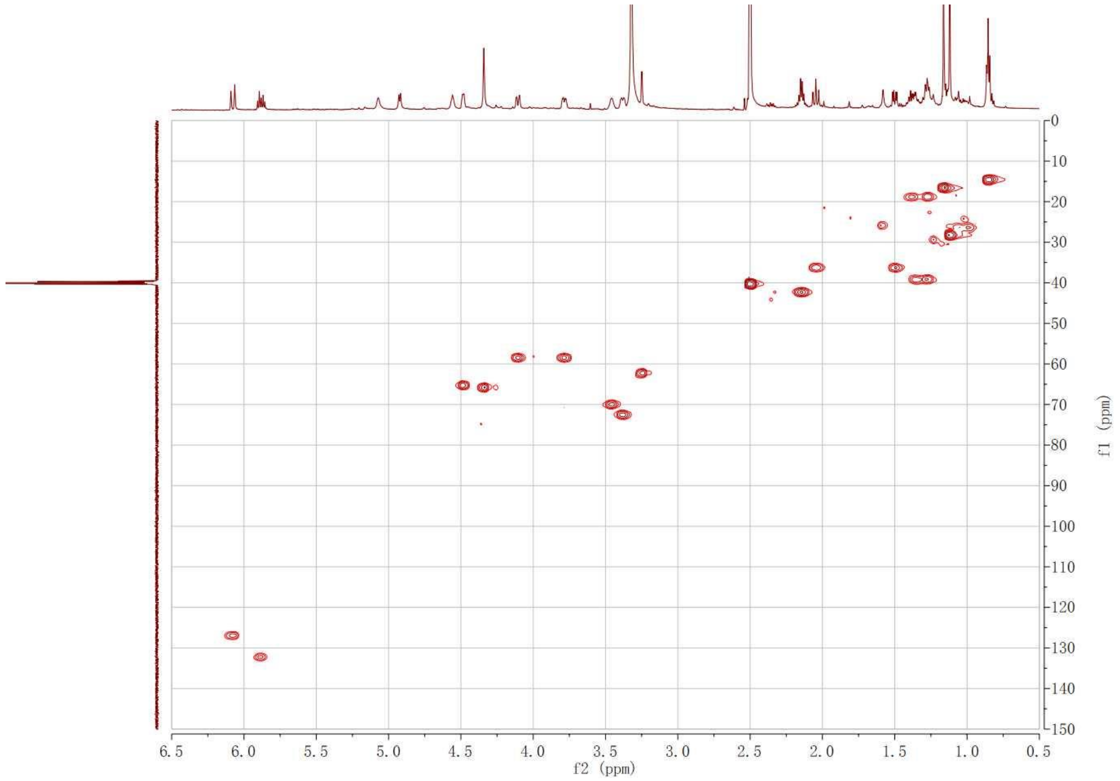


**Figure S106.** The HSQC (600MHz, DMSO-$d_6$) spectrum of compound **19**.

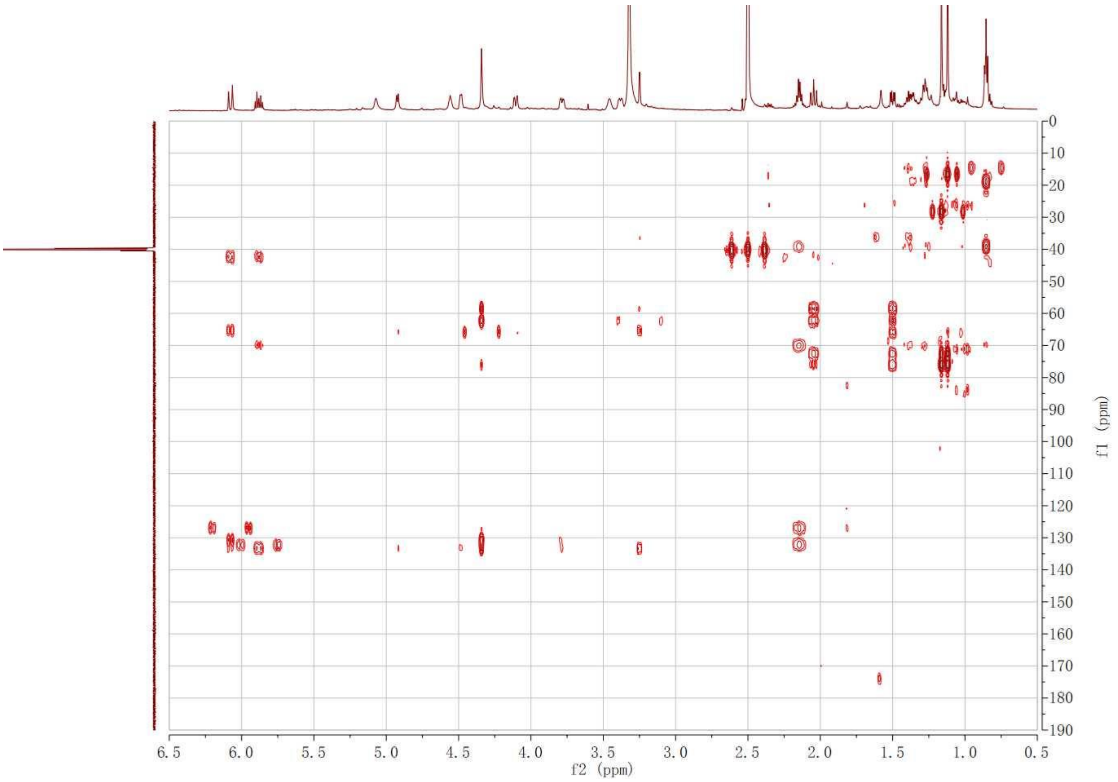


**Figure S107.** The HMBC (600MHz, DMSO-$d_6$) spectrum of compound **19**.

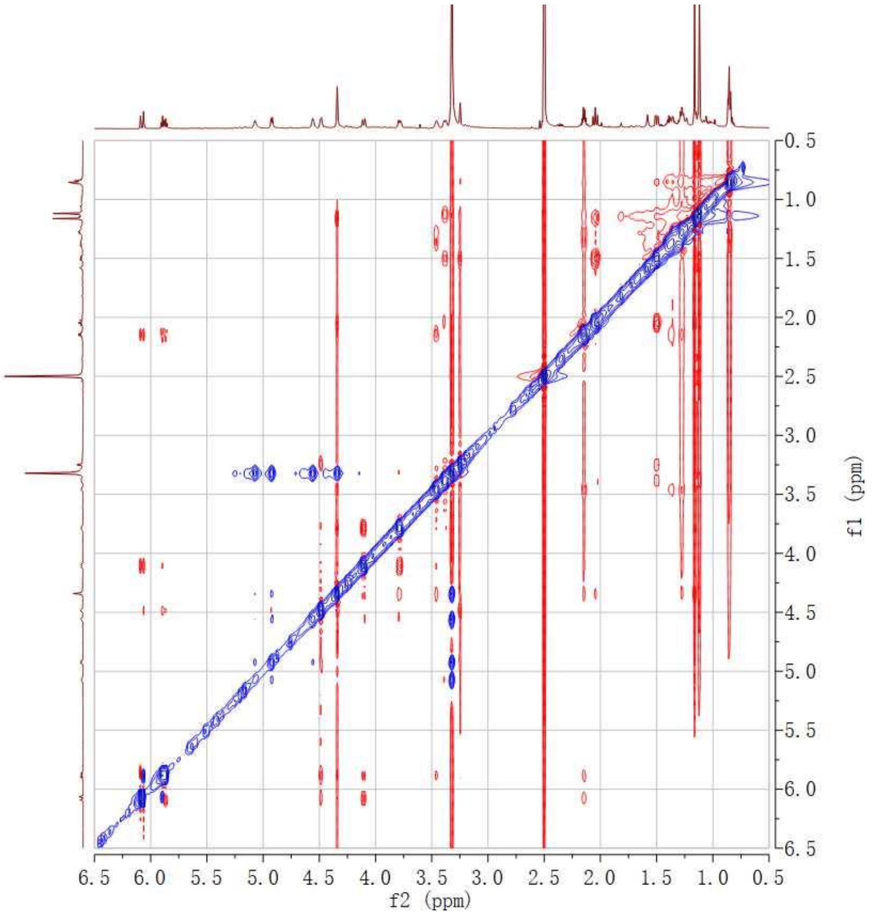


**Figure S108.** The ROESY (600 MHz, DMSO-$d_6$) spectrum of compound **19**.

**Acquisition Parameter**

| | | | | | |
|---|---|---|---|---|---|
| Source Type | ESI | Ion Polarity | Positive | Set Nebulizer | 0.3 Bar |
| Focus | Not active | Set Capillary | 4000 V | Set Dry Heater | 180 °C |
| Scan Begin | 50 m/z | Set End Plate Offset | -500 V | Set Dry Gas | 4.0 l/min |
| Scan End | 1500 m/z | Set Collision Cell RF | 600.0 Vpp | Set Divert Valve | Source |

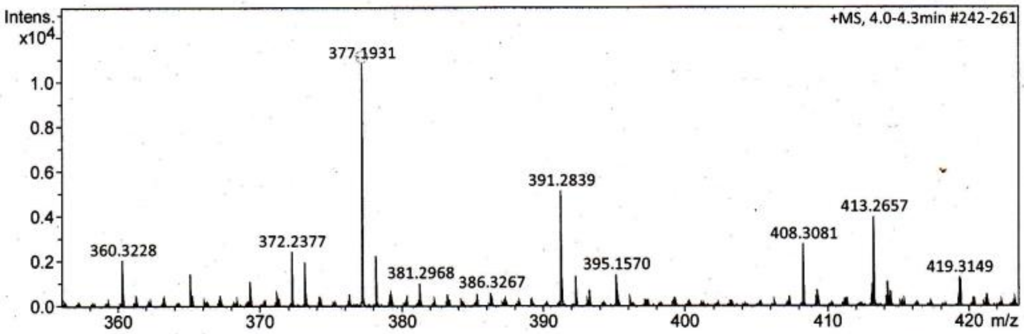


| Meas. m/z | # | Ion Formula | m/z | err [ppm] | mSigma | # mSigma | Score | rdb | e⁻ Conf | N-Rule |
|---|---|---|---|---|---|---|---|---|---|---|
| 377.1931 | 1 | C19H30NaO6 | 377.1935 | 1.1 | 8.5 | 1 | 100.00 | 4.5 | even | ok |
| | 2 | C16H22N10Na | 377.1921 | -2.5 | 15.3 | 2 | 66.94 | 10.5 | even | ok |
| | 3 | C20H26N4NaO2 | 377.1948 | 4.6 | 19.3 | 3 | 36.92 | 9.5 | even | ok |

**Figure S109.** The HREISMS of compound **20**.

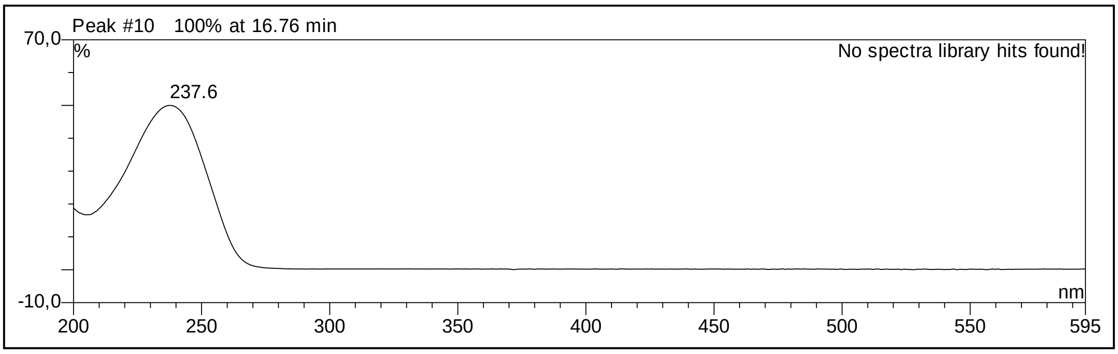


**Figure S110.**The UV spectrum of compound **20**.

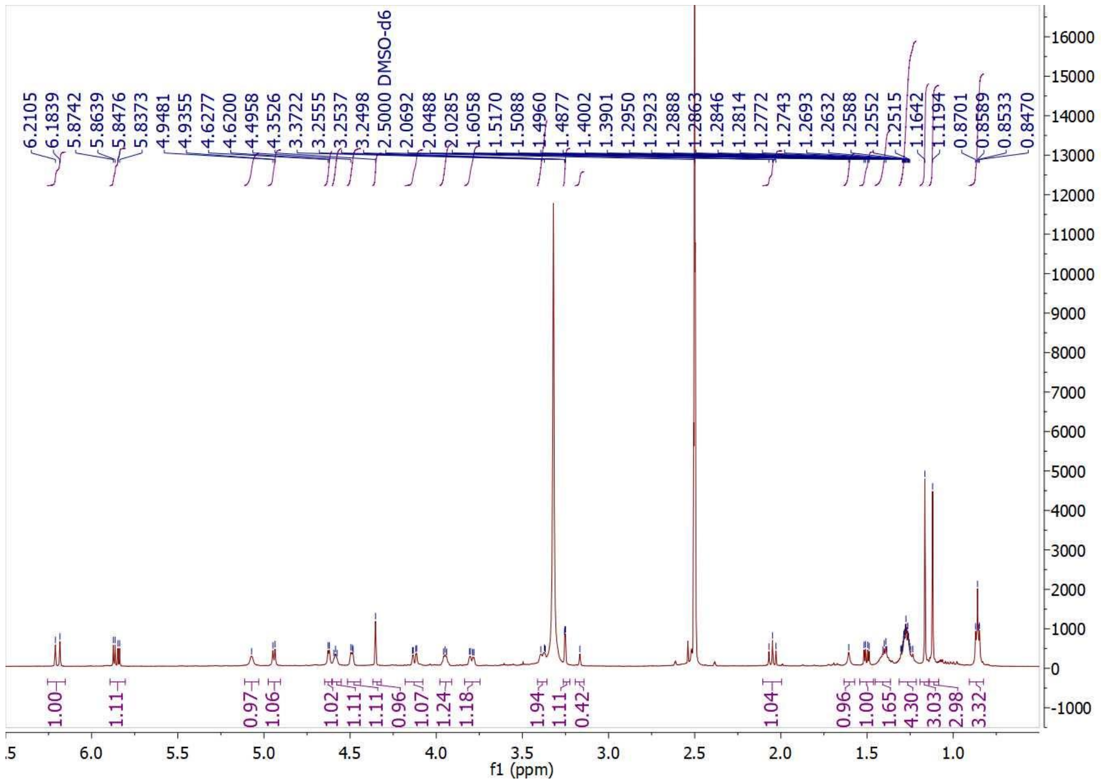

**Figure S111.**The $^{1}$H-NMR (600 MHz, DMSO-$d_6$) spectrum of compound **20**.

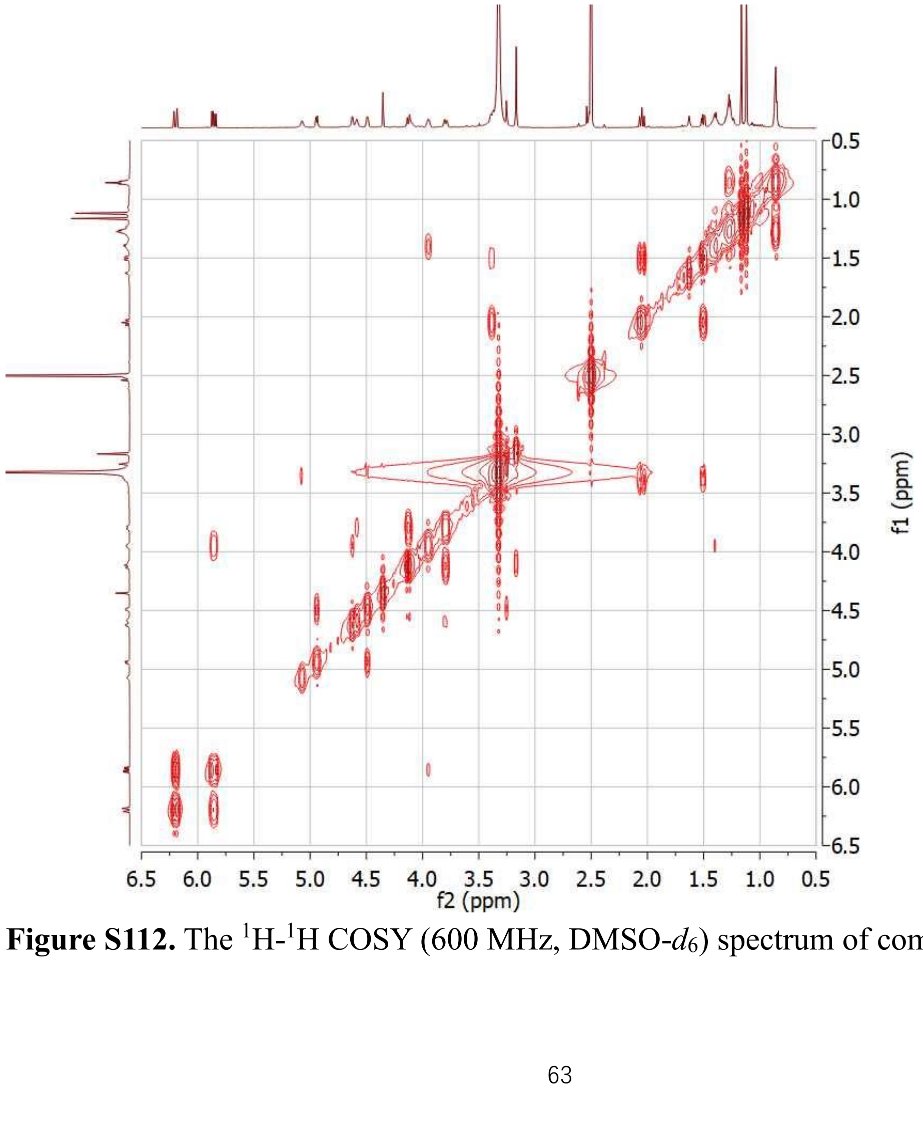

**Figure S112.** The $^{1}$H-$^{1}$H COSY (600 MHz, DMSO-$d_6$) spectrum of compound **20**.

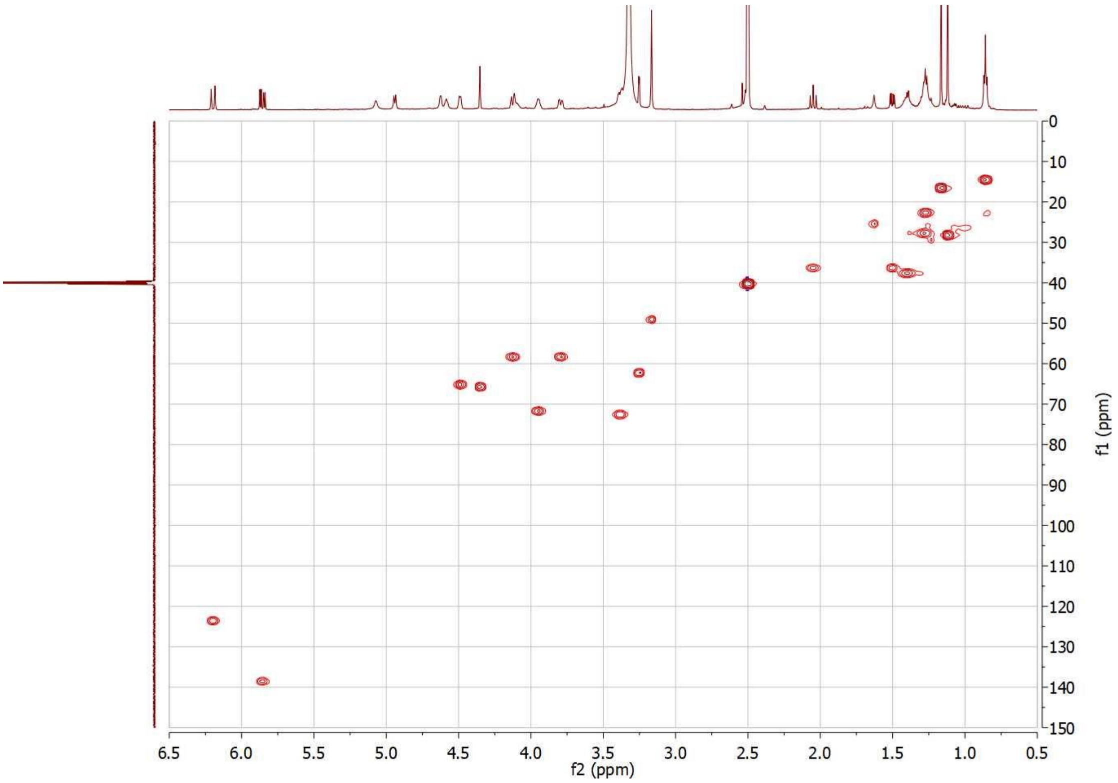


**Figure S113.** The HSQC (600 MHz, DMSO-$d_6$) spectrum of compound **20**.

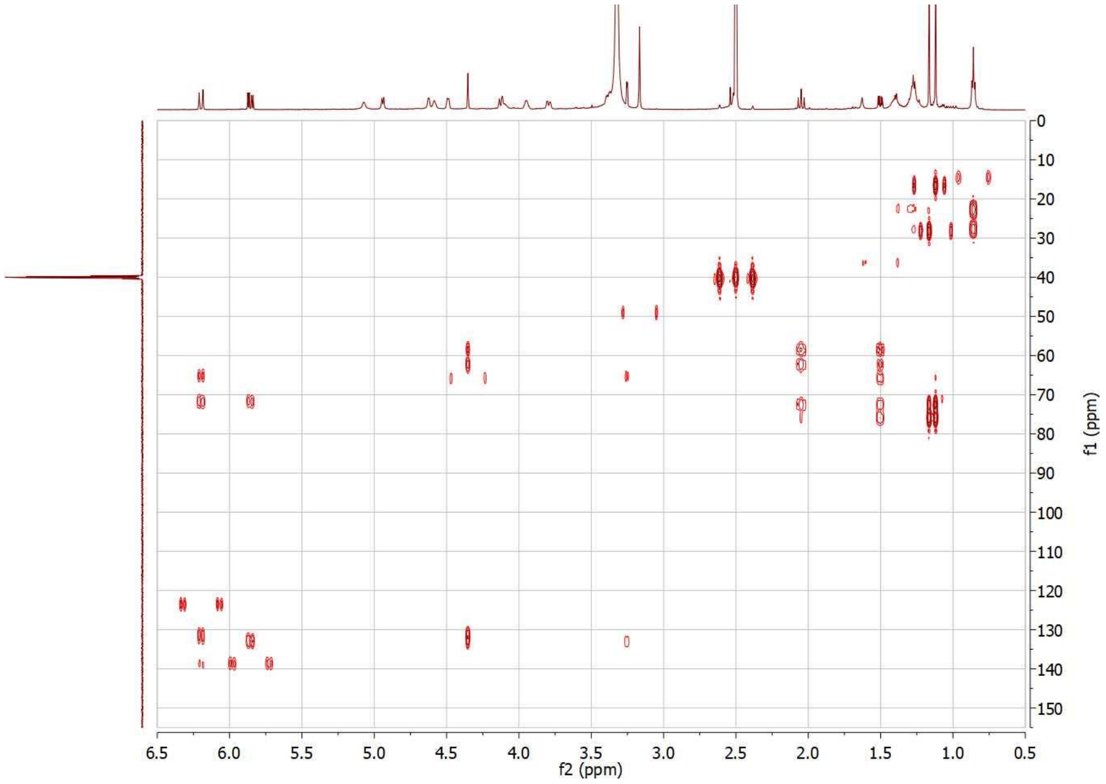


**Figure S114.** The HMBC (600 MHz, DMSO-$d_6$) spectrum of compound **20**.

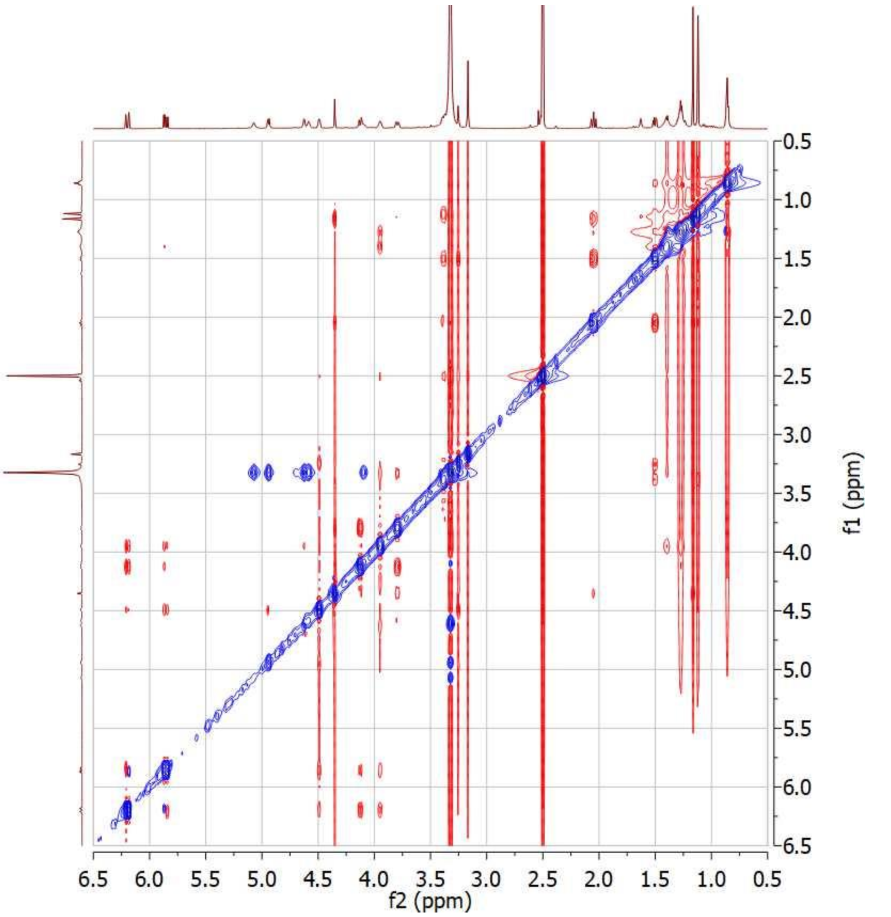


**Figure S115.** The ROESY ((600 MHz, DMSO-$d_6$) spectrum of compound **20**.

**Acquisition Parameter**

| | | | | | |
|---|---|---|---|---|---|
| Source Type | ESI | Ion Polarity | Positive | Set Nebulizer | 0.3 Bar |
| Focus | Not active | Set Capillary | 4000 V | Set Dry Heater | 180 °C |
| Scan Begin | 50 m/z | Set End Plate Offset | -500 V | Set Dry Gas | 4.0 l/min |
| Scan End | 1500 m/z | Set Collision Cell RF | 600.0 Vpp | Set Divert Valve | Source |

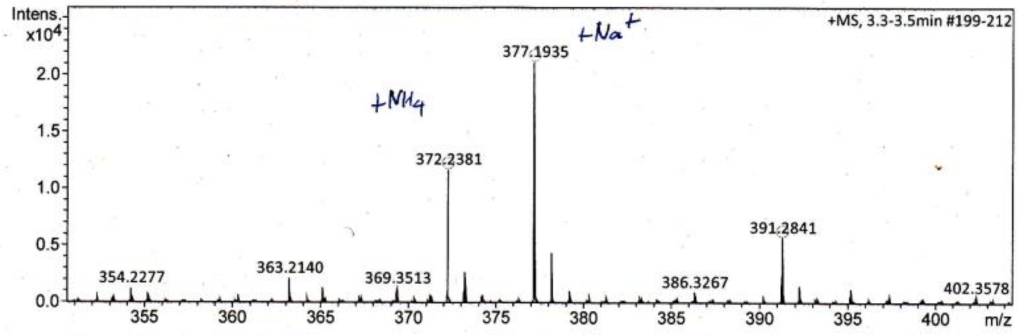


| Meas. m/z | # | Ion Formula | m/z | err [ppm] | mSigma | # mSigma | Score | rdb | e⁻ Conf | N-Rule |
|---|---|---|---|---|---|---|---|---|---|---|
| 372.2381 | 1 | C20H30N5O2 | 372.2394 | 3.6 | 16.0 | 1 | 51.00 | 8.5 | even | ok |
| | 2 | C19H34NO6 | 372.2381 | 0.0 | 16.7 | 2 | 100.00 | 3.5 | even | ok |
| | 3 | C18H31N5NaO2 | 372.2370 | -2.9 | 20.0 | 3 | 55.33 | 5.5 | even | ok |
| | 4 | C16H26N11 | 372.2367 | -3.6 | 22.5 | 4 | 44.20 | 9.5 | even | ok |
| 377.1935 | 1 | C19H30NaO6 | 377.1935 | -0.1 | 9.8 | 1 | 100.00 | 4.5 | even | ok |
| | 2 | C16H22N10Na | 377.1921 | -3.7 | 16.7 | 2 | 43.32 | 10.5 | even | ok |
| | 3 | C20H26N4NaO2 | 377.1948 | 3.4 | 19.6 | 3 | 43.24 | 9.5 | even | ok |

**Figure S116.** The HREISMS of compound **21**.

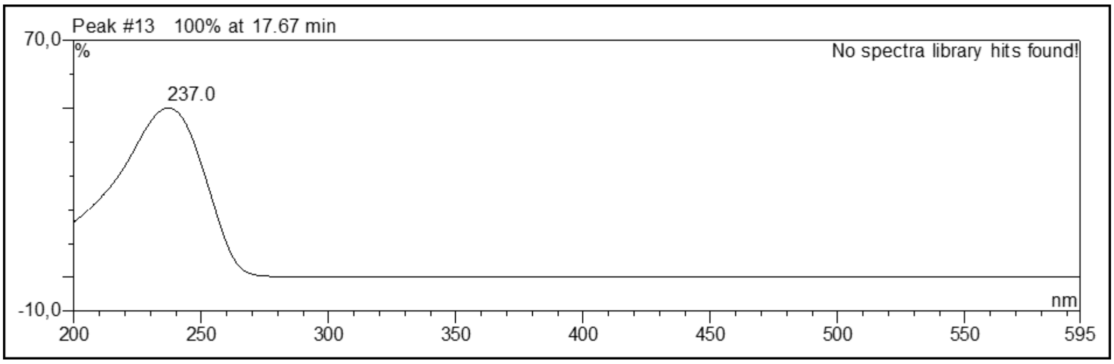


**Figure S117.** The UV spectrum of compound **21**.

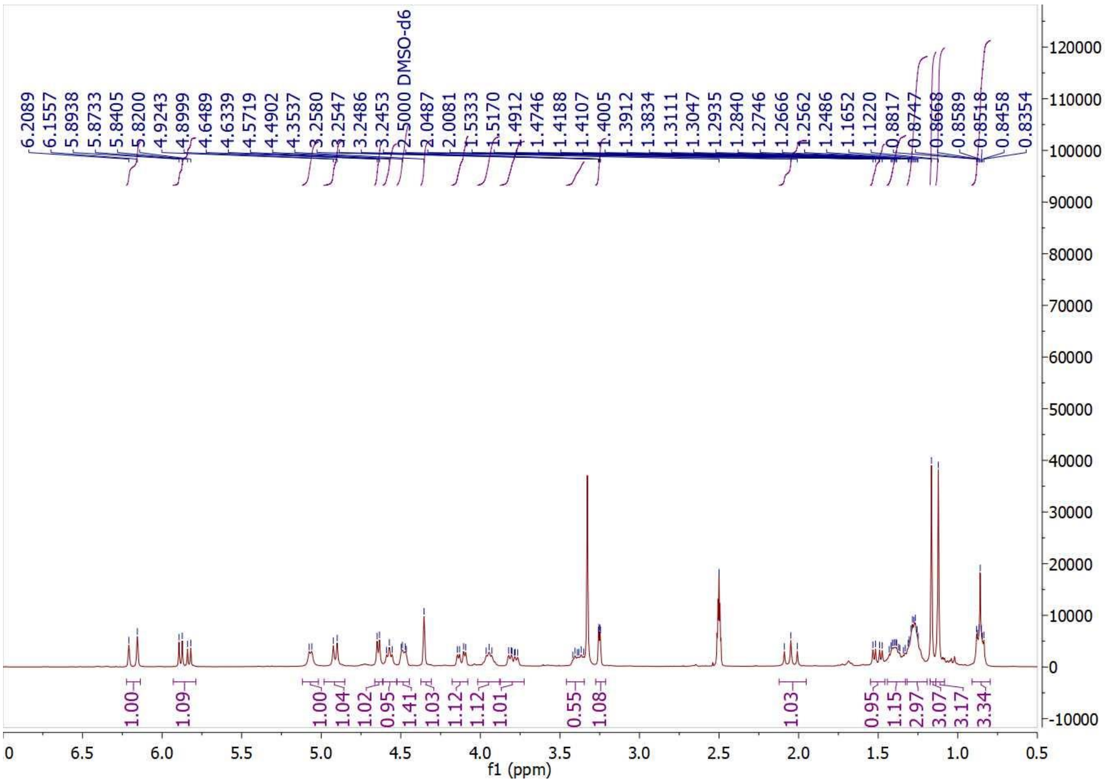

**Figure S118**. The $^{1}$H-NMR (300 MHz, DMSO-$d_6$) spectrum of compound **21.**

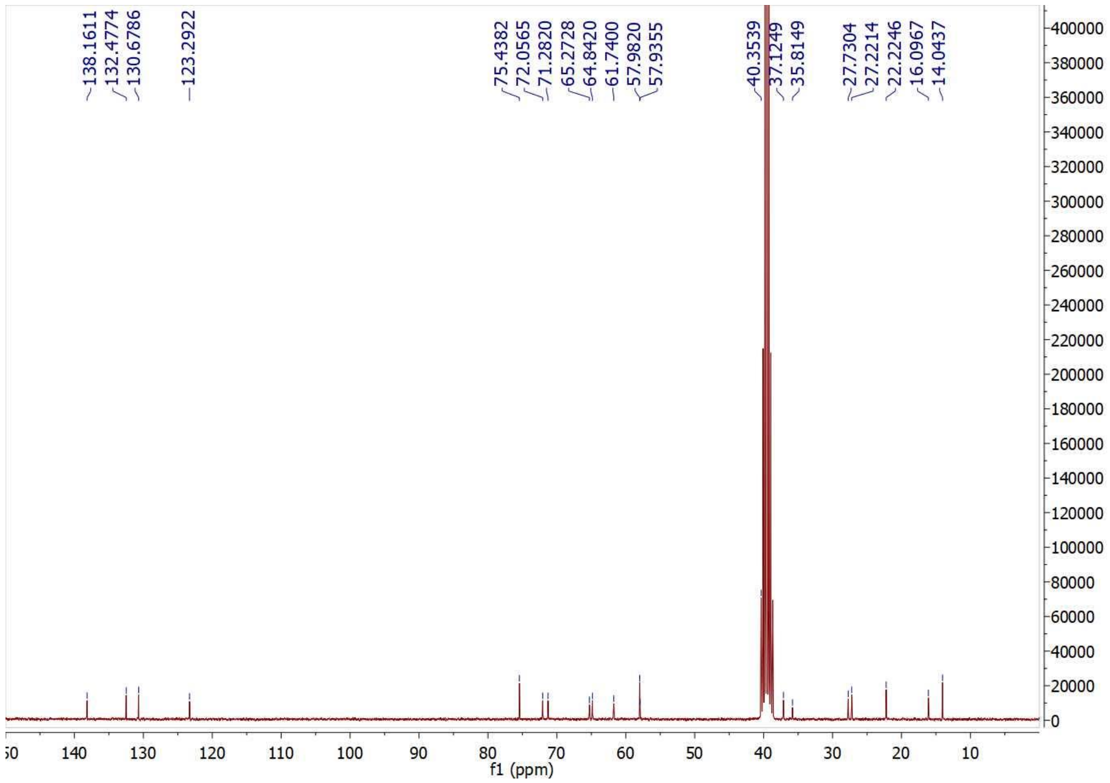


**Figure S119.** The $^{13}$C-NMR (75 MHz, DMSO-$d_6$) spectrum of compound **21**.

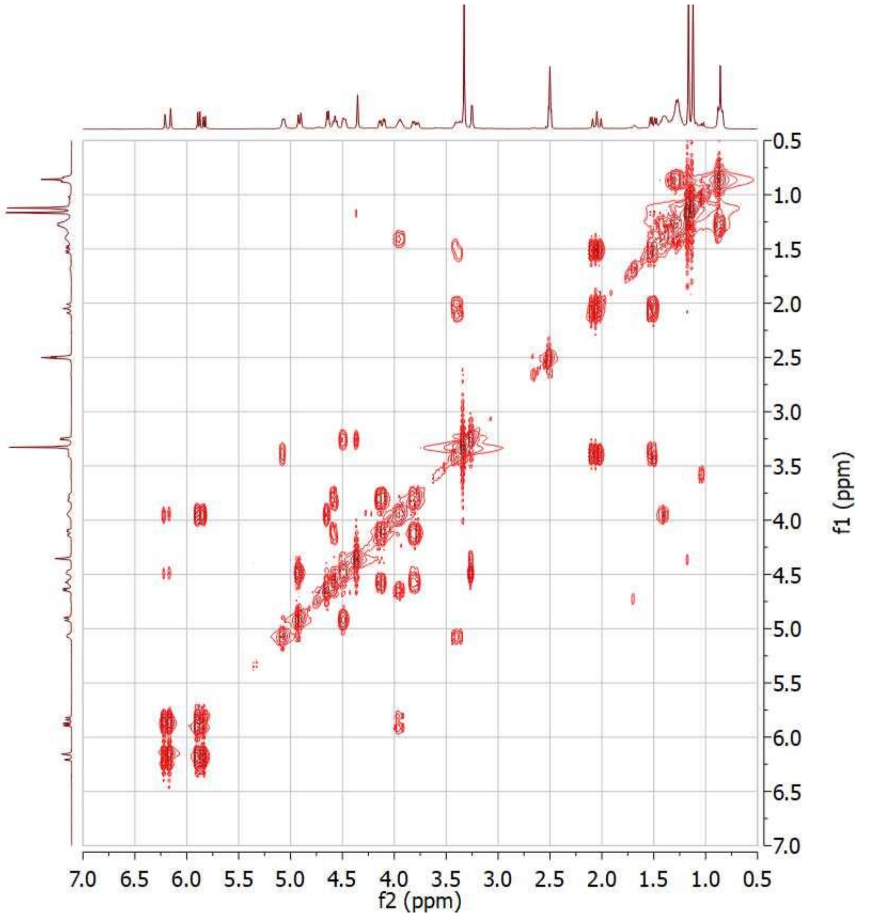


**Figure S120.** The $^{1}$H-$^{1}$H COSY (300 MHz, DMSO-$d_6$) spectrum of compound **21**.

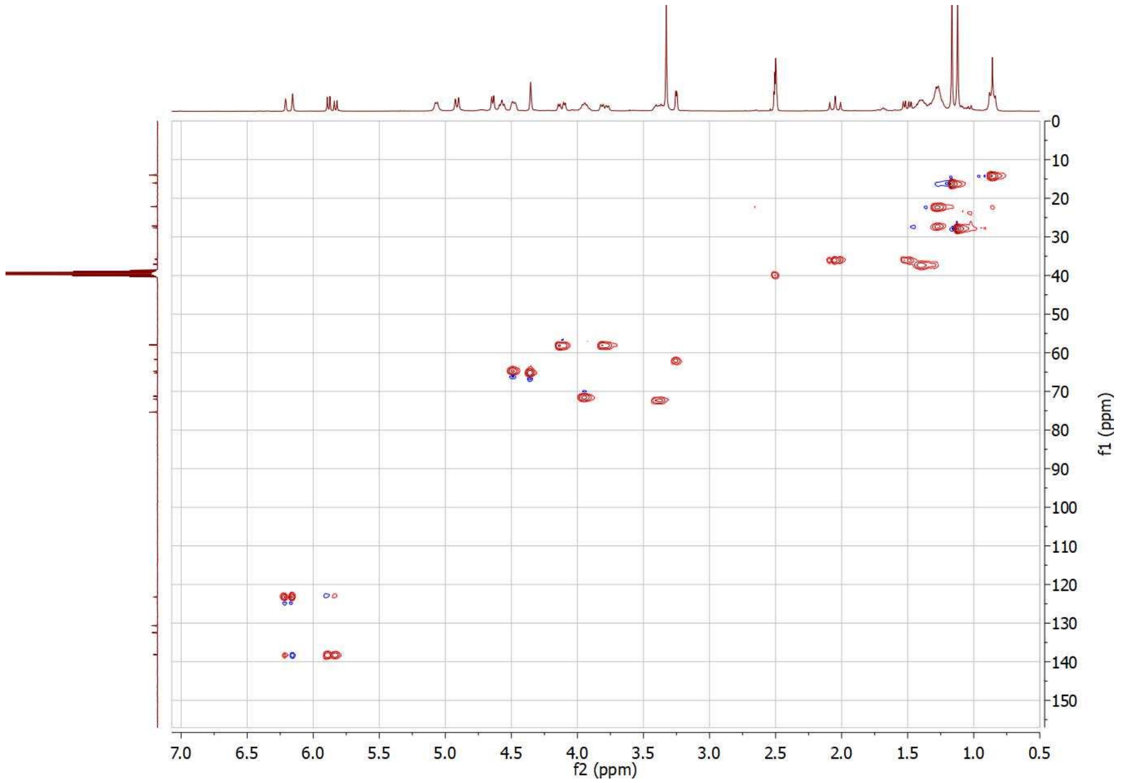


**Figure S121.** The HSQC (300 MHz, DMSO-$d_6$) spectrum of compound **21**.

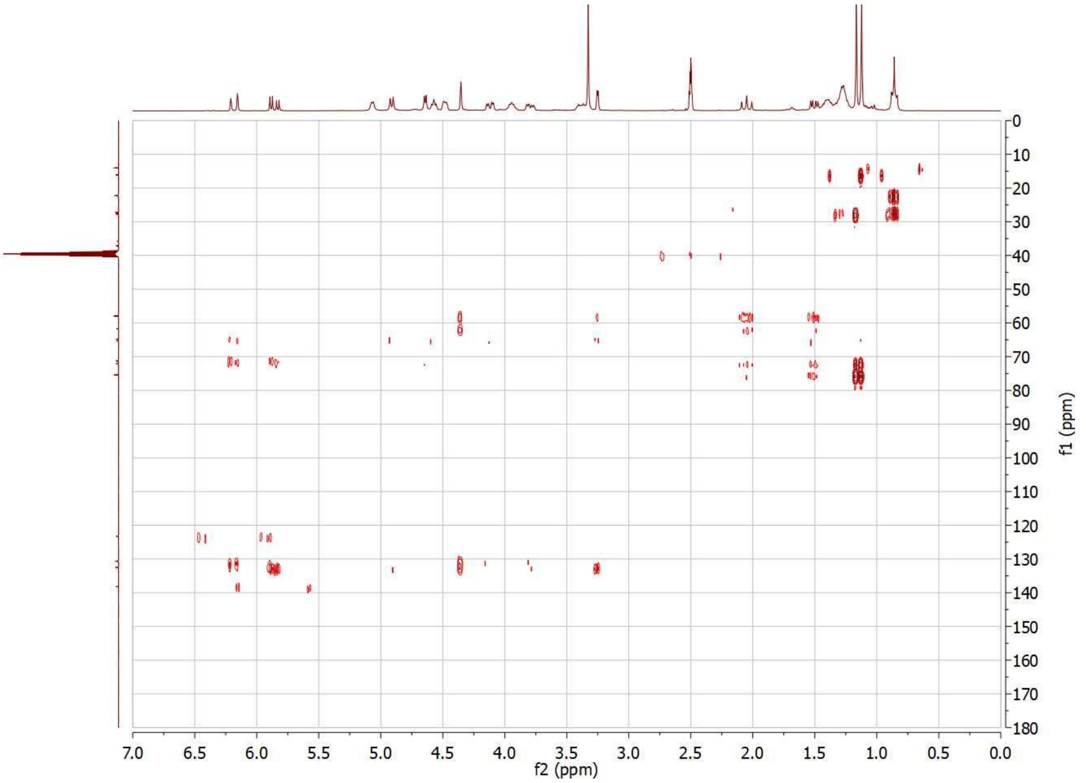


**Figure S122.** The HMBC (300 MHz, DMSO-$d_6$) spectrum of compound **21**.

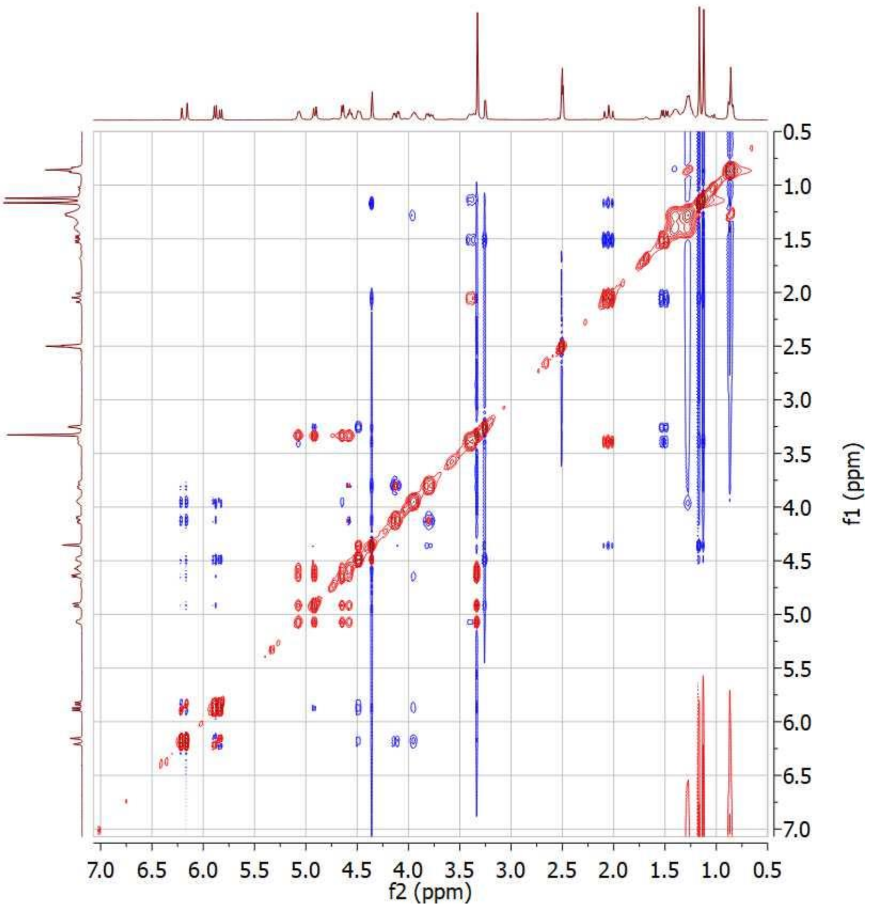


**Figure S123.** The ROESY (300 MHz, DMSO-$d_6$) spectrum of compound **21**.

**Acquisition Parameter**

| | | | | | |
|---|---|---|---|---|---|
| Source Type | ESI | Ion Polarity | Positive | Set Nebulizer | 0.3 Bar |
| Focus | Not active | Set Capillary | 4000 V | Set Dry Heater | 180 °C |
| Scan Begin | 50 m/z | Set End Plate Offset | -500 V | Set Dry Gas | 4.0 l/min |
| Scan End | 1500 m/z | Set Collision Cell RF | 600.0 Vpp | Set Divert Valve | Source |

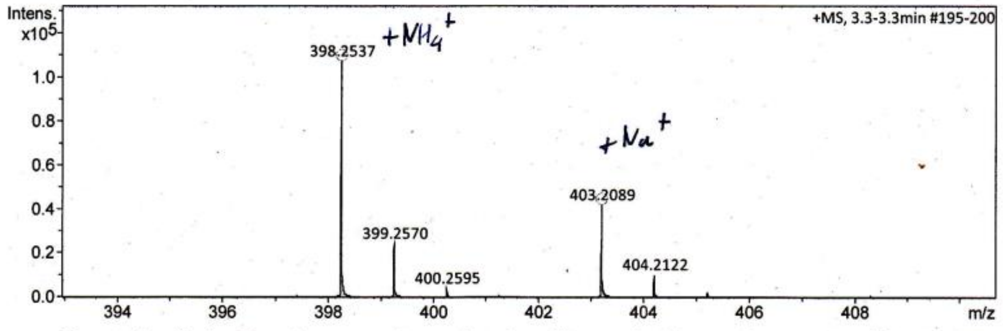


| Meas. m/z | # | Ion Formula | m/z | err [ppm] | mSigma | # mSigma | Score | rdb | e⁻ Conf | N-Rule |
|---|---|---|---|---|---|---|---|---|---|---|
| 398.2537 | 1 | C21H36NO6 | 398.2537 | 0.1 | 6.5 | 1 | 100.00 | 4.5 | even | ok |
| 403.2089 | 1 | C21H32NaO6 | 403.2091 | 0.4 | 3.6 | 1 | 100.00 | 5.5 | even | ok |

**Figure S124.** The HREISMS of compound **22**.

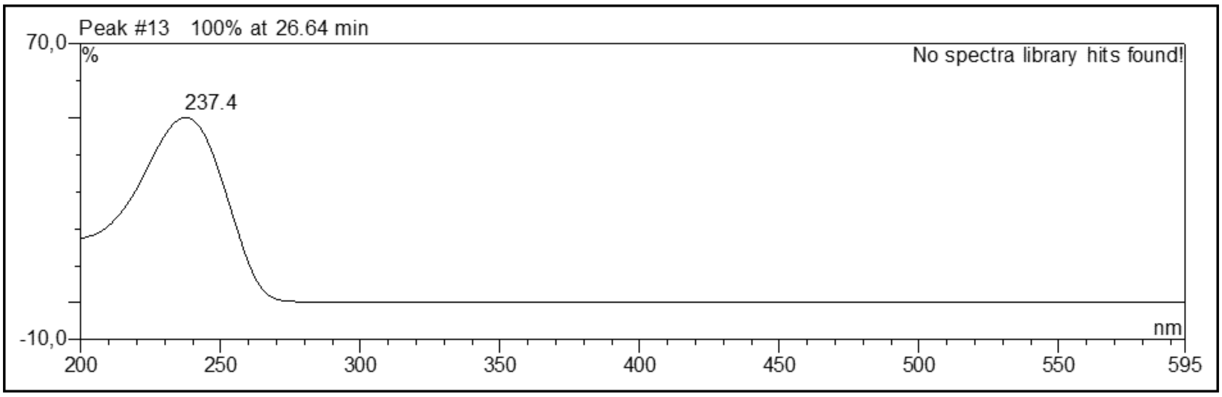


**Figure S125.** The UV spectrum of compound **22**.

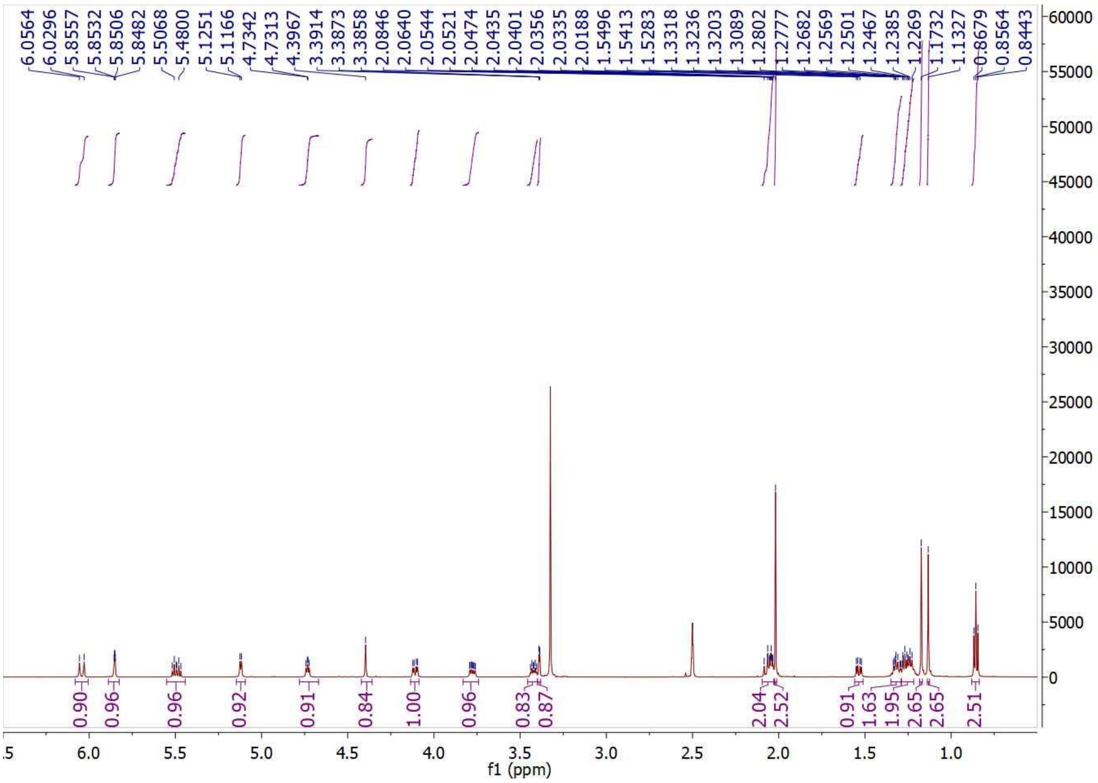


**Figure S126.** The $^1$H-NMR (600 MHz, DMSO-$d_6$) spectrum of compound **22**.

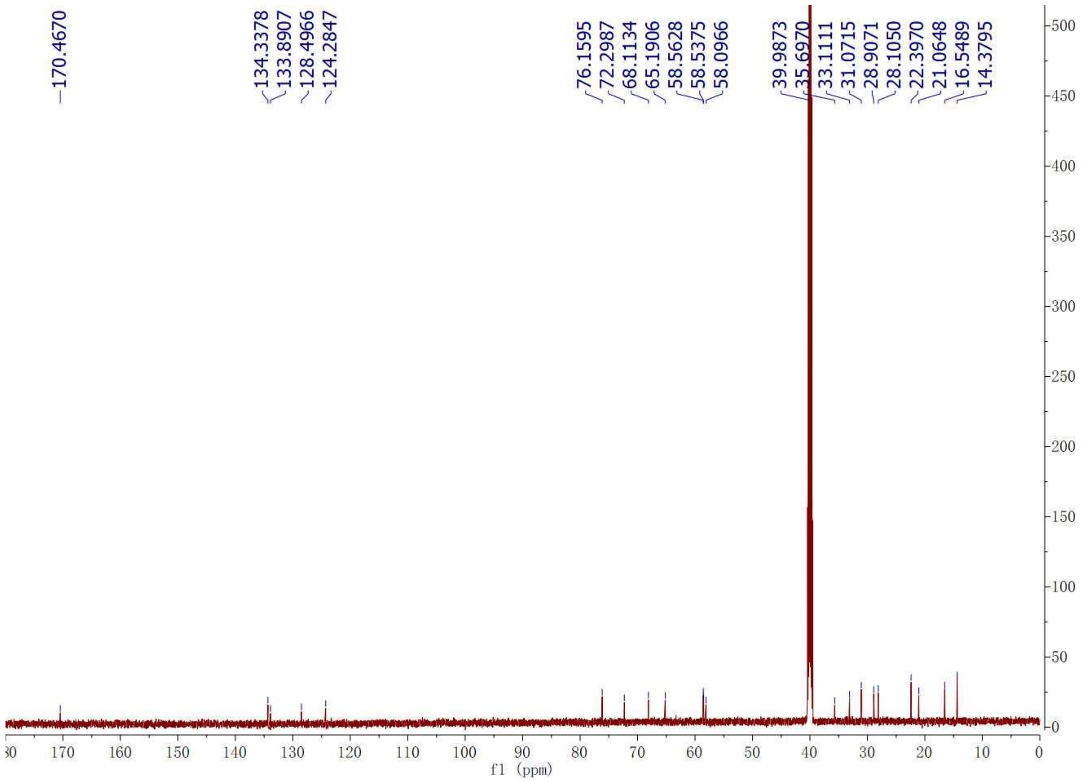


**Figure S127**. The $^{13}$C-NMR (150 MHz, DMSO-$d_6$) spectrum of compound **22**.

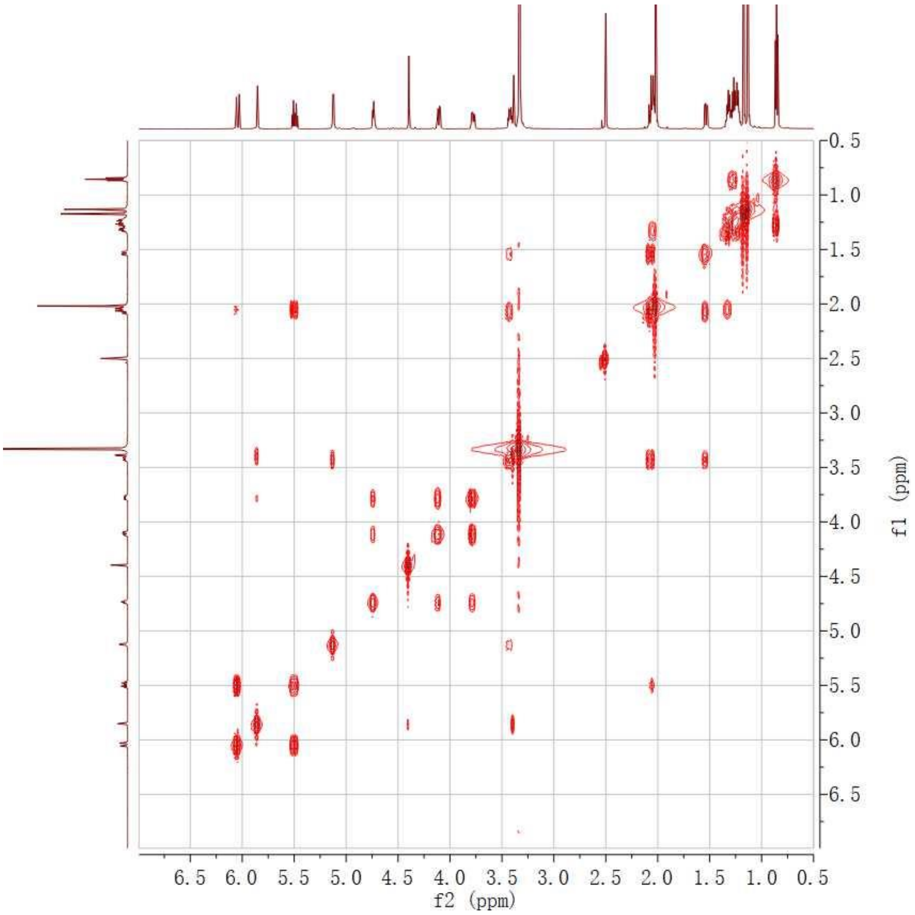


**Figure S128.** The $^{1}$H-$^{1}$H COSY (600 MHz, DMSO-$d_6$) spectrum of compound **22**.

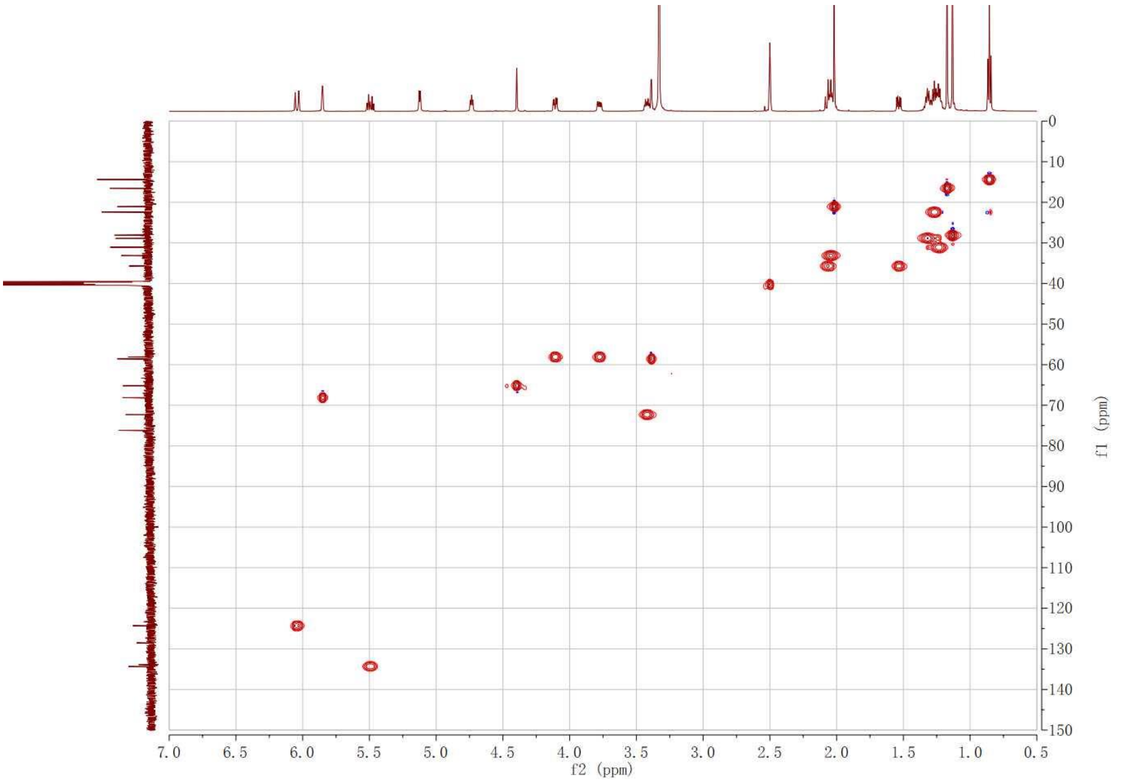


**Figure S129.** The HSQC (600 MHz, DMSO-$d_6$) spectrum of compound **22**.

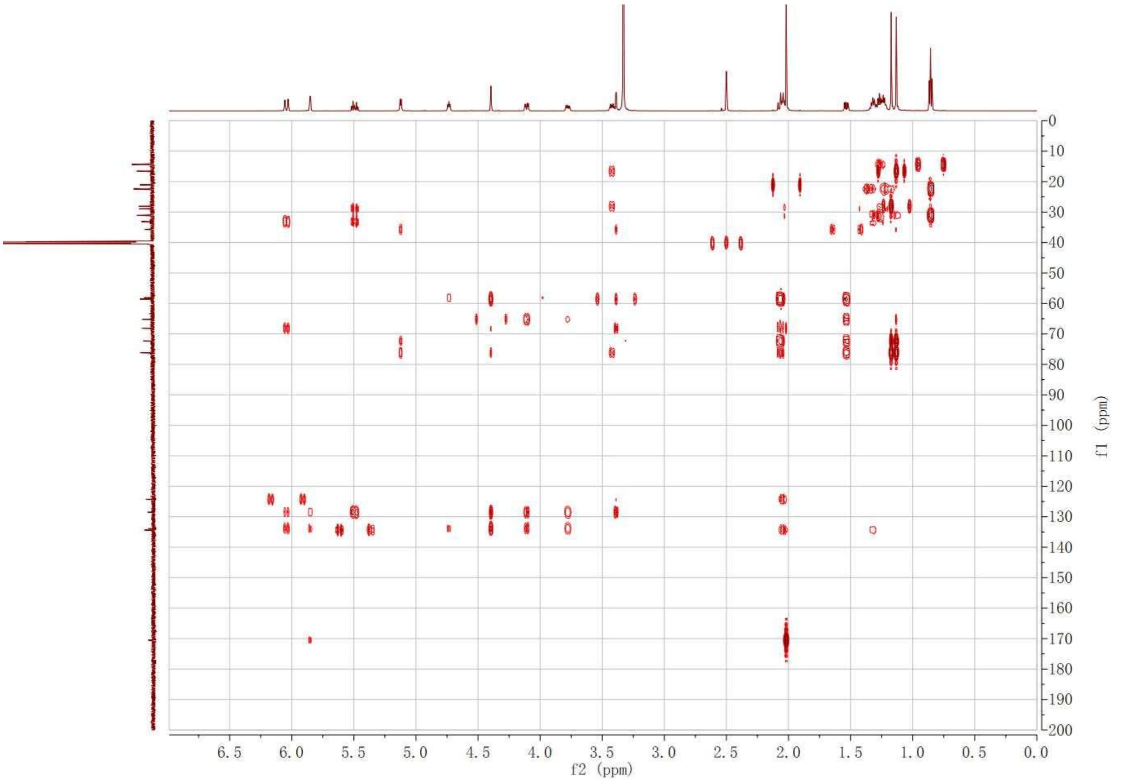


**Figure S130.** The HMBC (600 MHz, DMSO-$d_6$) spectrum of compound **22**.

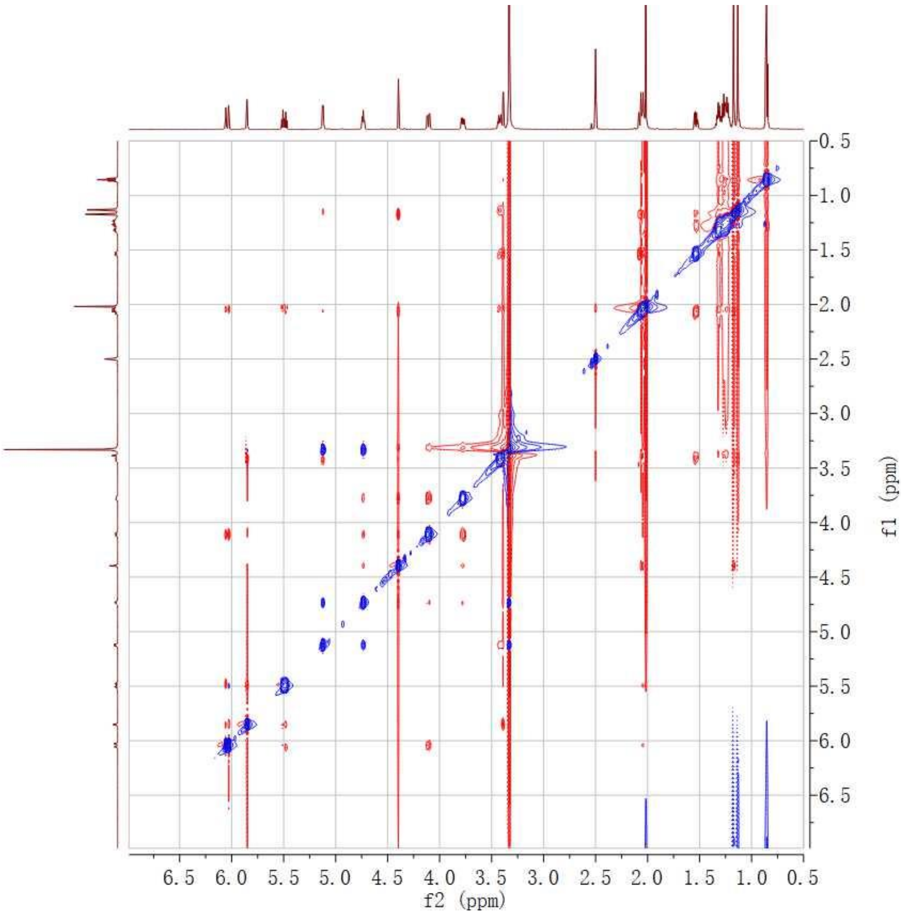


**Figure S131.** The ROESY (600 MHz, DMSO-$d_6$) spectrum of compound **22**.

**Acquisition Parameter**

| | | | | | |
|---|---|---|---|---|---|
| Source Type | ESI | Ion Polarity | Positive | Set Nebulizer | 0.3 Bar |
| Focus | Not active | Set Capillary | 4000 V | Set Dry Heater | 180 °C |
| Scan Begin | 50 m/z | Set End Plate Offset | -500 V | Set Dry Gas | 4.0 l/min |
| Scan End | 1500 m/z | Set Collision Cell RF | 600.0 Vpp | Set Divert Valve | Source |

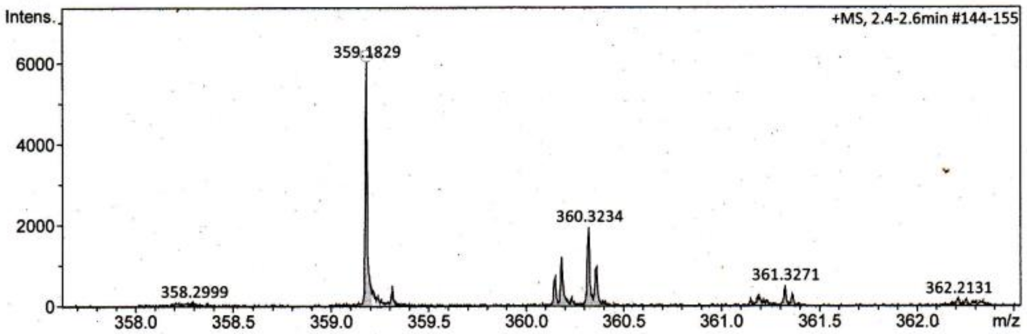


| Meas. m/z | # | Ion Formula | m/z | err [ppm] | mSigma | # mSigma | Score | rdb | e⁻ Conf | N-Rule |
|---|---|---|---|---|---|---|---|---|---|---|
| 359.1829 | 1 | C19H28NaO5 | 359.1829 | -0.1 | 8.2 | 1 | 100.00 | 5.5 | even | ok |

**Figure S132.** The HREISMS of compound **23**.

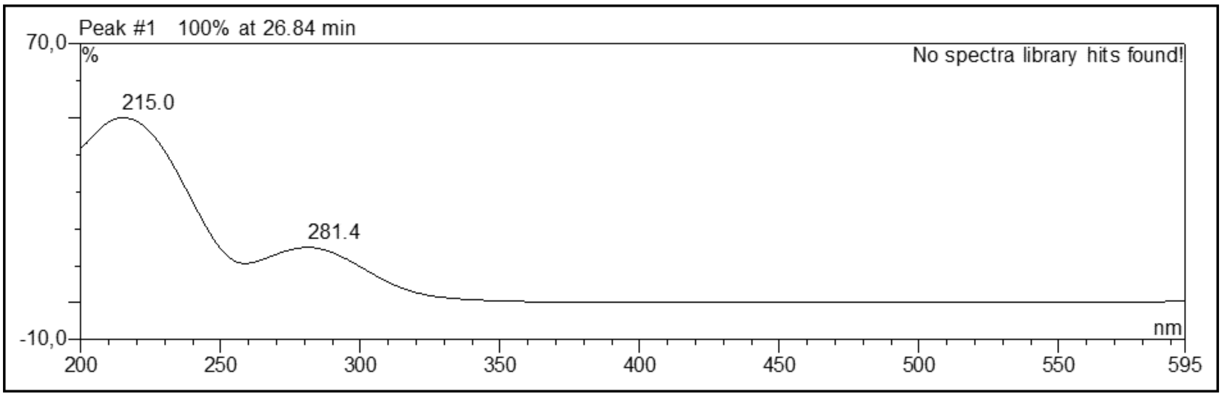


**Figure S133.** The UV spectrum of compound **23**.

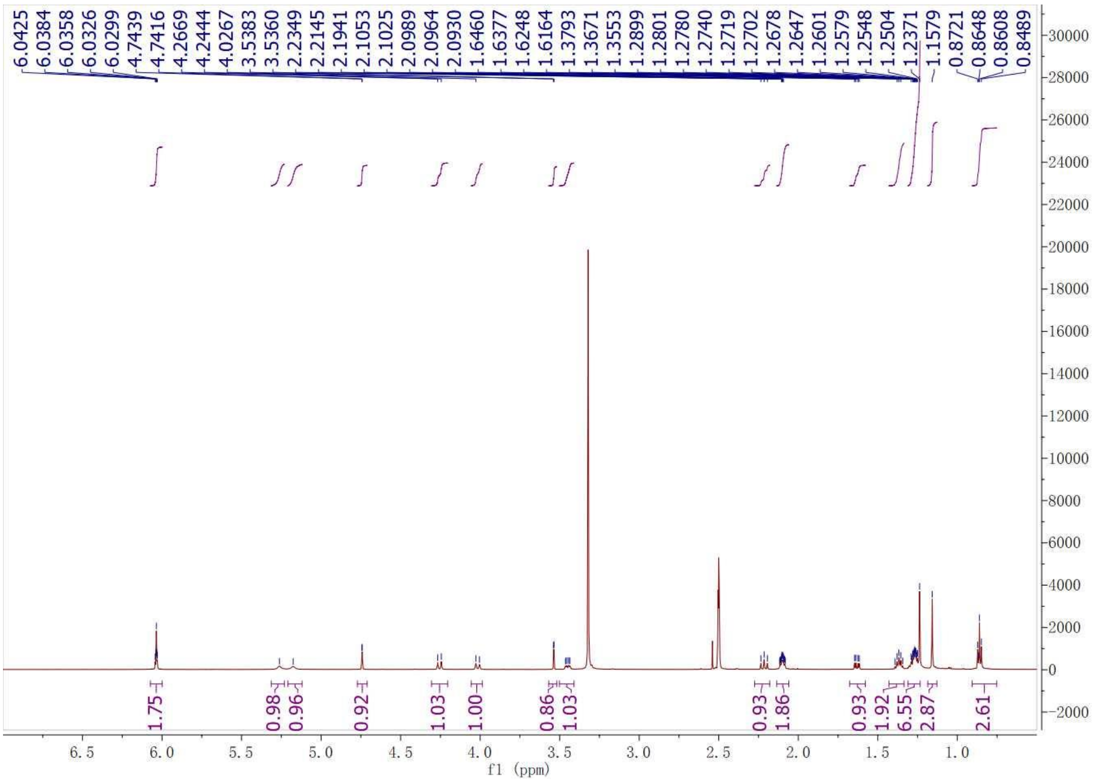


**Figure S134.** The $^{1}$H-NMR (600 MHz, DMSO-$d_6$) spectrum of compound **23**.

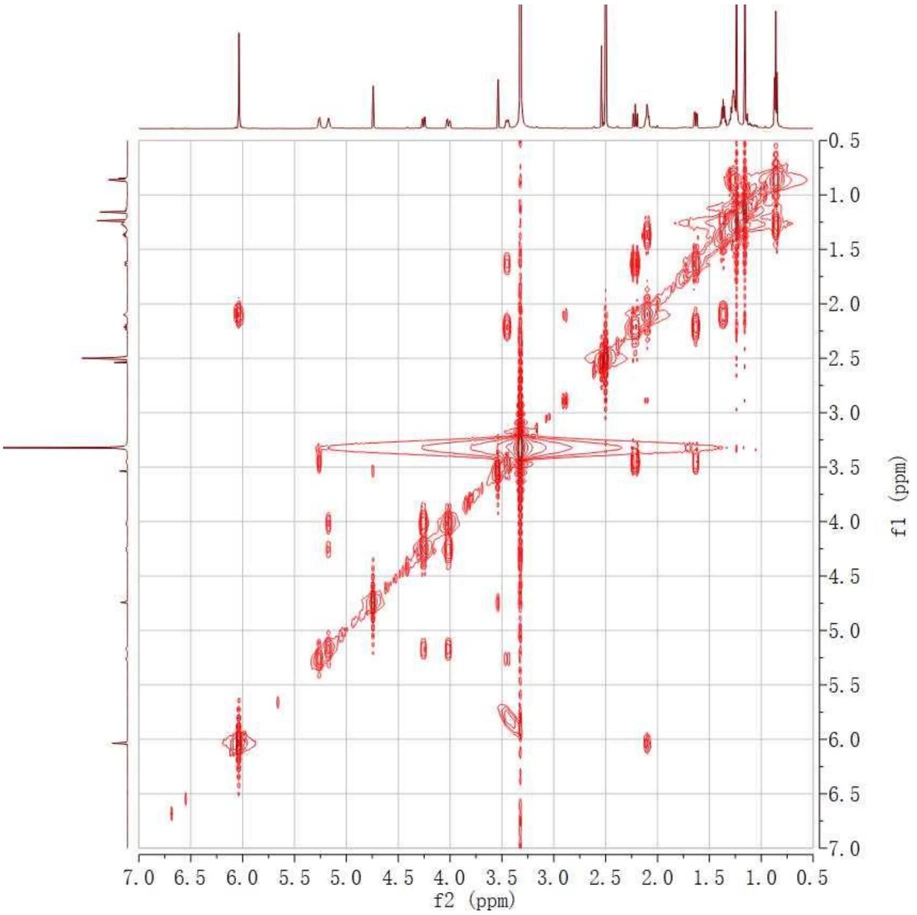


**Figure S135.** The $^{1}$H-$^{1}$H COSY (600 MHz, DMSO-$d_6$) spectrum of compound **23**.

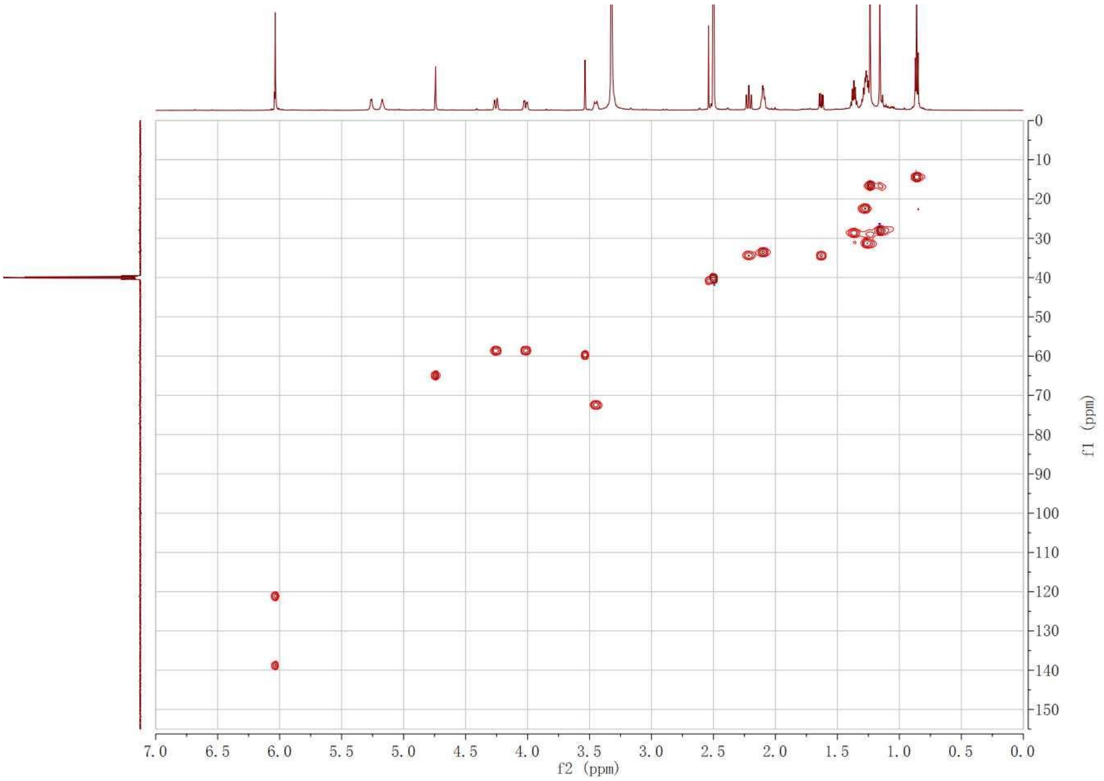


**Figure S136.** The HSQC (600 MHz, DMSO-$d_6$) spectrum of compound **23**.

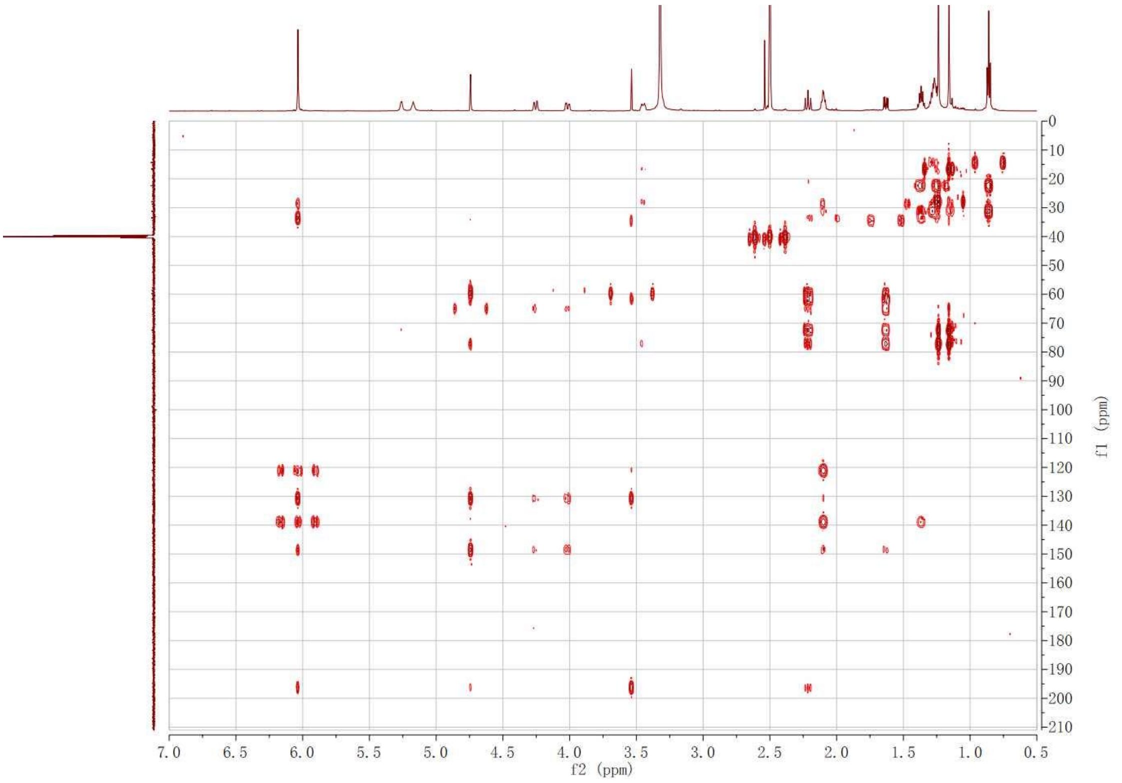


**Figure S137.** The HMBC (600 MHz, DMSO-$d_6$) spectrum of compound **23**.

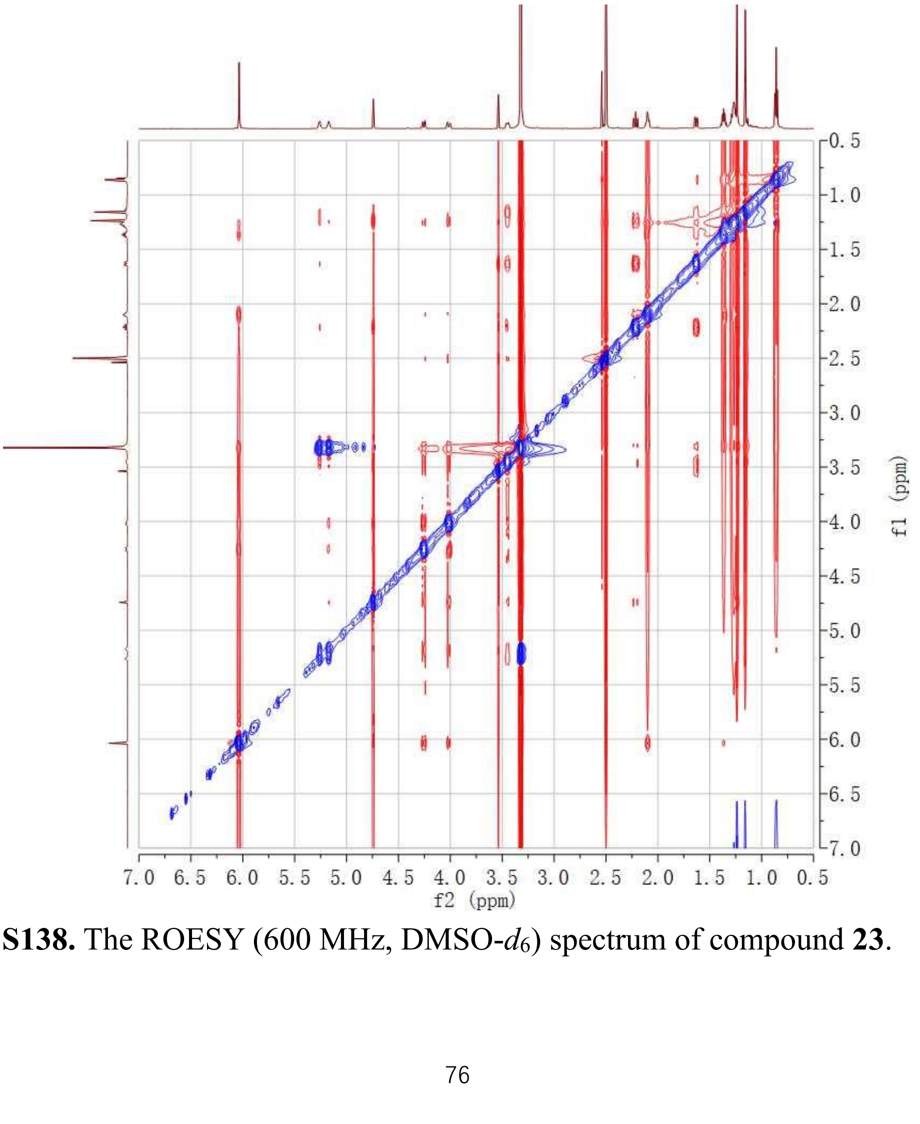


**Figure S138.** The ROESY (600 MHz, DMSO-$d_6$) spectrum of compound **23**.

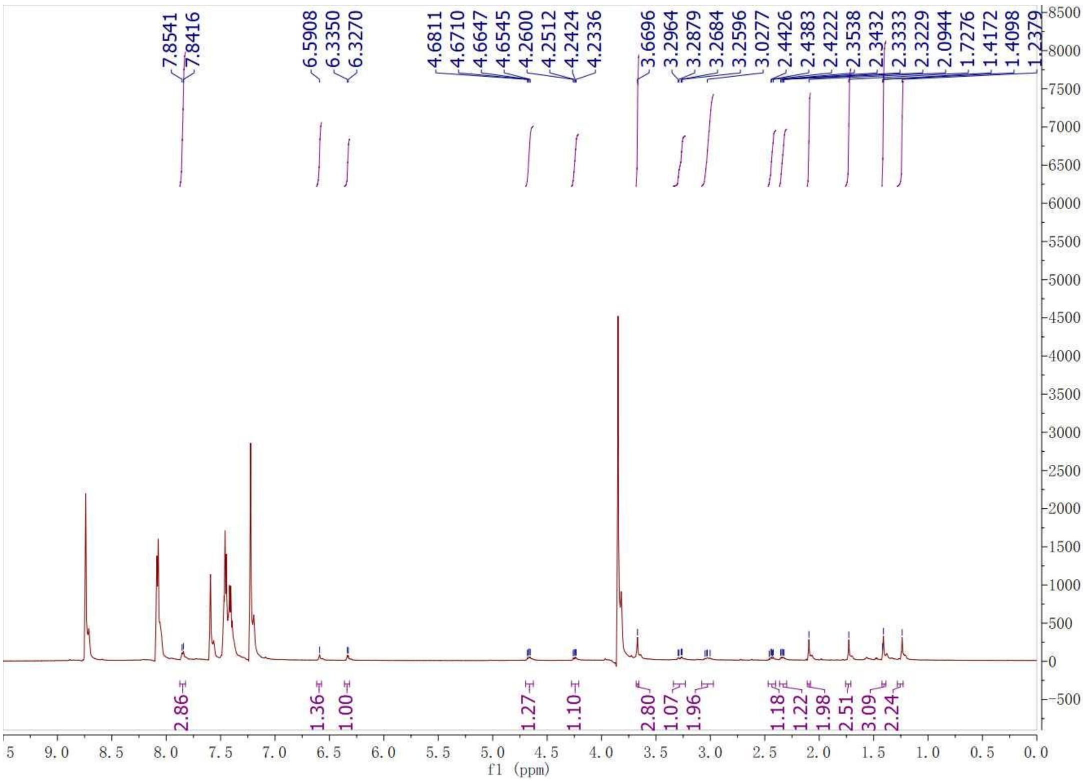


**Figure S139.** The $^{1}$H-NMR (600 MHz, Prydine-$d_5$) spectrum of **1a**.

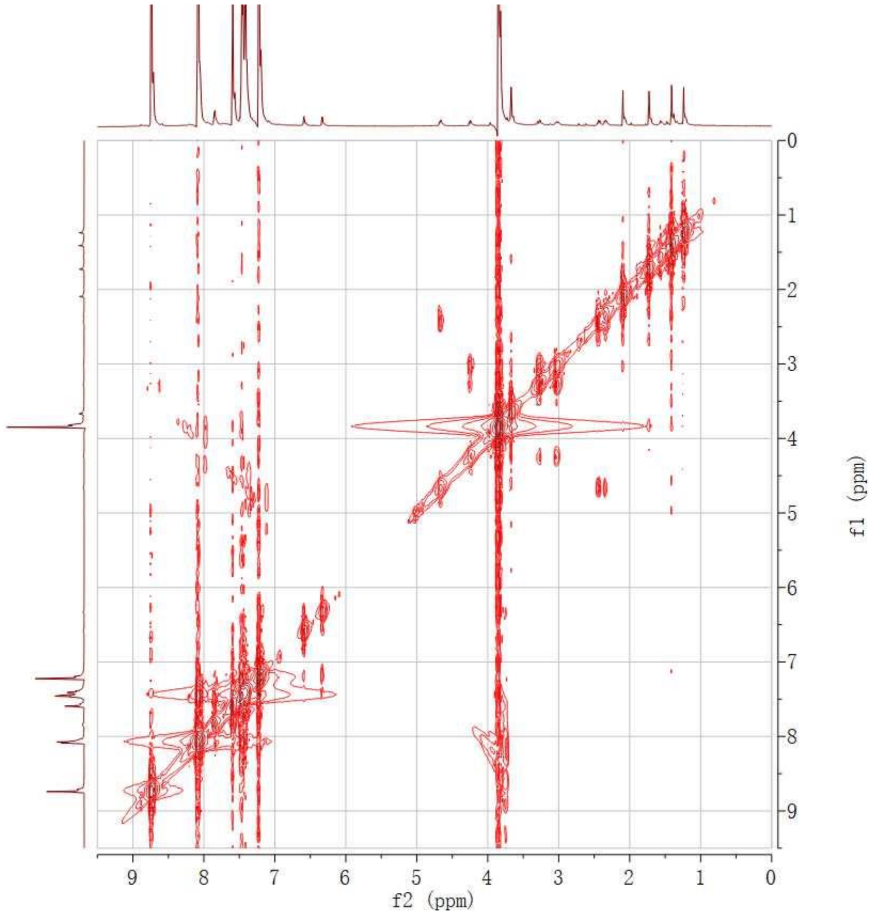


**Figure S140.** The $^{1}$H-$^{1}$H COSY (600 MHz, Prydine-$d_5$) spectrum of **1a**.

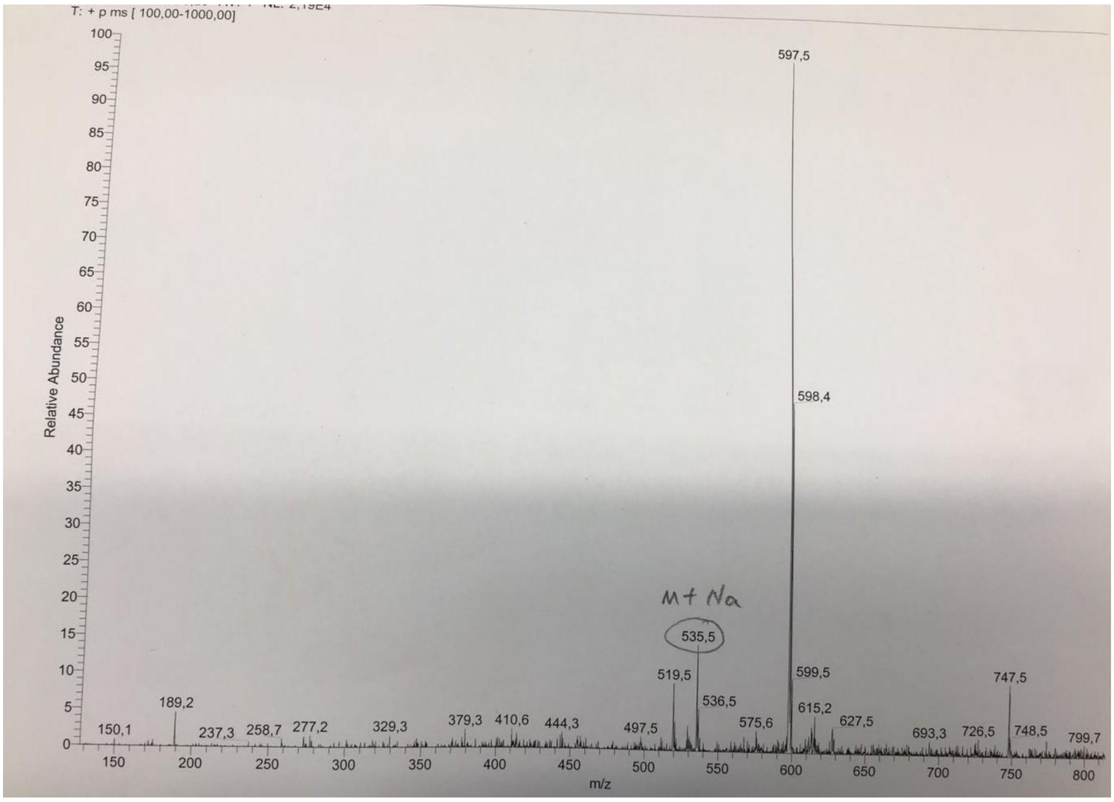


**Figure S141.** The ESIMS of **1a**.

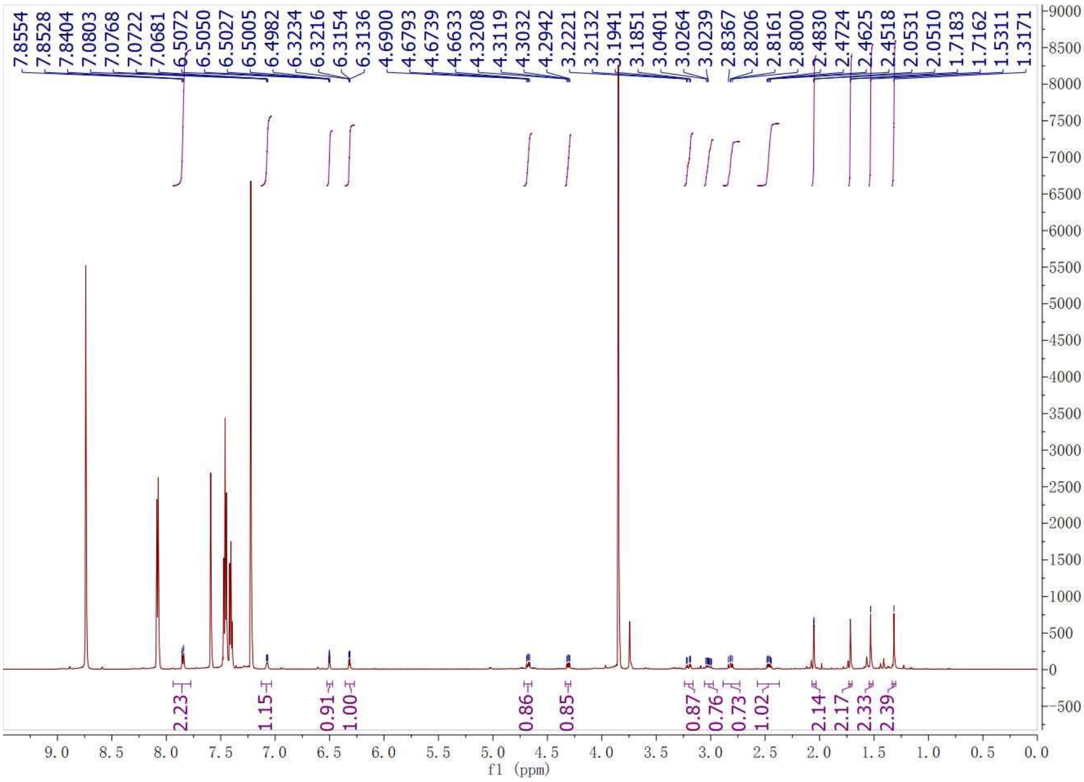


**Figure S142.** The $^{1}$H-NMR (600 MHz, Prydine-$d_5$) spectrum of **1b**.

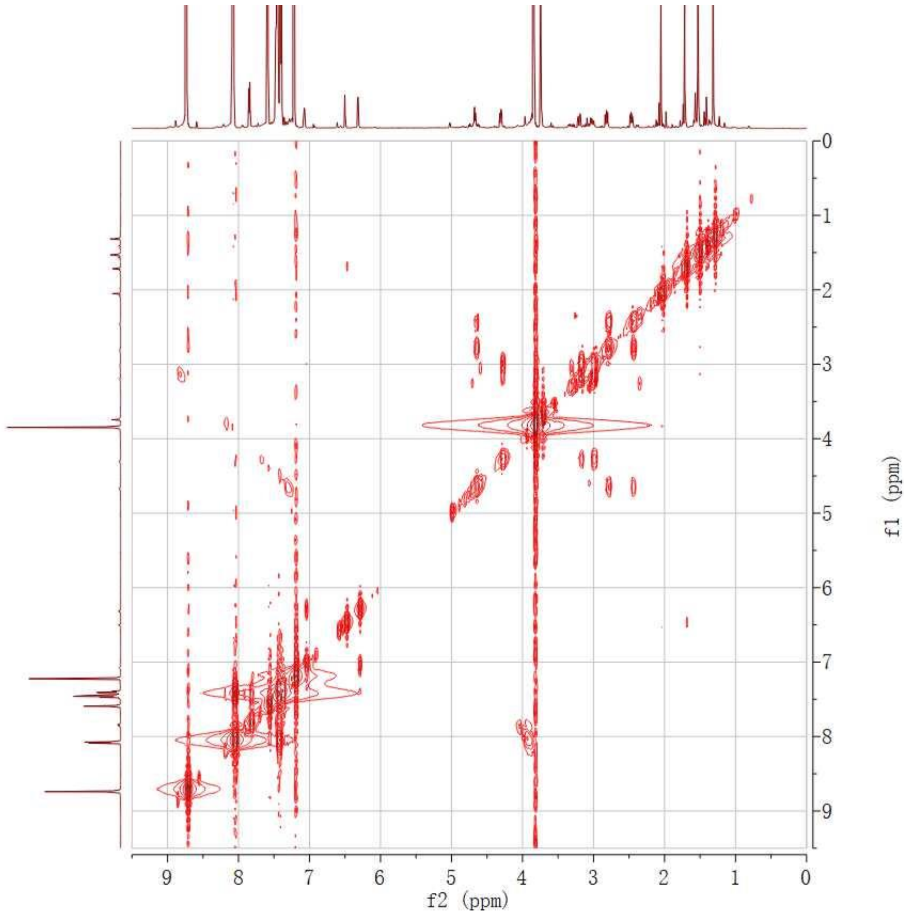


**Figure S143.** The $^{1}$H-$^{1}$H COSY (600 MHz, Prydine-$d_5$) spectrum of **1b**.

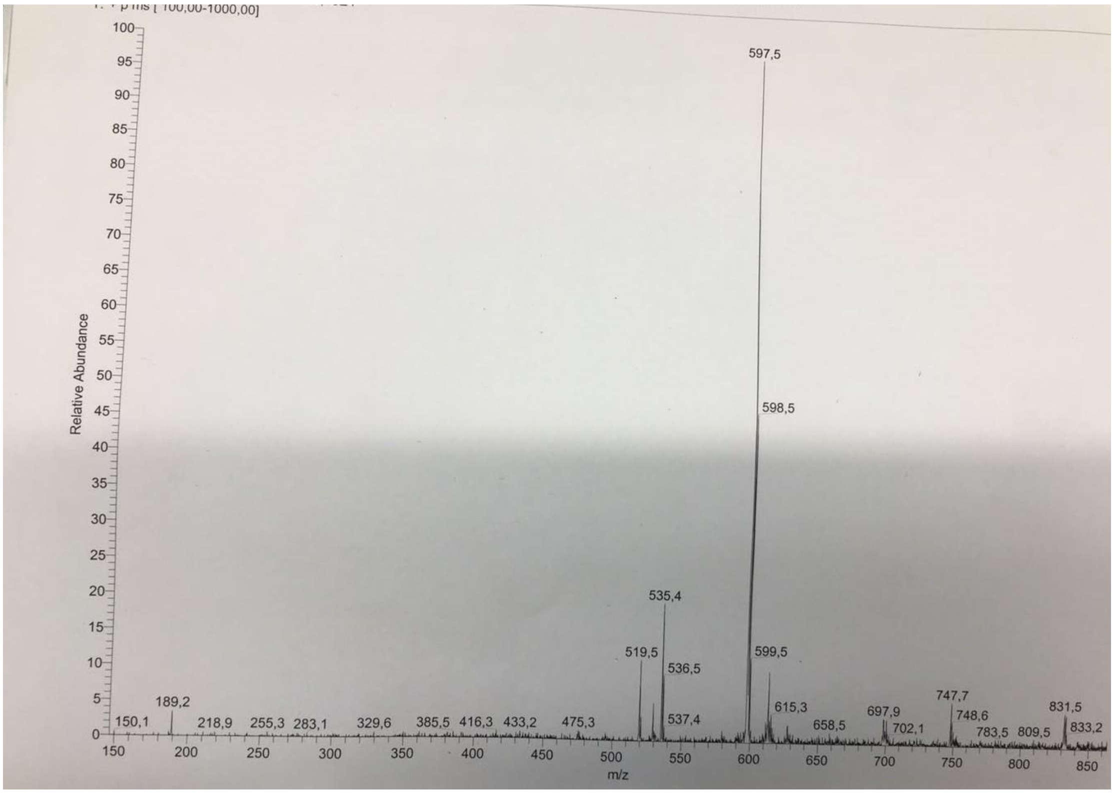


**Figure S144.** The ESIMS of **1b**.

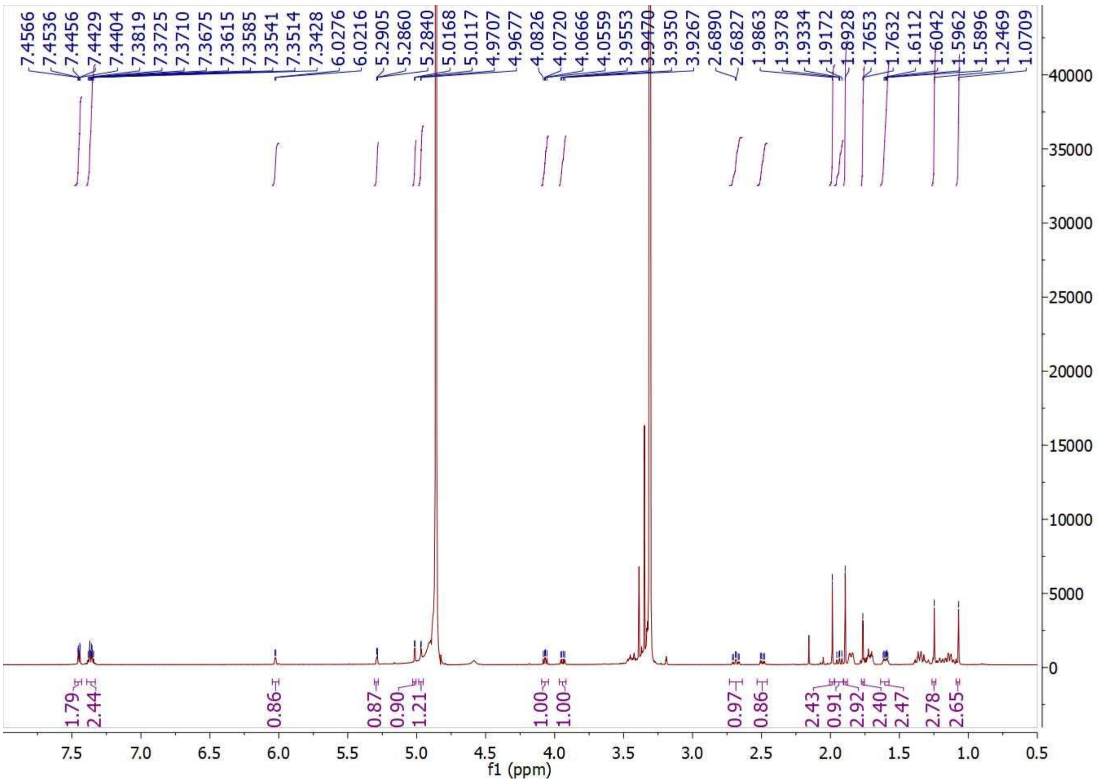

**Figure S145.** The $^{1}$H-NMR (600 MHz, $CD_3OD$) spectrum of **7a**.

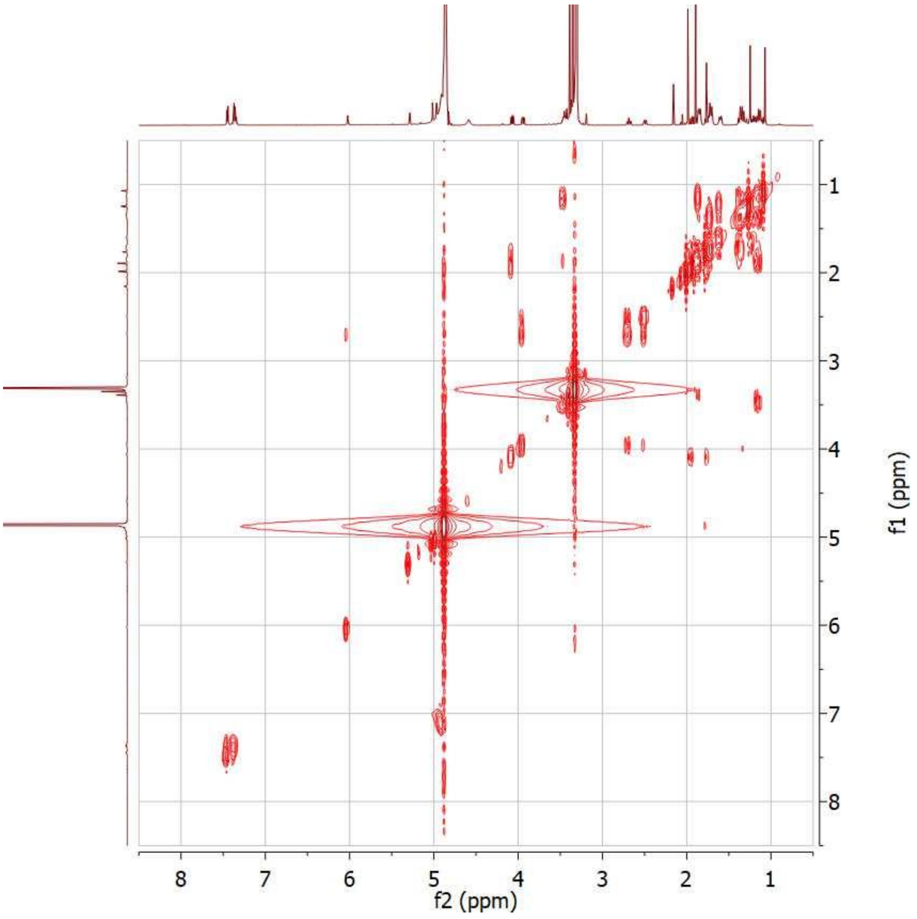

**Figure S146.** The $^{1}$H-$^{1}$H COSY (600 MHz, $CD_3OD$) spectrum of **7a**.

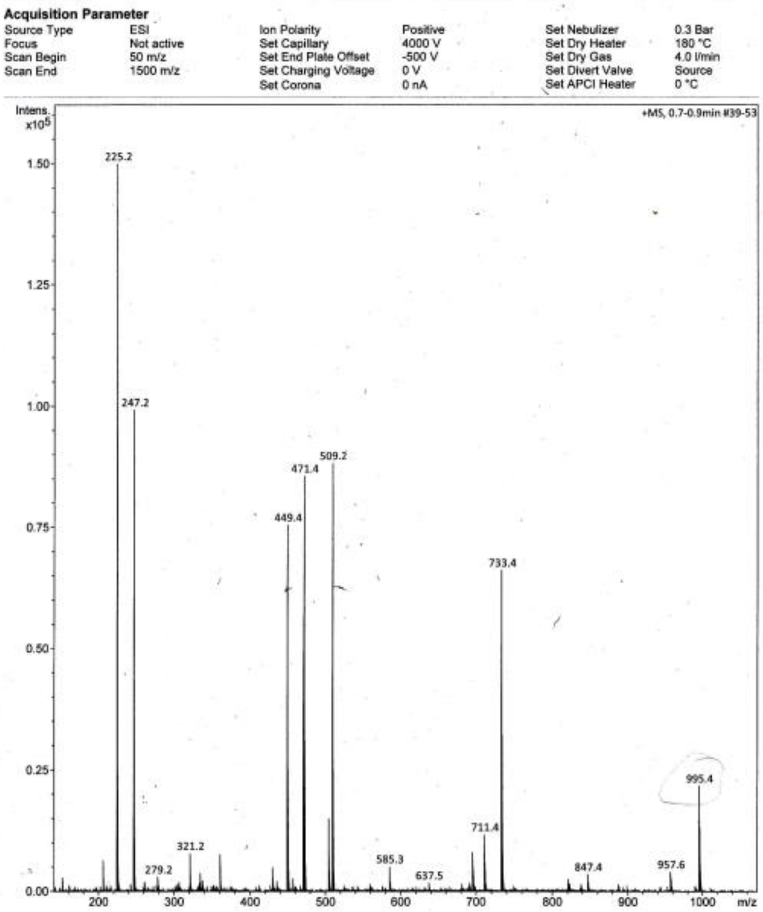


**Figure S147.** The ESIMS of **7a**.

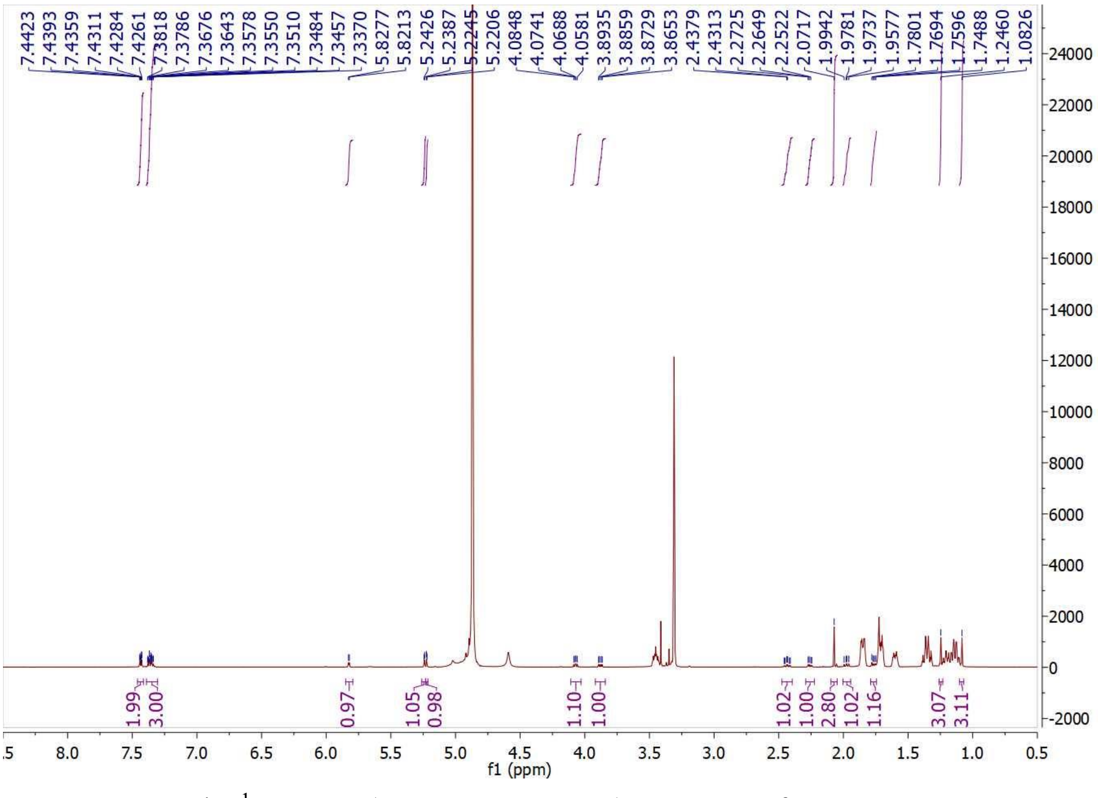


**Figure S148.** The $^{1}$H-NMR (600 MHz, $CD_3OD$) spectrum of **7b**.

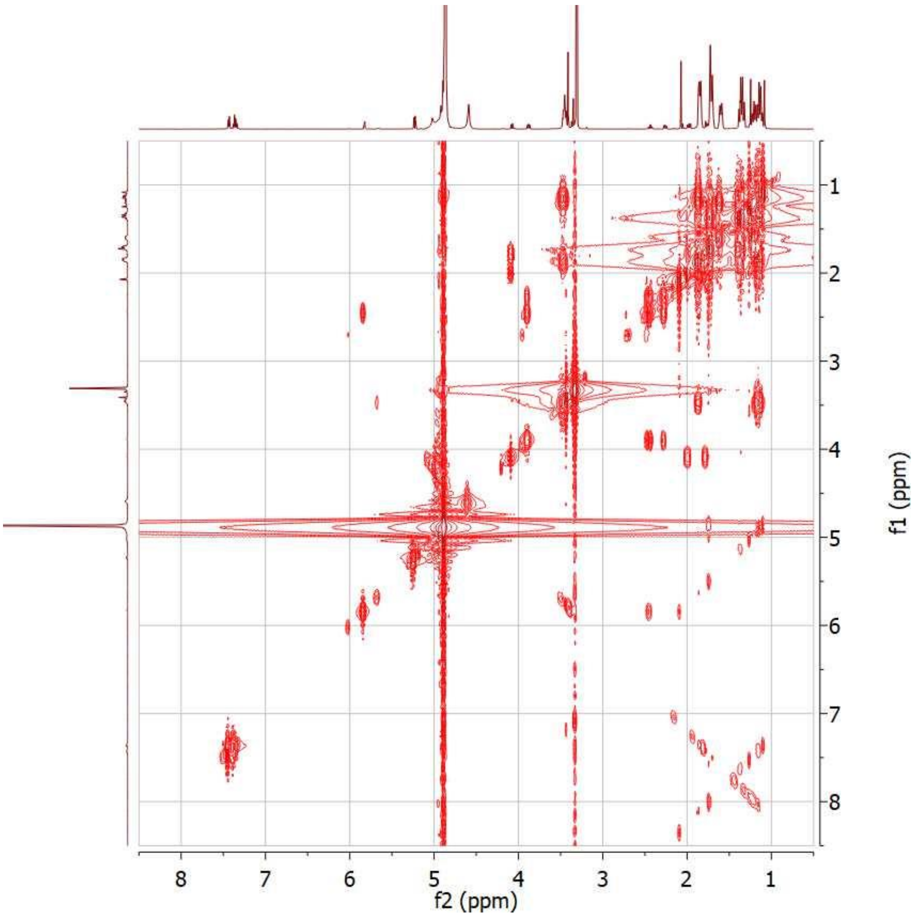


**Figure S149.** The $^{1}H$-$^{1}H$ COSY (600 MHz, $CD_3OD$) spectrum of **7b**.

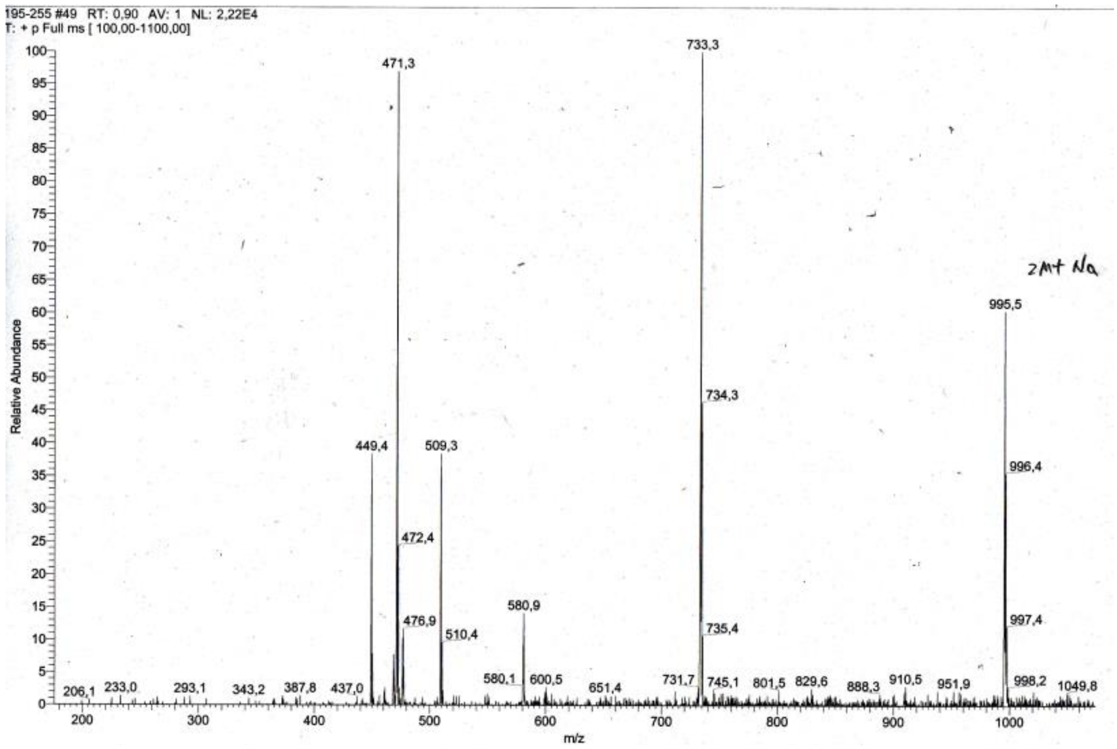


**Figure S150.** The ESIMS of **7b**.

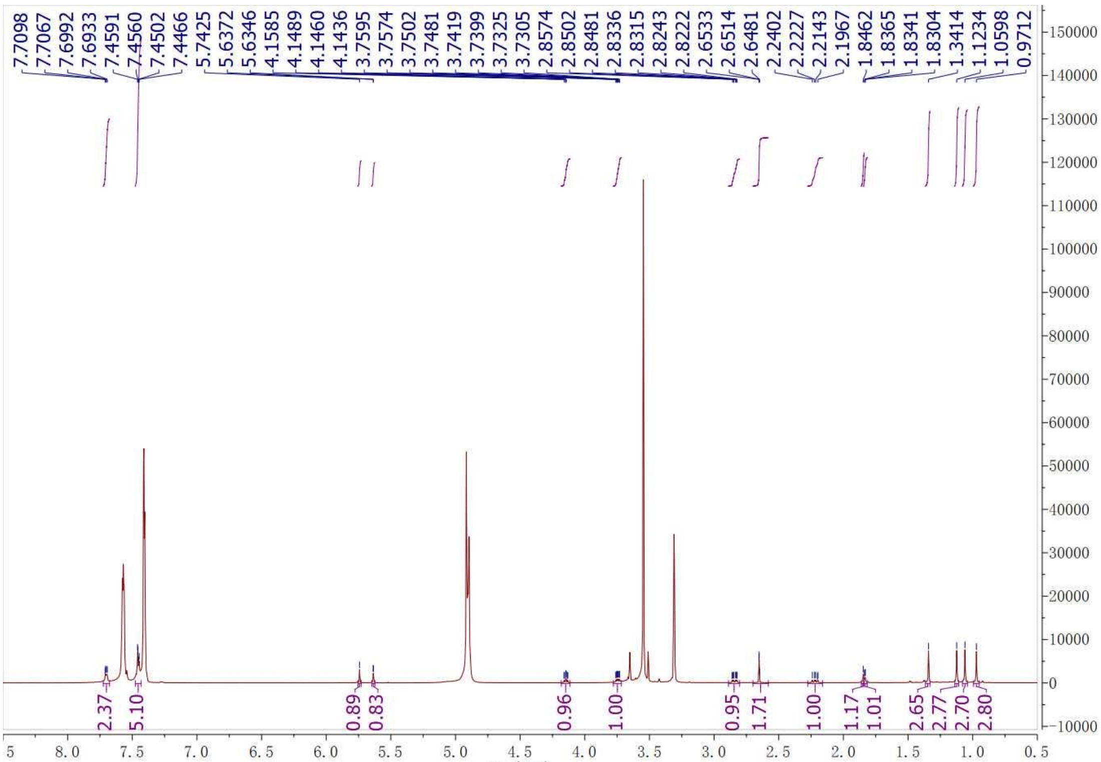


**Figure S151.** The $^{1}$H-NMR (600 MHz, $CD_3OD$) spectrum of **8a**.

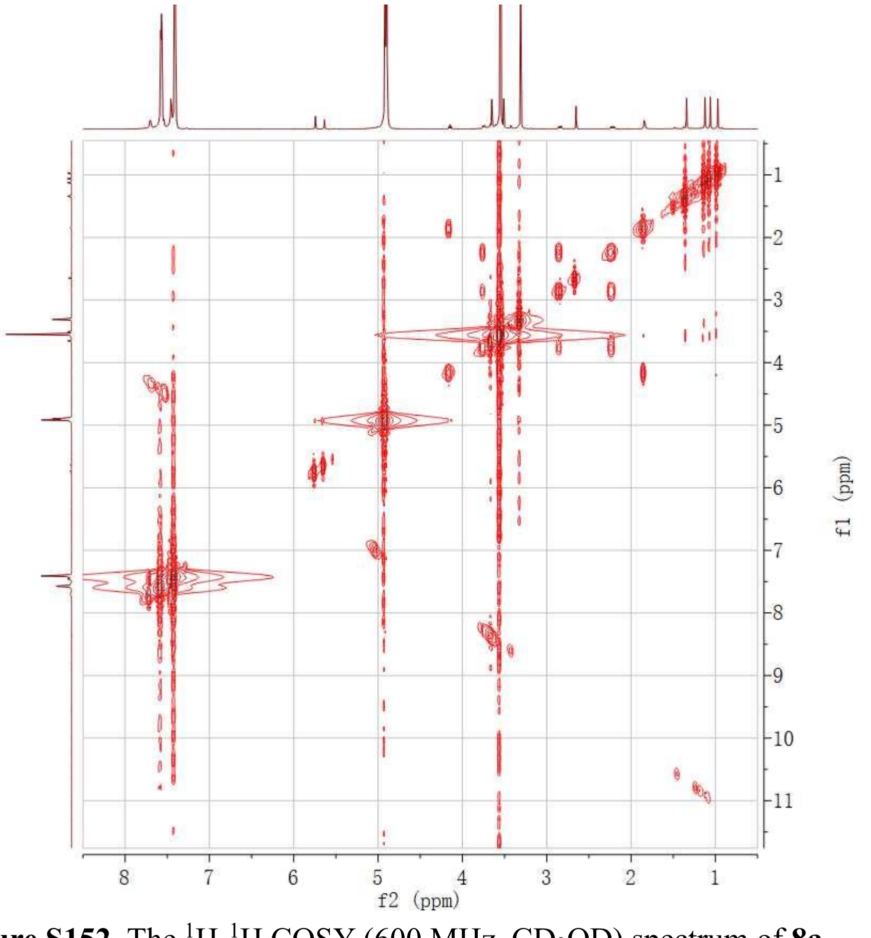


**Figure S152.** The $^{1}$H-$^{1}$H COSY (600 MHz, $CD_3OD$) spectrum of **8a**.

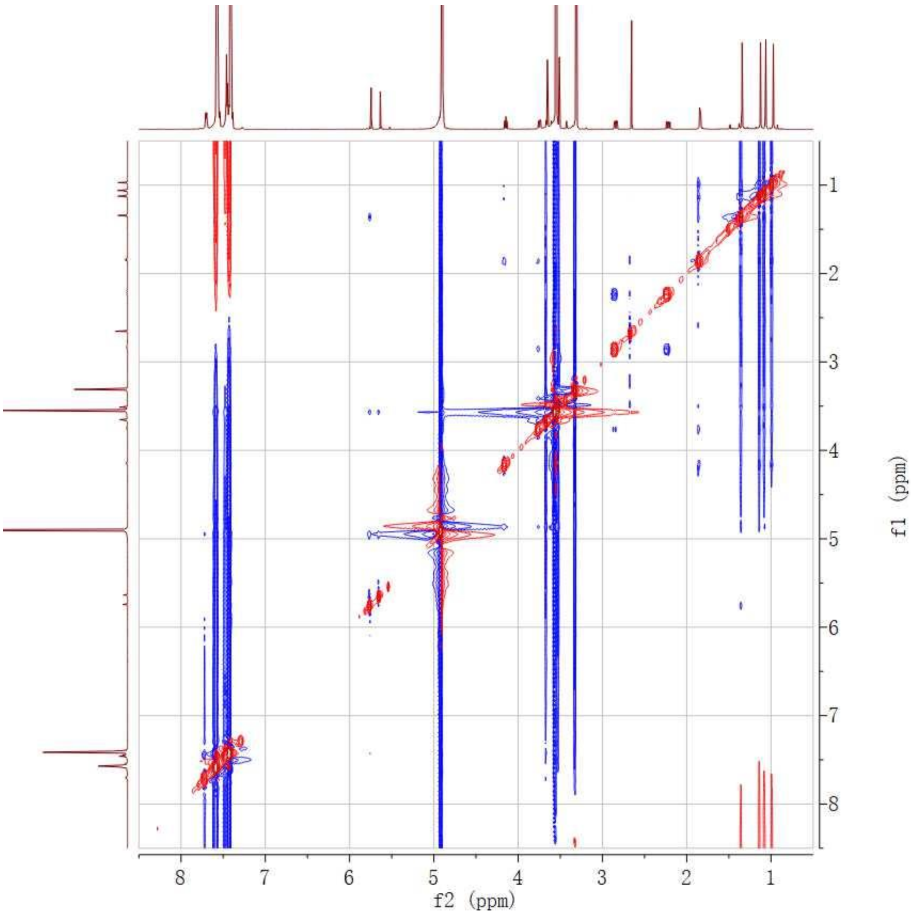


**Figure S153.** The ROESY (600 MHz, $CD_3OD$) spectrum of **8a.**

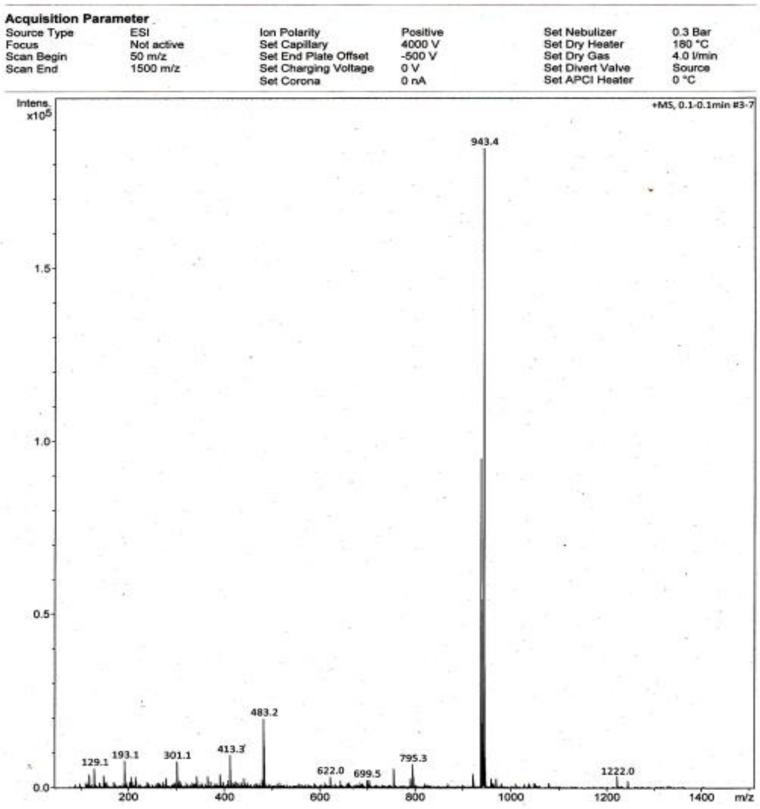


**Figure S154.** The ESIMS of **8a**.

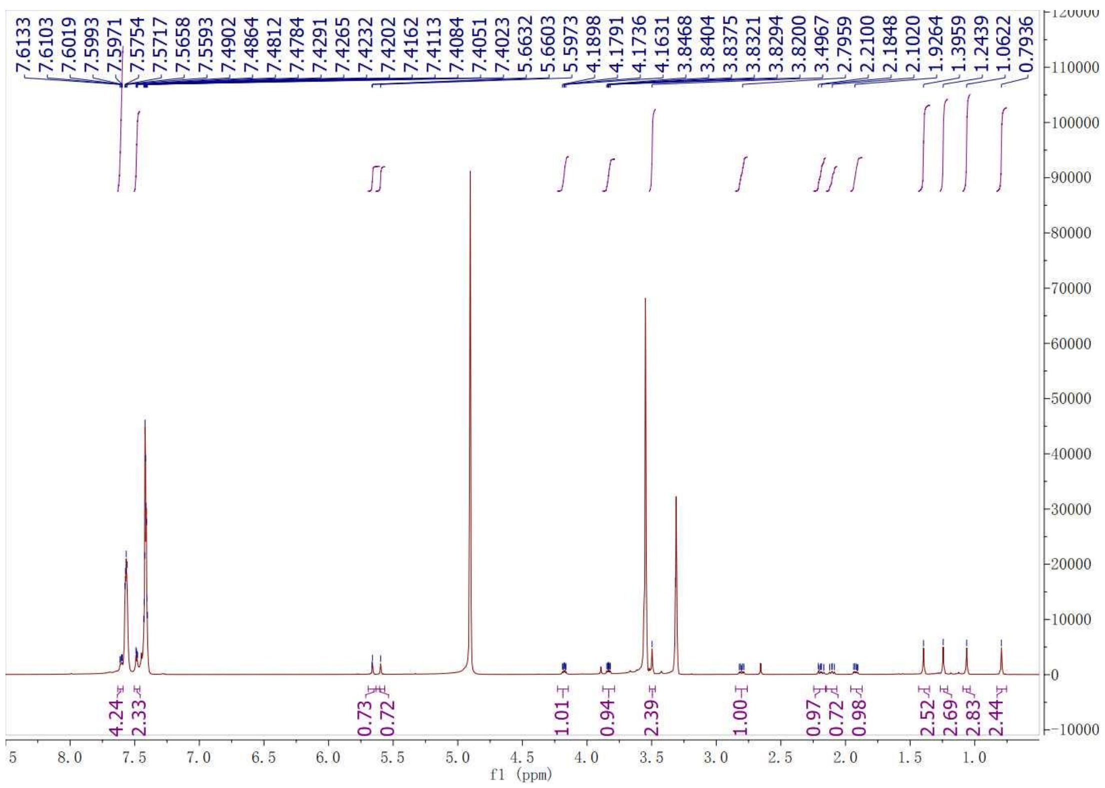

**Figure S155.** The $^1$H-NMR (600 MHz, $CD_3OD$) spectrum of **8b**.

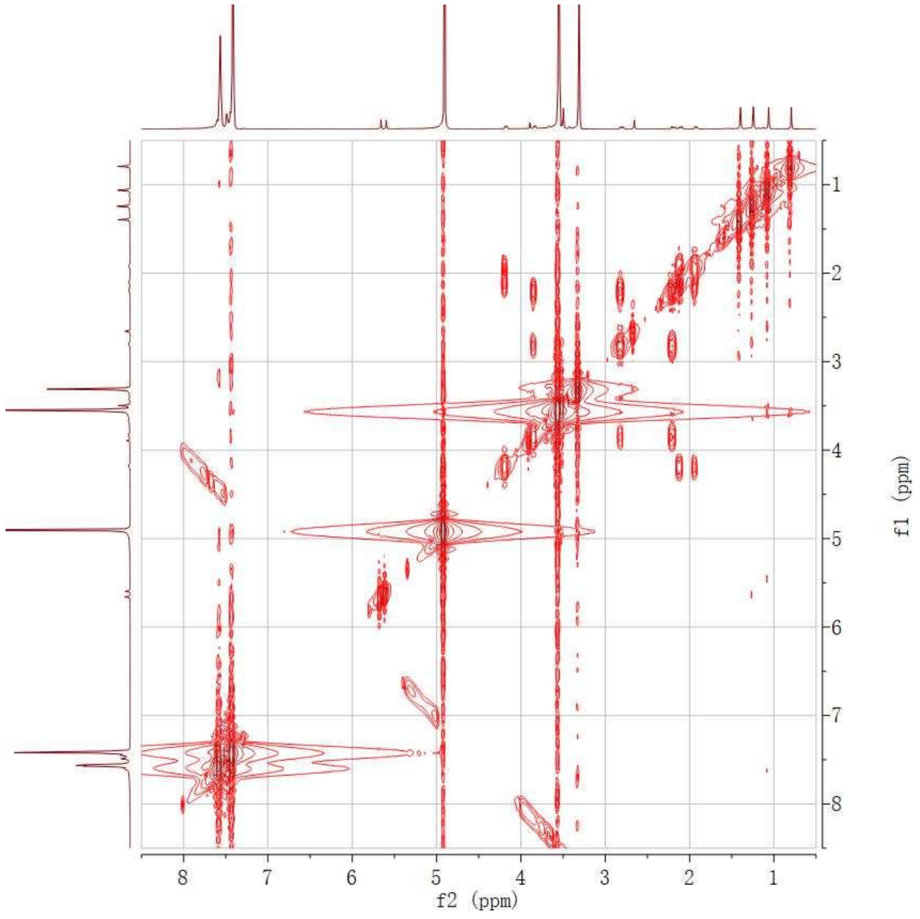

**Figure S156.** The $^1$H-$^1$H COSY (600 MHz, $CD_3OD$) spectrum of **8b**.

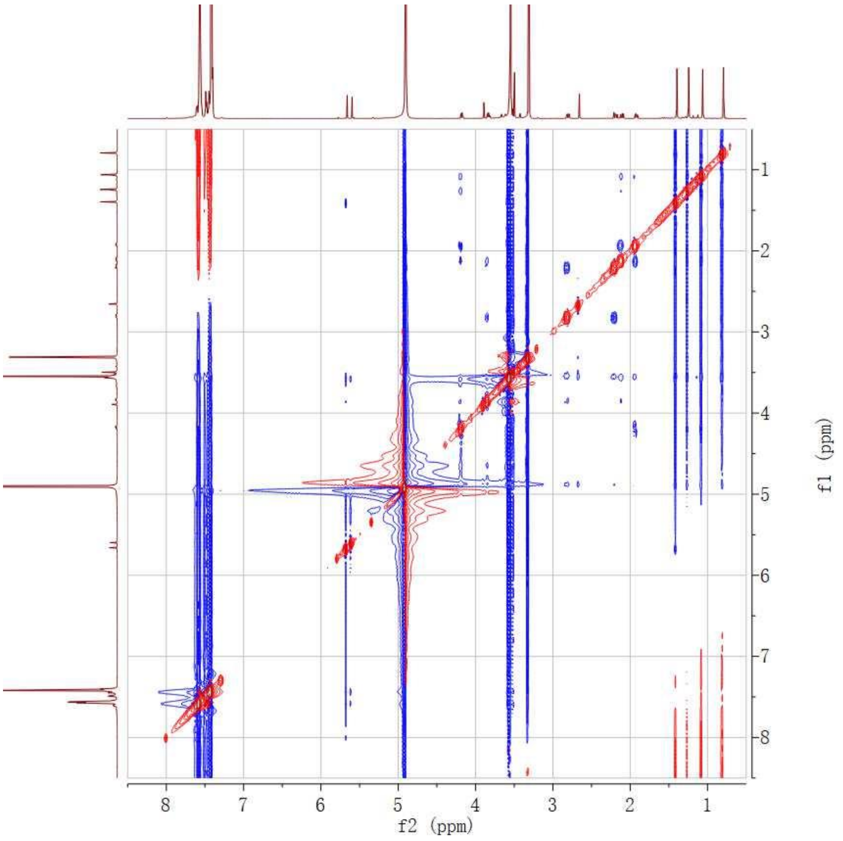


**Figure S157.** The ROESY (600 MHz, $CD_3OD$) spectrum of **8b.**

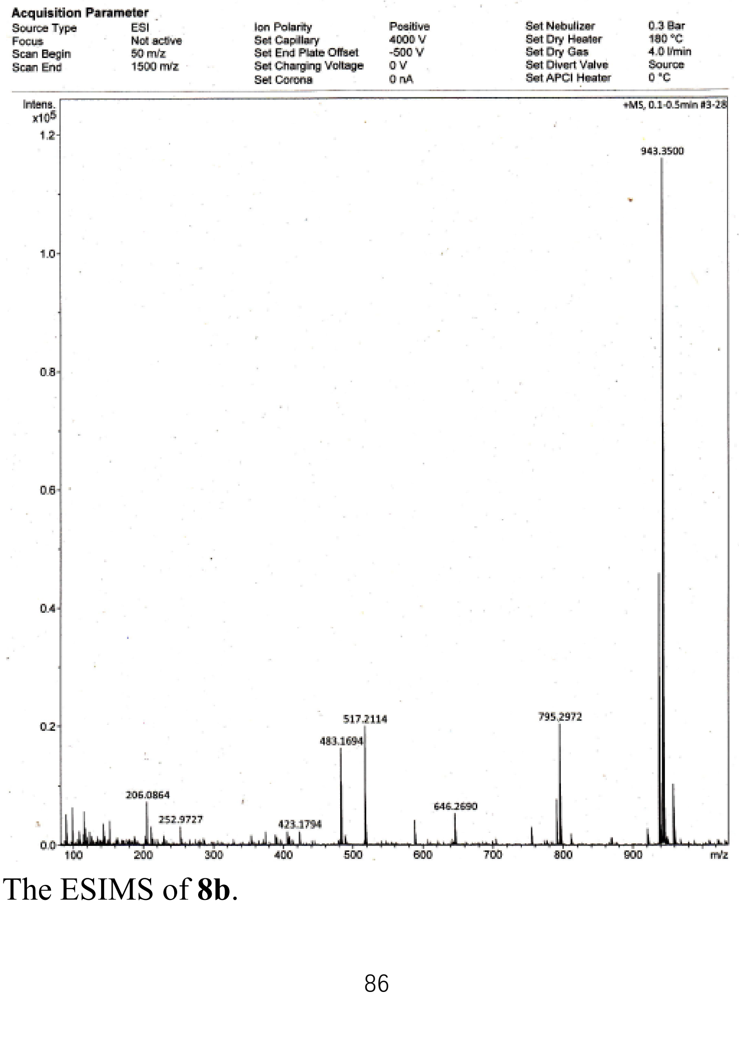


**Figure S158.** The ESIMS of **8b**.

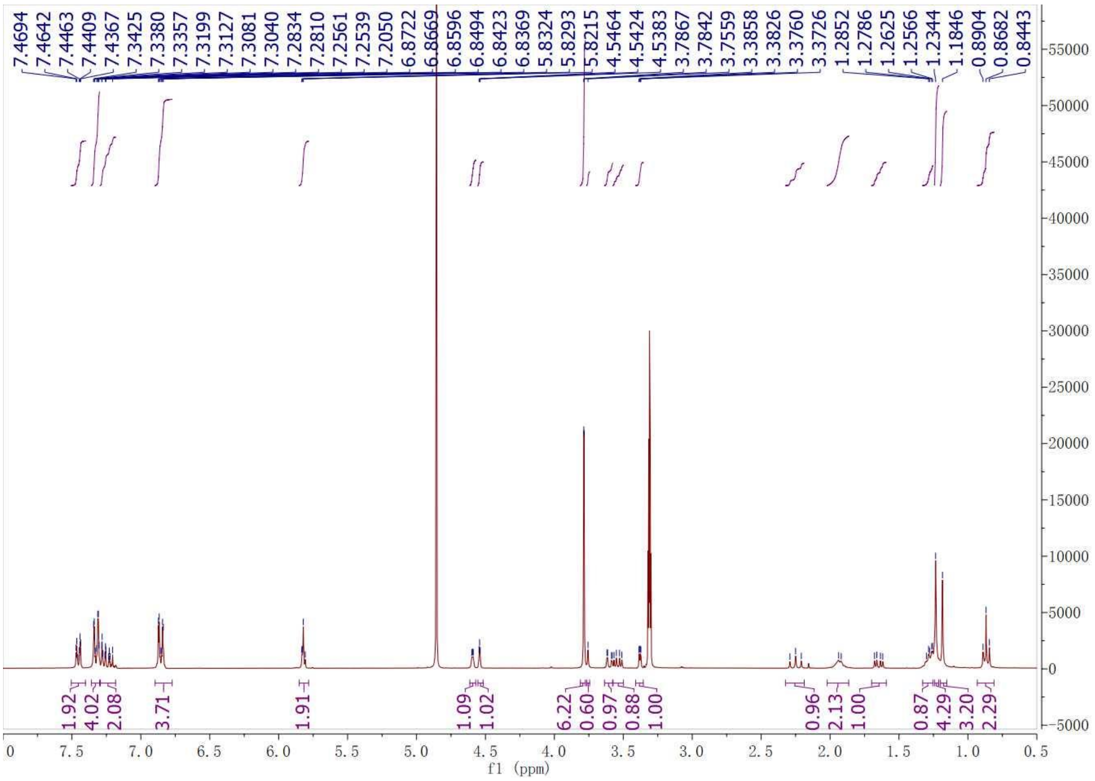


**Figure S159.** The $^{1}$H-NMR (600 MHz, $CD_3OD$) spectrum of 1**7a**.

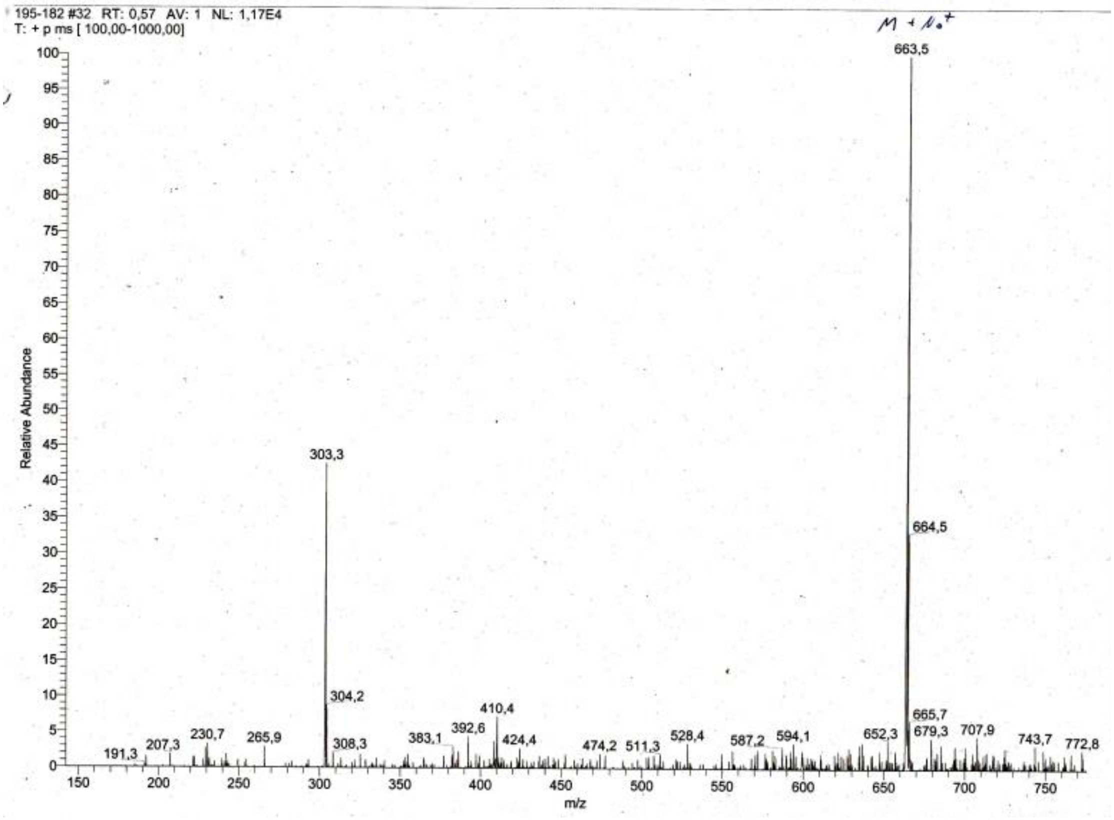


**Figure S160.** The ESIMS of 1**7a**.

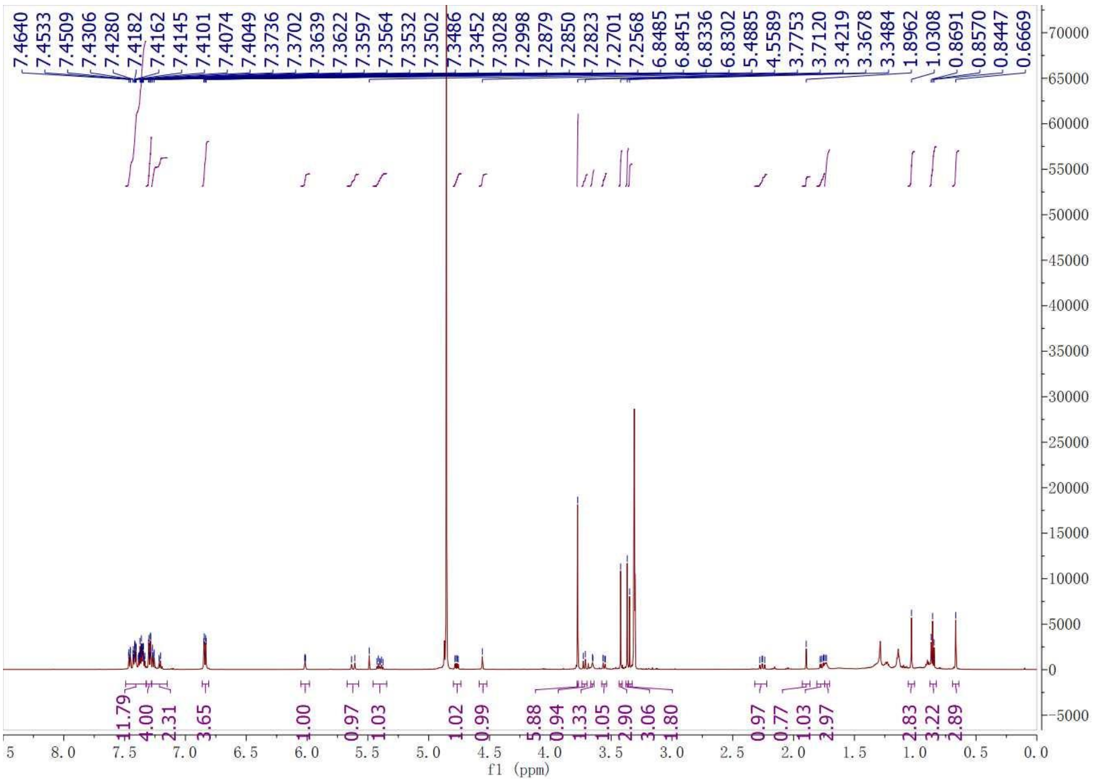

**Figure S161.** The $^{1}$H-NMR (600 MHz, $CD_3OD$) spectrum of 1**7b**.

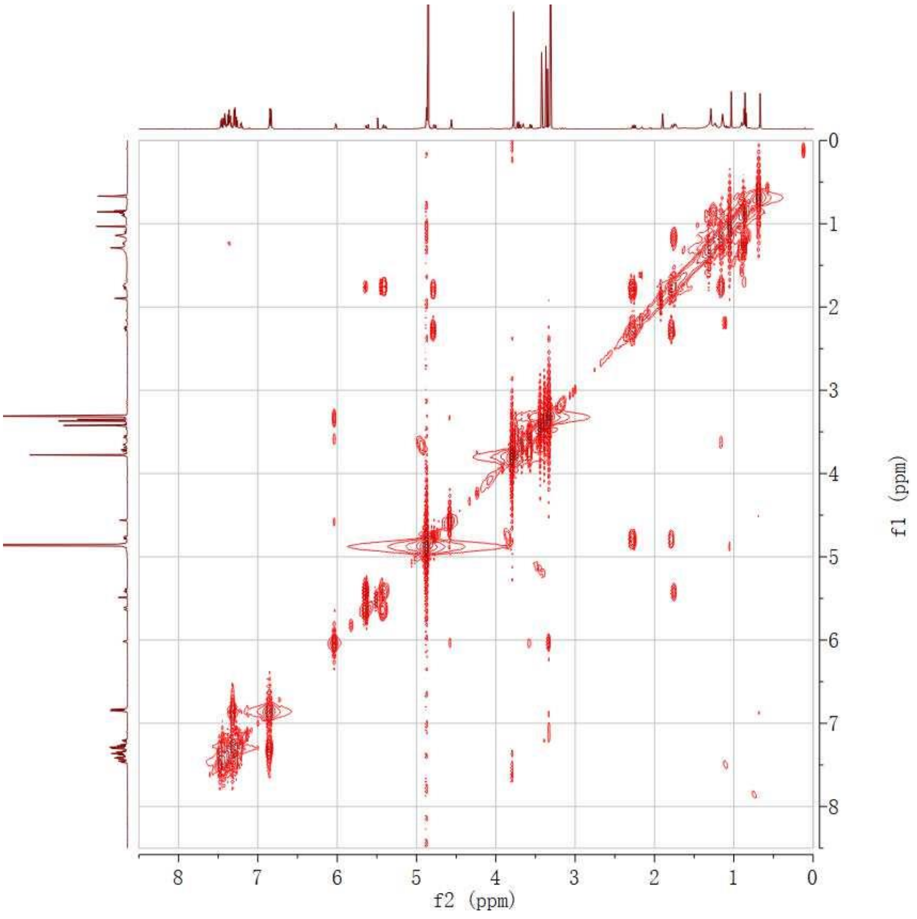

**Figure S162.** The $^{1}$H-$^{1}$H COSY (600 MHz, $CD_3OD$) spectrum of 1**7b**.

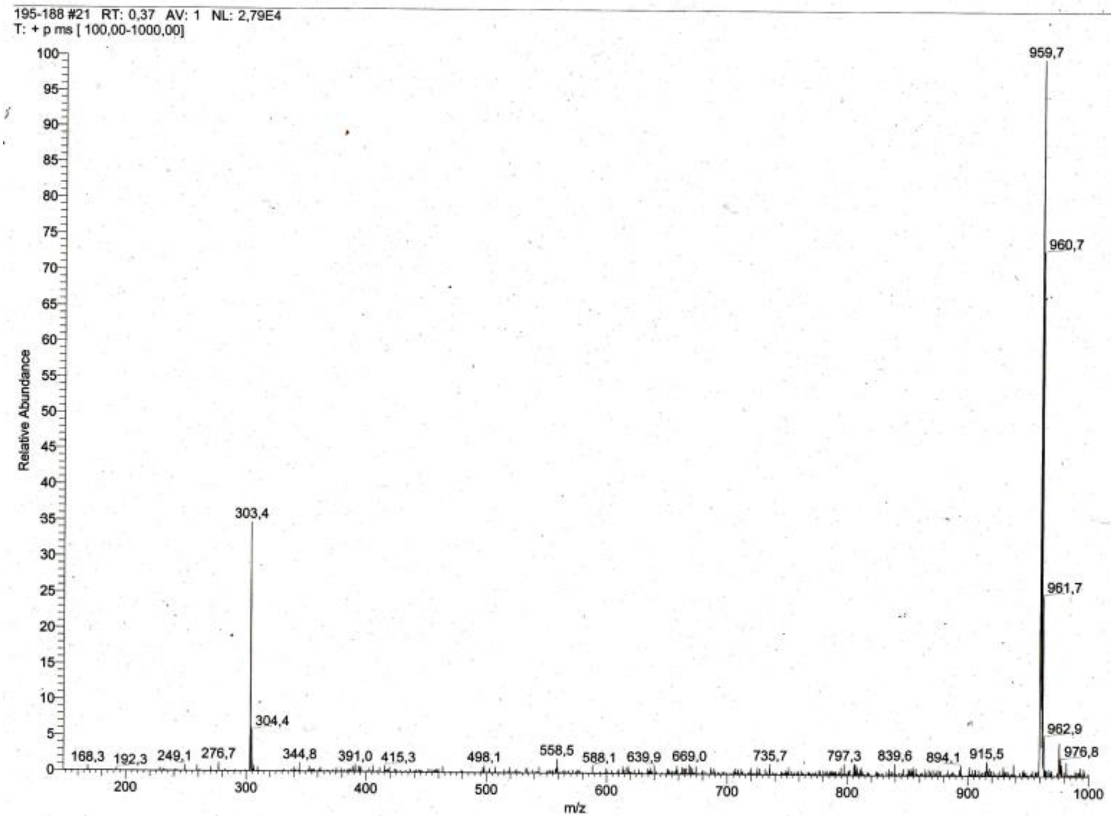


**Figure S163.** The ESIMS of 1**7b**.

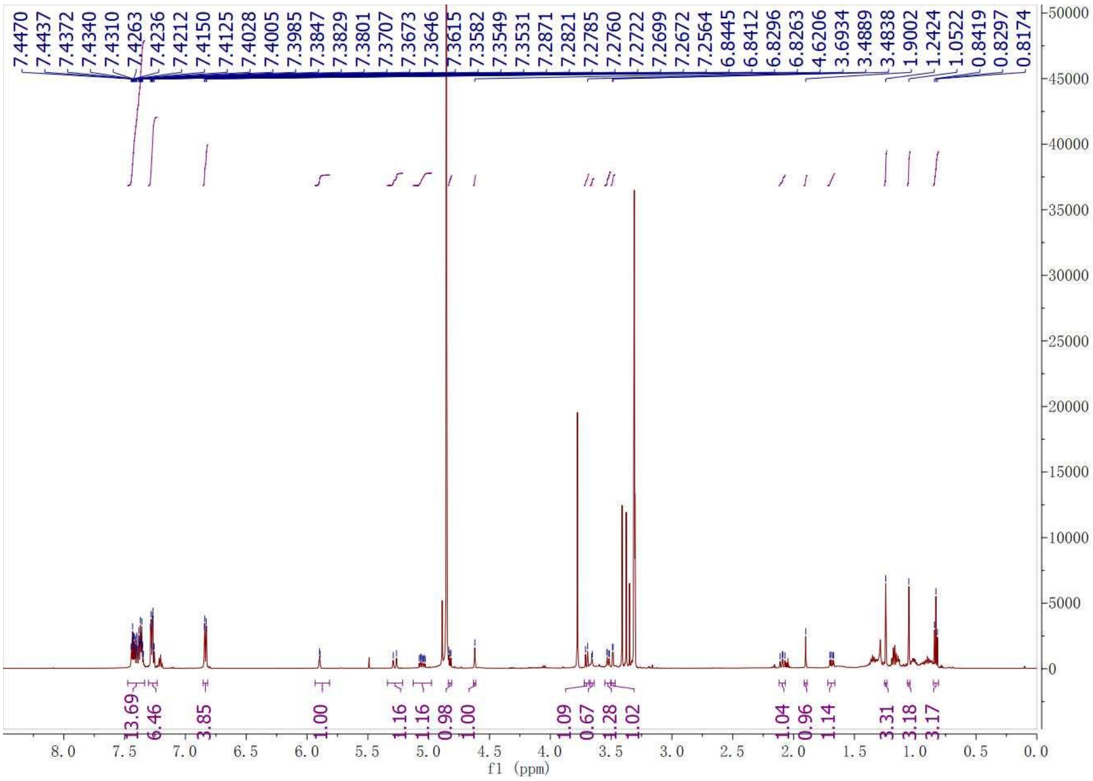


**Figure S164.** The $^{1}$H-NMR (600 MHz, $CD_3OD$) spectrum of **17c**.

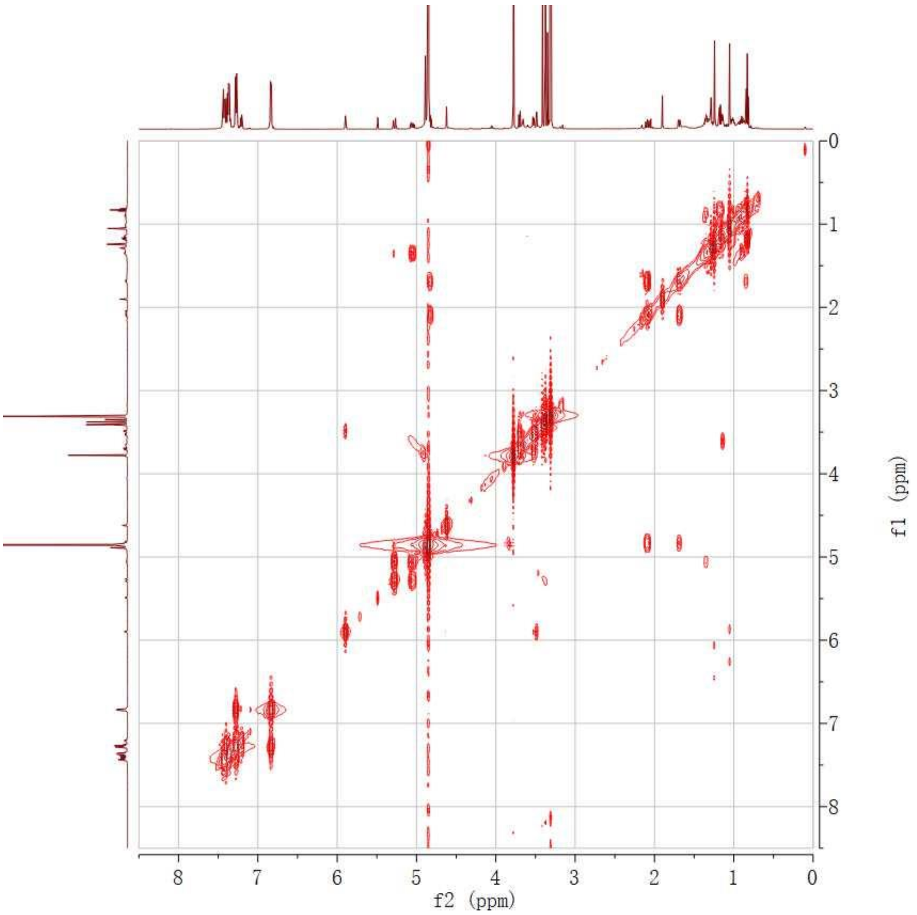


**Figure S165.** The $^{1}H$-$^{1}H$ COSY (600 MHz, $CD_3OD$) spectrum of 1**7c.**

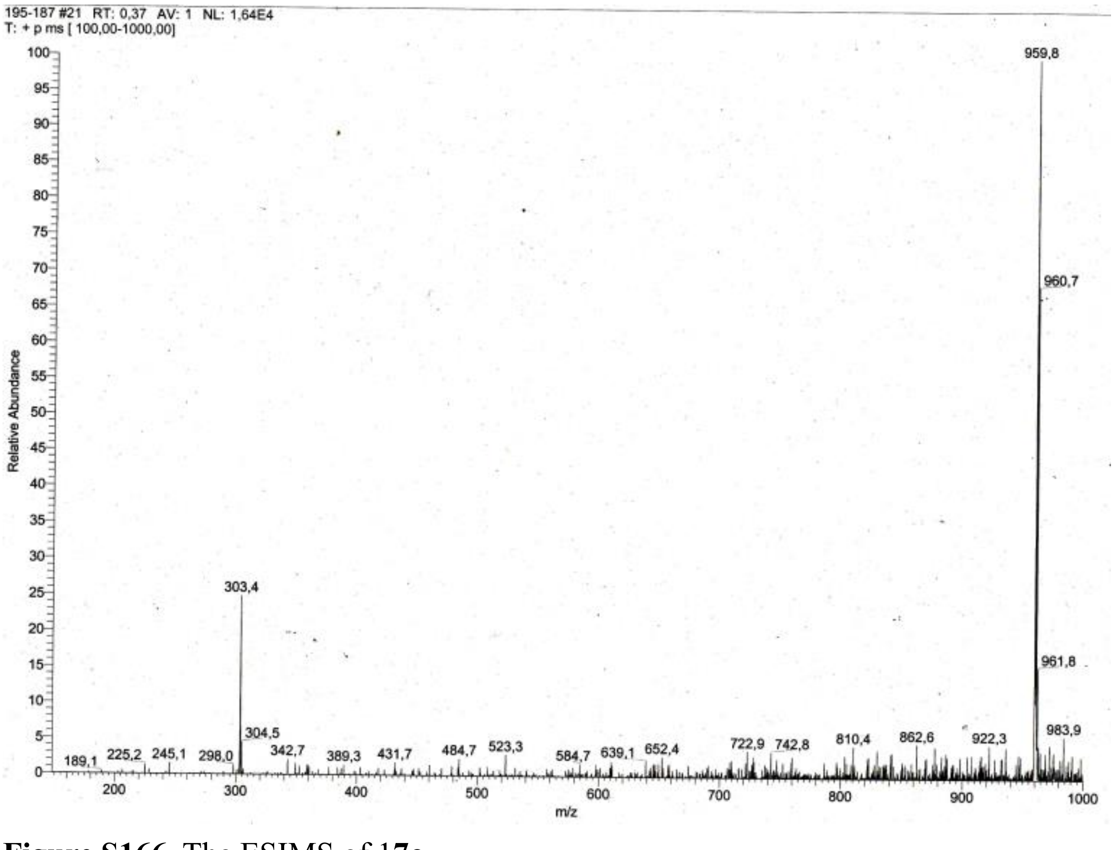


**Figure S166.** The ESIMS of 1**7c**.

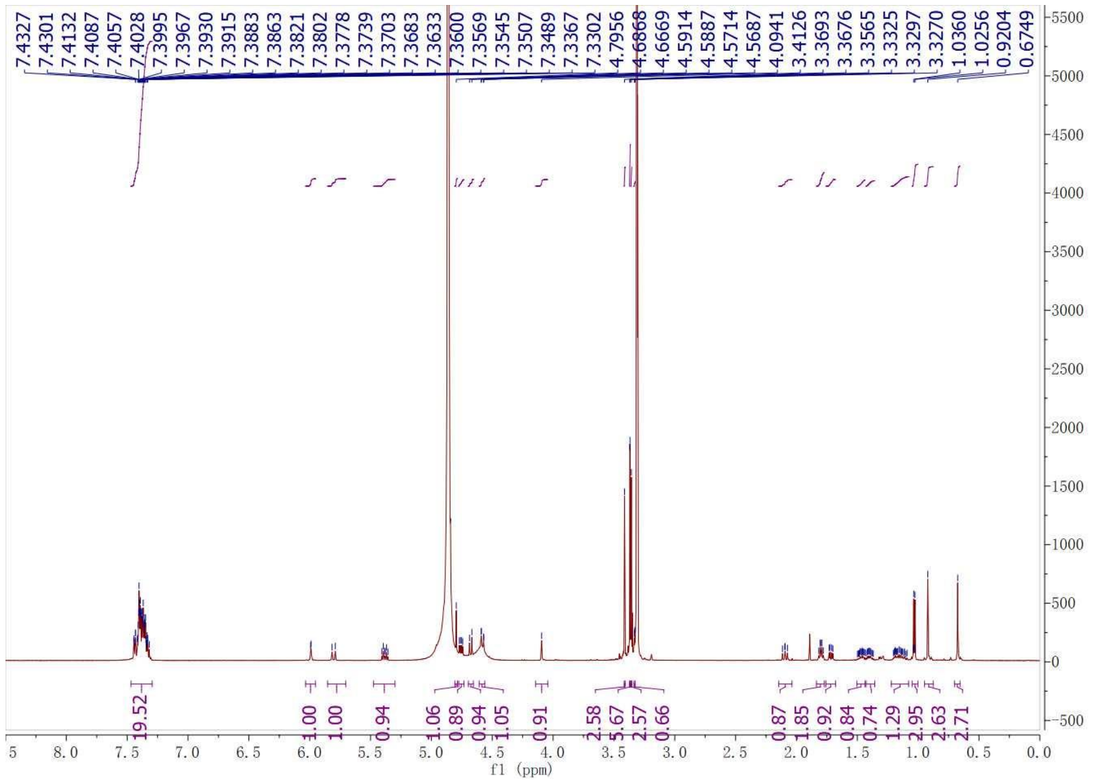

**Figure S167.** The $^{1}$H-NMR (600 MHz, $CD_3OD$) spectrum of **18a**.

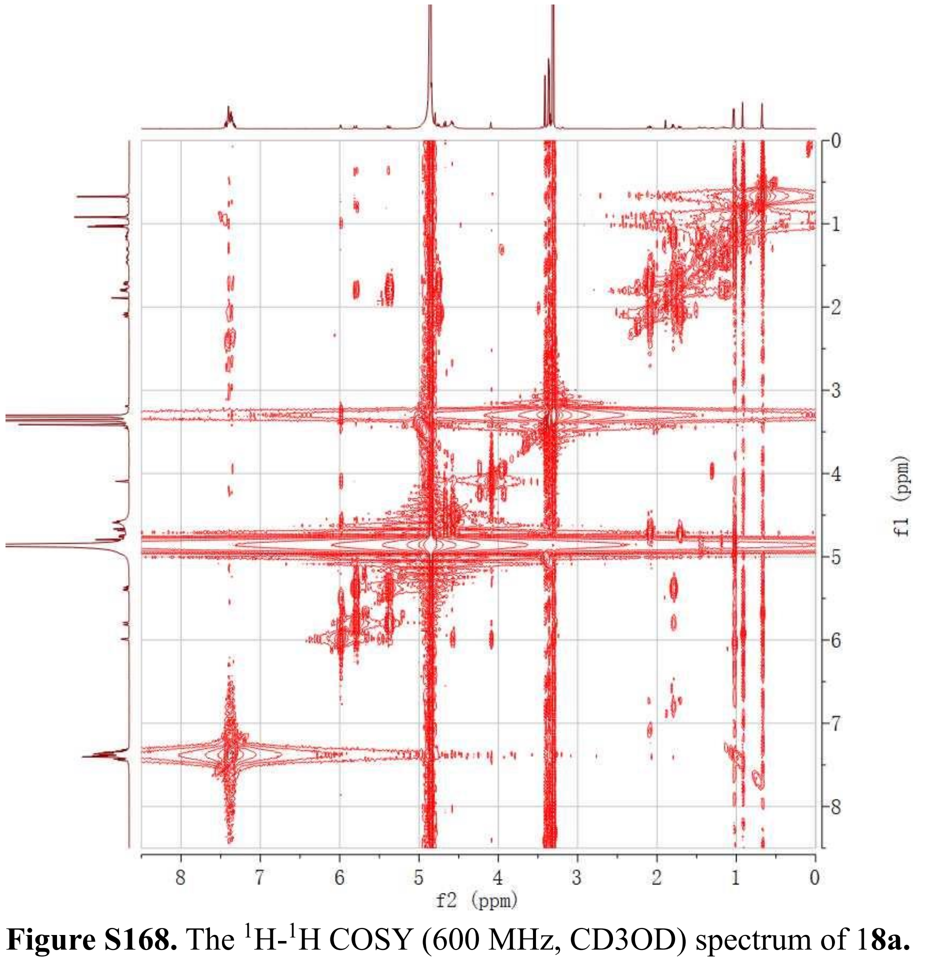

**Figure S168.** The $^{1}$H-$^{1}$H COSY (600 MHz, CD3OD) spectrum of 1**8a.**

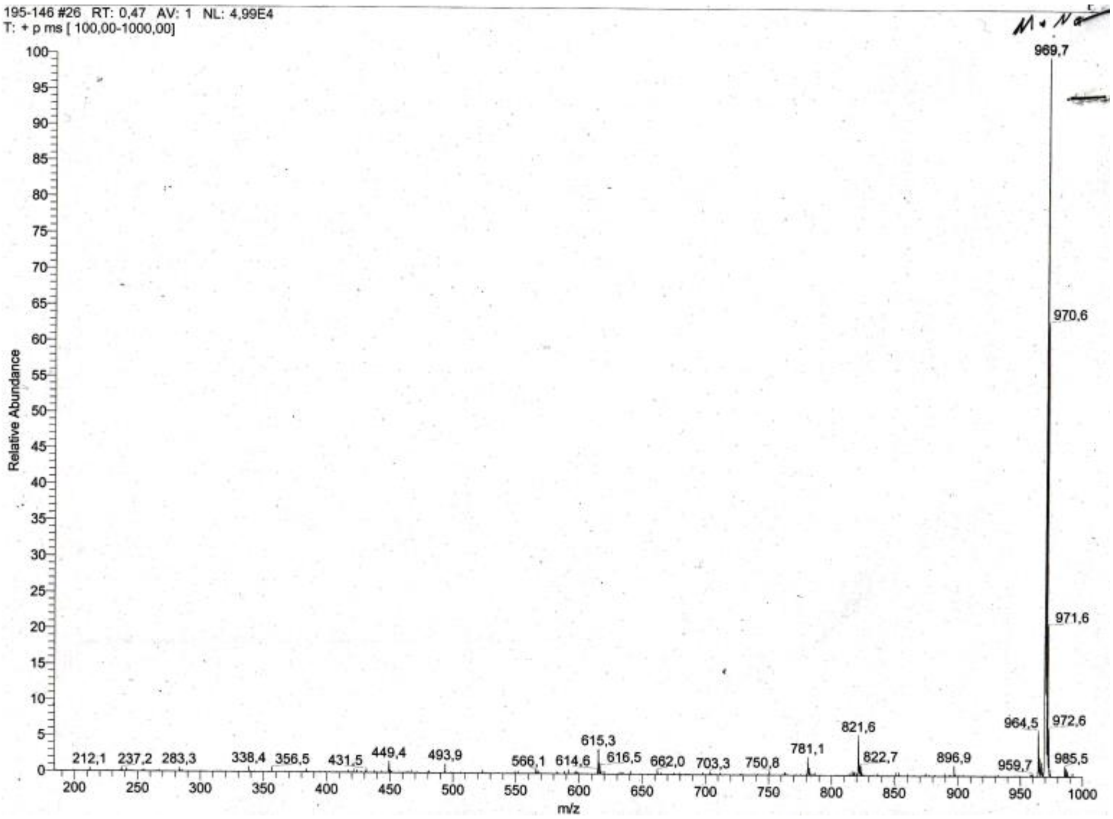


**Figure S169.** The ESIMS of 1**8a**.

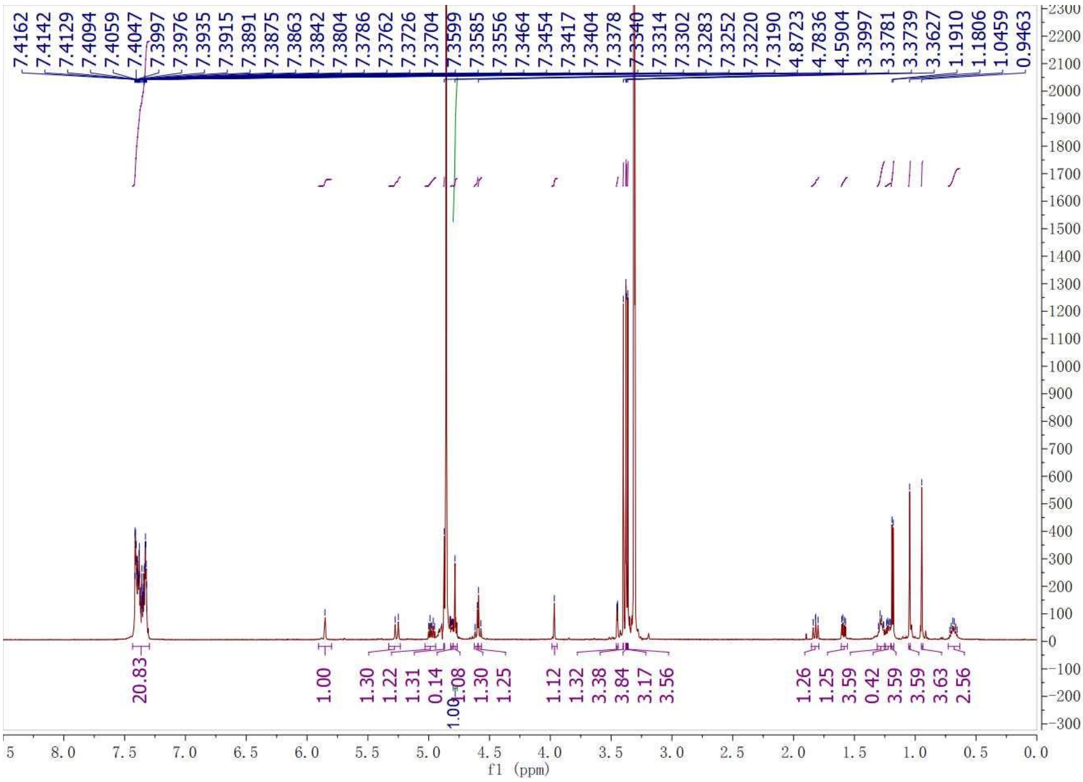


**Figure S170.** The $^{1}$H-NMR (600 MHz, $CD_3OD$) spectrum of **18b**.

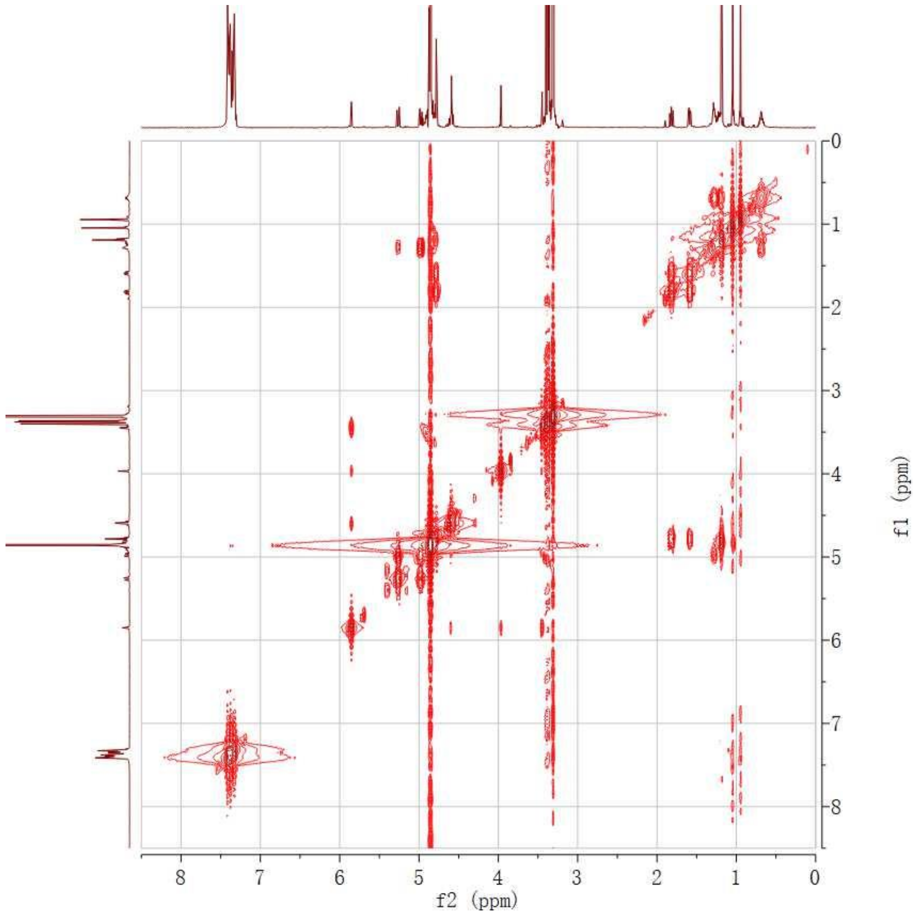


**Figure S171.** The $^{1}H$-$^{1}H$ COSY (600 MHz, $CD_3OD$) spectrum of 1**8b**.

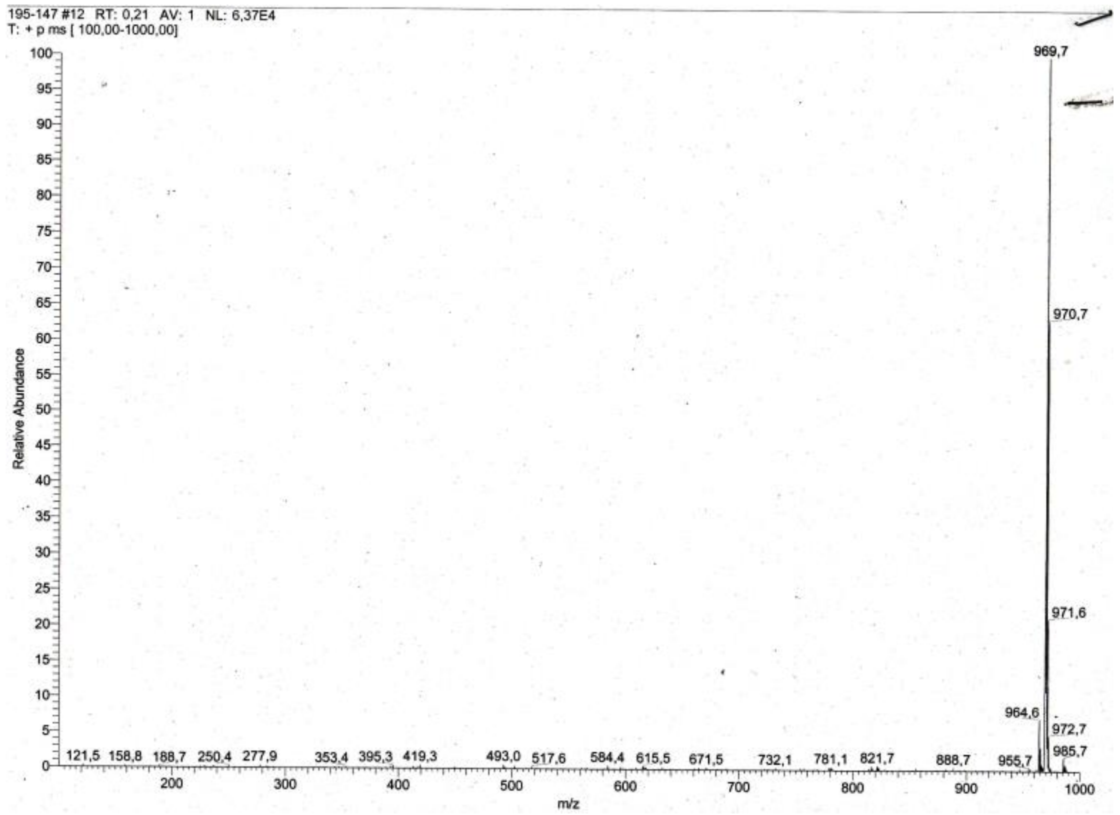


**Figure S172.** The ESIMS of 1**8b**.

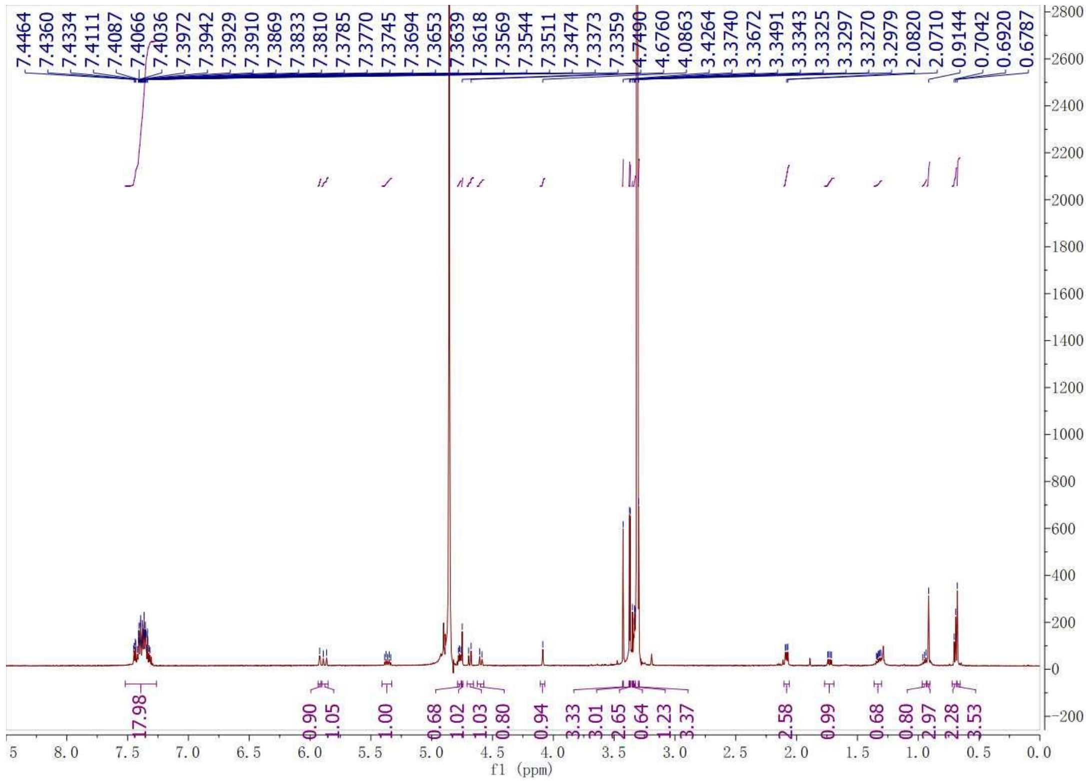

**Figure S173.** The $^1$H-NMR (600 MHz, $CD_3OD$) spectrum of **25a**.

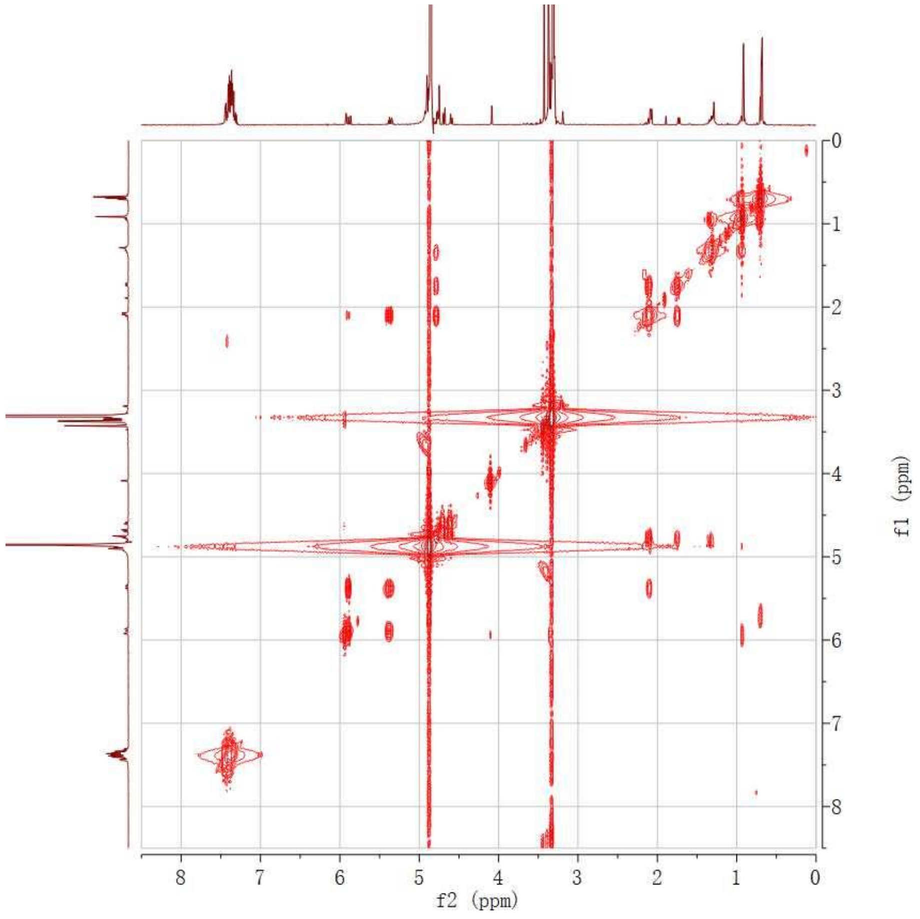

**Figure S174.** The $^1$H-$^1$H COSY (600 MHz, $CD_3OD$) spectrum of **25a**.

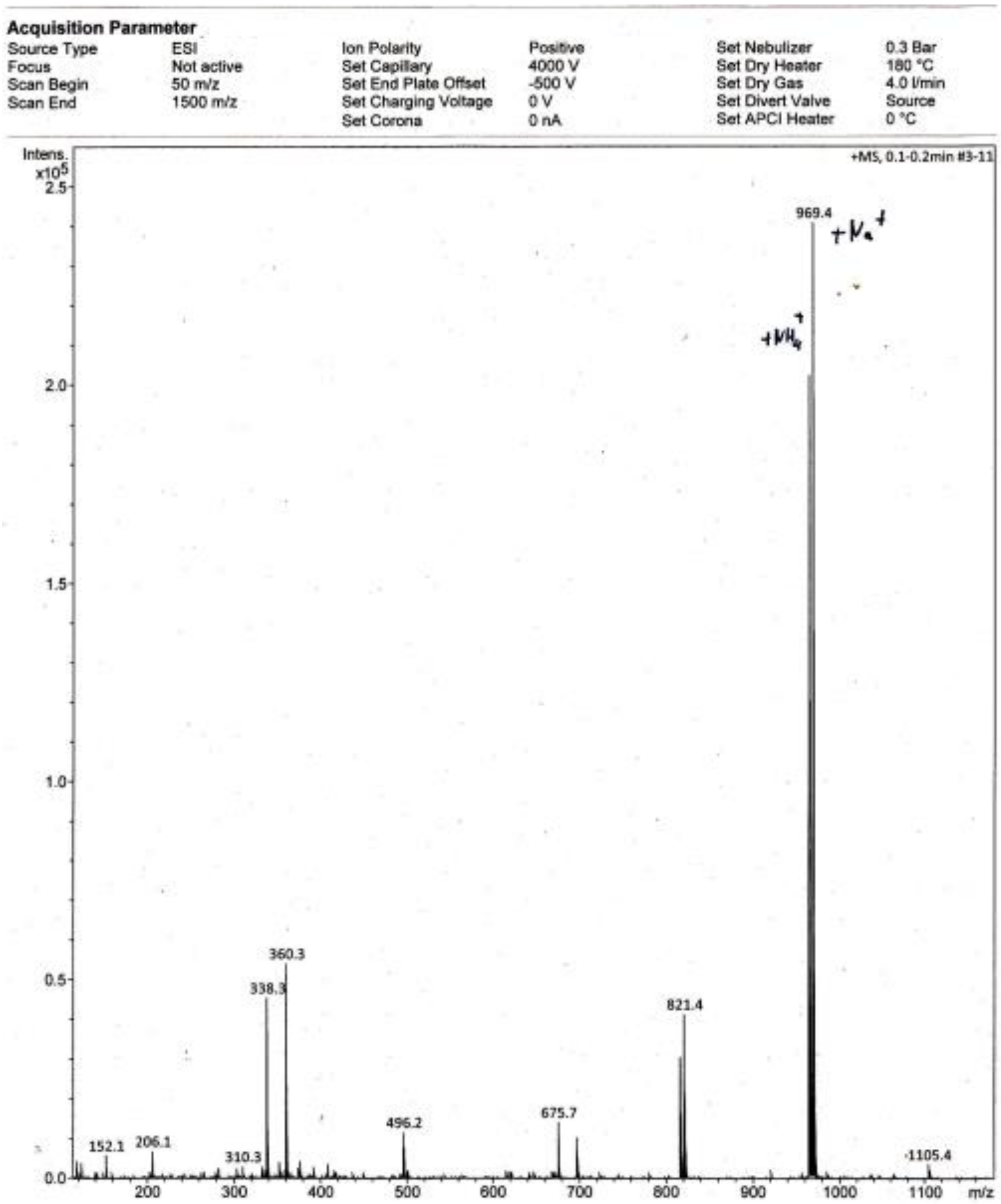


**Figure S175.** The ESIMS of **25a**.

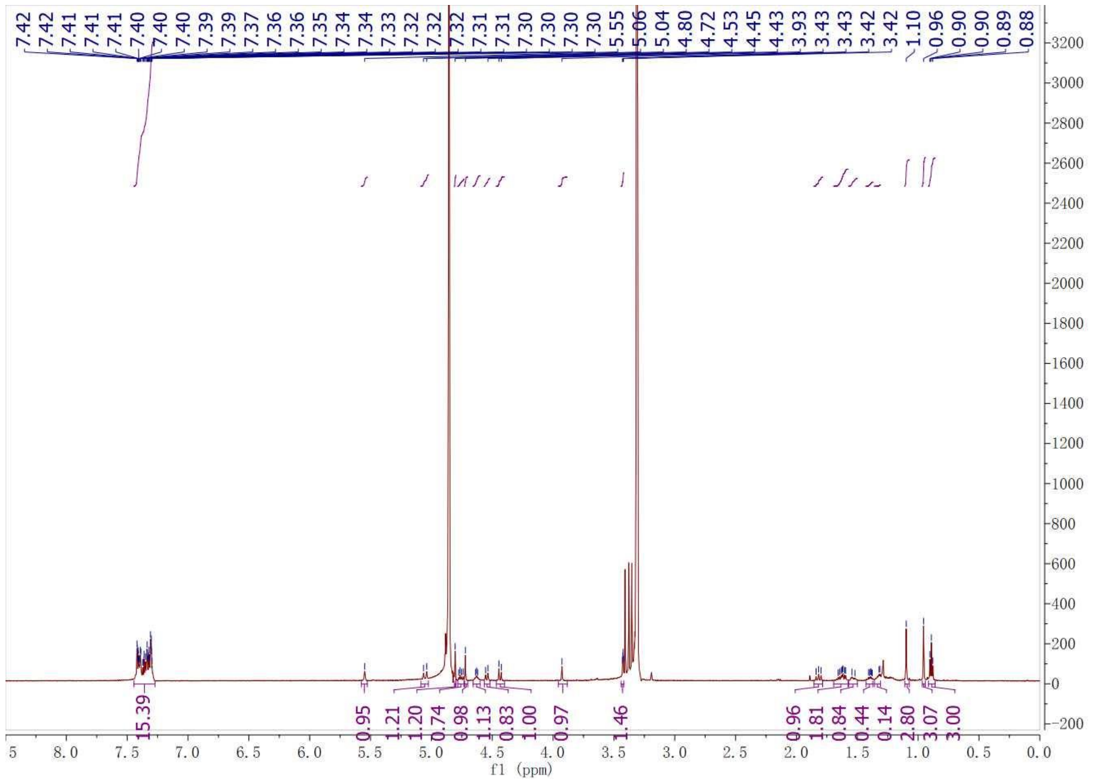

**Figure S176.** The $^{1}$H-NMR (600 MHz, $CD_3OD$) spectrum of **25b**.

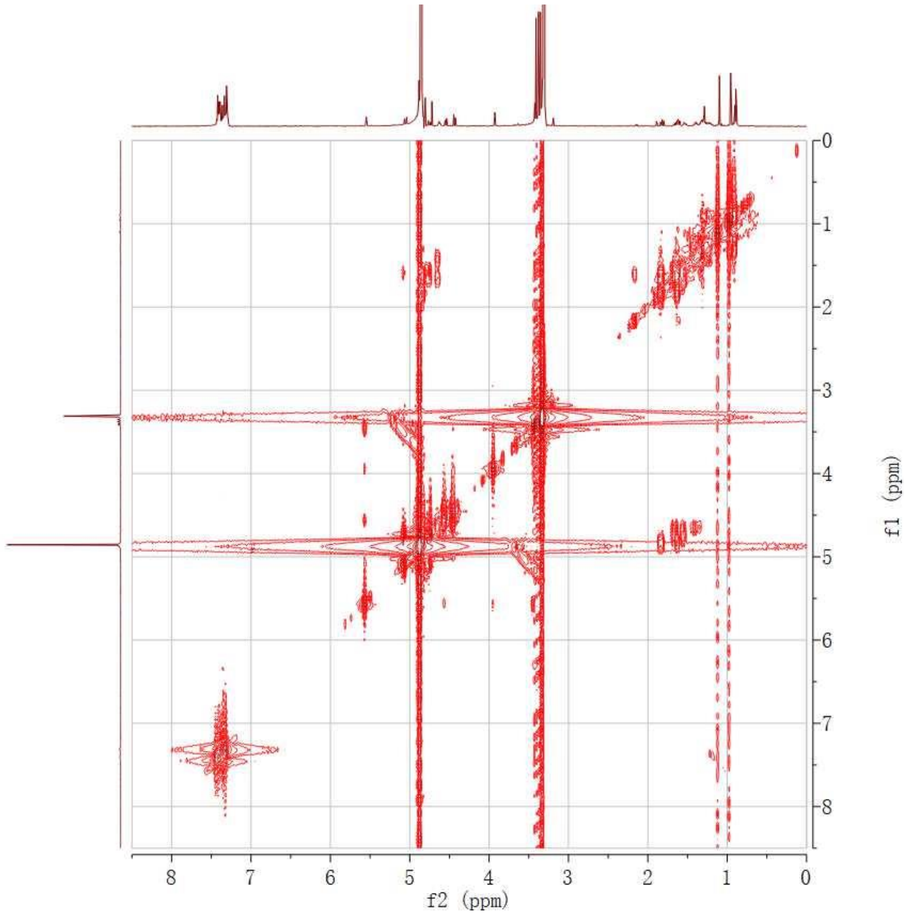

**Figure S177.** The $^{1}$H-$^{1}$H COSY (600 MHz, $CD_3OD$) spectrum of **25b**.

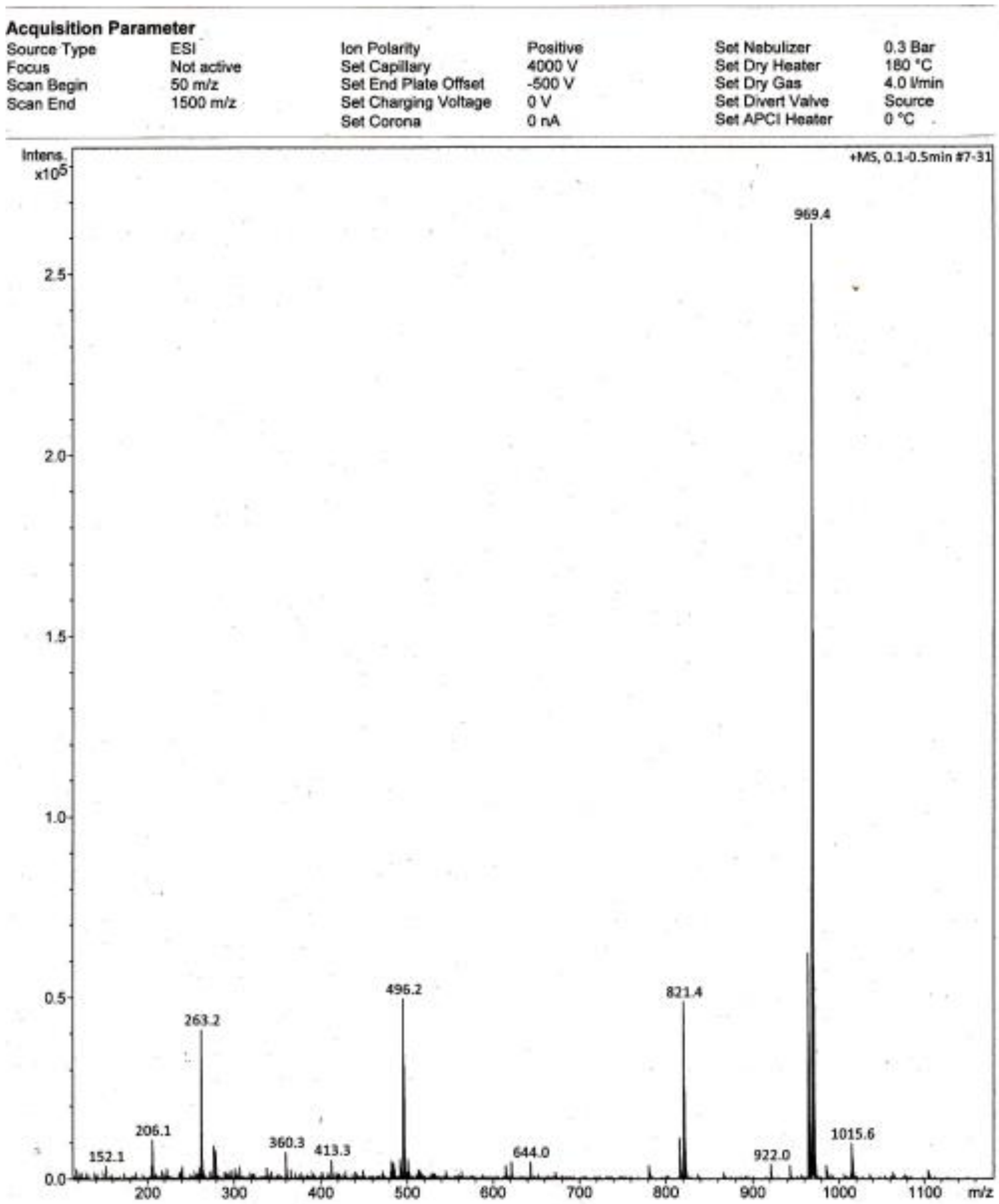


**Figure S178.** The ESIMS of **25b**.

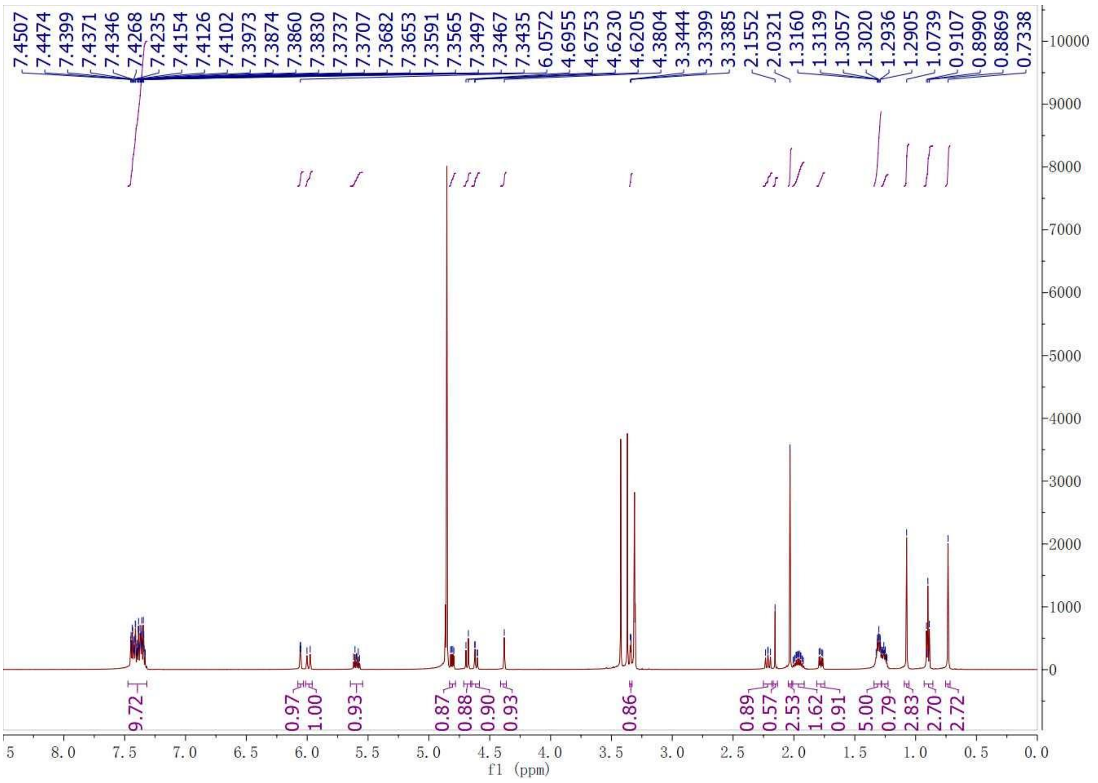

**Figure S179.** The $^{1}$H-NMR (600 MHz, $CD_3OD$) spectrum of **26a**.

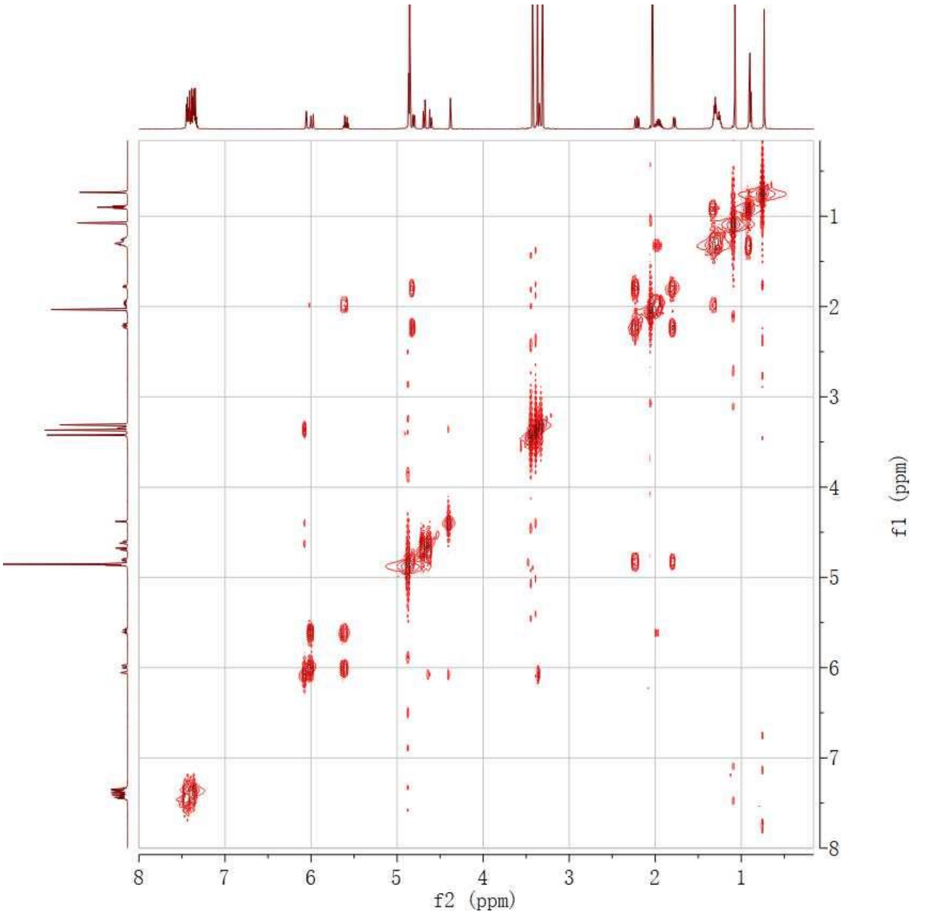

**Figure S180.** The $^{1}$H-$^{1}$H COSY (600 MHz, $CD_3OD$) spectrum of **26a**.

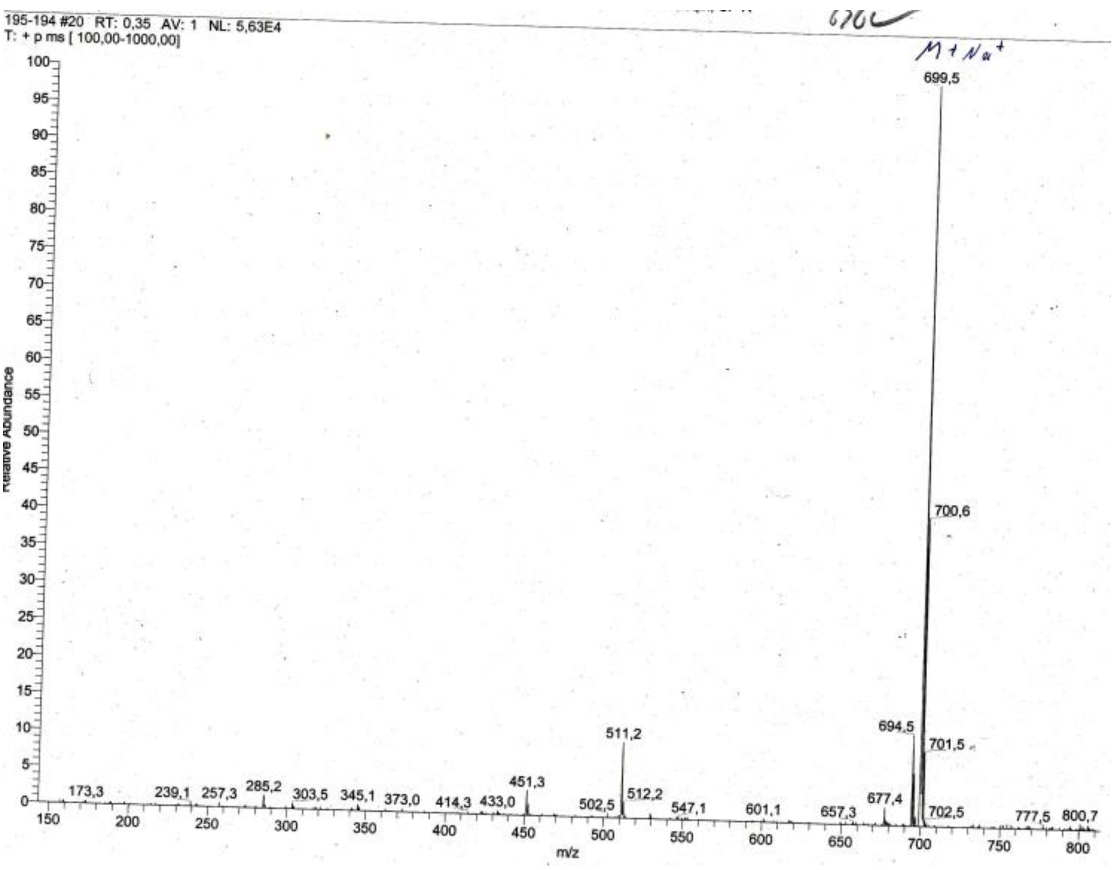

**Figure S181.** The ESIMS of **26a**.

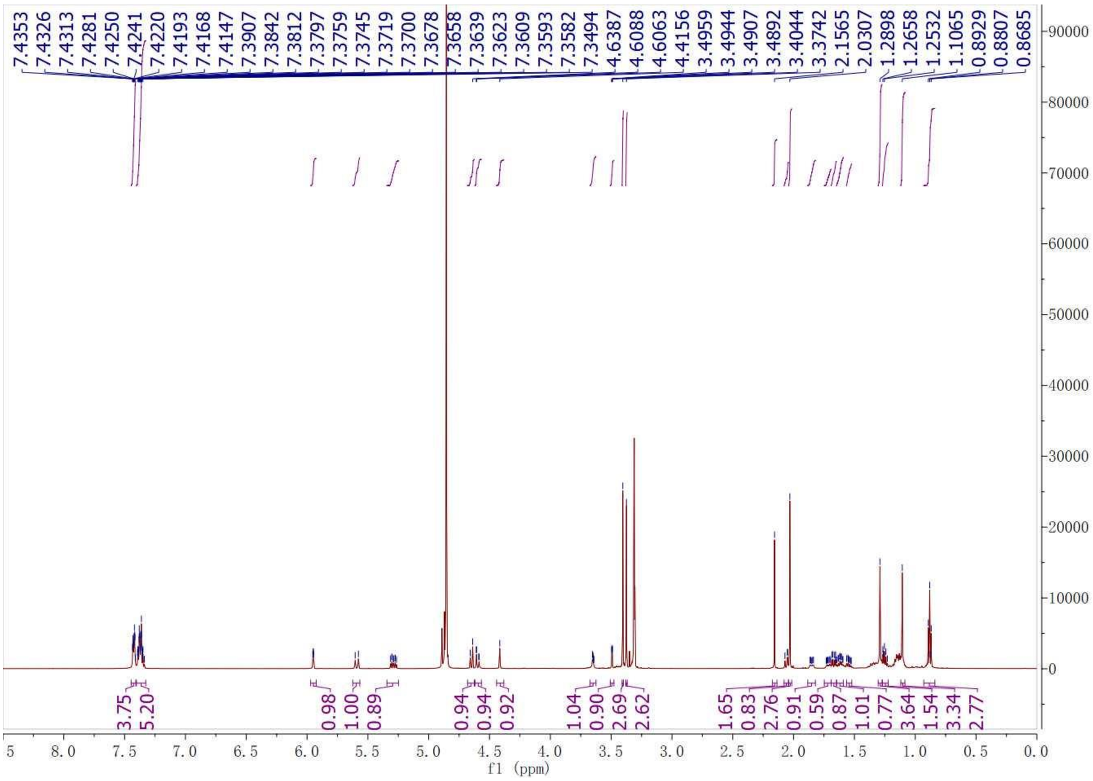

**Figure S182.** The $^{1}$H-NMR (600 MHz, $CD_3OD$) spectrum of **26b**.

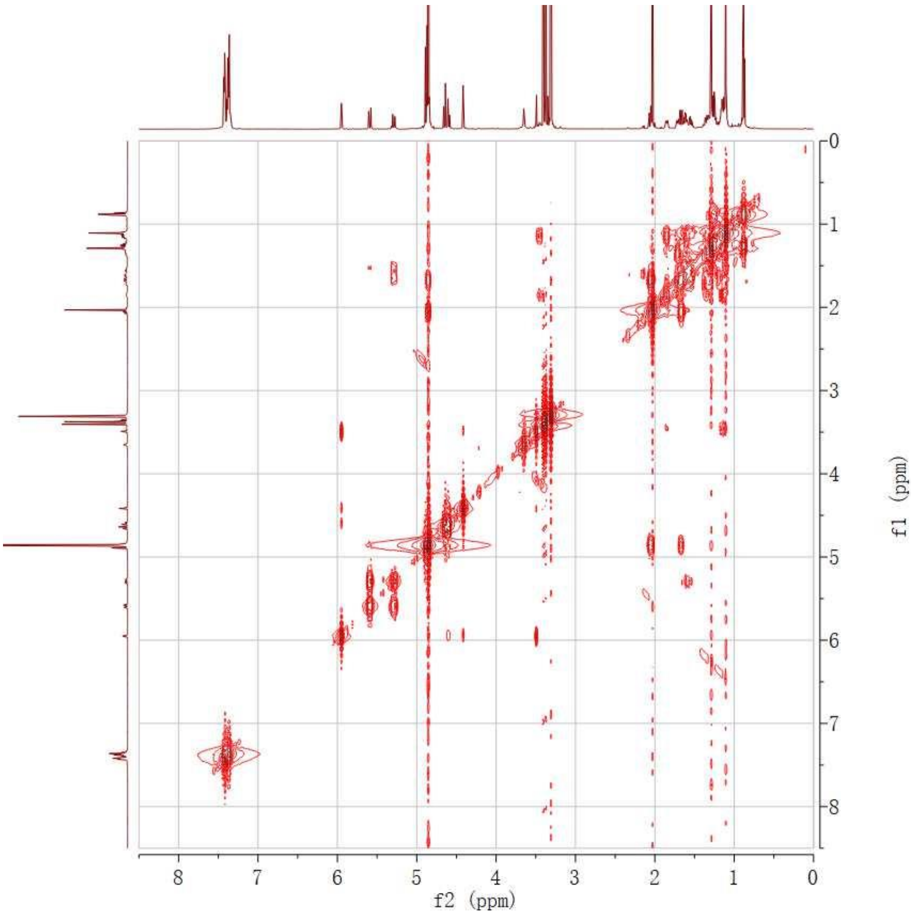


**Figure S183.** The $^{1}H$-$^{1}H$ COSY (600 MHz, $CD_3OD$) spectrum of **26b**.

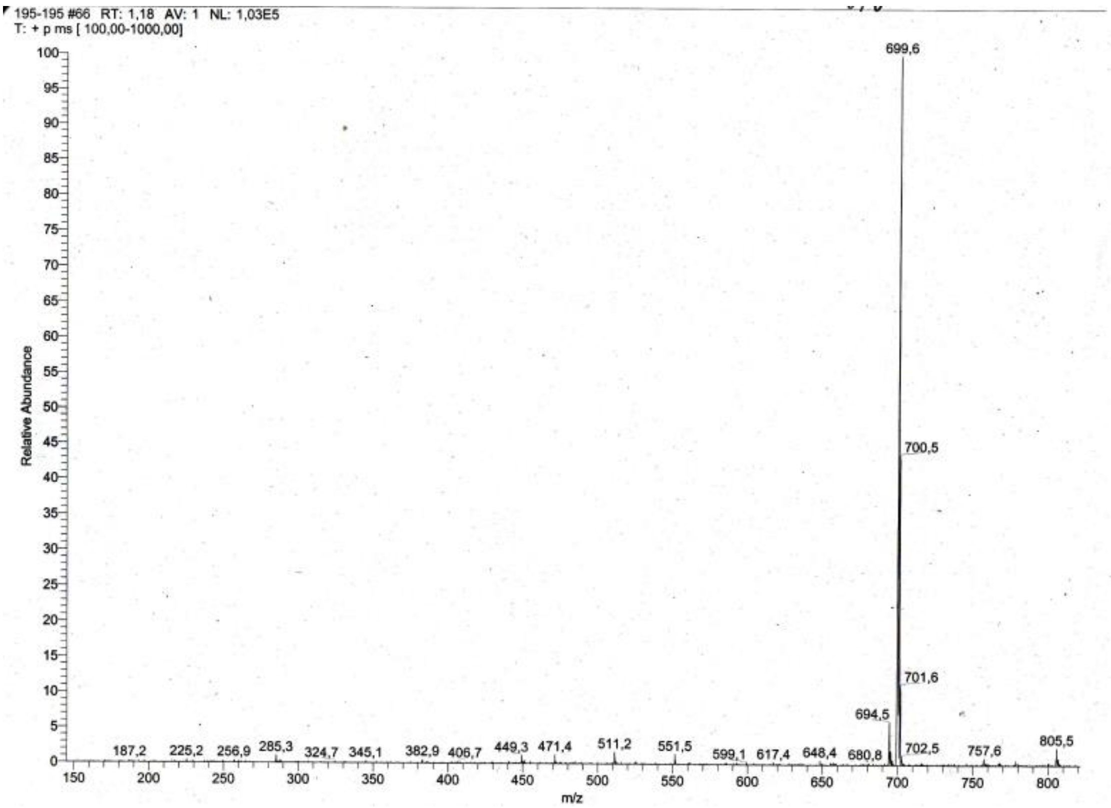


**Figure S184.** The ESIMS of **26b**.

**Table S1.** SMILES table of compounds **1**−**26**.

| No. | **SMILES** |
|---|---|
| **1** | CC(C)(O)[C@H](O1)C[C@]2([R1])[C@]1([H])CC(C(/C=C(C)/C)=O)=C[C@@H]2[R2] |
| **2** | CC(C)(O)[C@H](O1)C[C@@]2([R1])[C@]1([H])CC(C(/C=C(C)/C)=O)=C[C@H]2[R2] |
| **3** | CC(C)(O)[C@H](O1)C[C@]23[C@]1([H])C/C([C@@H](O)[C@H]2O3)=C/C=C(CO)/C |
| **4** | CC(C)(O)[C@H](O1)C[C@@]2(O)[C@]1([H])C=C(C#CC(C)=C)[C@@H](O)[C@H]2O |
| **5** | CC(C)(O)C(O1)C[C@]2(O)[C@]1([H])C=C(C#CC(C)=C)[C@@H](O)[C@@H]2O |
| **6** | CC(C)(O)[C@H](O1)C[C@]2(O)[C@]1([H])CC([C@@H](O)[C@@H]2O)=[C@]=CC(C)=C |
| **7** | CC(C)(O)[C@H](O1)C[C@]2(O)[C@]1([H])CC([C@@H](O)[C@@H]2OC(C)=O)=[C@]=CC(C)=C |
| **8** | CC(C)(O)[C@H](O1)C[C@]2(O)[C@]1([H])CC(C([C@H](O)C(C)(C)O3)=O)=C3[C@@H]2O |
| **9** | CC(C)(O)[C@H](O1)C[C@]2(O)[C@]1([H])CC(C(C([H])C(C)(C)O3)=O)=C3[C@@H]2OC(C)=O |
| **10** | C=C(C)C#C[C@H]1C[C@@](OC(C)(C)[C@@H](O)C2)([H])[C@]2(O3)[C@H]3[C@@H]1O |
| **11** | CC(C)(O)[C@H](O1)C[C@]23[C@]1([H])CC([C@@H](O)[C@H]2O3)=[C@]=CC(C)=C |
| **12** | CC(C)(O)[C@H](O1)C[C@]2(O)[C@]1([H])CC([C@@H](O)[C@@H]2Cl)=[C@]=CC(C)=C |
| **13** | CC(C)(O)[C@H](O1)C[C@]2(O)[C@]1([H])CC(C(C([H])C(C)(C)O3)=O)=C3[C@@H]2O |
| **14** | CC1(C)CC(C2=C(O1)C=C(C[C@H](C(C)(O)C)O3)C3=C2)=O |
| **15** | O[C@@H]1C(/C=C/CCCCC)=C(CO)[C@@]2([H])OC(C)(C)[C@H](O)C[C@@]2(O)C1[H] |
| **16** | O[C@@H]1C(/C=C/CCCCC)=C(CO)[C@@]2([H])OC(C)(C)[C@H](O)C[C@@]2(O)[C@H]1O |
| **17** | O[C@@H]1C(/C=C/CCCCC)=C(CO)[C@@]2([H])OC(C)(C)[C@H](O)C[C@@]23[C@@H]1O3 |
| **18** | O[C@@H]1C(/C=C/CCC([H])[C@@H](O)C)=C(CO)[C@@]2([H])OC(C)(C)[C@H](O)C[C@@]23[C@@H]1 O3 |
| **19** | O[C@@H]1C(/C=C/C[C@H](O)CCC)=C(CO)[C@@]2([H])OC(C)(C)[C@H](O)C[C@@]23[C@@H]1O3 |
| **20** | O[C@@H]1C(/C=C/[C@@H](O)CCCC)=C(CO)[C@@]2([H])OC(C)(C)[C@H](O)C[C@@]23[C@@H]1O3<br>or<br>O[C@@H]1C(/C=C/[C@H](O)CCCC)=C(CO)[C@@]2([H])OC(C)(C)[C@H](O)C[C@@]23[C@@H]1O3 |
| **21** | O[C@@H]1C(/C=C/[C@@H](O)CCCC)=C(CO)[C@@]2([H])OC(C)(C)[C@H](O)C[C@@]23[C@@H]1O3<br>or<br>O[C@@H]1C(/C=C/[C@H](O)CCCC)=C(CO)[C@@]2([H])OC(C)(C)[C@H](O)C[C@@]23[C@@H]1O3 |
| **22** | CCCCC/C=C/C1=C(CO)[C@@]2([H])OC(C)(C)[C@H](O)C[C@@]23[C@H](O3)[C@]1([H])OC(C)=O |
| **23** | CCCCC/C=C/C1=C(CO)[C@@]2([H])OC(C)(C)[C@H](O)C[C@@]23[C@H](O3)C1=O |
| **24** | O[C@@H]1C(/C=C/CCC(O)C([H])C)=C(CO)[C@@]2([H])OC(C)(C)[C@H](O)C[C@@]23[C@@H]1O3 |
| **25** | O[C@@H]1C(/C=C/C[C@@H](O)CCC)=C(CO)[C@@]2([H])OC(C)(C)[C@H](O)C[C@@]23[C@@H]1O3 |
| **26** | O[C@@H]1C(/C=C/CCCCC)=C(COC(C)=O)[C@@]2([H])OC(C)(C)[C@H](O)C[C@@]23[C@@H]1O3 |

**Table S2**. Results of cytotoxicity and antibacterial activity assay of compounds **1−26**.

| No. | Antibacterial activity (MIC, μM) | | | | | Cell growth (%) at 10 μg/mL | Cytotoxity ($IC_{50}$, μM) |
|---|---|---|---|---|---|---|---|
| | *A. baumannii* (BAA1605 ) | *A.baumannii* (BAA1605) + colistin (0.1 μM) | *P. aeruginosa* (27853) | *S. aureus* (29213) | *M. tuberculosis* (H37Rv) | Mouse lymphoma cell line L5178Y | |
| **1** | >100 | >100 | >100 | >100 | >100 | 58.5 | >20 |
| **2** | >100 | >100 | >100 | >100 | >100 | 65.4 | >20 |
| **3** | >100 | >100 | >100 | >100 | >100 | 64.8 | >20 |
| **4** | >100 | >100 | >100 | >100 | >100 | 77.4 | >20 |
| **5** | >100 | >100 | >100 | >100 | >100 | 64.3 | >20 |
| **6** | >100 | >100 | >100 | >100 | >100 | 68.3 | >20 |
| **7** | >100 | >100 | >100 | >100 | >100 | 67.1 | >20 |
| **8** | >100 | >100 | >100 | >100 | >100 | 77.8 | >20 |
| **9** | >100 | >100 | >100 | >100 | >100 | 53.0 | >20 |
| **10** | >100 | >100 | >100 | >100 | >100 | 56.1 | >20 |
| **11** | >100 | >100 | >100 | >100 | >100 | 76.5 | >20 |
| **12** | >100 | >100 | >100 | >100 | >100 | 66.7 | >20 |
| **13** | >100 | >100 | >100 | >100 | >100 | 77.5 | >20 |
| **14** | >100 | >100 | >100 | >100 | >100 | 67.2 | >20 |
| **15** | >100 | >100 | >100 | >100 | >100 | 54.4 | >20 |
| **16** | >100 | >100 | >100 | >100 | >100 | 53.2 | >20 |
| **17** | >100 | >100 | >100 | >100 | >100 | 70.5 | >20 |
| **18** | >100 | >100 | >100 | >100 | >100 | 64.8 | >20 |
| **19** | >100 | >100 | >100 | >100 | >100 | 78.1 | >20 |
| **20** | >100 | >100 | >100 | >100 | >100 | 70.1 | >20 |
| **21** | >100 | >100 | >100 | >100 | >100 | 63.6 | >20 |
| **22** | >100 | 50 | >100 | >100 | >100 | 44.2 | >20 |
| **23** | >100 | 100 | >100 | >100 | >100 | 1.90 | 3.0 |
| **24** | >100 | >100 | >100 | >100 | >100 | 80.0 | >20 |
| **25** | >100 | >100 | >100 | >100 | >100 | 77.4 | >20 |
| **26** | >100 | >100 | >100 | >100 | >100 | 65.7 | >20 |
